# Simple electrochemical synthesis of cyclic hydroxamic acids by reduction of nitroarenes†

**DOI:** 10.1039/d4cc02118e

**Published:** 2024-06-14

**Authors:** Johannes Winter, Susan Lühr, Kyra Hochadel, María de Jesús Gálvez-Vázquez, Tobias Prenzel, Dieter Schollmeyer, Siegfried R. Waldvogel

**Affiliations:** a Department of Chemistry, Johannes Gutenberg University Duesbergweg 10-14 55128 Mainz Germany; b Department of Chemistry, Faculty of Science, University of Chile Las Palmeras 3425 Ñuñoa 775000 Santiago Chile; c Institute of Biological and Chemical Systems – Functional Molecular Systems (IBCS-FMS), Karlsruher Institut für Technologie (KIT) Kaiserstraße 12 76131 Karlsruhe Germany; d Max Planck Institute for Chemical Energy Conversion (MPI-CEC), Stiftstrasse 34-36 45470 Mülheim an der Ruhr Germany siegfried.waldvogel@cec.mpg.de

## Abstract

The electrochemical reduction of nitroarenes allows direct access to manifold nitrogen containing heterocycles. This work reports the simple and direct electro-organic synthesis of 18 different examples of 2*H*,4*H*-4-hydroxy-1,4-benzoxazin-3-ones in up to 81% yield. The scalability of the method was demonstrated on a gram-scale.

## Abstract 2

A simple and scalable electrosynthesis of a broad scope of 4-hydroxy-1,4-benzoxazin-3-ones was developed by simple reduction of inexpensive nitroarenes.

## Graphical abstract

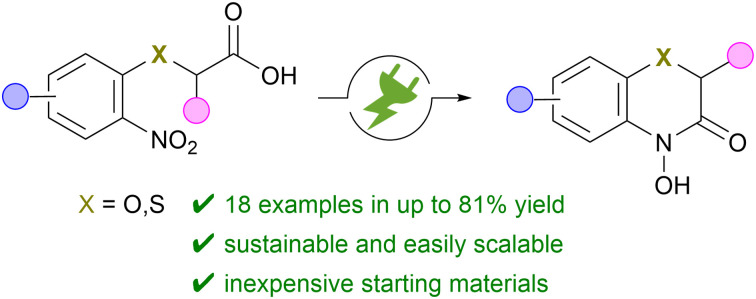

Nitrogen containing heterocycles are found in the majority of highly potent APIs and natural products.^1^ In particular, exocyclic N–O heterocycles are often highly bioactive key motifs in naturally occurring products.^2^ Thus, their synthesis plays a crucial role in modern organic chemistry by developing novel synthetic approaches to these compounds. 2*H*,4*H*-4-Hydroxy-1,4-benzoxazin-3-ones and their derivatives are found in natural products having herbicidal, fungicidal and therapeutic properties.^3^

1,4-Benzoxazin-3-ones are investigated as promising motifs in novel potassium channel modulators for treating high blood pressure (Schemes 1, 1).^4^ D-DIBOA (2) is naturally found in plants and has shown herbicidal and fungicidal activity and therefore could find application in agrochemistry.^5^ Furthermore, the corresponding pyridine derivative showed activity against a variety of bacterial strains leading to potential agents for treating bacterial infections (3).^6^ Additionally, the various activities against a broad range of pests highlights the significance as a highly promising motif for modern pharmaceutical and agrochemical research.^7^

**Scheme 1:**
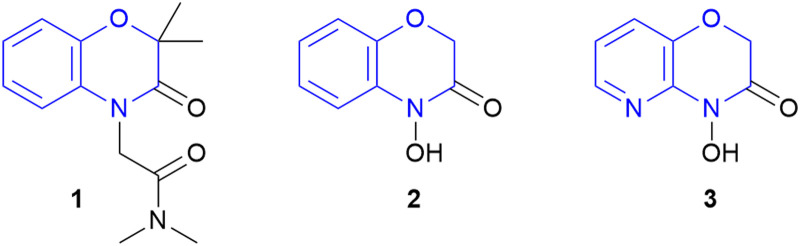
Important examples of 1,4-benzoxazin-3-ones: possible K^+^ channel modulator (1) D-DIBOA (natural product, 2) and a possible antibiotic (3).

A common and widely used strategy for the synthesis of N–O heterocycles is the selective reduction of nitroarenes, followed by cyclo-condensation. However, achieving a selective reduction remains quite challenging. The conventional approach for the selective synthesis of 2*H*,4*H*-4-hydroxy-1,4-benzoxazin-3-one requires the use of palladium catalysts (Scheme 2, A) or equimolar amounts of reducing agents (B).^8^*Tallec et al.* investigated the first potential controlled electro-synthesis in a divided cell using a mercury cathode (C).^9^ In addition, the electrolysis has only been demonstrated on a milli-gram-scale and no further scale-up was attempted. This approach is severely limited for the practical synthesis in modern laboratories. Modern research faces the challenge of meeting the growing need for innovative, sustainable methods to synthesize highly functional compounds. Organic electrochemistry is experiencing a renaissance as it fulfils several aspects of the principles of green chemistry.^10,11^ The use of electric current instead of hazardous redox reagents contributes to process safety. Moreover, the electrochemical reaction can be stopped by turning off the electrical current, preventing runaway reactions and enhancing the overall process safety.^12^ This method is in particular attractive when a suitable drown-stream processing is combined.^13^ Therefore, reduction and intramolecular cyclo-condensation of easily accessible and inexpensive nitroarenes offers a simple synthetic strategy for a large number of valuable fine chemicals and possible drug candidates.^14,15^ Based on previous work on the direct electrochemical reductive synthesis of N–O heterocycles, a synthetic protocol for the selective and scalable synthesis of 2*H*,4*H*-4-hydroxy-1,4-benzoxazin-3-ones was established, providing direct access to this highly promising class of compounds.^16–19^

**Scheme 2:**
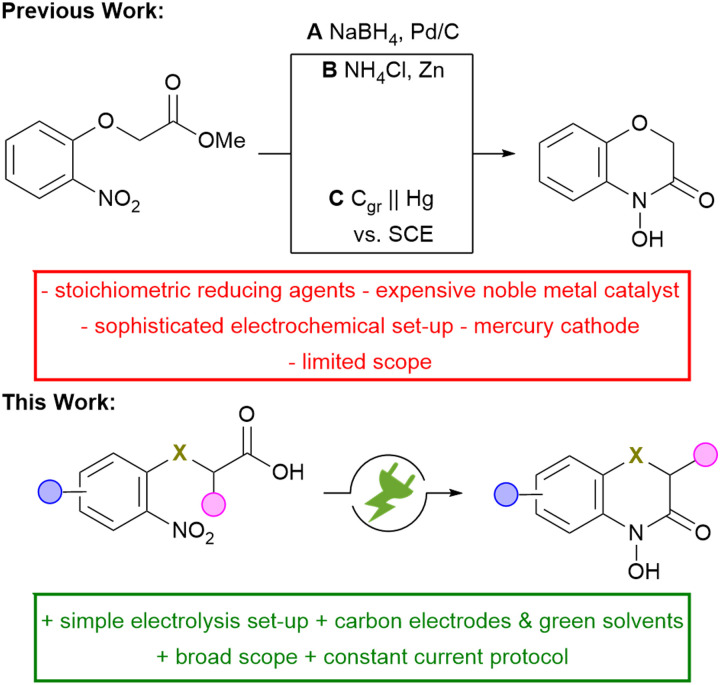
Conventional and electrochemical reduction of nitroarenes for the synthesis of 2*H*,4*H*-4-hydroxy-1,4-benzoxazin-3-ones; C_gr_ = graphite.

For the optimisation of the electrolytic conditions, methyl 2-(2-nitrophenoxy)acetate (6a) was prepared in a single step. The initial electrolytic conditions were chosen based on previous reductions.^16,18^ Sulphuric acid serves a dual role as a supporting electrolyte and catalyst in the cyclo-condensation by protonation.^20^ The best yields and selectivity were obtained using methanol and water as green solvents. Boron-doped diamond (BDD) has a high overpotential for the hydrogen evolution reaction, providing selectivity for the reduction of the nitro group, and shows no cathodic corrosion.^11,21^

Glassy carbon was chosen as the anode material as the counter reaction plays an important role in electrochemical transformations.^22^ The initial optimisation was performed using the theoretical amount of applied charge (4.0 *F*) and a current density of 3.7 mA cm^−2^. Initially, 5a was obtained in 31% NMR yield (Table 1, entry 1). The structure of the targeted 5a was confirmed by NMR spectroscopy and mass spectrometry after isolation. Additionally, the deoxygenated 1,4-benzoxazin-3-one 5s was prepared for comparison and verification of the analytical data used for NMR quantification (see ESI† for details). Alternatively, 5s can be prepared by Yoshida amination reaction.^23^

**Table 1:** Optimisation of the electrochemical synthesis of 2*H*,4*H*-4-hydroxy-1,4-benzoxazin-3-one (5a) under constant current conditions

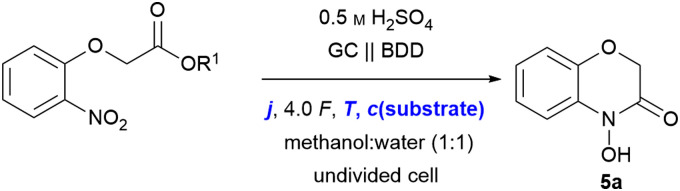					
Entry	R^1^	*j*/mA cm^−2^	*c*(substrate)	*T*/°C	Yield^a^ (%)
1	6a, Me	3.7	0.04 M	rt	31
2	6a, Me	5.2	0.04 M	rt	51
3	4a, H	3.7	0.04 M	rt	47
4	4a, H	3.7	0.04 M	50	22
5	4a, H	5.2	0.03 M	rt	60
6	4a, H	5.2	0.08 M	rt	26
7	4a, H	5.2	0.03 M	10	70 (60)^b^
8	6t, ^*t*^Bu	5.2	0.03 M	10	0

a Yield determined by ^1^H NMR; 1,3,5-trimethoxybenzene as internal standard.

b Isolated yield; BDD = boron-doped diamond; GC = glassy carbon.

By increasing the current density (*j*) to 5.2 mA cm^−2^, the yield increased up to 51% (entry 2). To our delight, the less electrophilic carboxylic acid 4a gave the product in 47% (Table 1, entry 3) yield by applying 3.7 mA cm^−2^. Moreover, the influence of the reaction temperature was investigated. It was hypothesized that elevated temperatures could accelerate the cyclo-condensation by promoting water elimination. However, increasing the reaction temperature from rt to 50 °C resulted in a significant drop of the yield to 22% (entry 4), while no starting material was detected after electrolysis. As expected for intramolecular cyclisations, the substrate concentration was found to be crucial, since lower concentrations can suppress intermolecular side reactions. An optimum substrate concentration of 0.03 M in combination with the increased current density of 5.2 mA cm^−2^ yielded 60% of the desired 5a (entry 5), whilst the yield dropped drastically to 26% (entry 6) when higher substrate concentrations of 0.08 M were used. On the contrary, through decreasing the temperature to 10 °C during electrolysis, the yield of 5a was further increased to 70% NMR yield (60% isolated yield, entry 7). *tert*-Butyl esters are known to hydrolyse easily under acidic conditions, which might be beneficial for the cyclo-condensation. However, no product was obtained when using *tert*-butyl ester 6t as a substrate and solely degradation of the substrate was observed (entry 8). Further variation of the electrode materials and additives did not increase the yield of 5a (see ESI† for further details).

The scope of the reaction was investigated with the following optimised conditions: glassy carbon (anode) and BDD (cathode) were used. 0.5 M sulphuric acid in a methanol : water (1 : 1) mixture was used as an electrolyte while applying a current density of 5.2 mA cm^−2^ in an undivided cell (Scheme 3).

**Scheme 3:**
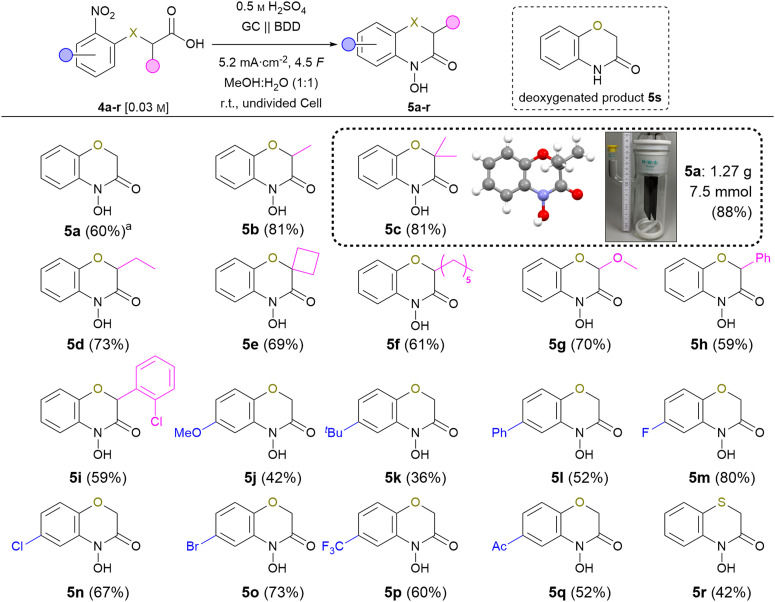
Scope of the electrochemical synthesis of 2*H*,4*H*-4-hydroxy-1,4-benzoxazin-3-ones, isolated yields. ^*a* ^Temperature: 10 °C.

Despite the fact that reaction optimisation using test substrate 4a indicated an optimum reaction temperature of 10 °C, most of the nitro-derivatives applied for investigation of the scope showed limited solubility under these conditions. Since this resulted in side-reactions significantly lowering the product yield, carrying out these syntheses at room temperature proved beneficial due to tremendously increased solubility. Furthermore, an amount of applied charge of 4.5 *F* was used to ensure full conversion of all substrates. First, the effect of a substitution in position 3 was studied. The 3-methyl substituted 5b and 5c were isolated in 81% yield. The corresponding ethyl substituted 5d was obtained in 73% yield. The increased yield compared to the standard product is a result of the *Thorpe–Ingold* effect promoting the cyclisation, which is a key-step of the overall reaction.^24^ Even spiro derivative 5e was obtained in 69% yield. The *n*-hexyl modified compound 5f was isolated in 61% yield. The sterical demand of long alkyl chains might counter the *Thorpe–Ingold* effect previously observed inhibiting the cyclisation. 5g represents a methoxylated variant of the natural compound D-DIBOA (2) and was isolated in 70% yield avoiding the use of noble metals.^25^ Derivatives 5h and 5i bearing an aryl-substituent were isolated in 59% yield each. The slightly decreased yields are most likely a result of steric effects as seen for the alkyl derivatives (5b–5f) yield. Afterwards, variation of the nitroarene was investigated. The electron-rich 5j and 5k were obtained in 41% and 36% yield, respectively. Generally, the reduction of electron-rich arenes is less favoured. The phenyl substituted derivative 5l was obtained in 52% yield.

A great tolerance for halogens was observed and 5m–o were isolated in up to 80% yield (F: 80% > Br: 73% > Cl: 67%).^26^ To underline the method's application potential, trifluoromethyl-substituted derivative 5p was isolated in 60% yield. The presence of trifluoromethyl groups in modern pharmaceuticals highlights the importance of tolerating this functional group during electrolysis.^27^ The electron-deficient derivative 5q with an acetyl group was isolated in 52% yield. Notably, no byproducts from intermolecular condensation were observed, highlighting the selectivity for the intramolecular product. Implementation of sulphur atoms in electrochemical reactions is a challenge due to their sensitivity of undergoing oxidation. To our delight, even 4-hydroxybenzo-1,4-thiazin-3-one (5r) was successfully obtained in 40% yield in an undivided cell. No oxidation of the sulphur could be observed, which emphasises that the methodology also tolerates oxidation-sensitive functional groups. Last, the scalability of the reaction was demonstrated employing derivative 5c. On gram-scale, the product was obtained in an outstanding yield of 88% (6.6 mmol, 1.27 g), using an undivided beaker-type electrolysis cell as the simplest set-up. Moreover, product purification was achieved solely *via* simple crystallisation. This underlines the application potential of this method as it allows for efficient product isolation and downstream processing. Furthermore, the molecular structure of this compound was verified by X-ray analysis (CCDC: 2349053†).

Mechanistic studies of the acids 4a and 4c and the corresponding 4-hydroxy-1,4-benzoxazin-3-ones 5a and 5c by cyclic voltammetry (CV) were conducted (for details see ESI†). The acids 4a and 4c showed a broad reductive wave (−1.00 V *vs.* FcH/FcH^+^) relating to the 4e^−^/4H^+^ reduction of the nitroarene to the intermediary formed hydroxylamine, which is reported in the literature (Scheme 4).^15,17,18^ Interestingly, only 4a showed a weak oxidative wave (+0.41 V *vs.* FcH/FcH^+^) related to the reoxidation of the hydroxylamine to the nitroso arene. The 4-hydroxy-1,4-benzoxazin-3-ones 5a and 5c both showed two oxidative waves, which correspond to a N–O radical formed, which might later be oxidized to a cation.^9,28^ This literature-known step might be the major side reaction resulting in methoxylated side products when applying higher current densities, which were observed during optimisation by LC-MS. However, this might give rise to another field of application, as the N–O radical formed could potentially be used as a mediator, similar to nitroxyl compounds such as TEMPO.^29^

**Scheme 4:**
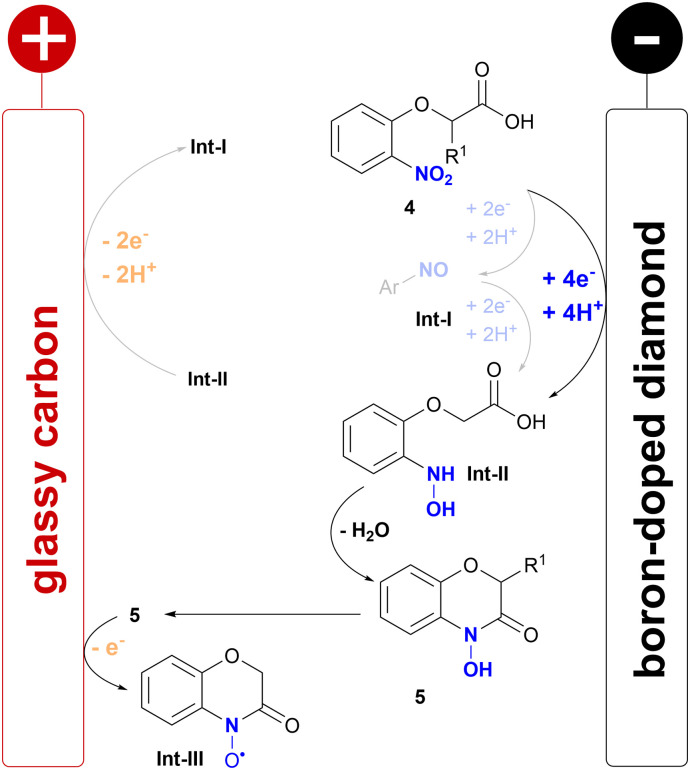
Proposed mechanism of the electrochemical synthesis of 4-hydroxybenzoxazinones.

In summary, a simple and easily scalable electrochemical protocol has been established, enabling direct access to a wide range of highly functionalised 2*H*,4*H*-4-hydroxy-1,4-benzoxazin-3-ones. The simplicity of the protocol was underlined by using widely available 2-nitrophenols as inexpensive starting materials. Compared to the established electrochemical protocols, simple constant current conditions were applied instead of complex constant potential conditions where an additional reference electrode is needed. The toxic mercury electrodes were avoided and a broad scope of 18 examples was prepared in up to 81% yield, tolerating electrochemically sensitive functional groups.

Moreover, the scalability was demonstrated in up to gram-scale, without any loss in yield, and the product was purified by a simple crystallisation. Compared to classical methods, equimolar amounts of reductants were avoided diminishing the amount of generated waste.

J. W. conceived the project together with S. R. W., J. W., K. H., S. L., M. G. and T. P. conducted the experiments and interpreted and analysed the results. D. S. performed the X-ray analysis. J. W., S. L. and S. R. W. wrote and reviewed the manuscript. S. R. W. supervised the project. All authors discussed the results and agreed to the manuscript.

We wish to acknowledge financial support from the Deutsche Forschungsgemeinschaft (DFG WA1276/17-2 and WA1276/31-1), the Swiss National Science Foundation (SNSF, P500PN_210727) and the German Academic Exchange Service (DAAD 57681226). Open Access funding provided by the Max Planck Society.

## Conflicts of interest

There are no conflicts to declare.

## Supplementary Material

CC-060-D4CC02118E-s001

CC-060-D4CC02118E-s002
