# Supplementary material for: Simple electrochemical synthesis of cyclic hydroxamic acids by reduction of nitroarenes
Source: Chem Commun (Camb). 2024 Jun 14;60(55):7065–8. doi: 10.1039/d4cc02118e (PMC11223186; doi:10.1039/d4cc02118e)
Supplement: CC-060-D4CC02118E-s001 [file CC-060-D4CC02118E-s001.pdf]

Electronic Supplementary Material (ESI) for ChemComm.
This journal is © The Royal Society of Chemistry 2024

Electronic Supporting Information (ESI)

Simple Electrochemical Synthesis of Cyclic Hydroxamic Acids by Reduction of Nitro Arenes

Johannes Winter, Susan Lühr, Kyra Hochadel, María de Jesús Gálvez-Vázquez, Tobias Prenzel, Dieter Schollmeyer and Siegfried R. Waldvogel

1. General Information 2
2. General Protocols 6
2.1. General Protocol for the Synthesis of Substituted 2-Nitrophenoxyaceticacid esters (GPI) 6
2.2. General Protocol for the Synthesis of Substituted 2-Nitrophenoxyacetic acids (GPII) 6
2.3. General Protocol for the Electrochemical Synthesis of substituted 2*H*,4*H*-4-Hydroxybenzo[*b*]-1,4-oxazin-3-one (GPIII) 6
3. Optimization of the Electrolytic Conditions 8
4. Scale-up of the Electrochemical Reductive Synthesis of 2*H*,4*H*-2,2-Dimethyl-4-hydroxybenzo[*b*]-1,4-oxazin-3-one (5c) 12
5. CV Studies 13
6. Preparation of Products and Analytical Data 15
6.1. 2-Nitrophenoxyaceticacid esters (6a–6t) 15
6.2. 2-Nitrophenoxyacetic acid (4a-4r) 20
6.3. 4-Hydroxybenzo[*b*]-1,4-oxazin-3-ones (5a-5r) 25
7. Crystallographic Data 31
8. NMR Spectra 33
9. References 93

1. General Information

If not stated otherwise, all reactions were performed under ambient conditions and chemicals in analytical grade were used as purchased without further purification. Cyclohexane and ethyl acetate were purchased in technical grade and purified by distillation under reduced pressure prior to use. Milli-Q® water was obtained using Simplicity® System (UV) (*Merck KGaA*, Darmstadt, Germany) for chromatography purposes. Anhydrous solvents were prepared by a solvent purification system SPS-5 (*M. Braun Incorporated*, Stratham, USA).

Chromatography

Thin layer chromatography was performed using DC silica gel 60 F254 on aluminum plates (*Merck KGaA,* Darmstadt, Germany). A UV lamp (λ = 254 nm, NU-4 KL, *Benda*, Wiesloch, Germany) was used for detection. Preparative flash column chromatography was performed on silica gel 60 M (0.040–0.063 mm, 12 g or 80 g, Macherey-Nagel GmbH & Co, Düren, Germany) using a Büchi Pure C-815 Flash (*Büchi-Labortechnik GmbH*, Essen, Germany). Reversed phase column chromatography of the different products was performed on a Puriflash® PF-30C18HP-F0080 (*Interchim SAS*, Montluçon Cedex, France) column using a Sepacore® system with a Büchi Control Unit C-620, Büchi Pump Modules C-605, a UV detector Büchi UV photometer C-635, and Büchi Fraction Collector C-660 (*Büchi-Labortechnik GmbH*, Essen, Germany) using different mixtures of water (0.1% formic acid (*v/v*)) and acetonitrile as eluents.

High Resolution Mass Spectrometry

Mass spectra *via* electrospray-ionization (ESI+/ESI–) mass spectrometry were recorded using an Agilent 6545 QTOF-MS (*Agilent*, Santa Clara (CA), USA). Mass-charge ratios (*m*/*z*) were obtained for the characterized compounds.

X-ray Crystallography

The measurements of the crystal structures were carried out on a STOE IPDS-2T (*STOE & Cie GmbH*, Darmstadt, Germany) using a Mo source with graphite tube monochromator.

High Performance Liquid Chromatography (HPLC)

Analysis of crude reaction mixtures, purified products and method development was performed using a modular system LC-20A *Prominence* (*Shimadzu Deutschland GmbH*, Duisburg, Germany), UV/VIS-detector SPD-20A/AV (*Shimadzu Deutschland GmbH*, Duisburg, Germany), and LCMS-2020 Single Quadrupole (*Shimadzu Deutschland GmbH*, Duisburg, Germany). Analytical separation was performed using an Eurospher II 100-5 C-18-Trennsäule (*Knauer Wissenschaftliche Geräte GmbH*, Berlin, Germany) column (length of 150 mm, diameter of 4 mm, pore size of 100 Å, particle size 5 µm). As eluents, acetonitrile, and water with 5% (*v/v*) acetonitrile and formic acid (0.1% (*v/v*)) were used. Given retention times were obtained at λ = 254 nm.

Nuclear Magnetic Resonance (NMR) Spectroscopy

Nuclear magnetic resonance experiments were performed using a nuclear magnetic resonance spectrometer Avance III HD300 (*Bruker*, Karlsruhe, Germany) ^{1}H NMR (300 MHz), ^{19}F NMR (282 MHz), Avance II 400 (*Bruker*, Karlsruhe, Germany) ^{1}H NMR (400 MHz), ^{13}C NMR (101 MHz), ^{19}F NMR (376 MHz) and Avance III 600 (*Bruker*, Karlsruhe, Germany) ^{1}H NMR (600 MHz), ^{13}C NMR (151 MHz). The spectra were recorded using deuterated solvents. To normalize the spectra obtained, reference was made to the existing solvent signal of non-deuterated fractions according to the data provided by *Fulmer et al.*:[1] $CDCl_3$ (^{1}H NMR: δ = 7.26 ppm, ^{13}C NMR: δ = 77.2 ppm), dichloromethane-d_2 (^{1}H NMR: δ = 5.32 ppm, ^{13}C NMR: δ =53.8 ppm), acetonitrile-d_3 (^{1}H NMR δ = 1.94 ppm, ^{13}C NMR: δ = 118.3 ppm), CD_3OD (^{1}H NMR δ = 3.31 ppm, ^{13}C NMR: δ =49.0 ppm) and DMSO$_{d6}$ (^{1}H NMR δ = 2.50 ppm, ^{13}C NMR: δ =39.5 ppm). Besides ^{1}H, ^{13}C and ^{19}F NMR experiments, the 2D techniques ^{1}H,^{1}H COSY, ^{1}H,^{13}C HSQC and ^{1}H,^{13}C HMBC were used assisting to assign the signals. The following abbreviations were used to describe the signals: s (singlet), d (doublet), t (triplet), dd (doublet of doublets), td (triplet of doublets), m (multiplet), q (quartet), sep (septet), ddd (doublet of doublets of doublets). The spectra obtained were evaluated with MestReNova 14.2.0-26256 (*Mestrelab Research S.L.*, Spain).

Melting Points

Melting points of crystallised products were determined using a M-565 (*Büchi Labortechnik*, Essen, Germany) with a heating rate of 1 °C/min. The melting points are reported uncorrected.

Cyclic Voltammetry (CV) Measurements

The mechanism of the reaction was studied by cyclic voltammetry using an electrochemical glass cell (figure S1) equipped with a BDD tip electrode (with a diameter of 4 mm), a boron-doped diamond disc (diameter: 2 mm), glassy carbon rod and a Ag/AgCl (saturated LiCl in ethanol, *Metrohm AG*, Herisau, Switzerland) as working, counter and reference electrode, respectively. The electrode potentials are reported with reference to the redox system ferrocene/ferrocenium (FcH/FcH$^+$).
Cyclic voltammograms were measured using a potentiostat/galvanostat PGSTAT302N (*Metrohm AG*, Herisau, Switzerland) with a scan rate of 50 mV s^{-1} in a methanol (HPLC LC-MS grade, *VWR International GmbH*, Darmstadt, Germany) and water solution (1:1 v/v) containing 0.5 M of H_2SO_4 (analytical reagent grade, *Fisher Scientific GmbH*, Schwerte, Germany) and 5 mM of the corresponding molecule. Prior to the CV measurements, the electrolyte was degassed with an argon flow for 25 min. An argon flow was kept flowing over the electrolyte during the measurements.

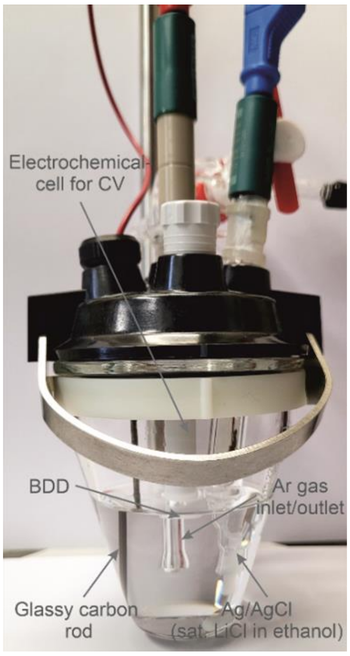

Figure S1: Electrochemical cell for cyclic voltammetry measurements.

Electrode Materials

Electrode material, purity, and their supplier are listed in table S1. Boron-doped diamond (BDD) electrodes were treated prior to electrosynthesis in 20% aqueous sulfuric acid (10 C·cm^{-2}) with current density of 10 mA·cm^{-2} by polarizing subsequently anodically and then cathodically. After the treatment, the cathode was rinsed with water, methanol, and dried. Lead and leaded bronze CuSn7Pb15 were polished with sandpaper (600 grit and 1000 grit), rinsed with water and methanol and dried. Isostatic graphite was polished with sandpaper (1000 grit), rinsed with methanol and dried.

Table S1: Electrode materials, purity, and their supplier.

Entry	Electrode Material	Purity	Supplier
1	Boron-doped diamond (DIACHEM™)	15 µm boron-doped diamond layer on silicon	*CONDIAS GmbH*, Itzehoe, Germany
2	Glassy carbon (Sigradur G)	-	*HTW*, Thierhaupten, Germany
3	Isostatic graphite (V2100)	-	*SGL Carbon*, Bonn, Germany
4	Lead	-	*Globus Fachmärkte GmbH & Co. KG,* Völklingen, Germany
5	Leaded bronze (CuSn7Pb15 and CuSn7ZnPb7)	-	*Metallwerk Langenau GMBH,* Langenau, Germany
6	Dimensionally Stable Anodes (DSA) (Ru/Ir)O2 on Ta	-	*DeNora,* Mailand, Italy
7	Stainless Steel (1.4571)	-	*Montanstahl GmbH,* Oelde, Deutschland
8	Zinc	-	*Grillo-Werke AG,* Duisburg, Germany
9	Reticulated Vitreous Carbon (RVC)	-	*ERG Aerospace Corporation*, USA

Electrochemical Set-Up

Electrochemical reactions were carried out using a multichannel galvanostat HMP4040 (*Rohde & Schwarz*, München, Germany). The different cells used for screening or batch reactions are described below.

Screening Reactions

Teflon™ cells with a volume of 5 mL were used for the undivided set-up (figure S2, left). Divided Teflon™ screening cells with a volume of 7 mL were equipped with a glass frit as separator material as shown (figure S2, right). Glass frits used as separator materials were pre-treated in the corresponding electrolyte prior to use. Stirring bars were used during electrolysis in each cell. The described screening systems are commercially available as IKA Screening System Package (*IKA™ Werke GmbH & Co. KG*, Staufen, Germany).

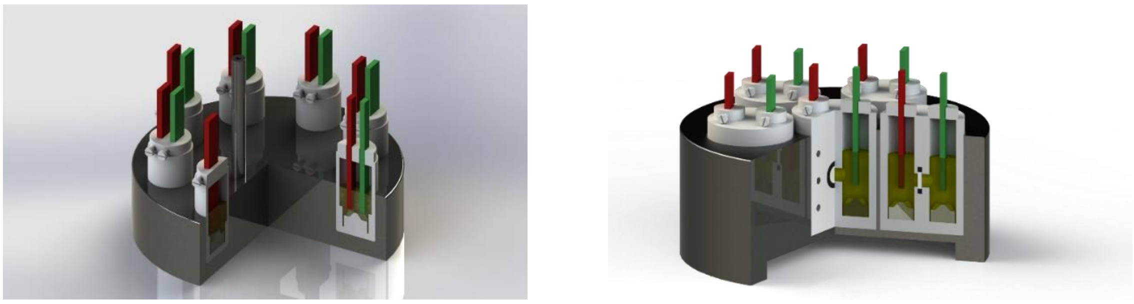

Figure S2: Undivided screening set-up (left) and divided screening set-up (right).[2]

Scale-up Reactions

Scale-up experiments were performed in undivided 25 mL, 100 mL and 250 mL batch-type cells with a PTFE stopper and sleeve, electrodes, and electrode holders (figure S2). A TDK-Lambda Z+ series (*TDK-Lambda UK Limited*, Devon, United Kingdom) or a multichannel power supply HMP4040 (*Rohde & Schwarz*, München, Germany) were used as power sources. In the undivided 5 mL electrolysis set-up glassy carbon and BDD electrodes with dimensions of 7 cm·1 cm were used. In the undivided 25 mL and 100 mL electrolysis set-up glassy carbon and BDD electrodes with identical dimensions of 6 cm·2 cm were used. In the undivided 250 mL electrolysis set-up glassy carbon and BDD electrodes with identical dimensions of 12 cm·4 cm were used.

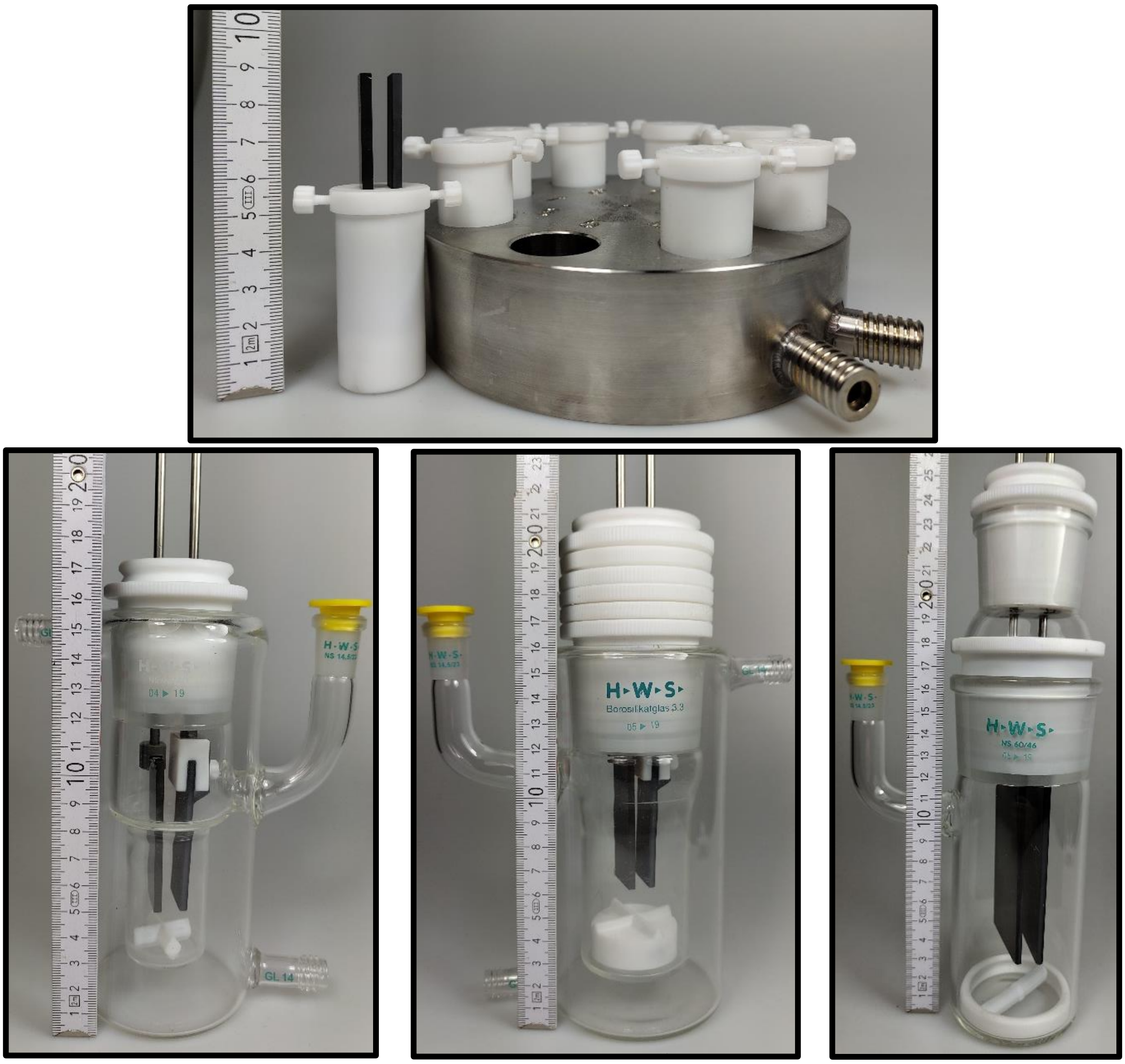

Figure S3: Different batch-type cells; size compared to a ruler: top: 5 mL undivided Teflon™ screening cells with glassy carbon and BDD electrodes and screening cell holder with integrated radiator loop; bottom left: 25 mL undivided glass cell with glassy carbon and BDD electrodes; bottom centre: 100 mL undivided glass cell with glassy carbon and BDD electrodes; bottom right: 300 mL divided glass cell with glassy carbon and BDD electrodes.

2. General Protocols

2.1. General Protocol for the Synthesis of Substituted 2-Nitrophenoxyaceticacid esters (GPI)

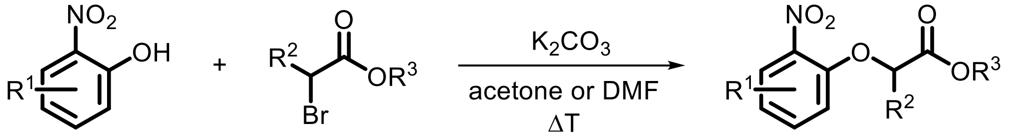

Scheme S1: Synthesis of substituted 2-nitrophenoxyaceticacid esters.

Under argon atmosphere potassium carbonate (2.0-3.0 eq.) was suspended in anhydrous acetone and degassed for 15 min. The corresponding 2-nitrophenol (1.0 eq.) was added and the reaction mixture was stirred for 10 min at room temperature. Afterwards the corresponding 2-bromoacetate (1.0–2.0 eq.) was added and the reaction mixture was stirred under reflux until completion of the reaction (TLC). The crude mixture was filtered, and the acetone was removed under reduced pressure. 50 mL of water and 50 mL of ethyl acetate were added, and the fractions were separated. The aqueous fraction was extracted three times with 50 mL of ethyl acetate. The combined organic fractions were washed twice with saturated sodium carbonate solution to remove residual phenol. Afterwards the organic fractions were washed once with 50 mL of brine, dried over sodium sulphate and the solvent was removed under reduced pressure. The crude product was purified by crystallisation or flash column chromatography.

2.2. General Protocol for the Synthesis of Substituted 2-Nitrophenoxyacetic acids (GPII)

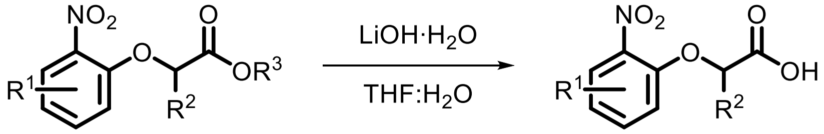

Scheme S2: Synthesis of substituted 2-nitrophenoxyacetic acids.

The corresponding 2-nitrophenoxyaceticacid ester (1.0 eq.) was dissolved in THF at room temperature. Lithium hydroxide monohydrate (1.5 eq.) was dissolved in water and added in one portion to the reaction mixture. The solution was stirred at room temperature until completion of the reaction (TLC). Afterwards the solution was neutralized with 1 M hydrochloric acid. The THF was removed under reduced pressure and the aqueous fraction was extracted three times with 50 mL dichloromethane. The combined organic fractions were washed once with 50 mL of brine, dried over sodium sulphate and the solvent was removed under reduced pressure. If necessary, the crude product was further purified by crystallisation or flash column chromatography.

2.3. General Protocol for the Electrochemical Synthesis of substituted 2*H*,4*H*-4-Hydroxy-benzo[*b*]-1,4-oxazin-3-one (GPIII)

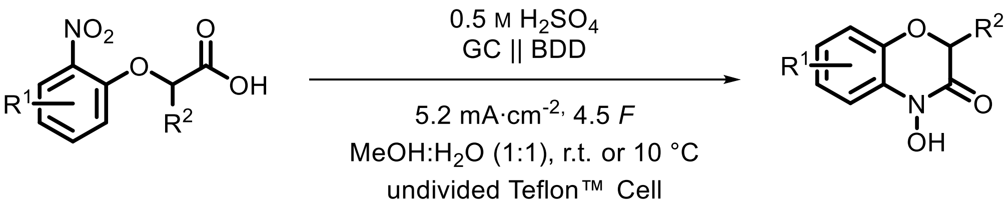

Scheme S3: Synthesis of substituted 2-nitrobenzenesulfonamides.

5 mL Undivided Teflon™ Screening Cell: 0.15 mmol of the starting material was dissolved in 2.5 mL of methanol in the undivided cell and 2.5 mL of 1.0 M sulphuric acid was added. The electrodes (1 cm·7 cm) immersed 1.5 cm into the solution resulting in an area of 1.5 cm^{-2}. Prior to the electrolysis the solution was cooled to 10 °C, if necessary. The electrolysis was performed under constant current conditions (current density j = 5.2 $mA{\cdot}cm^{-2}$ for 65.1 C (4.5 *F*)). After the electrolysis the reaction mixture was diluted with 5 mL of water and 5 mL of brine and extracted three times with 10 mL of ethyl acetate. The aqueous fraction was analysed via LC-MS to ensure the complete extraction of the product. If necessary, the aqueous fraction was extracted additionally twice with 10 mL of ethyl acetate. The combined organic fractions were washed once with 5 mL of brine, dried over sodium sulphate and the solvent was removed under reduced pressure. The crude product was purified with reverse phase column chromatography (C_{18}).

25 mL Undivided Glass Cell: 0.75 mmol of the starting material was dissolved in 12.5 mL of methanol in the undivided cell and 12.5 mL of 1.0 M sulphuric acid was added. The electrodes (2 cm·6 cm) immersed 3.0 cm into the solution resulting in an area of 6.0 cm^{-2}. The electrolysis was performed under constant current conditions (current density j = 5.2 mA·cm^{-2} for 325.6 C (4.5 F)). After the electrolysis the reaction mixture was diluted with 15 mL of water and 15 mL of brine and extracted three times with 30 mL of ethyl acetate. The aqueous fraction was analysed via LC-MS to ensure the complete extraction of the product. If necessary, the aqueous fraction was extracted additionally twice with 30 mL of ethyl acetate. The combined organic fractions were washed once with 15 mL of brine, dried over sodium sulphate and the solvent was removed under reduced pressure. The crude product was purified with reverse phase column chromatography (C_{18}).

100 mL Undivided Glass Cell: 3.0 mmol of the starting material was dissolved in 50 mL of methanol in the undivided cell and 50 mL of 1.0 M sulphuric acid was added. The electrodes (2 cm·6 cm) immersed 3.0 cm into the solution resulting in an area of 6.0 cm^{-2}. The electrolysis was performed under constant current conditions (current density j = 5.2 mA·cm^{-2} for 1302.6 C (4.5 F)). After the electrolysis the reaction mixture was diluted with 25 mL of water and 25 mL of brine and extracted three times with 30 mL of ethyl acetate. The aqueous fraction was analysed via LC-MS to ensure the complete extraction of the product. If necessary, the aqueous fraction was extracted additionally twice with 30 mL of ethyl acetate. The combined organic fractions were washed once with 25 mL of brine, dried over sodium sulphate and the solvent was removed under reduced pressure. The crude product was purified with reverse phase column chromatography (C_{18}).

250 mL Undivided Glass Cell: 7.5 mmol of the starting material was dissolved in 125 mL of methanol in the undivided cell and 125 mL of 1.0 M sulphuric acid was added. The electrodes (4 cm·13 cm) immersed 8.0 cm into the solution resulting in an area of 32.0 cm^{-2}. The electrolysis was performed under constant current conditions (current density j = 5.2 mA·cm^{-2} for 3256.4 C (4.5 F)). After the electrolysis the reaction mixture was diluted with 50 mL of water and 50 mL of brine and extracted three times with 50 mL of ethyl acetate. The aqueous fraction was analysed via LC-MS to ensure the complete extraction of the product. If necessary, the aqueous fraction was extracted additionally twice with 50 mL of ethyl acetate. The combined organic fractions were washed once with 50 mL of brine, dried over sodium sulphate and the solvent was removed under reduced pressure. The crude product was purified by crystallisation.

Note: *It is advisable to use sodium sulphate as a drying agent as we observed more consistent yields compared to magnesium sulphate. We suspect a complexation of* Mg^{2+} *by the 2H,4H-4-Hydroxybenzo[b]-1,4-oxazin-3-ones. Furthermore, by using glassware it is possible to form metal complexes which can be observed by a strong colouration of the product. To avoid this problem, it is necessary to use formic acid as an additive during column chromatography.*

3. Optimization of the Electrolytic Conditions

Table S2: Optimisation of the electrolytic reaction conditions for the synthesis of 2*H*,4*H*-4-hydroxybenzo[*b*]-1,4-oxazin-3-one (5a).

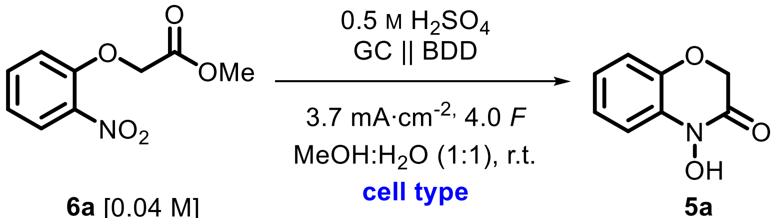

6a [0.04 M] **5a**

Entry	Cell Type	Yield[a] [%]
1	undivided	31
2	divided	20

[a]Yield determined by ^{1}H NMR, internal standard: 1,3,5-Trimethoxybenzene.

Table S3: Optimisation of the electrolytic reaction conditions for the synthesis of 2*H*,4*H*-4-hydroxybenzo[*b*]-1,4-oxazin-3-one (5a).

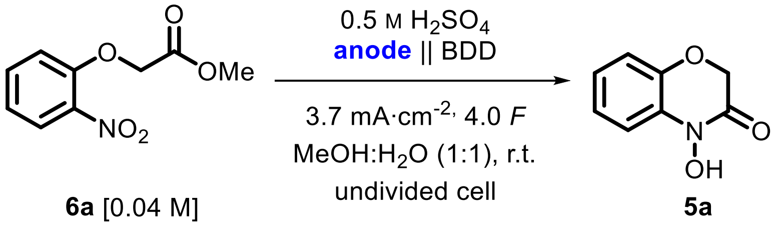

6a [0.04 M] **5a**

Entry	Anode Material	Yield[a] [%]
1	graphite	12
2	glassy carbon	31
3	BDD	10
4	RVC	2
5	stainless steel (1.4571)	5
6	DSA $(Ru/Ir)O_2$ on Ta	28

[a]Yield determined by ^{1}H NMR, internal standard: 1,3,5-Trimethoxybenzene.

Table S4: Optimisation of the electrolytic reaction conditions for the synthesis of 2*H*,4*H*-4-hydroxybenzo[*b*]-1,4-oxazin-3-one (5a).

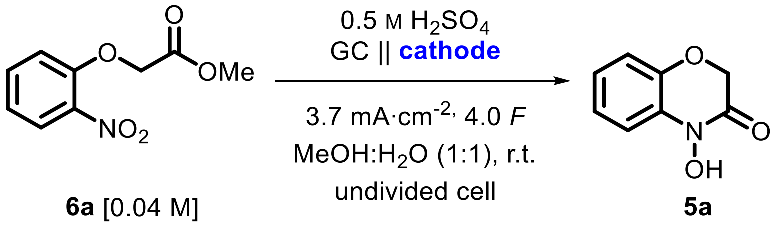

6a [0.04 M] **5a**

Entry	Cathode Material	Yield[a] [%]
1	graphite	24
2	glassy carbon	23
3	BDD	31
4	RVC	24
5	Zn	0[b]
6	Pb	3
7	CuSn7Pb15	13
8	CuSn7Zn4Pb7	10

[a]Yield determined by ^{1}H NMR, internal standard: 1,3,5-Trimethoxybenzene; [b]repeated twice, full reduction without electricity observed.

Table S5: Optimisation of the electrolytic reaction conditions for the synthesis of 2*H*,4*H*-4-hydroxybenzo[*b*]-1,4-oxazin-3-one (5a).

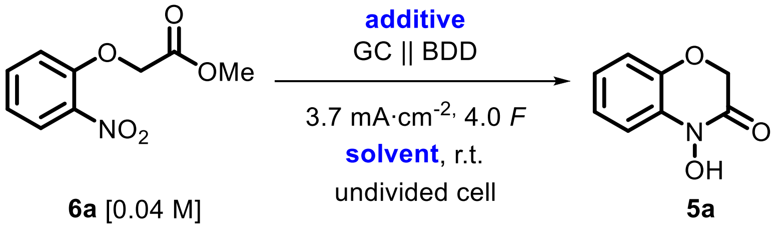

6a [0.04 M] **5a**

Entry	Additive/Solvent	Yield[a] [%]
1	0.5 M H_2SO_4 in methanol:water (1:1)	31
2	0.5 M H_2SO_4 in methanol	4
3	0.05 M H_2SO_4 in methanol	0
4	0.5 M $MeSO_3H$ in methanol	0
5	0.05 M $MeSO_3H$ in methanol	0
6	0.01 M Bu_4NBF_4 in methanol	0
7	0.025 M H_2SO_4+0.25 M NH_4COO in methanol	0
8	0.5 M AcOH+0.05 M NaOAc in methanol	3
9	0.5 M H_2SO_4 in methanol+5 vol% water	5
10	0.5 M H_2SO_4 in methanol+10 vol% water	9
11	0.5 M H_2SO_4 in methanol+20 vol% water	27
12	0.5 M H_2SO_4 in methanol+30 vol% water	19
13	0.5 M H_2SO_4 in methanol+40 vol% water	23

[a]Yield determined by ^{1}H NMR, internal standard: 1,3,5-Trimethoxybenzene.

Table S6: Optimisation of the electrolytic reaction conditions for the synthesis of 2*H*,4*H*-4-hydroxybenzo[*b*]-1,4-oxazin-3-one (5a).

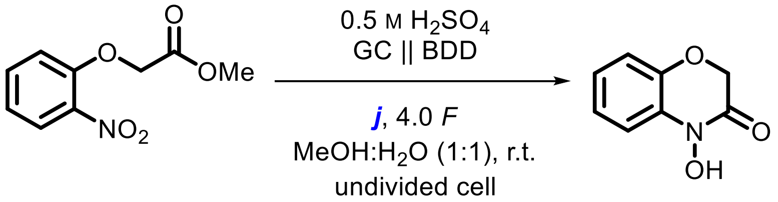

6a [0.04 M] **5a**

Entry	*j* = Current Density / mA·cm^{-2}	Yield[a] [%]
3	3.7	31
4	4.2	29
5	4.7	29
6	5.2	30
7	5.7	35

[a]Yield determined by ^{1}H NMR, internal standard: 1,3,5-Trimethoxybenzene.

Table S7: Optimisation of the electrolytic reaction conditions for the synthesis of 2*H*,4*H*-4-hydroxybenzo[*b*]-1,4-oxazin-3-one (5a).

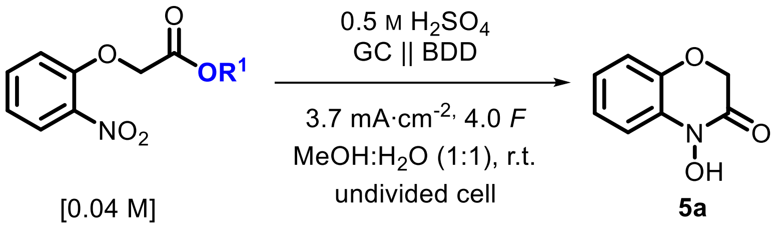

[0.04 M] **5a**

Entry	Carbonyl Motif	Yield[a] [%]
1	**6a** R^1 = Me	31
2	**4a** R^1 = H	47
3	**6s** R^1 = Et	6
4	**6t** R^1 = *t*-Bu	0[b]

[a]Yield determined by ^{1}H NMR, internal standard: 1,3,5-Trimethoxybenzene; [b]Low solubility of the substrate, 0.03 M of the substrate was used.

Table S8: Optimisation of the electrolytic reaction conditions for the synthesis of 2*H*,4*H*-4-hydroxybenzo[*b*]-1,4-oxazin-3-one (5a).

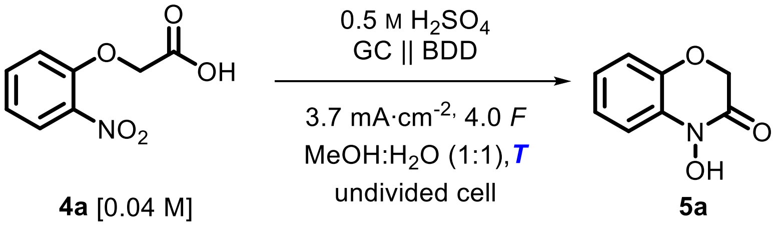

Entry	Temperature / °C	Yield[a] [%]
1	10	70 [b] (60)[c]
2	r.t.	47
3	30	42
4	40	39
5	50	22

[a]Yield determined by ^{1}H NMR, internal standard: 1,3,5-Trimethoxybenzene; [b]Low solubility of the substrate, 0.03 M of the substrate was used; [c]isolated yield.

Table S9: Optimisation of the electrolytic reaction conditions for the synthesis of 2*H*,4*H*-4-hydroxybenzo[*b*]-1,4-oxazin-3-one (5a).

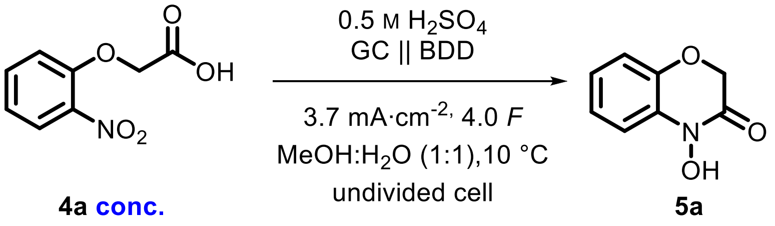

Entry	Substrate Concentration	Yield[a] [%]
1	0.03 M	70 [b] (60)[c]
2	0.05 M	42
3	0.06 M	36
4	0.08 M	26

[a]Yield determined by ^{1}H NMR, internal standard: 1,3,5-Trimethoxybenzene; [b]Low solubility of the substrate, 0.03 M of the substrate was used; [c]isolated yield.

NMR Quantification

After work-up of the crude reaction mixture according to general protocol III (**GPIII**) 0.1 mmol (16.8 mg) of 1,3,5-trimethoxybenzene was added. The mixture was completely dissolved in 1 mL of DMSO*d6* and analysed by NMR. The signals were assigned according to figure S4.

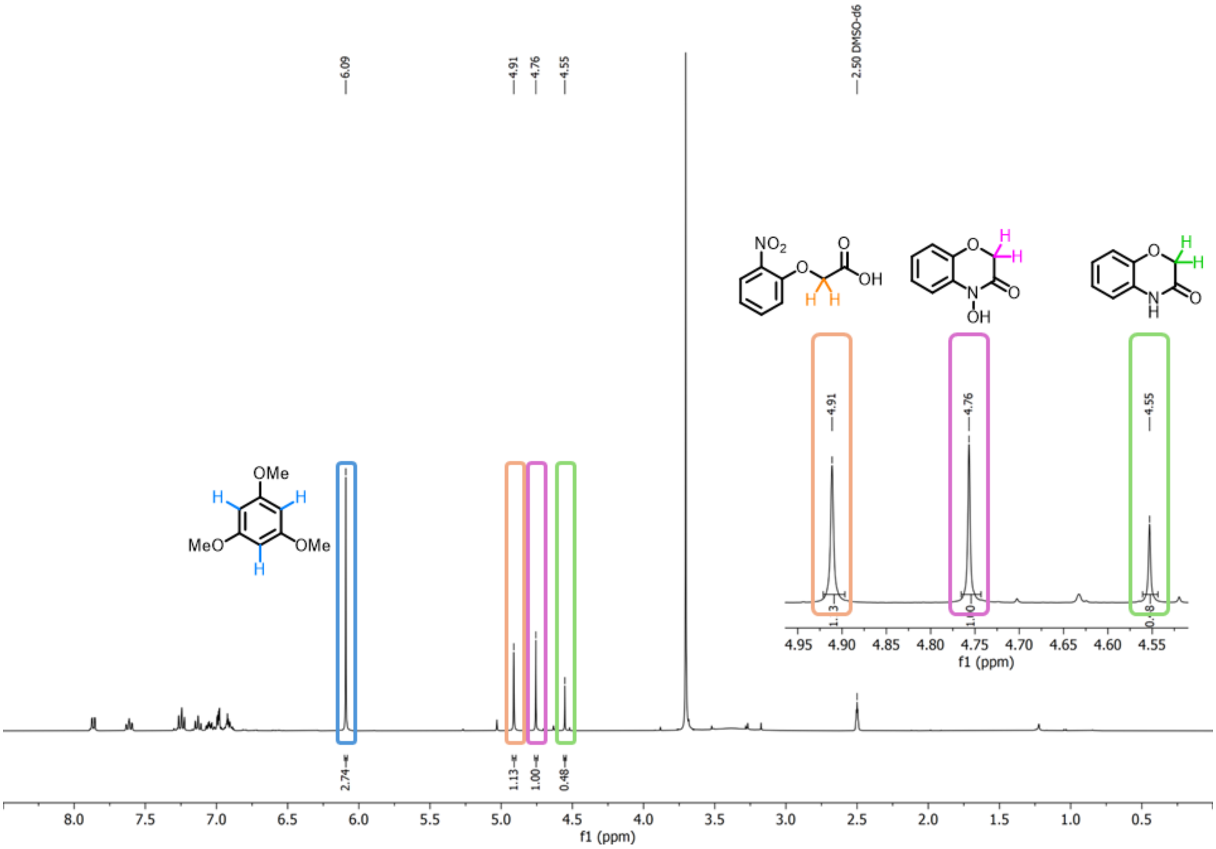

Figure S4: Assignment of the ^{1}H NMR signals for NMR quantification of the optimisation of the electrolytic conditions.

4. Scale-up of the Electrochemical Reductive Synthesis of 2*H*,4*H*-2,2-Dimethyl-4-hydroxybenzo[*b*]-1,4-oxazin-3-one (5c)

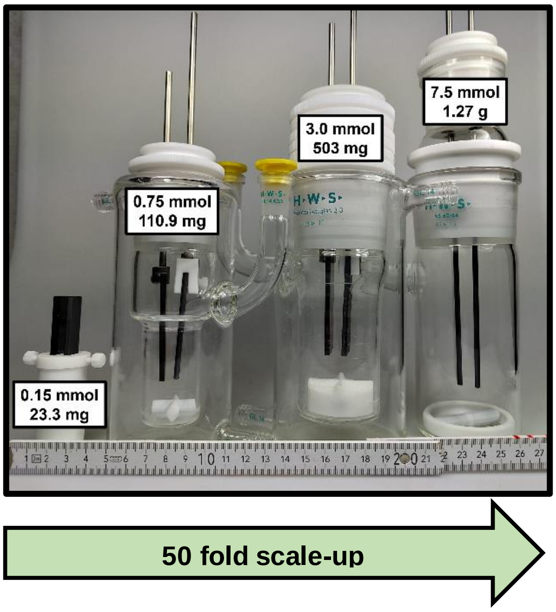

Figure S5: Different batch-type undivided cells used for the scale-up of the reaction. The ruler depicted on the bottom is in cm.

Table S10: Scale-up of the synthesis of **5c**.

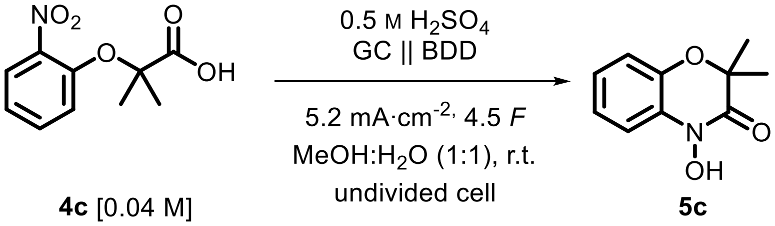

4c [0.04 M] **5c**

Cell Volume [mL]	Scale [mmol]	Purification Method	Applied Charge	Yield[a] [%] of **5c**
5	0.15	Reverse phase column	57.9 C (4.0 *F*)	23.3 mg (0.121 mmol, 81%)
25	0.75	Reverse phase column	325.6 C (4.5 *F*)	110.9 mg (0.574 mmol, 77%)
100	3.0	Reverse phase column	1302.6 C (4.5 *F*)	503 mg (2.6 mmol, 87%)
250	7.5	crystallisation	3256.4 C (4.5 *F*)	1.27 g (7.5 mmol, 88%)

5. CV Studies

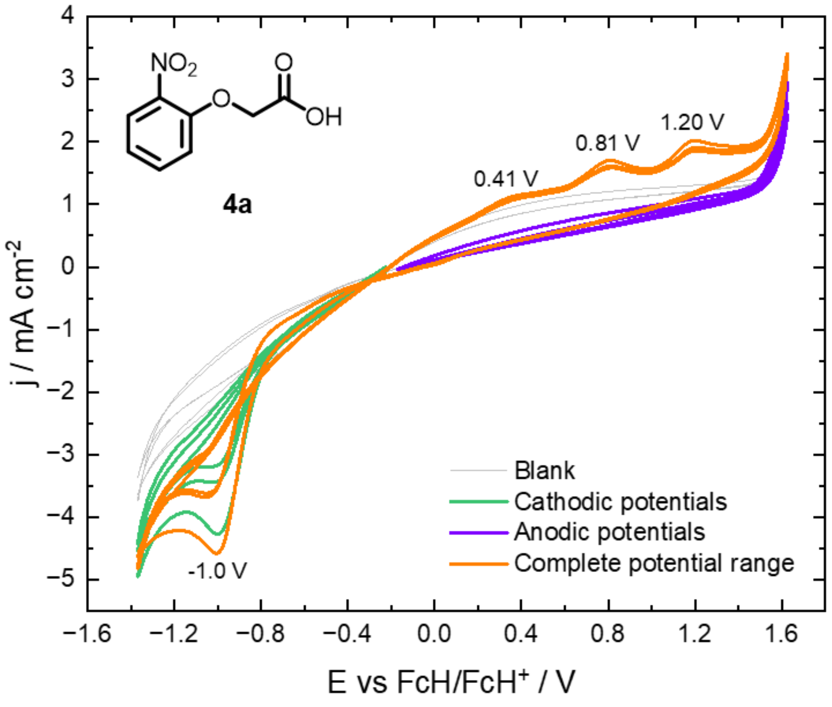

Figure S6: Cyclic voltammogram of **4a** with 0.5 M H_2SO_4 as additive.

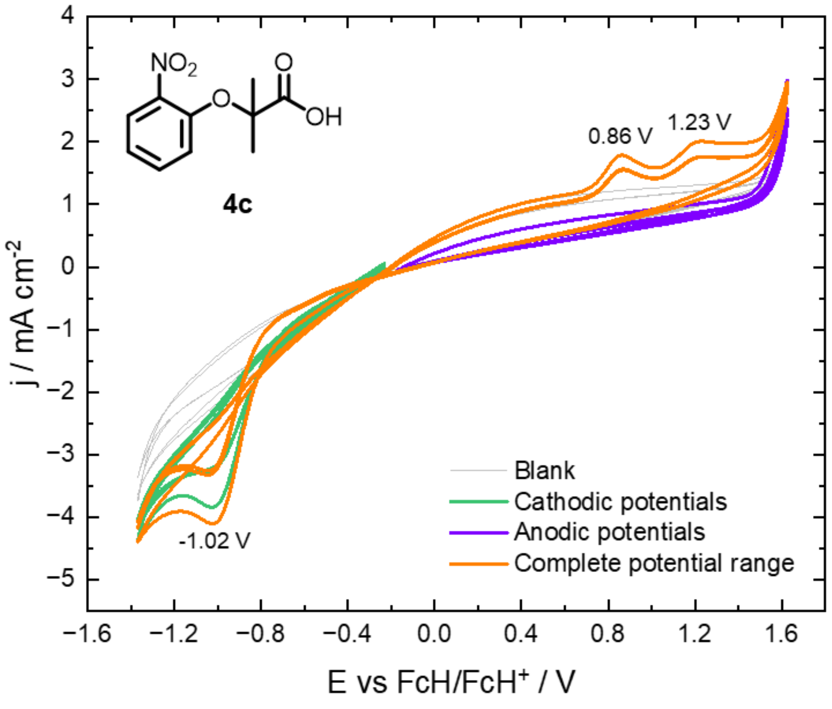

Figure S7: Cyclic voltammogram of **4c** with 0.5 M H_2SO_4 as additive.

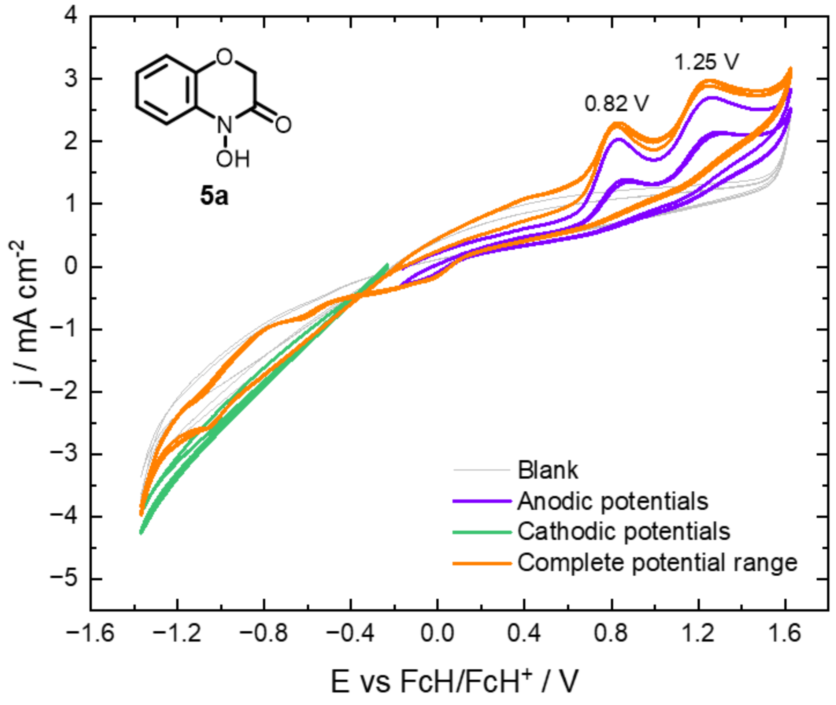

Figure S8: Cyclic voltammogram of **5a** with 0.5 M H_2SO_4 as additive.

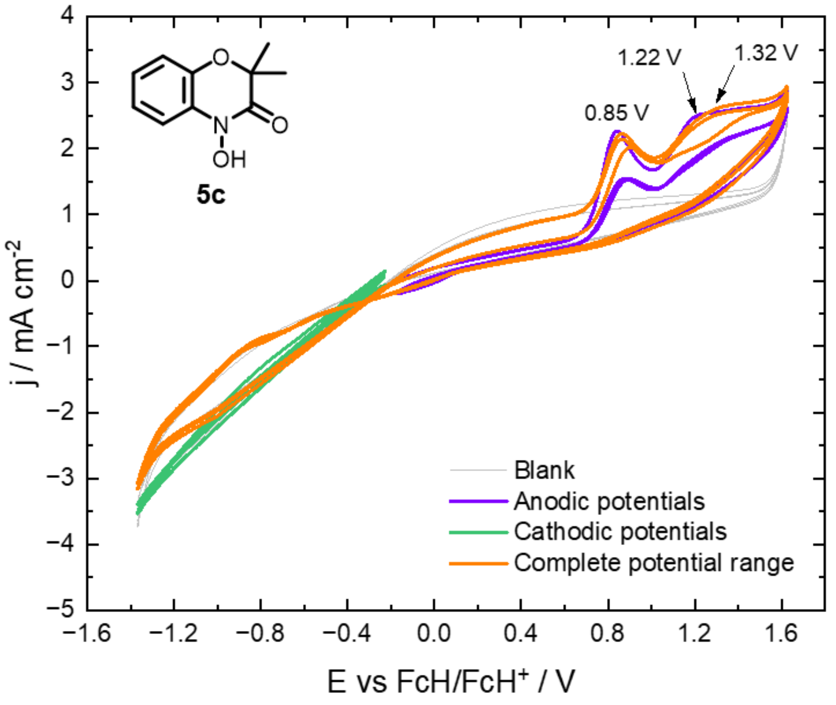

Figure S9: Cyclic voltammogram of **5c** with 0.5 M H_2SO_4 as additive.

6. Preparation of Products and Analytical Data

6.1. 2-Nitrophenoxyaceticacid esters (6a–6t)

Methyl 2-(2-nitrophenoxy)acetate (6a)

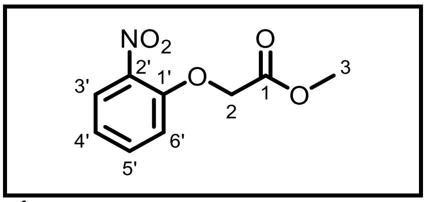

According to general protocol **GPI**, 2-nitrophenol (7.24 g, 52 mmol, 1.0 eq.), potassium carbonate (20.83 g, 151 mmol, 2.9 eq.) and methyl bromoacetate (11.93 g, 78 mmol, 1.5 eq.) were reacted in 150 mL of anhydrous acetone. 9.31 g (44.1 mmol, 85%) of the product was obtained as a colourless solid after crystallisation (80 mL ^{i}PrOH; crystallisation at 6 °C).

^{1}H NMR (400 MHz, CDCl$_3$) δ [ppm]: 7.84 (dd, 1H, *J* = 8.2 Hz, 1.7 Hz, *H*-3’), 7.51 (ddd, *J* = 8.4 Hz, 7.5 Hz, 1.7 Hz, *H*-5’), 7.08 (ddd, 1H, *J* = 8.2 Hz, 7.5 Hz, 1.1 Hz, *H*-4’), 6.98 (dd, 1H, *J* = 8.4 Hz, 1.1 Hz, *H*-6’), 4.77 (s, 2H, *H*-2), 3.78 (s, 3H, *H*-3).
^{13}C NMR (101 MHz, CDCl$_3$) δ [ppm]: 168.3, 151.2, 140.4, 134.2, 125.9, 121.9, 115.2, 66.5, 52.6.
LC-MS: t_R = 11.027 min (method: 10 → 90% acetonitrile in 10 min, 5 min at 100% acetonitrile), m/z for $C_9H_9NO_5^+$ [M+H]+ = 212.
m.p. (^{i}PrOH): 54.1–54.6 °C.
Known compound, spectroscopic data match to literature.[1]

Ethyl 2-(2-nitrophenoxy)propanoate (6b)

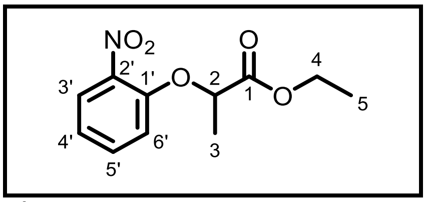

According to general protocol **GPI**, 2-nitrophenol (1.40 g, 10 mmol, 1.0 eq.), potassium carbonate (4.16 g, 30 mmol, 3.0 eq.) and ethyl 2-bromopropanoate (2.76 g, 15 mmol, 1.5 eq.) were reacted in 30 mL of anhydrous acetone. 1.59 g (6.6 mmol, 66%) of the product was obtained as a slightly yellow solid after crystallisation (30 mL cyclohexane; crystallisation at 6 °C).

^{1}H NMR (400 MHz, CDCl$_3$) δ [ppm]: 7.82 (dd, 1H, *J* = 8.1 Hz, 1.7 Hz, *H*-3’), 7.47 (ddd, 1H, *J* = 8.4 Hz, 7.4 Hz, 1.7 Hz, *H*-5’), 7.11 – 7.02 (m, 1H, *H*-4’), 6.95 (dd, 1H, *J* = 8.4 Hz, 1.1 Hz, *H*-6’), 4.83 (q, 1H, *J* = 6.8 Hz, *H*-2), 4.21 (qd, 2H, *J* = 7.1 Hz, 2.9 Hz, *H*-4), 1.68 (d, 3H, *J* = 6.8 Hz, *H*-3), 1.23 (t, 3H, *J* = 7.1 Hz, *H*-5).
^{13}C NMR (101 MHz, CDCl$_3$) δ [ppm]: 171.0, 151.1, 140.9, 133.9, 125.8, 121.6, 115.9, 74.7, 61.8, 18.5, 14.2.
LC-MS: t_R = 6.464 min (method: 50 → 100% acetonitrile in 10 min, 5 min at 100% acetonitrile), m/z for $C_9H_9NO_5^+$ [M+H]+ = 240.
m.p. (Cy): 43.8–47.2 °C.
Known compound, spectroscopic data match to literature.[1]

Methyl 2-methyl-2-(2-nitrophenoxy)propanoate (6c)

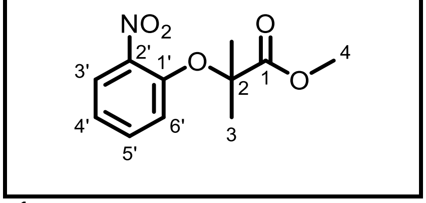

According to general protocol **GPI**, 2-nitrophenol (16.02 g, 115 mmol, 1.0 eq.), potassium carbonate (47.91 g, 347 mmol, 3.0 eq.) and methyl 2-bromo-2-methylpropanoate (31.31 g, 173 mmol, 1.5 eq.) were reacted in 150 mL of anhydrous acetone. 17.50 g (78 mmol, 68%) of the product was obtained and used without further purification as a yellow oil.

^{1}H NMR (400 MHz, CDCl$_3$) δ [ppm]: 7.74 (dd, 1H, *J* = 8.1 Hz, 1.7 Hz, *H*-3’), 7.42 (ddd, 1H, *J* = 8.4 Hz, 7.4 Hz, 1.7 Hz, *H*-5’), 7.07 (ddd, 1H, *J* = 8.1 Hz, 7.4 Hz, 1.2 Hz, *H*-4’), 6.93 (dd, 1H, *J* = 8.4 Hz, 1.2 Hz, *H*-6’), 3.78 (s, 3H, *H*-4), 1.64 (s, 6H, *H*-3).
^{13}C NMR (101 MHz, CDCl$_3$) δ [ppm]: 174.2, 148.8, 143.2, 133.1, 125.3, 122.2, 120.1, 81.6, 52.9, 25.2.
LC-MS: t_R = 3.876 min (method: 60 → 100% acetonitrile in 10 min, 5 min at 100% acetonitrile), m/z for $C_9H_9NO_5^+$ [M+H]+ = 240.

Ethyl 2-(2-nitrophenoxy)butanoate (6d)

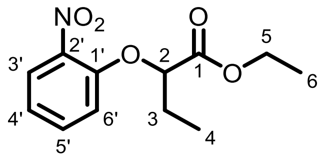

According to general protocol **GPI**, 2-nitrophenol (1.40 g, 10 mmol, 1.0 eq.), potassium carbonate (4.20 g, 30 mmol, 3.0 eq.) and ethyl 2-bromobutyrate (2.95 g, 15 mmol, 1.5 eq.) were reacted in 30 mL of anhydrous acetone. 1.82 g (7.2 mmol, 72%) of the product was obtained as a slightly yellow solid after column chromatography (SiO_2; cyclohexane:ethyl acetate, gradient: 0 → 10% ethyl acetate)

^{1}H NMR (400 MHz, $CDCl_3$) δ [ppm]: 7.82 (dd, 1H, *J* = 8.1 Hz, 1.7 Hz, *H*-3’), 7.46 (ddd, 1H, *J* = 8.4, 7.4 Hz, 1.7 Hz, *H*-5’), 7.04 (ddd, 1H, *J* = 8.1 Hz, 7.4 Hz, 1.1 Hz, *H*-4’), 6.90 (dd, 1H, *J* = 8.4 Hz, 1.1 Hz, *H*-6’), 4.67 (t, 1H, *J* = 6.0 Hz, *H*-2), 4.20 (qd, 2H, *J* = 7.1 Hz, 2.8 Hz, *H*-5), 2.05 (qd, 2H, *J* = 7.4 Hz, 6.0 Hz, *H*-3), 1.22 (t, 3H, *J* = 7.1 Hz, *H*-6), 1.10 (t, 3H, *J* = 7.4 Hz, *H*-4).
^{13}C NMR (101 MHz, $CDCl_3$) δ [ppm]: 170.5, 151.4, 140.5, 133.9, 125.8, 121.3, 115.2, 79.1, 61.6, 26.1, 14.2, 9.5.
HR-MS (ESI+): *m/z* for $C_{12}H_{15}NO_5$+H^+, $[M+H]^+$ calculated: 254.1023; found: 254.1012.

Ethyl 1-(2-nitrophenoxy)cyclobutane-1-carboxylate (6e)

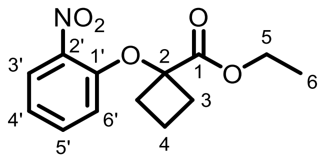

According to general protocol **GPI**, 2-nitrophenol (2.08 g, 15 mmol, 1.0 eq.), potassium carbonate (4.22 g, 31 mmol, 2.0 eq.) and ethyl 1-bromocyclobutane-1-carboxylate (4.76 g, 23 mmol, 1.5 eq.) were reacted in 70 mL of anhydrous DMF. 0.74 g (2.8 mmol, 19%) of the product was obtained as a slightly yellow oil after column chromatography (SiO_2; cyclohexane:ethyl acetate, gradient: 0 → 5% ethyl acetate).

^{1}H NMR (300 MHz, $CDCl_3$) δ [ppm]: 7.80 (dd, 1H, *J* = 8.1 Hz, 1.7 Hz, *H*-3’), 7.38 (ddd, *J* = 8.5, 7.4, 1.7 Hz, 1H), 7.00 (ddd, *J* = 8.4, 7.4, 1.1 Hz, 1H), 6.55 (dd, *J* = 8.5, 1.1 Hz, 1H), 4.18 (q, *J* = 7.1 Hz, 2H), 2.74 (dddd, *J* = 11.3, 5.6, 4.3, 2.4 Hz, 2H), 2.58 – 2.44 (m, 2H), 2.08 – 1.95 (m, 2H), 1.14 (t, *J* = 7.1 Hz, 3H).
^{13}C NMR (75 MHz, $CDCl_3$) δ [ppm]: 172.0, 149.3, 140.5, 133.5, 125.9, 120.8, 115.9, 81.0, 61.8, 32.4, 13.8.
HR-MS (ESI+): *m/z* for $C_{13}H_{15}NO_5$+H^+, $[M+H]^+$ calculated: 266.1023; found: 266.1028.

Ethyl 2-(2-nitrophenoxy)octanoate (6f)

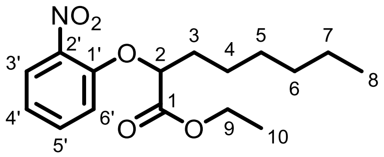

According to general protocol **GPI**, 2-nitrophenol (1.40 g, 10 mmol, 1.0 eq.), potassium carbonate (4.18 g, 30 mmol, 3.0 eq.) and ethyl 2-bromooctanoate (3.80 g, 15 mmol, 1.5 eq.) were reacted in 30 mL of anhydrous acetone. 2.36 g (7.6 mmol, 76%) of the product was obtained as a slightly yellow solid after crystallisation (35 mL cyclohexane; crystallisation at 6 °C).

^{1}H NMR (400 MHz, $CDCl_3$) δ [ppm]: 7.83 (dd, 1H, *J* = 8.1 Hz, 1.7 Hz, *H*-3’), 7.46 (ddd, 1H, *J* = 8.4, 7.5 Hz, 1.7 Hz, *H*-5’), 7.07 – 7.02 (m, 1H, *H*-4’), 6.90 (dd, 1H, *J* = 8.4 Hz, 1.1 Hz, *H*-6’), 4.71 (dd, 1H, *J* = 7.4 Hz, 4.9 Hz, *H*-2), 4.20 (qd, 2H, *J* = 7.1 Hz, 2.3 Hz, *H*-9), 2.09 – 1.92 (m, 2H, *H*-3), 1.63 – 1.46 (m, 2H, *H*-4), 1.40 – 1.27 (m, 6H, *H*-5, *H*-6, *H*-7), 1.22 (t, 3H, *J* = 7.1 Hz, *H*-10), 0.92 – 0.85 (m, 3H, *H*-8).
^{13}C NMR (101 MHz, $CDCl_3$) δ [ppm]: 170.7, 151.4, 140.6, 133.9, 125.9, 121.3, 115.2, 78.2, 61.7, 32.7, 31.7, 28.9, 25.0, 22.7, 14.2, 14.2.
HR-MS (ESI+): *m/z* for $C_{16}H_{23}NO_5$+H^+, $[M+H]^+$ calculated: 310.1649; found: 310.1641.
m.p. (Cy): 48.0–49.8 °C.

Methyl 2-(2-nitrophenoxy)-2-phenylacetate (6h)

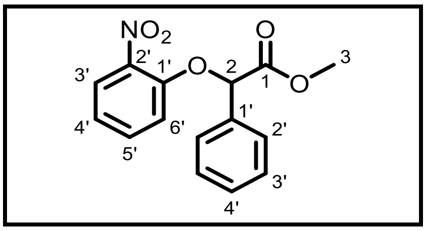

According to general protocol **GPI**, 2-nitrophenol (1.40 g, 10 mmol, 1.0 eq.), potassium carbonate (4.19 g, 30 mmol, 3.0 eq.) and methyl 2-bromo-2-phenylacetate (3.61 g, 16 mmol, 1.6 eq.) were reacted in 30 mL of anhydrous acetone. 1.01 g (3.5 mmol, 35%) of the product was obtained as a slightly yellow solid after column chromatography (SiO_2; cyclohexane:ethyl acetate, gradient: 3 → 15% ethyl acetate)

^{1}H NMR (400 MHz, $CDCl_3$) δ [ppm]: 7.90 (dd, 1H, *J* = 8.1 Hz, 1.7 Hz, *H*-3'), 7.62 (dd, 2H, *J* = 7.7 Hz, 1.8 Hz, *H*-2''), 7.49 (ddd, 1H, *J* = 8.4 Hz, 7.5 Hz, 1.7 Hz, *H*-5'), 7.47 – 7.34 (m, 3H, *H*-3'', *H*-4''), 7.10 (ddd, 1H, *J* = 8.4 Hz, 7.5 Hz, 1.1 Hz, *H*-4'), 6.99 (dd, 1H, *J* = 8.4 Hz, 1.2 Hz, *H*-6'), 5.76 (s, 1H, *H*-2), 3.73 (s, 3H, *H*-3).
^{13}C NMR (101 MHz, $CDCl_3$) δ [ppm]: 169.4, 150.7, 140.8, 134.2, 129.5, 129.1, 128.2, 127.1, 126.2, 121.9, 115.9, 79.9, 53.1.
HR-MS (ESI+): *m/z* for $C_{15}H_{13}NO_5+NH_4^+$, $[M+ NH_4]^+$ calculated: 305.1132; found: 305.1133.
Known compound, spectroscopic data match to literature.[2]

Methyl 2-(2-chlorophenyl)-2-(2-nitrophenoxy)acetate (6i)

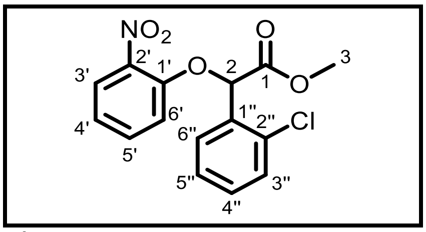

According to general protocol **GPI**, 2-nitrophenol (0.87 g, 6 mmol, 1.0 eq.), potassium carbonate (2.55 g, 18 mmol, 3.0 eq.) and 2-bromo-2-(2-chlorophenyl)acetate (2.42 g, 9 mmol, 1.5 eq.) were reacted in 30 mL of anhydrous acetone. 1.52 g (4.7 mmol, 77%) of the product was obtained as a beige solid after column chromatography (SiO_2; cyclohexane:ethyl acetate, gradient: 3 → 15% ethyl acetate)

^{1}H NMR (400 MHz, $CDCl_3$) δ [ppm]: 7.87 (dd, 1H, *J* = 8.1 Hz, 1.7 Hz, *H*-3'), 7.78 – 7.73 (m, 1H, *H*-3''), 7.49 (ddd, 1H, *J* = 8.4 Hz, 7.4, 1.7 Hz, *H*-5'), 7.44 – 7.40 (m, 1H, *H*-5''), 7.37 – 7.29 (m, 2H, *H*-4'', *H*-6''), 7.09 (ddd, 1H, *J* = 8.1 Hz, 7.4 Hz, 1.1 Hz, *H*-4'), 7.05 (dd, 1H, *J* = 8.4 Hz, 1.1 Hz, *H*-6'), 6.28 (s, 1H, *H*-2), 3.76 (s, 3H, *H*-3).
^{13}C NMR (101 MHz, $CDCl_3$) δ [ppm]: 168.5, 150.4, 140.8, 134.2, 133.3, 132.5, 130.7, 129.8, 129.2, 127.9, 126.1, 122.1, 116.0, 76.1, 53.1.
HR-MS (ESI+): *m/z* for $C_{15}H_{12}{}^{35}ClNO_5+Na^+$, $[M+Na]^+$ calculated: 322.0477; found: 322.0466.

Methyl 2-(4-methoxy-2-nitrophenoxy)acetate (6j)

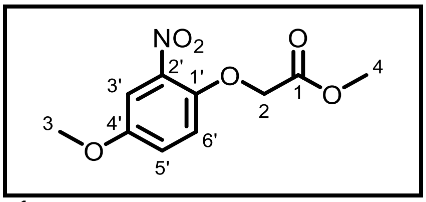

According to general protocol **GPI**, 4-methoxy-2-nitrophenol (1.73 g, 10 mmol, 1.0 eq.), potassium carbonate (4.29 g, 31 mmol, 3.1 eq.) and methyl 2-bromoacetate (2.32 g, 15 mmol, 1.5 eq.) were reacted in 30 mL of anhydrous acetone. 2.17 g (9.0 mmol, 90%) of the product was obtained as a colourless solid and was used without further purification.

^{1}H NMR (400 MHz, $CDCl_3$) δ [ppm]: 7.38 (d, 1H, *J* = 3.0 Hz, *H*-3'), 7.07 (dd, 1H, *J* = 9.1 Hz, 3.0 Hz, *H*-5'), 7.01 (d, 1H, *J* = 9.1 Hz, *H*-6'), 4.71 (s, 2H, *H*-2), 3.81 (s, 3H, *H*-3), 3.78 (s, 3H, *H*-4).
^{13}C NMR (101 MHz, $CDCl_3$) δ [ppm]: 168.8, 154.4, 145.6, 141.1, 120.8, 118.4, 110.1, 68.1, 56.2, 52.5.
HR-MS (ESI-): *m/z* for $C_9H_9NO_6-H^-$, $[M-H]^-$ calculated: 226.0357; found: 226.0361.

Methyl 2-(4-*tert*-butyl-2-nitrophenoxy)acetate (6k)

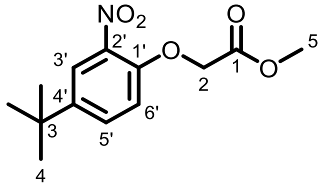

According to general protocol **GPI**, 4-*tert*-butyl-2-nitrophenol (1.97 g, 10 mmol, 1.0 eq.), potassium carbonate (4.22 g, 31 mmol, 3.1 eq.) and methyl 2-bromoacetate (2.35 g, 15 mmol, 1.5 eq.) were reacted in 30 mL of anhydrous acetone. 2.61 g (9.8 mmol, 98%) of the product was obtained as a beige solid and was used without further purification.

^{1}H NMR (400 MHz, CDCl$_3$) δ [ppm]: 7.86 (d, 1H, *J* = 2.5 Hz, *H*-3'), 7.52 (dd, 1H, *J* = 8.8 Hz, 2.5 Hz, *H*-5'), 6.92 (d, 1H, *J* = 8.8 Hz, *H*-6'), 4.76 (s, 2H, *H*-2), 3.80 (s, 3H, *H*-5), 1.31 (s, 9H, *H*-4).
^{13}C NMR (101 MHz, CDCl$_3$) δ [ppm]: 1 168.6, 149.1, 145.6, 140.1, 131.3, 122.9, 115.2, 66.8, 52.6, 34.6, 31.2.
HR-MS (ESI+): *m/z* for $C_{13}H_{17}NO_5$+Na$^+$, [M+Na]$^+$ calculated: 290.0999; found: 290.0988.

Ethyl 2-((3-nitro-biphenyl-4-yl)oxy)acetate (6l)

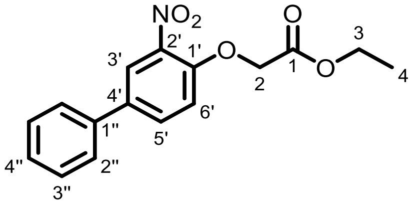

According to general protocol **GPI**, 3-nitro-biphenyl-4-ol (4.30 g, 20 mmol, 1.0 eq.), potassium carbonate (5.57 g, 40 mmol, 3.0 eq.) and ethyl 2-bromoacetate (5.01 g, 30 mmol, 1.5 eq.) were reacted in 150 mL of anhydrous acetone. 5.18 g (17.2 mmol, 86%) of the product was obtained as a slightly yellow solid after washing the solids with methanol.

^{1}H NMR (300 MHz, CDCl$_3$) δ [ppm]: 8.08 (d, 1H, *J* = 2.4 Hz, *H*-3'), 7.72 (dd, 1H, *J* = 8.7 Hz, 2.4 Hz, *H*-5'), 7.55 – 7.51 (m, 2H, *H*-2''), 7.48 – 7.33 (m, 3H, *H*-3'', *H*-4''), 7.06 (d, 1H, *J* = 8.7 Hz, *H*-6'), 4.81 (s, 2H, *H*-2), 4.28 (q, 1H, *J* = 7.1 Hz, *H*-3), 1.30 (t, *J* = 7.1 Hz, 3H, *H*-4).
^{13}C NMR (75 MHz, CDCl$_3$) δ [ppm]: 167.8, 150.6, 140.6, 138.2, 135.3, 132.4, 129.2, 128.1, 126.8, 124.2, 115.7, 66.7, 61.9, 14.2.
HR-MS (ESI+): *m/z* for $C_{16}H_{15}NO_5$+Na$^+$, [M+Na]$^+$ calculated: 324.0842; found: 324.0835.

Methyl 2-(4-fluoro-2-nitrophenoxy)acetate (6m)

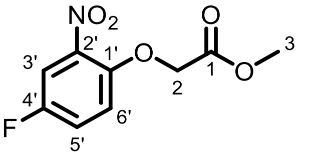

According to general protocol **GPI**, 4-fluoro-2-nitrophenol (1.58 g, 10 mmol, 1.0 eq.), potassium carbonate (4.17 g, 30 mmol, 3.0 eq.) and methyl 2-bromoacetate (2.34 g, 15 mmol, 1.5 eq.) were reacted in 30 mL of anhydrous acetone. 2.06 g (9.0 mmol, 90%) of the product was obtained as a slightly yellow solid after washing the solids with cyclohexane:ethanol (1:1).

^{1}H NMR (400 MHz, CDCl$_3$) δ [ppm]: 7.63 (dd, 1H, *J* = 7.7 Hz, 3.1 Hz, *H*-3'), 7.31 – 7.22 (m, 1H, *H*-5'), 7.03 (dd, 1H, *J* = 9.2 Hz, 4.3 Hz, *H*-6'), 4.76 (s, 2H, *H*-2), 3.80 (s, 3H, *H*-3).
^{13}C NMR (101 MHz, CDCl$_3$) δ [ppm]: 168.3, 156.4 (d, *J* = 246.1 Hz), 148.0 (d, *J* = 3.1 Hz), 140.7, 121.2 (d, *J* = 23.0 Hz), 117.7 (d, *J* = 8.0 Hz), 113.3 (d, *J* = 27.4 Hz), 67.5, 52.7.
^{19}F NMR (376 MHz, CDCl$_3$) δ [ppm]: -119.36 – -119.42 (m).
HR-MS (ESI+): *m/z* for $C_9H_8FNO_5$+Na$^+$, [M+Na]$^+$ calculated: 252.0279; found: 252.0269.

Methyl 2-(4-chloro-2-nitrophenoxy)acetate (6n)

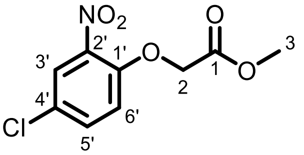

According to general protocol **GPI**, 4-chloro-2-nitrophenol (4.37 g, 25 mmol, 1.0 eq.), potassium carbonate (10.38 g, 30 mmol, 3.0 eq.) and methyl 2-bromoacetate (5.75 g, 15 mmol, 1.5 eq.) were reacted in 30 mL of anhydrous acetone. 4.14 g (16.8 mmol, 67%) of the product was obtained as a slightly yellow solid after crystallisation (42 mL cyclohexane; crystallisation at 6 °C).

^{1}H NMR (400 MHz, CDCl$_3$) δ [ppm]: 7.86 (d, 1H, *J* = 2.6 Hz, *H*-3'), 7.48 (dd, 1H, *J* = 8.9 Hz, 2.6 Hz, *H*-5'), 6.95 (d, 1H, *J* = 8.9 Hz, *H*-6'), 4.77 (s, 2H, *H*-2), 3.80 (s, 3H, *H*-3).
^{13}C NMR (101 MHz, CDCl$_3$) δ [ppm]: 168.0, 150.1, 140.7, 134.0, 127.1, 125.9, 116.8, 66.8, 52.7.
HR-MS (ESI+): *m/z* for $C_9H_8{}^{35}ClNO_5$+Na$^+$, [M+Na]$^+$ calculated: 267.9983; found: 267.9977.

Methyl 2-(4-bromo-2-nitrophenoxy)acetate (6o)

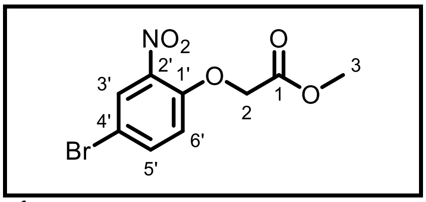

According to general protocol **GPI**, 4-bromo-2-nitrophenol (2.18 g, 10 mmol, 1.0 eq.), potassium carbonate (4.21 g, 30 mmol, 3.0 eq.) and methyl 2-bromoacetate (2.33 g, 15 mmol, 1.5 eq.) were reacted in 30 mL of anhydrous acetone. 2.65 g (9.1 mmol, 91%) of the product was obtained as a yellow solid after washing the solids with cyclohexane:ethanol (1:1).

^{1}H NMR (400 MHz, CDCl$_3$) δ [ppm]: 7.98 (d, 1H, *J* = 2.5 Hz, *H*-3’), 7.60 (dd, 1H, *J* = 8.9 Hz, 2.5 Hz, *H*-5’), 6.89 (d, 1H, *J* = 8.9 Hz, *H*-6’), 4.77 (s, 2H, *H*-2), 3.79 (s, 3H, *H*-3).
^{13}C NMR (101 MHz, CDCl$_3$) δ [ppm]: 168.0, 150.5, 140.8, 136.9, 128.6, 117.0, 113.6, 66.6, 52.7.
HR-MS (ESI+): *m/z* for $C_9H_8{}^{79}BrNO_5+Na^+$, $[M+Na]^+$ calculated: 311.9478; found: 311.9475.
Known compound, spectroscopic data match to literature.[3]

Methyl 2-(2-nitro-4-trifluoromethylphenoxy)acetate (6p)

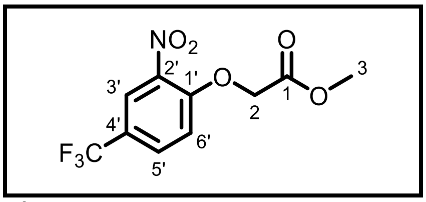

According to general protocol **GPI**, 2-nitro-4-trifluoromethylphenol (2.09 g, 10 mmol, 1.0 eq.), potassium carbonate (4.16 g, 30 mmol, 3.0 eq.) and methyl 2-bromoacetate (2.31 g, 15 mmol, 1.5 eq.) were reacted in 30 mL of anhydrous acetone. 1.67 g (6.0 mmol, 60%) of the product was obtained as a beige solid after washing the solids with cyclohexane:ethanol (1:1).

^{1}H NMR (400 MHz, CDCl$_3$) δ [ppm]: 8.16 (d, *J* = 2.3 Hz, 1H), 7.81 – 7.74 (m, 1H), 7.07 (d, *J* = 8.8 Hz, 1H), 4.86 (s, 2H), 3.82 (s, 3H).
^{13}C NMR (101 MHz, CDCl$_3$) δ [ppm]: 167.6, 153.6, 140.0, 130.9 (d, *J* = 3.7 Hz), 127.8 – 124.0 (m), 123.8 (q, *J* = 3.5 Hz), 115.2, 66.3, 52.9.
^{19}F NMR (376 MHz, CDCl$_3$) δ [ppm]: -63.34.
HR-MS (ESI+): *m/z* for $C_{10}H_8F_3NO_5+Na^+$, $[M+Na]^+$ calculated: 302.0247; found: 302.0237.

Ethyl 2-(2-nitrophenoxy)acetate (6s)

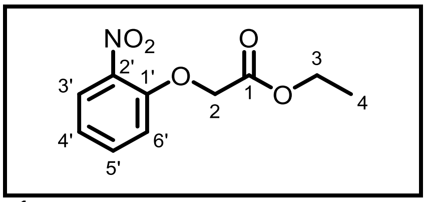

According to general protocol **GPI**, 2-nitrophenol (13.91 g, 100 mmol, 1.0 eq.), potassium carbonate (42.23 g, 300 mmol, 3.0 eq.) and ethyl 2-bromoacetate (23.1 mL, 20 mmol, 2.0 eq.) were reacted in 300 mL of anhydrous acetone. 15.90 g (70.6 mmol, 71%) of the product was obtained as a colourless solid after crystallisation (100 mL ethanol; crystallisation at 6 °C).

^{1}H NMR (400 MHz, CDCl$_3$) δ [ppm]: 7.81 (dd, 1H, *J* = 8.1 Hz, 1.6 Hz, *H*-3’), 7.52 – 7.45 (m, 1H, *H*-5’), 7.06 (ddd, 1H, *J* = 8.3 Hz, 7.4 Hz, 1.1 Hz, *H*-4’), 6.97 (dd, 1H, *J* = 8.3 Hz, 1.1 Hz, *H*-6’), 4.74 (s, 2H, *H*-2), 4.22 (q, 2H, *J* = 7.1 Hz, *H*-3), 1.24 (t, 3H, *J* = 7. Hz, *H*-4).
^{13}C NMR (101 MHz, CDCl$_3$) δ [ppm]: 167.7, 151.2, 140.3, 134.1, 125.8, 121.7, 115.2, 66.5, 61.7, 14.1.
HR-MS (ESI+): *m/z* for $C_{10}H_{11}NO_5+Na^+$, $[M+Na]^+$ calculated: 248.0529; found: 248.0522.
m.p. (EtOH): 44.0–45.1 °C.
Known compound, spectroscopic data match to literature.[4]

***tert*-Butyl 2-(2-nitrophenoxy)acetate (6t)**

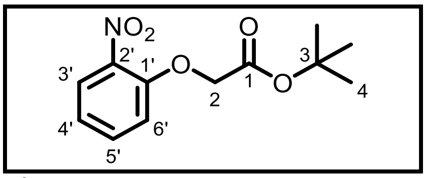

According to general protocol **GPI**, 2-nitrophenol (1.39 g, 10 mmol, 1.0 eq.), potassium carbonate (2.81 g, 20 mmol, 2.0 eq.) and *tert*-butyl 2-bromoacetate (2.98 g, 15 mmol, 1.5 eq.) were reacted in 30 mL of anhydrous acetone. 1.59 g (6.6 mmol, 66%) of the product was obtained as a yellow oil.

^{1}H NMR (400 MHz, CDCl$_3$) δ [ppm]: 7.87 – 7.81 (m, 1H, *H*-3’), 7.54 – 7.46 (m, 1H, *H*-5’), 7.10 – 7.04 (m, 1H, *H*-4’), 6.98 – 6.93 (m, 1H, *H*-6’), 4.66 (s, 2H, *H*-2), 1.45 (s, 9H, *H*-4).
^{13}C NMR (101 MHz, CDCl$_3$) δ [ppm]: 166.8, 151.5, 140.3, 134.0, 125.9, 121.5, 114.9, 83.1, 66.8, 28.1.
HR-MS (ESI+): *m/z* for $C_{12}H_{15}NO_5+Na^+$, $[M+Na]^+$ calculated: 276.0842; found: 276.0829.
Known compound, spectroscopic data match to literature.[1]

6.2. 2-Nitrophenoxyacetic acid (4a-4r)

2-(2-Nitrophenoxy)acetic acid (4a)

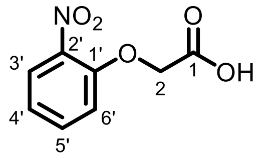

According to general protocol **GPII**, methyl 2-(2-nitrophenoxy)acetate (**6a**, 2.12 g, 10 mmol, 1.0 eq.) and lithium hydroxide monohydrate (1.01 g, 20 mmol, 2.0 eq.) were reacted in 40 mL THF and 20 mL of water. 1.90 g (9.7 mmol, 97%) of the product was obtained as a colourless solid without further purification.

^{1}H NMR (400 MHz, DMSO$_{d6}$) δ [ppm]: 13.25 (s, 1H, 1-COO*H*), 7.86 (dd,1H, *J* = 8.0 Hz, 1.7 Hz, *H*-3'), 7.61 (ddd, 1H, *J* = 8.6 Hz, 7.4 Hz, 1.7 Hz, *H*-5'), 7.25 (dd, 1H, *J* = 8.6 Hz, 1.1 Hz, , *H*-6'), 7.13 (ddd, 1H, *J* = 8.0 Hz, 7.4 Hz, 1.1 Hz, *H*-4'), 4.91 (s, 2H, , *H*-2).
^{13}C NMR (101 MHz, DMSO$_{d6}$) δ [ppm]: 169.8, 150.9, 140.1, 134.5, 125.4, 121.5, 115.5, 65.7.
HR-MS (ESI-): *m/z* for $C_8H_7NO_5$-H^-, $[M-H]^-$ calculated: 196.0251; found: 196.0254.
Known compound, spectroscopic data match to literature.[1]

2-(2-Nitrophenoxy)propanoic acid (4b)

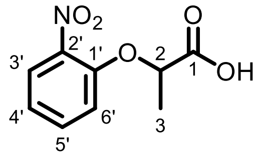

According to general protocol **GPII**, ethyl 2-(2-nitrophenoxy)propionate (**6b**, 1.00 g, 4 mmol, 1.0 eq.) and lithium .hydroxide monohydrate (0.37 g, 9 mmol, 2.3 eq.) were reacted in 40 mL THF and 20 mL of water. 0.41 g (1.9 mmol, 45%) of the product was obtained as a beige solid without further purification.

^{1}H NMR (400 MHz, DMSO$_{d6}$) δ [ppm]: 13.25 (s, 1H, 1-COO*H*), 7.85 (dd, 1H, *J* = 8.0 Hz, 1.7 Hz, *H*-3'), 7.61 (ddd, 1H, *J* = 8.5 Hz, 7.4 Hz, 1.7 Hz, *H*-5'), 7.19 (dd, 1H, *J* = 8.5 Hz, 1.1 Hz, *H*-6'), 7.12 (ddd, 1H, *J*= 8.0 Hz, 7.4 Hz, 1.1 Hz, *H*-6'), 5.11 (q, 1H, *J* = 6.8 Hz, *H*-2), 1.52 (d, 3H, *J* = 6.8 Hz, *H*-3).
^{13}C NMR (101 MHz, DMSO$_{d6}$) δ [ppm]: 172.1, 150.1, 140.0, 134.1, 125.0, 121.1, 115.5, 72.9, 18.0.
HR-MS (ESI-): *m/z* for $C_9H_9NO_5$-H^-, $[M-H]^-$ calculated: 210.0408; found: 210.0412.

2-Methyl-2-(2-nitrophenoxy)propanoic acid (4c)

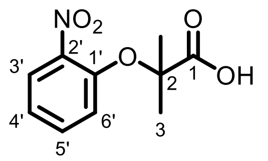

According to general protocol **GPII**, methyl 2-methyl-2-(2-nitrophenoxy)propanoate (**6c**, 20.34 g, 85 mmol, 1.0 eq.) and lithium hydroxide monohydrate (5.38 g, 128 mmol, 1.5 eq.) were reacted in 100 mL THF and 60 mL of water. 17.41 g (77.3 mmol, 91%) of the product was obtained as a colourless solid without further purification.

^{1}H NMR (400 MHz, DMSO$_{d6}$) δ [ppm]: 13.37 (s, 1H, 1-COO*H*), 7.83 (dd, 1H, *J* = 8.1 Hz, 1.7 Hz, *H*-3'), 7.58 (ddd, 1H, *J* = 8.5 Hz, 7.4 Hz, 1.7 Hz, *H*-5'), 7.16 (ddd, 1H, *J* = 8.3 Hz, 7.5 Hz, 1.1 Hz, *H*-4'), 7.05 (dd, 1H, *J* = 8.5 Hz, 1.1 Hz, *H*-6'), 1.55 (s, 6H, *H*-3).
^{13}C NMR (101 MHz, DMSO$_{d6}$) δ [ppm]: 174.1, 147.8, 142.2, 133.3, 124.8, 121.8, 119.0, 80.6, 24.9.
HR-MS (ESI-): *m/z* for $C_{10}H_{11}NO_5$-H^-, $[M-H]^-$ calculated: 224.0564; found: 224.0570.

2-(2-Nitrophenoxy)butyric acid (4d)

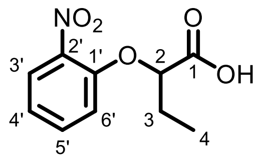

According to general protocol **GPII**, ethyl 2-(2-nitrophenoxy)butanoate (**6d**, 0.92 g, 4 mmol, 1.0 eq.) and lithium hydroxide monohydrate (0.32 g, 8 mmol, 2.0 eq.) were reacted in 40 mL THF and 20 mL of water. 0.63 g (2.8 mmol, 70%) of the product was obtained as a colourless solid without further purification.

^{1}H NMR (400 MHz, DMSO$_{d6}$) δ [ppm]: 13.24 (s, 1H, 1-COO*H*), 7.86 (dd, 1H, *J* = 8.1 Hz, 1.7 Hz, *H*-3'), 7.60 (ddd, 1H, *J* = 8.6 Hz, 7.4 Hz, 1.7 Hz, *H*-5'), 7.16 (dd, 1H, *J* = 8.6 Hz, 1.1 Hz, *H*-6'), 7.11 (ddd, 1H, *J* = 8.1 Hz, 7.4 Hz, 1.1 Hz, *H*-4'), 4.97 (dd, 1H, *J* = 6.9 Hz, 4.6 Hz, *H*-2), 2.03 – 1.83 (m, 2H, *H*-3), 1.00 (t, 3H, *J* = 7.4 Hz, *H*-4).
^{13}C NMR (101 MHz, DMSO$_{d6}$) δ [ppm]: 171.4, 150.5, 139.9, 134.2, 125.1, 121.0, 115.3, 77.3, 25.3, 9.2.
HR-MS (ESI-): *m/z* for $C_{10}H_{11}NO_5$-H^-, $[M-H]^-$ calculated: 224.0564; found: 224.0569.

1-(2-Nitrophenoxy)cyclobutane-1-carboxylic acid (4e)

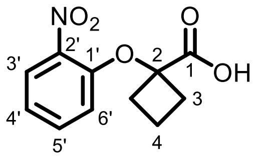

According to general protocol **GPII**, ethyl 1-(2-nitrophenoxy)cyclobutane-1-carboxylate (**6e**, 0.74 g, 3 mmol, 1.0 eq.) and lithium hydroxide monohydrate (0.18 g, 4 mmol, 1.3 eq.) were reacted in 40 mL THF and 20 mL of water. 0.442 g (1.9 mmol, 63%) of the product was obtained as a colourless solid without further purification.

^{1}H NMR (400 MHz, CD_2Cl_2) *δ* [ppm]: 7.82 (dd, 1H, *J* = 8.1 Hz, 1.7 Hz, *H*-3'), 7.46 (ddd, 1H, *J* = 8.4 Hz, 7.5 Hz, 1.7 Hz, *H*-5'), 7.07 (ddd, 1H, *J* = 8.1 Hz, 7.4 Hz, 1.1 Hz, *H*-4'), 6.65 (dd, 1H, *J* = 8.4 Hz, 1.1 Hz, *H*-6'), 2.87 – 2.74 (m, 2H, *H*-3), 2.63 – 2.41 (m, 2H, *H*-3), 2.18 – 1.97 (m, 2H, *H*-4).

^{13}C NMR (101 MHz, CD_2Cl_2) *δ* [ppm]: 176.9, 148.9, 140.9, 134.0, 126.2, 121.6, 116.4, 80.8, 32.7, 14.0.

HR-MS (ESI-): *m/z* for $C_{11}H_{11}NO_5$-H^-, $[M-H]^-$ calculated: 236.0564; found: 236.0569.

2-(2-Nitrophenoxy)caprylic acid (4f)

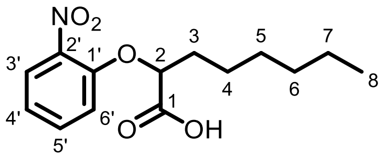

According to general protocol **GPII**, ethyl 2-(2-nitrophenoxy)octanoate (**6f**, 1.51 g, 5 mmol, 1.0 eq.) and lithium hydroxide monohydrate (0.45 g, 11 mmol, 2.2 eq.) were reacted in 40 mL THF and 20 mL of water. 1.350 g (4.8 mmol, 96%) of the product was obtained as a colourless solid without further purification.

^{1}H NMR (400 MHz, DMSO$_{d6}$) *δ* [ppm]: 13.25 (s, 1H, 1-COO*H*), 7.86 (dd, 1H, *J* = 8.1 Hz, 1.7 Hz, *H*-3'), 7.60 (ddd, 1H, *J* = 8.5 Hz, 7.5 Hz, 1.7 Hz, *H*-3'), 7.19 – 7.07 (m, 2H), 4.99 (dd, *J* = 7.1, 4.7 Hz, 1H), 1.95 – 1.80 (m, 2H), 1.53 – 1.36 (m, 1H), 1.36 – 1.21 (m, 7H), 0.88 – 0.83 (m, 3H).

^{13}C NMR (101 MHz, DMSO$_{d6}$) *δ* [ppm]: 167.8, 150.6, 140.6, 138.2, 135.3, 132.4, 129.2, 128.1, 126.8, 124.2, 115.7, 66.7, 61.9, 14.2.

HR-MS (ESI-): *m/z* for $C_{14}H_{19}NO_5$-H^-, $[M-H]^-$ calculated: 280.1190; found: 280.1197.

1-(2-Nitrophenoxy)cyclobutane-1-carboxylic acid (4g)

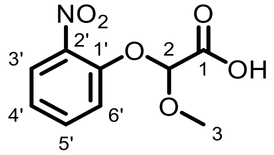

According to general protocol **GPI**, 2-nitrophenol (0.70 g, 5 mmol, 1.0 eq.), potassium carbonate (2.11 g, 15 mmol, 3.0 eq.) and methyl 2-chloro-2-methoxyacetate (technical grade 90%, 0.91 g, 6 mmol, 1.2 eq.) were reacted in 50 mL of DMF. The crude product was directly saponificated after work-up.

According to general protocol **GPII**, the crude ester and lithium hydroxide monohydrate (0.32 g, 8 mmol, 1.6 eq.) were reacted in 40 mL THF and 20mL of water. 0.271 g (1.2 mmol, 24%) of the product was obtained as a colourless solid after column chromatography (SiO_2; cyclohexane:ethyl acetate, gradient: 0 → 33% ethyl acetate +0.5 vol% AcOH).

^{1}H NMR (400 MHz, DMSO$_{d6}$) *δ* [ppm]: 13.68 (s, 1H, 1-COO*H*), 7.89 (dd, *J* = 8.1 Hz, 1.7 Hz, *H*-3'), 7.64 (ddd, 1H, *J* = 8.5 Hz, 7.4 Hz, 1.7 Hz, *H*-5'), 7.37 (dd, 1H, *J* = 8.5 Hz, 1.1 Hz, *H*-6'), 7.22 (ddd, 1H, *J* = 8.1 Hz, 7.5 Hz, 1.1 Hz, *H*-4'), 5.84 (s, 1H, *H*-2), 3.44 (s, 3H, *H*-3).

^{13}C NMR (101 MHz, DMSO$_{d6}$) *δ* [ppm]: 166.8, 148.2, 140.7, 134.1, 124.9, 122.5, 117.7, 97.9, 54.7.

HR-MS (ESI-): *m/z* for $C_9H_9NO_6$-H^-, $[M-H]^-$ calculated: 226.0357; found: 226.0360.

2-(2-Nitrophenoxy)-2-phenylacetic acid (4h)

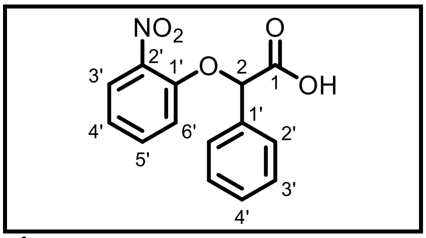

According to general protocol **GPII**, methyl 2-(2-nitrophenoxy)-2-phenylacetate (**6h**, 0.58 g, 2 mmol, 1.0 eq.) and lithium hydroxide monohydrate (0.17 g, 4 mmol, 2.0 eq.) were reacted in 40 mL THF and 20 mL of water. 0.357 g (1.3 mmol, 65%) of the product was obtained as a yellow solid after column chromatography (SiO_2; cyclohexane:ethyl acetate, gradient: 40 → 67% ethyl acetate +0.5 vol% AcOH).

^{1}H NMR (400 MHz, CD_3OD) δ [ppm]: 7.83 (dd, 1H, *J* = 8.3 Hz, 1.7 Hz, *H*-3'), 7.65 – 7.61 (m, 2H, *H*-2''), 7.52 (ddd, 1H, *J* = 8.6 Hz, 7.4 Hz, 1.7 Hz, *H*-5'), 7.42 – 7.33 (m, 3H, *H*-3'', *H*-4''), 7.19 (dd, 1H, *J* = 8.6 Hz, 1.1 Hz, *H*-6'), 7.08 h, (ddd, 1H, *J* = 8.3 Hz, 7.4 Hz, 1.1 Hz, *H*-4'), 5.94 (s, 1H, *H*-2).
^{13}C NMR (101 MHz, CD_3OD) δ [ppm]: 172.1, 151.6, 142.0, 136.5, 135.0, 130.0, 129.7, 128.3, 126.5, 122.5, 116.8, 80.2.
HR-MS (ESI-): *m/z* for $C_{14}H_{11}NO_5$-H^-, $[M-H]^-$ calculated: 272.0564; found: 272.0569.

2-(2-Chlorophenyl)-2-(2-nitrophenoxy)acetic acid (4i)

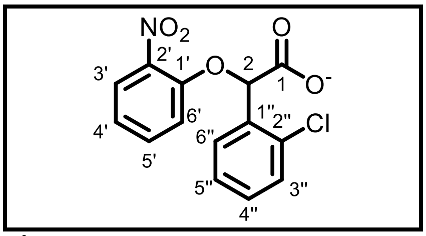

According to general protocol **GPII**, methyl 2-(2-chlorophenyl)-2-(2-nitrophenoxy)acetate (**6i**, 0.82 g, 3 mmol, 1.0 eq.) and lithium hydroxide monohydrate (0.22 g, 5 mmol, 1.7 eq.) were reacted in 40 mL THF and 20 mL of water. 0.430 g (1.4 mmol, 56%) of the product was obtained as a yellow solid after column chromatography (SiO_2; cyclohexane:ethyl acetate, gradient: 40 → 67% ethyl acetate +0.5 vol% AcOH).

^{1}H NMR (400 MHz, CD_3OD) δ [ppm]: 7.83 (dd, 1H, *J* = 8.1 Hz, 1.7 Hz, *H*-3'), 7.68 (dt, 1H, *J* = 6.9 Hz, 2.3 Hz, *H*-3''), 7.55 (ddd, 1H, *J* = 8.8 Hz, 7.5 Hz, 1.7 Hz, *H*-5'), 7.47 (dt, 1H, *J* = 6.8 Hz, 2.3 Hz, *H*-5''), 7.39 – 7.34 (m, 2H, *H*-4''. *H*-6''), 7.21 – 7.17 (m, 1H, *H*-6'), 7.15 – 7.09 (m, 1H, *H*-4'), 6.32 (s, 1H, *H*-2).
^{13}C NMR (101 MHz, CD_3OD) δ [ppm]: 171.2, 151.4, 142.2, 135.0, 134.8, 134.5, 131.7, 130.8, 130.3, 128.6, 126.5, 122.9, 117.1, 77.1.
HR-MS (ESI-): *m/z* for $C_8H_6{}^{35}ClNO_5$-H^-, $[M-H]^-$ calculated: 306.0175; found: 306.0169.

2-(4-Methoxy-2-nitrophenoxy)acetic acid (4j)

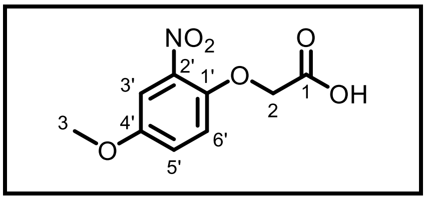

According to general protocol **GPII**, methyl 2-(4-methoxy-2-nitrophenoxy)acetate (**6j**, 1.08 g, 5 mmol, 1.0 eq.) and lithium hydroxide monohydrate (0.40 g, 10 mmol, 2.0 eq.) were reacted in 40 mL THF and 20 mL of water. 0.949 g (4.2 mmol, 93%) of the product was obtained as a colourless solid without further purification.

^{1}H NMR (400 MHz, DMSO$_{d6}$) δ [ppm]: 13.14 (s, 1H, 1-COO*H*), 7.45 – 7.43 (m, 1H, *H*-3'), 7.23 – 7.20 (m, 2H, *H*-5', *H*-6'), 4.83 (s, 2H, *H*-2), 3.77 (s, 3H, *H*-3).
^{13}C NMR (101 MHz, DMSO$_{d6}$) δ [ppm]: 169.6, 152.9, 144.5, 140.0, 120.1, 116.7, 109.4, 65.9, 56.1.
HR-MS (ESI-): *m/z* for $C_9H_9NO_6$-H^-, $[M-H]^-$ calculated: 226.0357; found: 226.0361.

2-(4-*tert*-Butyl-2-nitrophenoxy)acetic acid (4k)

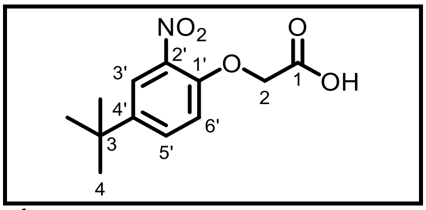

According to general protocol **GPII**, methyl 2-(4-*tert*-butyl-2-nitrophenoxy)acetate (**6k**, 1.34 g, 5 mmol, 1.0 eq.) and lithium hydroxide monohydrate (0.43 g, 10 mmol, 2.0 eq.) were reacted in 40 mL THF and 20 mL of water. 1.189 g (4.7 mmol, 94%) of the product was obtained as a colourless solid without further purification.

^{1}H NMR (400 MHz, DMSO$_{d6}$) δ [ppm]: 7.80 (d, 1H, *J* = 2.5 Hz, *H*-3'), 7.64 (dd, 1H, *J* = 8.9 Hz, 2.5 Hz, *H*-5'), 7.16 (d, 1H, *J* = 8.9 Hz, *H*-6'), 4.87 (s, 2H, *H*-2), 1.27 (s, 9H, *H*-4).
^{13}C NMR (101 MHz, DMSO$_{d6}$) δ [ppm]: 169.5, 148.2, 143.9, 139.3, 131.0, 121.4, 114.7, 65.3, 34.2, 30.9.
HR-MS (ESI-): *m/z* for $C_{12}H_{15}NO_5$-H^-, $[M-H]^-$ calculated: 252.0877; found: 252.0881.

2-((3-Nitro-biphenyl-4-yl)oxy)acetic acid (4l)

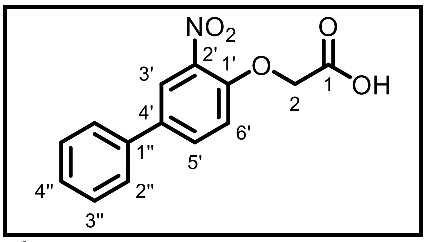

According to general protocol **GPII**, ethyl 2-((3-nitro-biphenyl-4-yl)oxy)acetate (**6l**, 3.01 g, 10 mmol, 1.0 eq.) and lithium hydroxide monohydrate (0.70 g, 17 mmol, 1.7 eq.) were reacted in 70 mL THF and 30 mL of water. 2.490 g (9.1 mmol, 91%) of the product was obtained as a colourless solid without further purification.

^{1}H NMR (400 MHz, DMSO$_{d6}$) δ [ppm]: 13.28 (s, 1H, 1-COO*H*), 8.15 (d, 1H, *J* = 2.4 Hz, *H*-3'), 7.93 (dd, 1H, *J* = 8.8 Hz, 2.4 Hz, *H*-5'), 7.73 – 7.67 (m, 2H, *H*-2''), 7.50 – 7.43 (m, 2H, *H*-3''), 7.41 – 7.33 (m, 1H, *H*-4''), 4.97 (s, 2H, *H*-2'').
^{13}C NMR (101 MHz, DMSO$_{d6}$) δ [ppm]: 169.4, 149.7, 140.2, 137.5, 133.1, 131.8, 129.1, 127.9, 126.5, 122.6, 115.6, 65.4.
HR-MS (ESI-): *m/z* for $C_{14}H_{11}NO_5$-H^-, $[M-H]^-$ calculated: 272.0564; found: 272.0568.

2-(4-Fluoro-2-nitrophenoxy)acetic acid (4m)

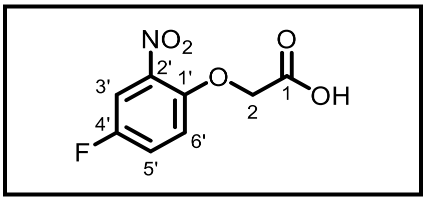

According to general protocol **GPII**, methyl 2-(4-fluoro-2-nitrophenoxy)acetate (**6m**, 1.03 g, 5 mmol, 1.0 eq.) and lithium hydroxide monohydrate (0.39 g, 9 mmol, 1.8 eq.) were reacted in 40 mL THF and 20 mL of water. 0.904 g (4.2 mmol, 93%) of the product was obtained as a colourless solid without further purification.

^{1}H NMR (400 MHz, DMSO$_{d6}$) δ [ppm]: 13.22 (s, 1H, 1-COO*H*), 7.88 (dd, 1H, *J* = 8.1 Hz, 3.2 Hz, *H*-3'), 7.54 (ddd, 1H, *J* = 9.3 Hz, 7.9 Hz, 3.2 Hz, *H*-4'), 7.33 (dd, 1H, *J* = 9.3 Hz, 4.4 Hz, *H*-5'), 4.90 (s, 2H, *H*-2).
^{13}C NMR (101 MHz, DMSO$_{d6}$) δ [ppm]: 169.3, 154.9 (d, *J* = 241.0 Hz), 147.1 (d, *J* = 2.6 Hz), 139.5 (d, *J* = 9.1 Hz), 120.8 (d, *J* = 23.1 Hz), 116.9 (d, *J* = 8.3 Hz), 112.1 (d, *J* = 28.2 Hz), 65.8.
^{19}F NMR (376 MHz, DMSO$_{d6}$) δ [ppm]: -122.06 (td, *J* = 7.9 Hz, 4.4 Hz).
HR-MS (ESI-): *m/z* for $C_8H_6FNO_5$-H^-, $[M-H]^-$ calculated: 214.0157; found: 214.0158.

2-(4-Chloro-2-nitrophenoxy)acetic acid (4n)

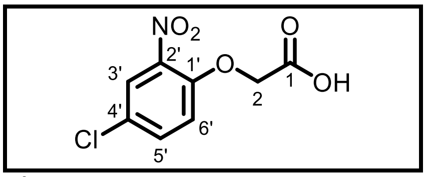

According to general protocol **GPII**, methyl 2-(4-chloro-2-nitrophenoxy)acetate (**6n**, 2.46 g, 10 mmol, 1.0 eq.) and lithium hydroxide monohydrate (1.01 g, 20 mmol, 2.0 eq.) were reacted in 40 mL THF and 20mL of water. 2.15 g (9.3 mmol, 93%) of the product was obtained as a beige solid without further purification.

^{1}H NMR (400 MHz, DMSO$_{d6}$) δ [ppm]: 13.28 (s, 1H, 1-COO*H*), 8.03 (d, 1H, *J* = 2.7 Hz, *H*-3'), 7.69 (dd, 1H, *J* = 9.1 Hz, 2.7 Hz, *H*-5'), 7.32 (d, 1H, *J* = 9.1 Hz, *H*-6'), 4.93 (s, 2H, *H*-2).
^{13}C NMR (101 MHz, DMSO$_{d6}$) δ [ppm]: 169.2, 149.3, 140.0, 133.6, 124.5, 117.0, 65.6.
HR-MS (ESI-): *m/z* for $C_8H_6{}^{35}ClNO_5$-H^-, $[M-H]^-$ calculated: 229.9862; found: 229.9868.

2-(4-Bromo-2-nitrophenoxy)acetic acid (4o)

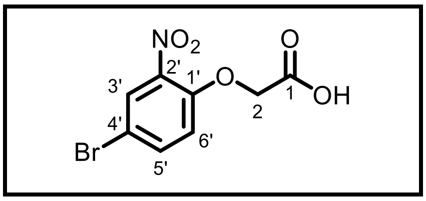

According to general protocol **GPII**, methyl 2-(4-bromo-2-nitrophenoxy)acetate (**6o**, 1.30 g, 5 mmol, 1.0 eq.) and lithium hydroxide monohydrate (0.39 g, 9 mmol, 1.8 eq.) were reacted in 40 mL THF and 20 mL of water. 1.187 g (4.3 mmol, 96%) of the product was obtained as a colourless solid without further purification.

^{1}H NMR (400 MHz, DMSO$_{d6}$) δ [ppm]: 13.27 (s, 1H, 1-COO*H*), 8.12 (d, 1H, *J* = 2.5 Hz, *H*-3'), 7.80 (dd, 1H, *J* = 9.0 Hz, 2.5 Hz, *H*-5'), 7.26 (d, 1H, *J* = 9.0 Hz, *H*-6'), 4.93 (s, 2H, *H*-2).
^{13}C NMR (101 MHz, DMSO$_{d6}$) δ [ppm]: 169.1, 149.7, 140.4, 136.4, 127.1, 117.3, 111.6, 65.5.
HR-MS (ESI-): *m/z* for $C_8H_6{}^{79}BrNO_5$-H^-, $[M-H]^-$ calculated: 273.9357; found: 273.9357.

2-(2-Nitro-4-trifluoromethylphenoxy)acetic acid (4p)

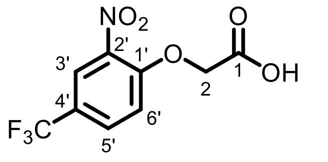

According to general protocol **GPII**, methyl 2-(2-nitro-4-trifluoromethylphenoxy)acetate (**6p**, 0.84 g, 3 mmol, 1.0 eq.) and lithium hydroxide monohydrate (0.26 g, 6 mmol, 2.0 eq.) were reacted in 40 mL THF and 20 mL of water. 0.689 g (2.6 mmol, 87%) of the product was obtained as a beige solid without further purification.

^{1}H NMR (400 MHz, DMSO$_{d6}$) δ [ppm]: 13.38 (s, 1H, 1-COO*H*), 8.31 (dd, 1H, *J* = 2.4 Hz, 0.8 Hz, *H*-3'), 7.99 (ddd, 1H, *J* = 8.9 Hz, 2.4 Hz, 0.8 Hz, *H*-5'), 7.49 (dd, 1H, *J* = 8.9, 0.9 Hz, *H*-6'), 5.05 (s, 2H, *H*-2).
^{13}C NMR (101 MHz, DMSO$_{d6}$) δ [ppm]: 168.9, 153.1, 139.5, 130.8 (d, *J* = 3.6 Hz), 123.3 (q, *J* = 271.8 Hz), 122.5 (q, *J* = 3.8 Hz), 121.7, 121.3, 65.7.
^{19}F NMR (376 MHz, DMSO$_{d6}$) δ [ppm]: -61.52.
HR-MS (ESI-): *m/z* for $C_9H_6F_3NO_5$-H^-, [M-H]$^-$ calculated: 264.0125; found: 264.0130.

2-(4-Acetyl-2-nitrophenoxy)acetic acid (4q)

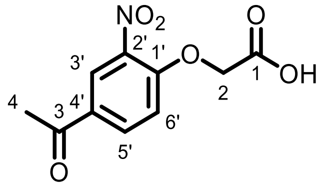

According to general protocol **GPI**, 4-acetyl-2-nitrophenol (1.84 g, 10 mmol, 1.0 eq.), potassium carbonate (4.15 g, 30 mmol, 3.0 eq.) and methyl 2-bromoacetate (2.29 g, 15 mmol, 1.5 eq.) were reacted in 30 mL of anhydrous acetone. The crude product was directly saponificated after work-up.
According to general protocol **GPII**, the crude ester and lithium hydroxide monohydrate (1.01 g, 20 mmol, 2.0 eq.) were reacted in 40 mL THF and 20mL of water. 0.831 g (3.5 mmol, 35%) of the product was obtained as a yellow solid after column chromatography (SiO_2; Cy:EA, gradient: 25 → 50% EA+0.5 vol% AcOH).

^{1}H NMR (400 MHz, CD$_3$CN) δ [ppm]: 8.33 (d, 1H, *J* = 2.2 Hz, *H*-3'), 8.12 (dd, 1H, *J* = 8.9 Hz, 2.2 Hz, *H*-5'), 7.20 (d, 1H, *J* = 8.9 Hz, *H*-6'), 4.90 (s, 2H, *H*-2), 2.54 (s, 3H, *H*-4).
^{13}C NMR (101 MHz, CD$_3$CN) δ [ppm]: 196.4, 169.1, 154.8, 140.9, 134.7, 131.6, 126.3, 115.8, 66.6, 26.8.
HR-MS (ESI-): *m/z* for $C_{10}H_9NO_6$-H^-, [M-H]$^-$ calculated: 238.0357; found: 238.0362.

2-((2-Nitrophenyl)thio)acetic acid (4r)

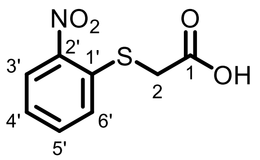

Sodium hydroxide (1.83 g, 46 mmol, 2.1 eq.) was dissolved in 15 mL of water and thioglycolic acid (1.8 mL, 25 mmol, 1.1 eq.) was added at 10 °C. 2-fluoronitrobenzene (3.11 g, 22 mmol, 1.0 eq.) dissolved in 100 mL of ethanol was added dropwise. The mixture was refluxed for 1 h. After completion of the reaction the mixture was added to 50 mL of water and acidified using 1 M hydrochloric acid. The solids were filtered and dried under reduced pressure. 3.433 g (16.1 mmol, 73%) of the product was obtained as a yellow solid.

^{1}H NMR (400 MHz, DMSO$_{d6}$) δ [ppm]: 13.01 (s, 1H, 1-COO*H*), 8.21 (dd, 1H, *J* = 8.3 Hz, 1.5 Hz, *H*-3'), 7.73 (ddd, 1H, *J* = 8.6 Hz, 7.2 Hz, 1.5 Hz, *H*-5'), 7.58 (dd, 1H, *J* = 8.4 Hz, 1.3 Hz, *H*-4'), 7.41 (ddd, *J* = 8.4 Hz, 7.2 Hz, 1.3 Hz, 1H), 4.01 (s, 2H, *H*-2).
^{13}C NMR (101 MHz, DMSO$_{d6}$) δ [ppm]: 170.0, 145.5, 135.9, 134.3, 127.3, 125.9, 125.6, 34.4.
HR-MS (ESI-): *m/z* for $C_8H_7NO_4S$-H^-, [M-H]$^-$ calculated: 212.0023; found: 212.0027.
Known compound and literature, spectroscopic data match to literature.[5]

6.3. 4-Hydroxybenzo[*b*]-1,4-oxazin-3-ones (5a-5r)

2*H*,4*H*-4-Hydroxybenzo[*b*]-1,4-oxazin-3-one (5a, D-DIBOA)

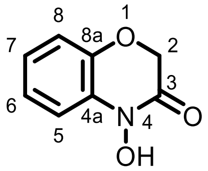

According to general protocol **GPIII**, 2-(2-nitrophenoxy)acetic acid (**4a**, 29.6 mg, 0.15 mmol), were reacted. 14.9 mg (0.090 mmol, 60%) of the product was obtained as an off-white solid by reversed phase column chromatography (C_{18} silica, gradient: 0 → 15% acetonitrile).

^{1}H NMR (400 MHz, DMSO$_{d6}$) δ [ppm]: 10.74 (s, 1H, 4-N-O*H*), 7.25 – 7.21 (m, 1H, *H*-5), 7.08 – 6.96 (m, 3H, *H*-6, *H*-7, *H*-8), 4.76 (s, 2H, *H*-2).
^{13}C NMR (101 MHz, DMSO$_{d6}$) δ [ppm]: 160.2, 143.6, 129.6, 123.7, 122.5, 115.9, 113.0, 67.8.
HR-MS (ESI+): *m/z* for $C_8H_7NO_3$+H^+, $[M+H]^+$ calculated: 166.0499; found: 166.0500.
Known compound and literature, spectroscopic data match to literature.[6]

2*H*,4*H*-4-Hydroxy-2-methylbenzo[*b*]-1,4-oxazin-3-one (5b)

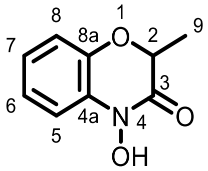

According to general protocol **GPIII**, 2-(2-nitrophenoxy)propionic acid (**4b**, 31.7 mg, 0.15 mmol), were reacted. 21.7 mg (0.121 mmol, 81%) of the product was obtained as an off-white solid by reversed phase column chromatography (C_{18} silica, gradient: 20 → 70% acetonitrile).

^{1}H NMR (400 MHz, DMSO$_{d6}$) δ [ppm]: 10.80 (s, 1H, 4-N-O*H*), 7.25 – 7.19 (m, 1H, *H*-5), 7.10 – 6.96 (m, 3H, *H*-6, *H*-7, *H*-8), 4.87 (q, 1H, *J* = 6.7 Hz, *H*-2), 1.46 (d, 3H, *J* = 6.7 Hz, *H*-9).
^{13}C NMR (101 MHz, DMSO$_{d6}$) δ [ppm]: 162.3, 142.7, 129.8, 123.8, 122.7, 116.2, 113.1, 74.0, 16.4.
HR-MS (ESI+): *m/z* for $C_9H_9NO_3$+Na^+, $[M+Na]^+$ calculated: 202.0475; found: 202.0446.

2*H*,4*H*-2,2-Dimethyl-4-hydroxybenzo[*b*]-1,4-oxazin-3-one (5c)

0.15 mmol scale: According to general protocol **GPIII**, 2-methyl-2-(2-nitrophenoxy)propanoic acid (**4a**, 34.4 mg, 0.15 mmol), were reacted. 23.3 mg (0.121 mmol, 81%) of the product was obtained as an off-white solid.

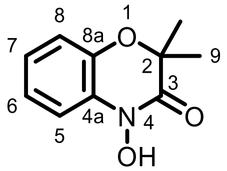

0.75 mmol scale: According to **GPIII**, (**4a**, 168.8 mg, 0.75 mmol), were reacted. 110.9 mg (0.574 mmol, 77%).
3.0 mmol scale: According to **GPIII**, (**4a**, 676 mg, 3.0 mmol), were reacted. 503 mg (2.6 mmol, 87%).
Purification 0.15–3.0 mmol scale: reversed phase column chromatography (C_{18} silica, gradient: 20 → 70% acetonitrile).

7.5 mmol scale: According to **GPIII**, (**4a**, 1.69 g, 7.5 mmol), were reacted. 1.27 g (6.6 mmol, 88%) of the product was obtained as a beige solid after crystallisation (30 mL MeCN; crystallisation at 6 °C).

^{1}H NMR (400 MHz, DMSO$_{d6}$) δ [ppm]: 10.76 (s, 1H, 4-N-O*H*), 7.20 (dd, 1H, *J* = 7.8 Hz, 1.7 Hz, *H*-5), 7.05 (td, 1H, *J* = 7.8 Hz, 1.8 Hz, *H*-6), 7.00 (td, 1H, *J* = 7.5, 1.7 Hz, *H*-7), 6.96 (dd, 1H, *J* = 7.5, 1.8 Hz, *H*-8), 1.43 (s, 6H, *H*-9).
^{13}C NMR (101 MHz, DMSO$_{d6}$) δ [ppm]: 164.0, 141.2, 129.6, 123.9, 122.5, 116.7, 112.7, 79.1, 23.6.
HR-MS (ESI+): *m/z* for $C_{10}H_{11}NO_3$+H^+, $[M+H]^+$ calculated: 194.0812; found: 194.0804.
m.p. (MeCN): 132.3–134.1 °C.

2*H*,4*H*-2-Ethyl-4-hydroxybenzo[*b*]-1,4-oxazin-3-one (5d)

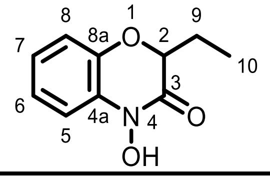

According to general protocol **GPIII**, 2-(2-nitrophenoxy)butyric acid (**4d**, 33.7 mg, 0.15 mmol), were reacted. 21.1 mg (0.109 mmol, 73%) of the product was obtained as an off-white solid by reversed phase column chromatography (C_{18} silica, gradient: 20 → 60% acetonitrile).

^{1}H NMR (400 MHz, DMSO$_{d6}$) δ [ppm]: 10.77 (s, 1H, 4-N-O*H*), 7.21 (dd, 1H, *J* = 7.4 Hz, 1.1 Hz, *H*-5), 7.07 – 6.98 (m, 3H, *H*-6, *H*-7, *H*-8), 4.73 – 4.69 (m, 1H, *H*-2), 1.92 – 1.73 (m, 2H, *H*-9), 0.99 (t, 3H, *J* = 7.4 Hz, *H*-10).
^{13}C NMR (101 MHz, DMSO$_{d6}$) δ [ppm]: 161.8, 142.5, 129.6, 123.8, 122.5, 116.3, 112.9, 78.4, 23.6, 9.1.
HR-MS (ESI-): *m/z* for $C_{10}H_{11}NO_3$-H$^-$, [M-H]$^-$ calculated: 192.0666; found: 192.0672.
Known compound and literature, spectroscopic data match to literature.[7]

4*H*-4-Hydroxyspiro[benzo[*b*]-1,4-oxazine-2,1'-cyclobutan]-3-one (5e)

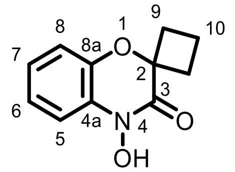

According to general protocol **GPIII**, 1-(2-nitrophenoxy)-cyclobutane-1-carboxylic acid (**4e**, 35.7 mg, 0.15 mmol), were reacted. 21.1 mg (0.103 mmol, 69%) of the product was obtained as an off-white solid by reversed phase column chromatography (C_{18} silica, gradient: 35 → 50% acetonitrile).

^{1}H NMR (400 MHz, DMSO$_{d6}$) δ [ppm]: 10.79 (s, 1H, 4-N-O*H*), 7.20 (dd, 1H, *J* = 7.8 Hz, 1.5 Hz, *H*-5), 7.10 – 6.97 (m, 3H, *H*-6, *H*-7, *H*-8), 2.55 – 2.45 (m, 2H, *H*-9), 2.31 – 2.21 (m, 1H, *H*-9), 2.00 – 1.89 (m, 1H, *H*-9), 1.87 – 1.74 (m, 2H, *H*-10).
^{13}C NMR (101 MHz, DMSO$_{d6}$) δ [ppm]: 162.9, 141.2, 129.7, 123.8, 122.9, 116.8, 112.9, 80.3, 31.0, 12.8.
HR-MS (ESI-): *m/z* for $C_{11}H_{11}NO_3$-H$^-$, [M-H]$^-$ calculated: 204.0666; found: 204.0661.

2*H*,4*H*-2-(*n*-Hexyl)-4-hydroxybenzo[*b*]-1,4-oxazin-3-one (5f)

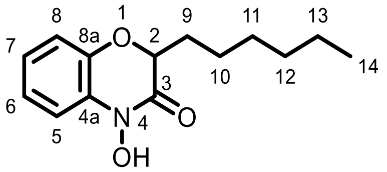

According to general protocol **GPIII**, 2-(2-nitrophenoxy)caprylic acid (**4f**, 42.0 mg, 0.15 mmol), were reacted. 22.6 mg (0.0907 mmol, 61%) of the product was obtained as an off-white solid by reversed phase column chromatography (C_{18} silica, gradient: 50 → 60% acetonitrile).

^{1}H NMR (400 MHz, CD_3OD) δ [ppm]: 7.31 – 7.27 (m, 1H, *H*-5), 7.08 – 7.00 (m, 2H, *H*-6, *H*-7), 6.98 – 6.93 (m, 1H, *H*-8), 4.68 (dd, 1H, *J* = 8.0 Hz, 4.7 Hz, *H*-2), 1.93 – 1.77 (m, 2H, *H*-9), 1.62 – 1.43 (m, 2H, *H*-10), 1.41 – 1.24 (m, 6H, *H*-11, *H*-12, *H*-13), 0.93 – 0.87 (m, 3H, *H*-14).
^{13}C NMR (101 MHz, CD_3OD) δ [ppm]: 164.5, 144.3, 130.5, 125.6, 123.6, 117.6, 114.3, 79.3, 32.8, 31.6, 29.9, 25.8, 23.6, 14.4.
HR-MS (ESI+): *m/z* for $C_{14}H_{19}NO_3$+H$^+$, [M+H]$^+$ calculated: 250.1438; found: 250.1435.

2*H*,4*H*-4-Hydroxy-2-methylbenzo[*b*]-1,4-oxazin-3-one (5g)

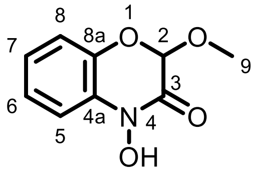

According to general protocol **GPIII**, 2-methoxy-2-(2-nitrophenoxy)acetic acid (**4g**, 34.2 mg, 0.15 mmol), were reacted. 20.5 mg (0.105 mmol, 70%) of the product was obtained as an off-white solid by reversed phase column chromatography (C_{18} silica, gradient: 20 → 30% acetonitrile).

^{1}H NMR (400 MHz, DMSO$_{d6}$) δ [ppm]: 11.08 (s, 1H, 4-N-O*H*), 7.31 – 7.26 (m, 1H, *H*-5), 7.17 – 7.04 (m, 3H, *H*-6, *H*-7, *H*-8), 5.55 (s, 1H, *H*-2), 3.44 (s, 3H, *H*-9).
^{13}C NMR (101 MHz, DMSO$_{d6}$) δ [ppm]: 155.9, 140.0, 128.8, 124.2, 123.2, 117.2, 113.2, 97.8, 55.9.
HR-MS (ESI-): *m/z* for $C_9H_9NO_4$-H$^-$, [M-H]$^-$ calculated: 194.0459; found: 194.0465.
Known compound and literature, spectroscopic data match to literature.[6]

2*H*,4*H*-4-Hydroxy-2-phenylbenzo[*b*]-1,4-oxazin-3-one (5h)

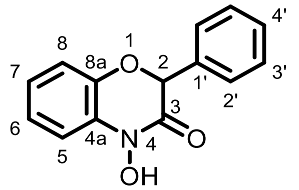

According to general protocol **GPIII**, 2-(2-nitrophenoxy)-2-phenylacetic acid (**4g**, 41.4 mg, 0.15 mmol), were reacted. 21.4 mg (0.0887 mmol, 59%) of the product was obtained as an off-white solid by reversed phase column chromatography (C_{18} silica, gradient: 35 → 50% acetonitrile).

^{1}H NMR (600 MHz, DMSO$_{d6}$) δ [ppm]: 11.14 (s, 1H, 4-N-O*H*), 7.40 – 7.34 (m, 5H, *H*-2', *H*-3', *H*-4'), 7.25 (dd, 1H, *J* = 7.9 Hz, 1.5 Hz, *H*-5), 7.07 – 6.98 (m, 3H, *H*-6, *H*-7, *H*-8), 5.99 (s, 1H, *H*-7).
^{13}C NMR (151 MHz, DMSO$_{d6}$) δ [ppm]: 160.2, 142.4, 135.6, 129.3, 129.0, 128.7, 127.2, 124.1, 122.8, 116.6, 113.2, 78.9.
HR-MS (ESI-): *m/z* for $C_{14}H_{11}NO_3$-H^-, $[M-H]^-$ calculated: 240.0666; found: 240.0675.

2*H*,4*H*-4-Hydroxy-2-phenylbenzo[*b*]-1,4-oxazin-3-one (5i)

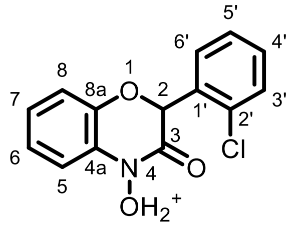

According to general protocol **GPIII**, 2-(2-chlorophenyl)-(2-nitrophenoxy)acetic acid (**4h**, 48.2 mg, 0.15 mmol), were reacted. 24.3 mg (0.0883 mmol, 59%) of the product was obtained as an off-white solid by reversed phase column chromatography (C_{18} silica, gradient: 35 → 60% acetonitrile).

^{1}H NMR (400 MHz, DMSO$_{d6}$) δ [ppm]: 11.02 (s, 1H, 4-N-O*H*), 7.56 (dd, 1H, *J* = 8.0 Hz, 1.3 Hz, *H*-3'), 7.48 – 7.42 (m, 2H, *H*-5', *H*-6'), 7.38 (ddd, 1H, *J* = 8.3 Hz, 6.8 Hz, 1.3 Hz, *H*-4'), 7.30 (dd, 1H, *J* = 7.9 Hz, 1.5 Hz, *H*-5), 7.09 (td, 1H, *J* = 7.6 Hz, 1.7 Hz, *H*-6), 7.03 – 6.95 (m, 2H, *H*-7, *H*-8), 6.16 (s, 1H, *H*-2).
^{13}C NMR (101 MHz, DMSO$_{d6}$) δ [ppm]: 159.9, 142.4, 133.6, 133.2, 131.1, 130.5, 130.1, 129.4, 127.4, 124.0, 122.8, 116.2, 113.1, 77.4.
HR-MS (ESI+): *m/z* for $C_{14}H_{10}{}^{35}ClNO_3+H^+$, $[M+H]^+$ calculated: 276.0422; found: 276.0418.

2*H*,4*H*-4-Hydroxy-6-methoxybenzo[*b*]-1,4-oxazin-3-one (5j)

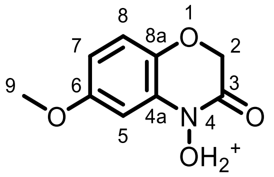

According to general protocol **GPIII**, 2-(4-methoxy-2-nitrophenoxy)acetic acid (**4i**, 33.6 mg, 0.15 mmol), were reacted. 12.4 mg (0.0635 mmol, 42%) of the product was obtained as an off-white solid by reversed phase column chromatography (C_{18} silica, gradient: 10 → 90% acetonitrile).

^{1}H NMR (400 MHz, DMSO$_{d6}$) δ [ppm]: 10.80 (s, 1H, 4-N-O*H*), 6.91 (d, 1H, *J* = 8.7 Hz, *H*-8), 6.77 (d, 1H, *J* = 2.9 Hz, *H*-5), 6.55 (dd, 1H, *J* = 8.7 Hz, 2.9 Hz, *H*-7), 4.68 (s, 2H, *H*-2), 3.72 (s, 3H, *H*-9).
^{13}C NMR (101 MHz, DMSO$_{d6}$) δ [ppm]: 160.8, 154.9, 137.4, 130.5, 116.5, 107.9, 99.3, 67.9, 55.6.
HR-MS (ESI+): *m/z* for $C_9H_9NO_4+H^+$, $[M+H]^+$ calculated: 196.0604; found: 196.0609.
Known compound and literature, spectroscopic data match to literature.[8]

2*H*,4*H*-6-*tert*-Butyl-4-hydroxybenzo[*b*]-1,4-oxazin-3-one (5k)

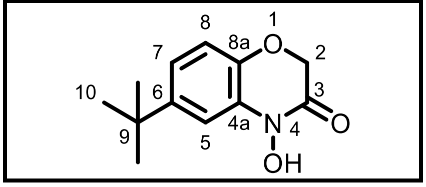

According to general protocol **GPIII**, 2-(4-*tert*-butyl-2-nitrophenoxy)acetic acid (**4j**, 37.9 mg, 0.15 mmol), were reacted. 12.1 mg (0.0547 mmol, 36%) of the product was obtained as an off-white solid by reversed phase column chromatography (C_{18} silica, gradient: 35 → 45% acetonitrile).

^{1}H NMR (400 MHz, DMSO$_{d6}$) δ [ppm]: 10.75 (s, 1H, 4-N-O*H*), 7.23 (d, *J* = 2.3 Hz, 1H, *H*-5), 7.01 (dd, *J* = 8.4 Hz, 2.3 Hz, 1H, *H*-7), 6.90 (d, *J* = 8.4 Hz, 1H, *H*-8), 4.71 (s, 2H, *H*-2), 1.26 (s, 9H, *H*-10).
^{13}C NMR (101 MHz, DMSO$_{d6}$) δ [ppm]: 160.3, 145.2, 141.5, 129.0, 120.3, 115.4, 109.9, 67.9, 34.2, 31.2.
HR-MS (ESI-): *m/z* for $C_{12}H_{15}NO_3$-H^-, $[M-H]^-$ calculated: 220.0979; found: 220.0972.

2*H*,4*H*-4-Hydroxy-6-phenylbenzo[*b*]-1,4-oxazin-3-one (5l)

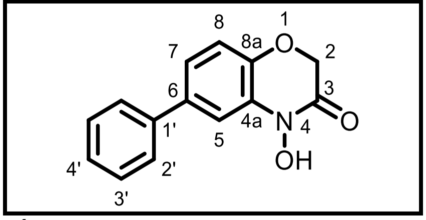

According to general protocol **GPIII**, 2-(2-nitro-4-phenyl-phenoxy)acetic acid (**4k**, 40.9 mg, 0.15 mmol), were reacted. 18.8 mg (0.0781 mmol, 52%) of the product was obtained as an off-white solid by reversed phase column chromatography (C_{18} silica, gradient: 40 → 50% acetonitrile).

^{1}H NMR (400 MHz, DMSO$_{d6}$) δ [ppm]: 10.96 (s, 1H, 4-N-O*H*), 7.64 – 7.57 (m, 2H, *H*-2'), 7.48 – 7.42 (m, 3H, *H*-5, *H*-3'), 7.39 – 7.32 (m, 1H, *H*-4), 7.29 (dd, 1H, *J* = 8.3 Hz, 2.1 Hz, *H*-7), 7.07 (d, 1H, *J* = 8.3 Hz, *H*-8), 4.81 (s, 2H, *H*-2).
^{13}C NMR (101 MHz, DMSO$_{d6}$) δ [ppm]: 160.2, 143.2, 139.5, 134.8, 130.0, 129.0, 127.3, 126.4, 121.9, 116.5, 111.0, 67.9.
HR-MS (ESI-): *m/z* for $C_{14}H_{11}NO_3$-H^-, $[M-H]^-$ calculated: 240.0666; found: 240.0665.

2*H*,4*H*-6-Fluoro-4-hydroxybenzo[*b*]-1,4-oxazin-3-one (5m)

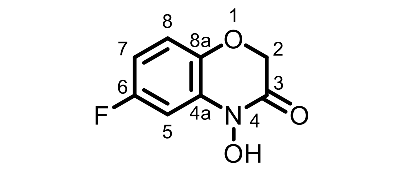

According to general protocol **GPIII**, 2-(4-fluoro-2-nitro-phenoxy)acetic acid (**4j**, 32.4 mg, 0.15 mmol), were reacted. 21.9 mg (0.120 mmol, 80%) of the product was obtained as an off-white solid by reversed phase column chromatography (C_{18} silica, gradient: 20 → 40% acetonitrile).

^{1}H NMR (400 MHz, DMSO$_{d6}$) δ [ppm]: 10.97 (s, 1H, 4-N-O*H*), 7.04 – 6.95 (m, 2H, *H*-5, *H*-7), 6.88 – 6.72 (m, 1H, *H*-8), 4.75 (s, 2H, *H*-2).
^{13}C NMR (101 MHz, DMSO$_{d6}$) δ [ppm]: 160.6, 158.8, 156.4, 139.8 (d, *J* = 2.2 Hz), 130.9 (d, *J* = 11.0 Hz), 117.0 (d, *J* = 9.2 Hz), 109.3 (d, *J* = 23.3 Hz), 100.5 (d, *J* = 29.7 Hz), 67.8.
^{19}F NMR (101 MHz, DMSO$_{d6}$) δ [ppm]: -120.79 (d, *J* = 5.2 Hz).
HR-MS (ESI-): *m/z* for $C_8H_6FNO_3$-H^-, $[M-H]^-$ calculated: 182.0259; found: 182.0264.
Known compound and literature, spectroscopic data match to literature.[9]

2*H*,4*H*-6-Chloro-4-hydroxybenzo[*b*]-1,4-oxazin-3-one (5n)

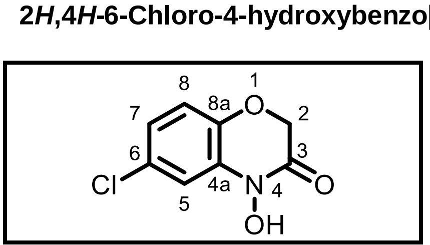

According to general protocol **GPIII**, 2-(4-chloro-2-nitro-phenoxy)acetic acid (**4m**, 33.8 mg, 0.15 mmol), were reacted. 18.5 mg (0.101 mmol, 67%) of the product was obtained as an off-white solid by reversed phase column chromatography (C_{18} silica, gradient: 20 → 40% acetonitrile).

^{1}H NMR (400 MHz, DMSO$_{d6}$) δ [ppm]: 10.93 (s, 1H, 4-N-O*H*), 7.18 (d, 1H, *J* = 2.2 Hz, *H*-5), 7.06 – 6.99 (m, 2H, *H*-7, *H*-8), 4.80 (s, 2H, *H*-2).
^{13}C NMR (101 MHz, DMSO$_{d6}$) δ [ppm]: 160.3, 142.4, 130.9, 126.3, 123.1, 117.5, 112.5, 67.8.
HR-MS (ESI-): *m/z* for $C_8H_6{}^{35}ClNO_3$-H^-, $[M-H]^-$ calculated: 197.9963; found: 197.9969.
Known compound and literature, spectroscopic data match to literature.[9]

2*H*,4*H*-6-Bromo-4-hydroxybenzo[*b*]-1,4-oxazin-3-one (5o)

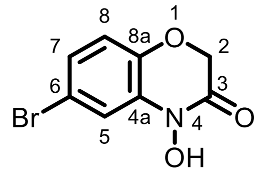

According to general protocol **GPIII**, 2-(4-bromo-2-nitro-phenoxy)acetic acid (**4n**, 41.4 mg, 0.15 mmol), were reacted. 26.5 mg (0.109 mmol, 73%) of the product was obtained as an off-white solid by reversed phase column chromatography (C_{18} silica, gradient: 30 → 40% acetonitrile).

^{1}H NMR (400 MHz, DMSO$_{d6}$) δ [ppm]: 11.04 (s, 1H, 4-N-O*H*), 7.35 – 7.26 (m, 1H, *H*-5), 7.22 – 7.08 (m, 1H, *H*-7), 7.06 – 6.85 (m, 1H, *H*-8), 4.79 (s, 2H, *H*-2).
^{13}C NMR (101 MHz, DMSO$_{d6}$) δ [ppm]: 160.2, 142.9, 131.1, 126.0, 117.9, 115.3, 113.7, 67.8.
HR-MS (ESI-): *m/z* for $C_8H_6{}^{79}BrNO_3$-H^-, $[M-H]^-$ calculated: 241.9458; found: 241.9466.

2*H*,4*H*-4-Hydroxy-trifluoromethylbenzo[*b*]-1,4-oxazin-3-one (5p)

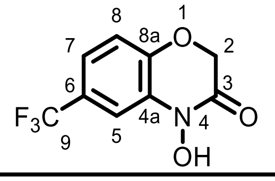

According to general protocol **GPIII**, 2-(4-trifluoromethyl-2-nitro-phenoxy)acetic acid (**4p**, 39.9 mg, 0.15 mmol), were reacted. 20.9 mg (0.0896 mmol, 60%) of the product was obtained as an off-white solid by reversed phase column chromatography (C_{18} silica, gradient: 35 → 45% acetonitrile).

^{1}H NMR (400 MHz, DMSO$_{d6}$) δ [ppm]: 11.13 (s, 1H, 4-N-O*H*), 7.43 (d, 1H, *J* = 2.2 Hz, *H*-5), 7.36 (dd, 1H, *J* = 8.5 Hz, 2.2 Hz, *H*-7), 7.17 (d, 1H, *J* = 8.5 Hz, *H*-8), 4.89 (s, 2H, *H*-2).
^{13}C NMR (101 MHz, DMSO$_{d6}$) δ [ppm]: 159.9, 146.5, 130.2, 124.1 (q, *J* = 271.4 Hz), 123.0 (q, *J* = 32.4 Hz), 120.9 (q, *J* = 3.9 Hz), 116.7, 109.5 (q, *J* = 4.0 Hz), 67.9.
^{19}F NMR (376 MHz, DMSO$_{d6}$) δ [ppm]: -60.41.
HR-MS (ESI-): *m/z* for $C_9H_6F_3NO_3$-H^-, $[M-H]^-$ calculated: 232.0227; found: 232.0228.
Known compound and literature, spectroscopic data match to literature.[9]

2*H*,4*H*-6-Acetyl-4-hydroxybenzo[*b*]-1,4-oxazin-3-one (5q)

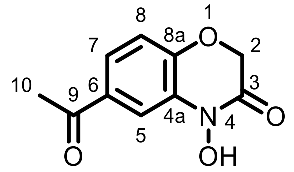

According to general protocol **GPIII**, 2-(4-acetyl-2-nitro-phenoxy)acetic acid (**4q**, 35.9 mg, 0.15 mmol), were reacted. 21.3 mg (0.0896 mmol, 60%) of the product was obtained as an off-white solid by reversed phase column chromatography (C_{18} silica, gradient: 15 → 30% acetonitrile).

^{1}H NMR (400 MHz, DMSO$_{d6}$) δ [ppm]: 11.07 (s, 1H, 4-N-O*H*), 7.75 – 7.71 (m, 1H, *H*-5), 7.69 – 7.65 (m, 1H, *H*-7), 7.12 – 6.95 (m, 1H, *H*-8), 4.89 (s, 2H, *H*-2), 2.54 (s, 3H, *H*-10).
^{13}C NMR (101 MHz, DMSO$_{d6}$) δ [ppm]: 196.2, 159.6, 147.5, 131.4, 129.5, 125.2, 115.9, 112.3, 67.9, 26.5.
HR-MS (ESI-): *m/z* for $C_{10}H_9NO_4$-H^-, $[M-H]^-$ calculated: 206.0459; found: 206.0463.

2*H*,4*H*-4-Hydroxybenzo[*b*]-1,4-thiazin-3-one (5r)

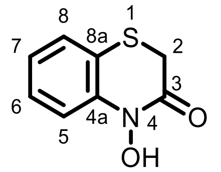

According to general protocol **GPIII**, 2-((2-nitrophenyl)thio)acetic acid (**4q**, 31.7 mg, 0.15 mmol), were reacted. 11.5 mg (0.0635 mmol, 42%) of the product was obtained as an off-white solid by reversed phase column chromatography (C_{18} silica, gradient: 20% acetonitrile isocratic).

^{1}H NMR (400 MHz, DMSO$_{d6}$) δ [ppm]: 10.76 (s, 1H, 4-N-O*H*), 7.38 – 7.33 (m, 2H, *H*-5, *H*-8), 7.28 (td, 1H, *J* = 7.3 Hz, 1.4 Hz, *H*-6), 7.03 (td, 1H, *J* = 7.5 Hz, 1.3 Hz, *H*-7), 3.66 (s, 2H, *H*-2).
^{13}C NMR (101 MHz, DMSO$_{d6}$) δ [ppm]: 161.0, 139.5, 127.3, 127.1, 123.4, 120.0, 115.4, 29.9.
HR-MS (ESI-): *m/z* for $C_8H_7NO_2S$-H^-, $[M-H]^-$ calculated: 180.0125; found: 180.0126.
Known compound and literature, spectroscopic data match to literature.[10]

2*H*,4*H*-Benzo[*b*]-1,4-oxazin-3-one (5s)

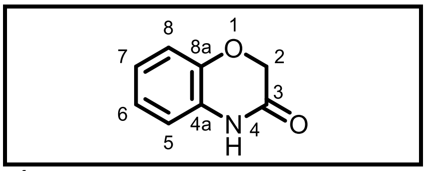

According to general protocol **GPIII**, using a zinc electrode, methyl 2-(2-nitrophenoxy)acetate (**4a**, 42.2 mg, 0.20 mmol), were reacted. **No electricity was applied.** 26.4 mg (0.177 mmol, 89%) of the product was obtained as an off-white solid by flash column chromatography (SiO_2, gradient: 5 → 47% ethyl acetate).

^{1}H NMR (400 MHz, DMSO$_{d6}$) δ [ppm]: 10.69 (s, 1H, 4-N*H*), 6.98 – 6.86 (m, 4H, *H*-5, *H*-6, *H*-7, *H*-8), 4.55 (s, 2H, *H*-2).

^{13}C NMR (101 MHz, DMSO$_{d6}$) δ [ppm]: 165.0, 143.3, 127.3, 123.1, 122.5, 116.2, 115.9, 66.8.

HR-MS (ESI+): *m/z* for $C_8H_7NO_2$+H$^+$, [M+H]$^+$ calculated: 150.0550; found: 150.0553.

Known compound and literature, spectroscopic data match to literature.[11]

7. Crystallographic Data

2*H*,4*H*- 2,2-Dimethyl-4-hydroxy-benzo[*b*]-1,4-oxazin-3-one (5c)

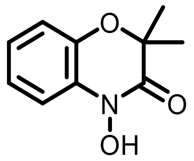

Crystallization was carried out by dissolving the compound in acetonitrile. Slow evaporating resulted in crystal formation.

CCDC Number	2349053
Empirical formular	$C_{10}H_{11}NO_3$
Moiety formular	$C_{10}H_{11}NO_3$
Formular weight	193.20 g·mol^{-1}
Temperature	120(2) K
Wavelength, radiation type	0.71073 Å, MoKα
Diffractometer	STOE IPDS 2T
Crystal system	monoclinic
Space group name, number	P 2_1/c, (14)
Unit cell dimensions	a = 9.9227(7) Å, α = 90°
	b = 5.6720(5) Å, β = 99.498(6)°
	c = 16.1656(12) Å, γ = 90°
Volume	897.35(12) Å^3
Number of reflections	10411
And range used for lattice parameters	3.02° ≤ Θ ≤ 28.29°
Z	4
Density (calculated)	1.430 Mg/m^3
Absorption coefficient	0.107 mm^{-1}
Absorption correction	none
F(000)	408
Crystal size, colour and form	0.200 · 0.230 · 0.590 mm^3, colorless block
Theta range for data collection	3.018 to 28.045°.
Index ranges	-13 ≤ h ≤ 12, -7 ≤ k ≤ 7, -21 ≤ l ≤ 21
Number of reflections:	
collected	4613
independent	2133 [R_{int} = 0.0186]
observed [I>2sigma(I)]	1864
Completeness to theta = 25.2°	99.5%
Refinement method	Full-matrix least-squares on F^2
Data / restraints / parameters	2133 / 0 / 167
Goodness-of-fit on F^2	1.074
Final R indices [I>2sigma(I)]	R1 = 0.0374, wR2 = 0.0921
R indices (all data)	R1 = 0.0455, wR2 = 0.0991
Largest diff. peak and hole	0.277 und -0.163 eÅ^{-3}

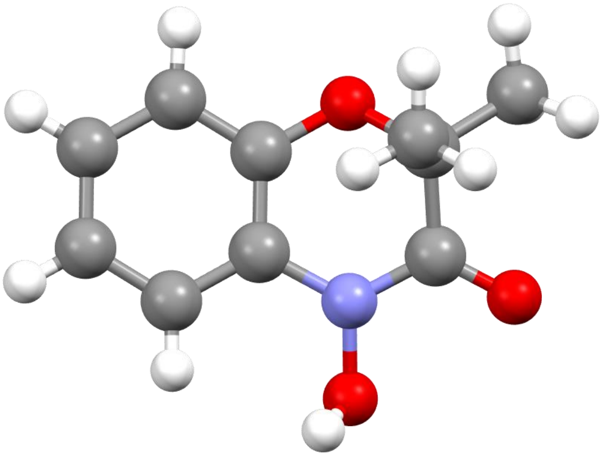

Figure S10: Molecular structure of **5c.**

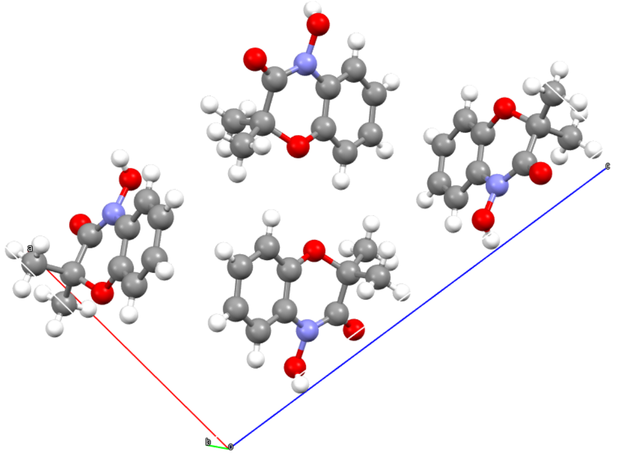

Figure S11: Packing of **5c**.

8. NMR Spectra

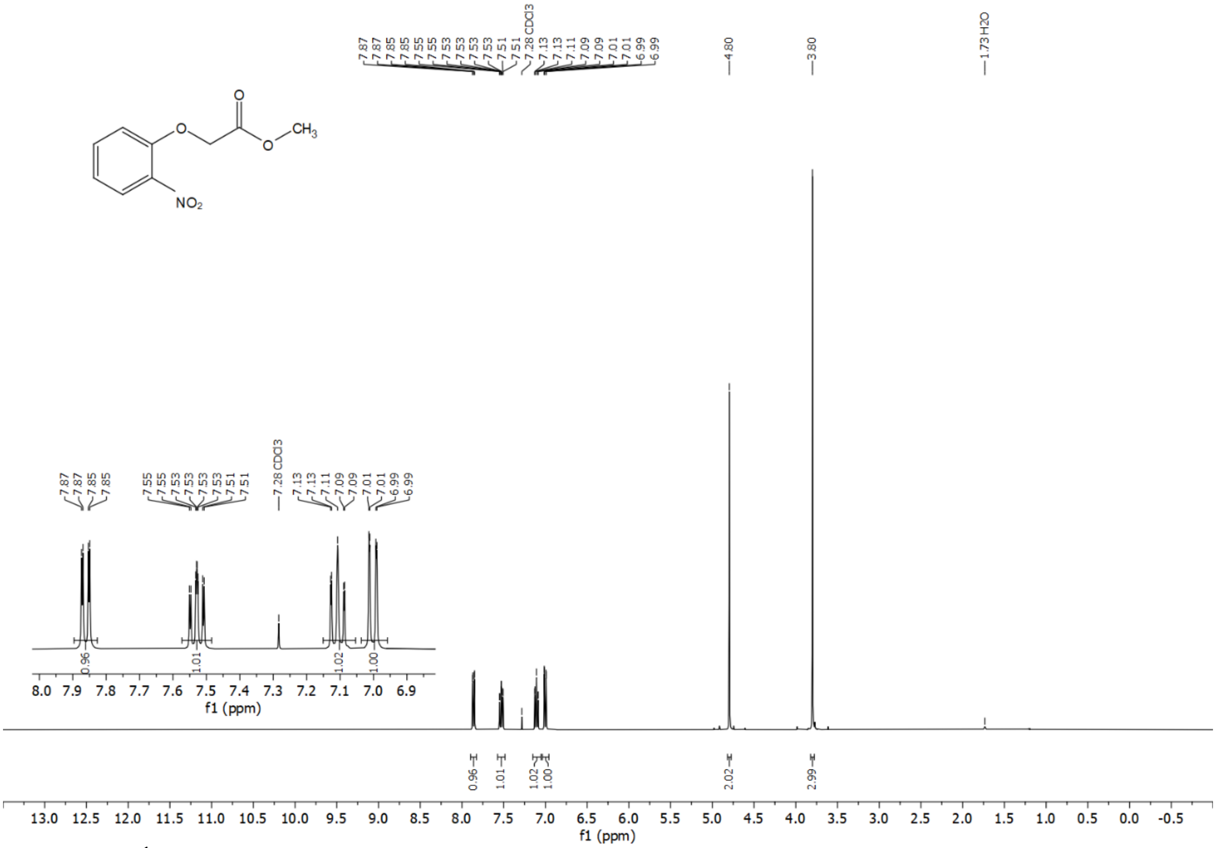

Figure S12: ^{1}H NMR spectrum (400 MHz, $CDCl_3$) of **6a**.

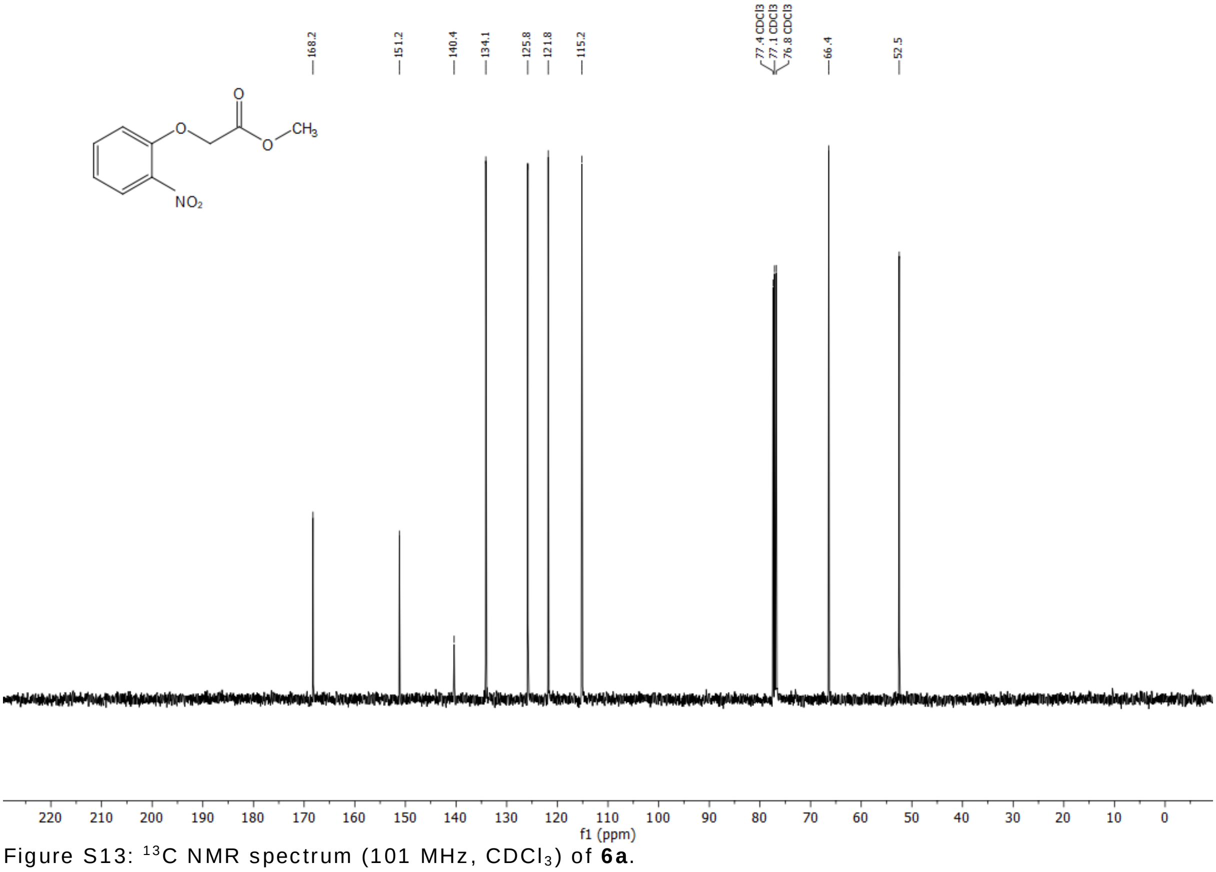

Figure S13: ^{13}C NMR spectrum (101 MHz, $CDCl_3$) of **6a**.

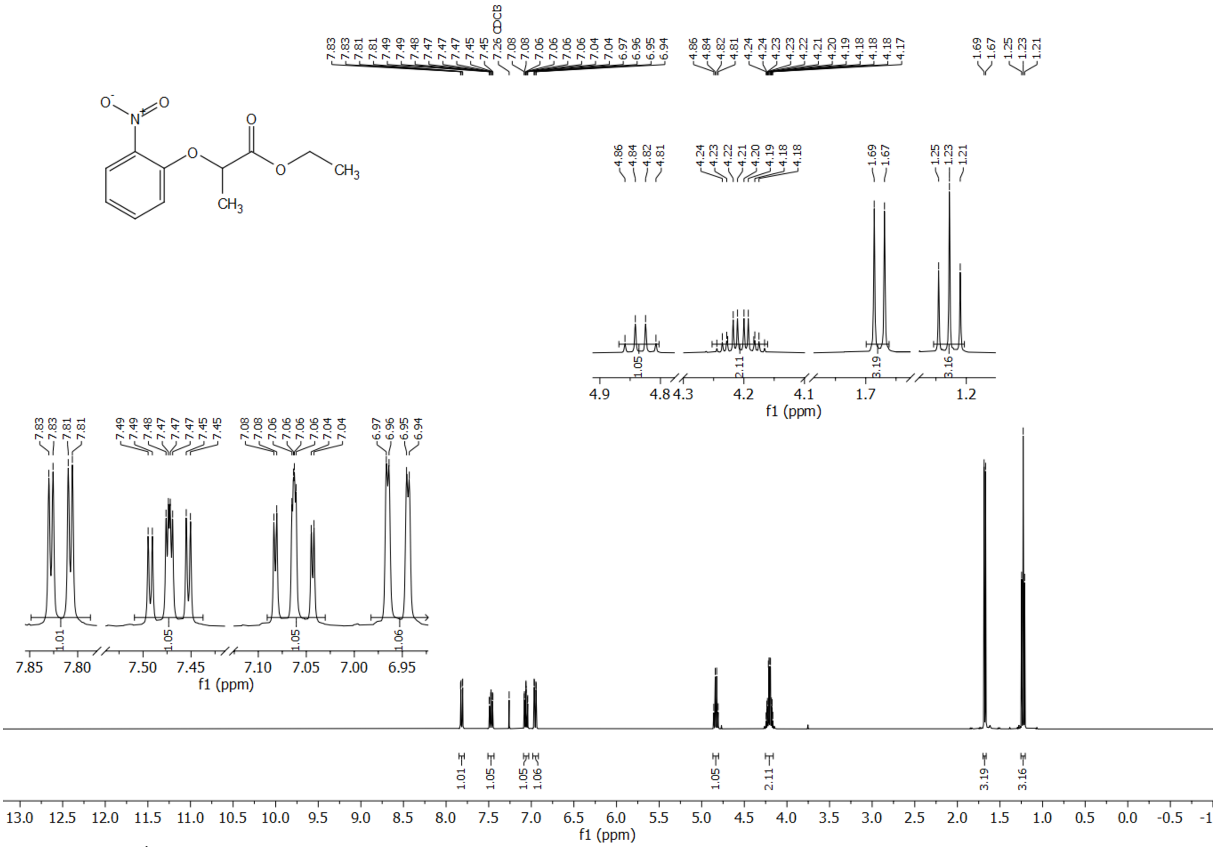

Figure S14: ^{1}H NMR spectrum (400 MHz, $CDCl_3$) of **6b**.

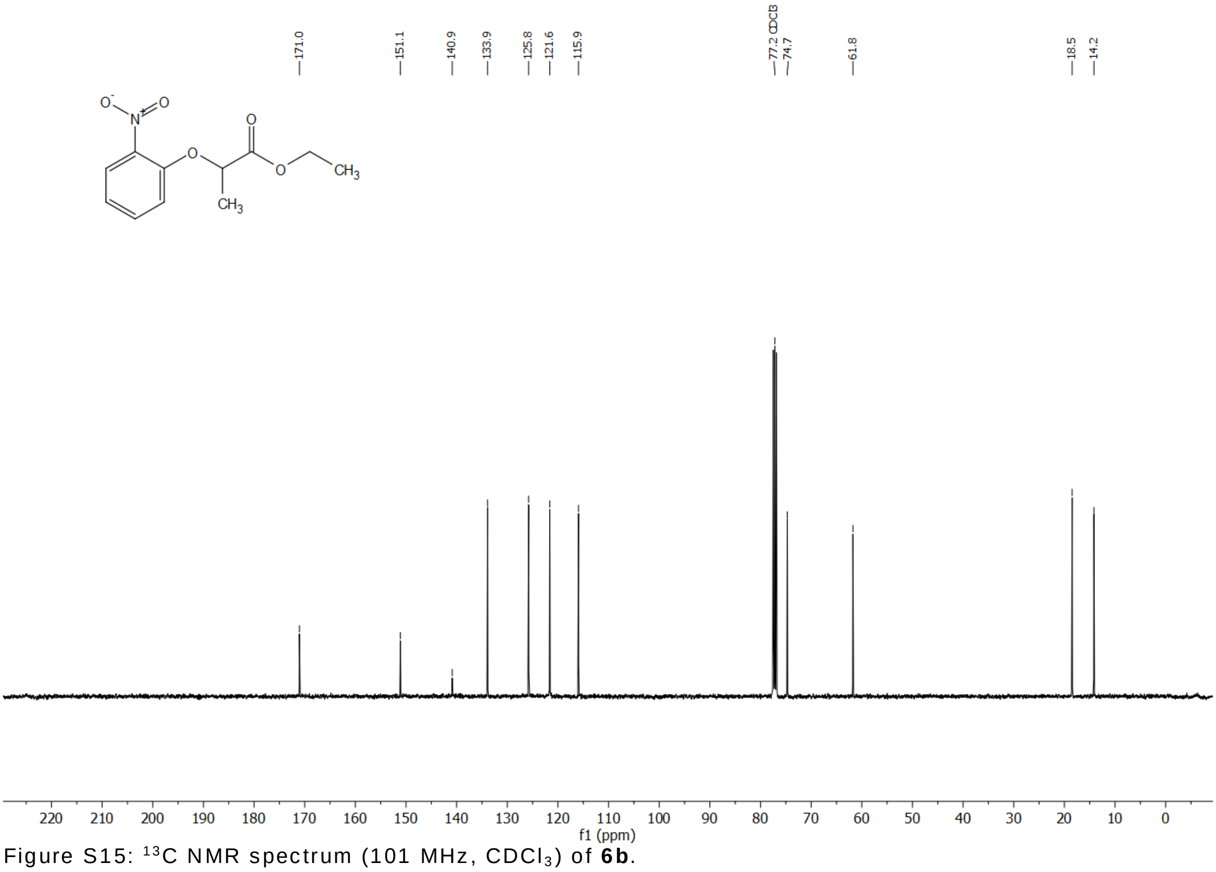

Figure S15: ^{13}C NMR spectrum (101 MHz, $CDCl_3$) of **6b**.

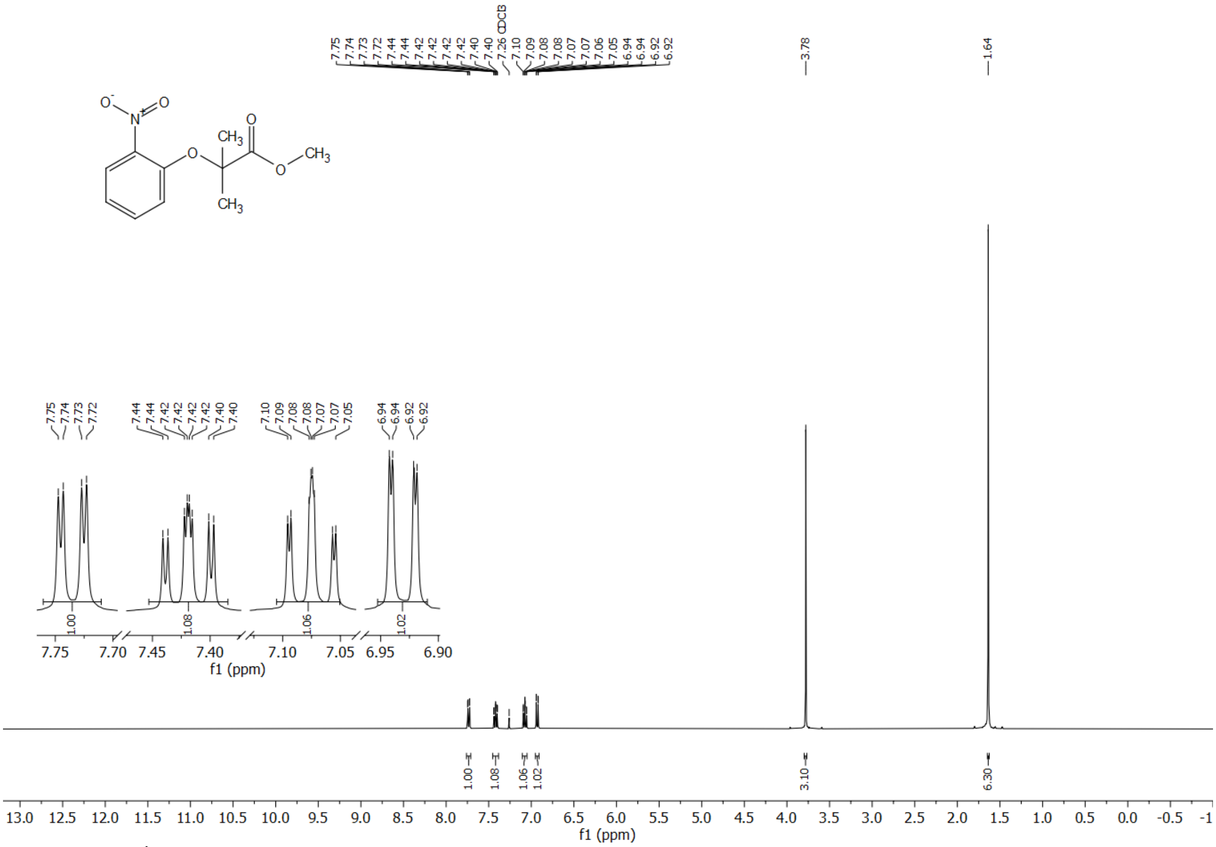

Figure S16: ^{1}H NMR spectrum (400 MHz, $CDCl_3$) of **6c**.

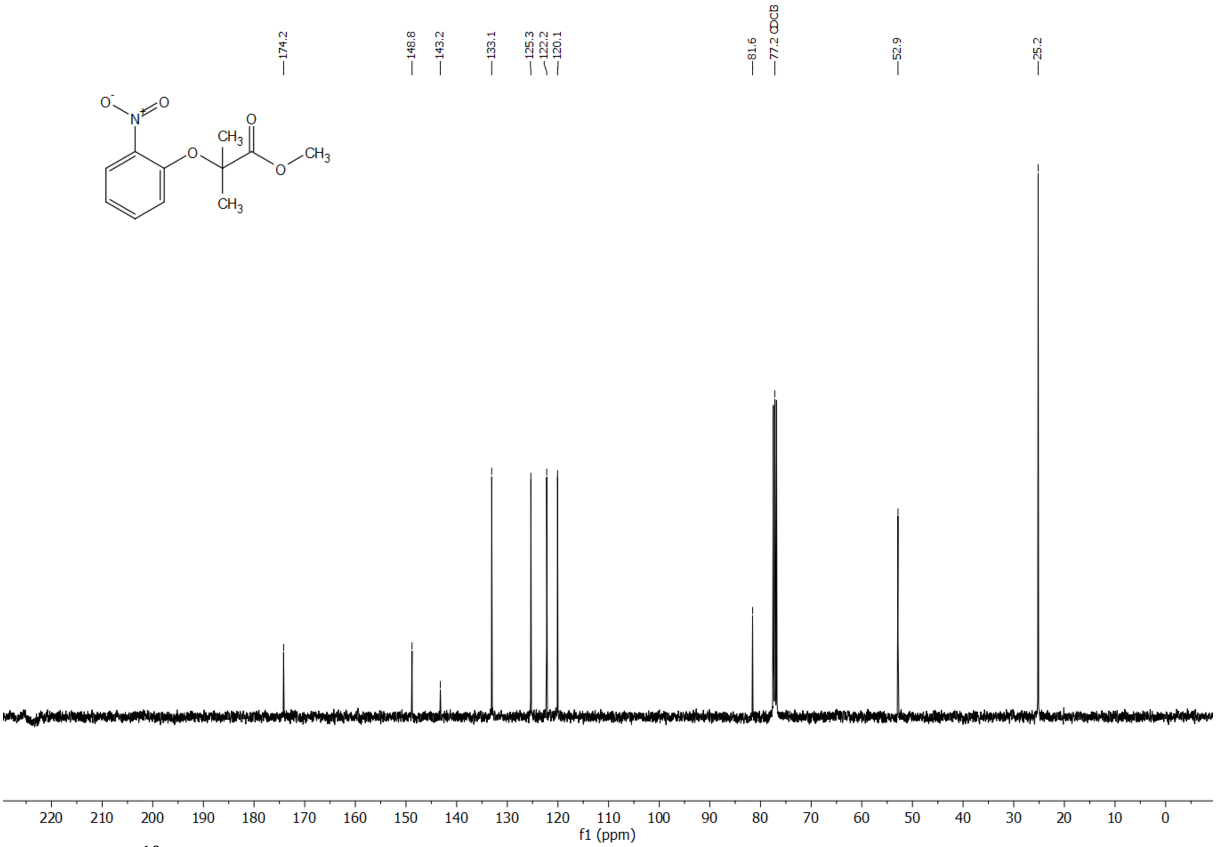

Figure S17: ^{13}C NMR spectrum (101 MHz, $CDCl_3$) of **6c**.

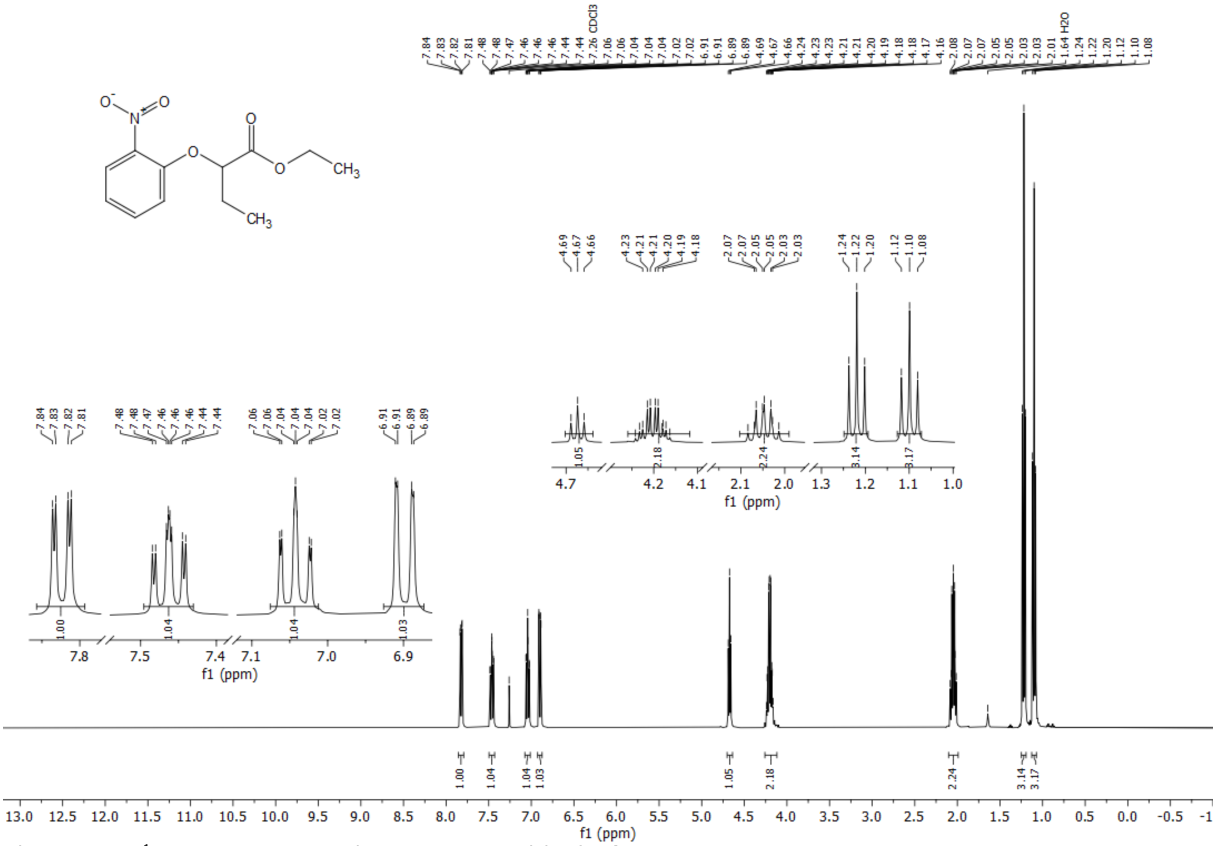

Figure S18: ^{1}H NMR spectrum (400 MHz, $CDCl_3$) of **6d**.

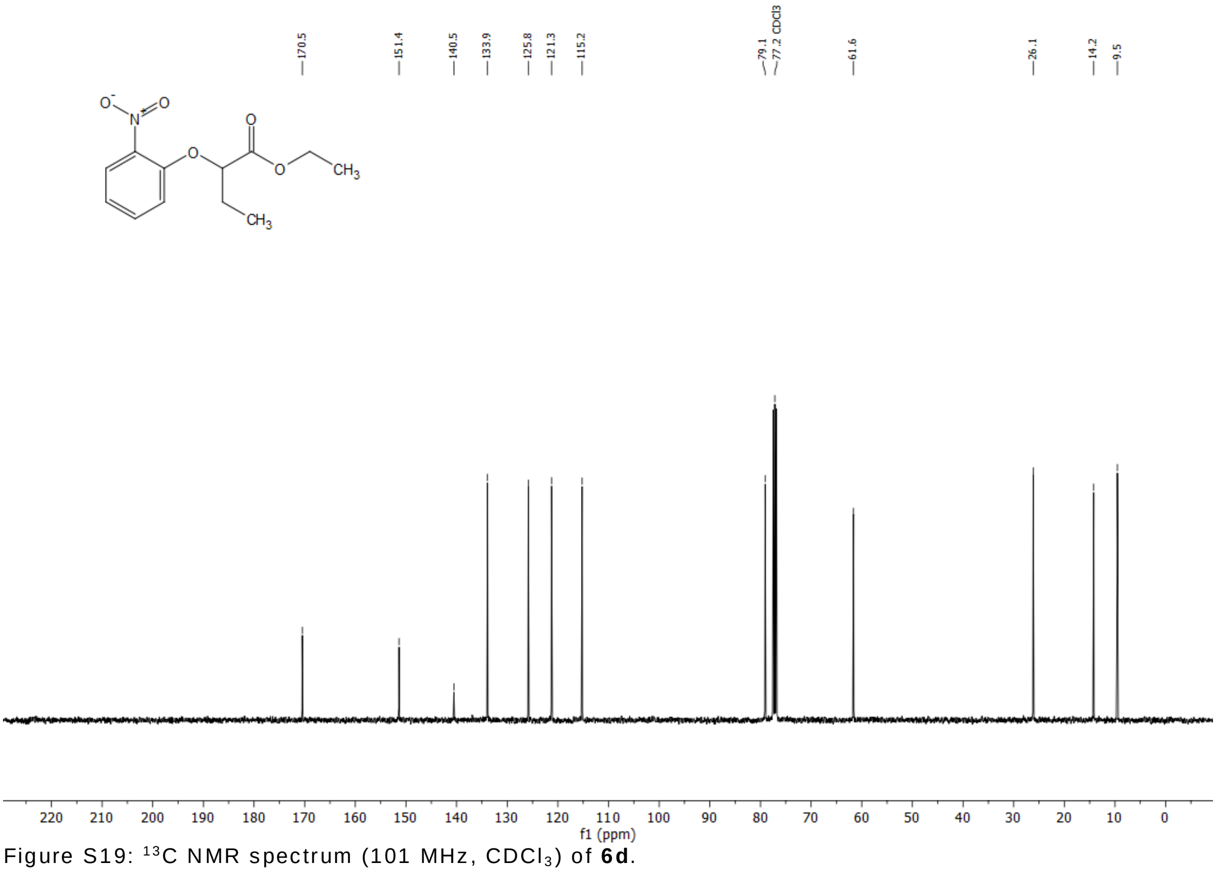

Figure S19: ^{13}C NMR spectrum (101 MHz, $CDCl_3$) of **6d**.

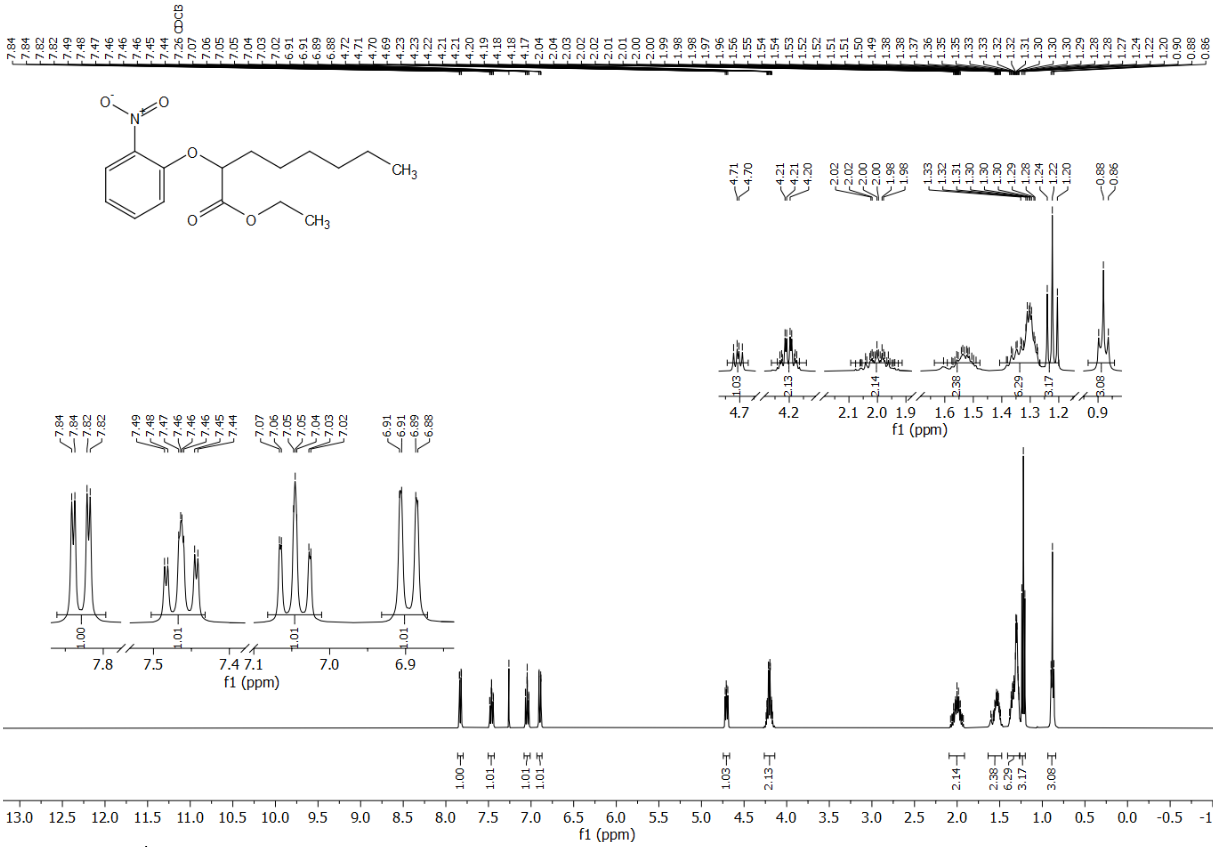

Figure S20: ^{1}H NMR spectrum (400 MHz, $CDCl_3$) of **6e**.

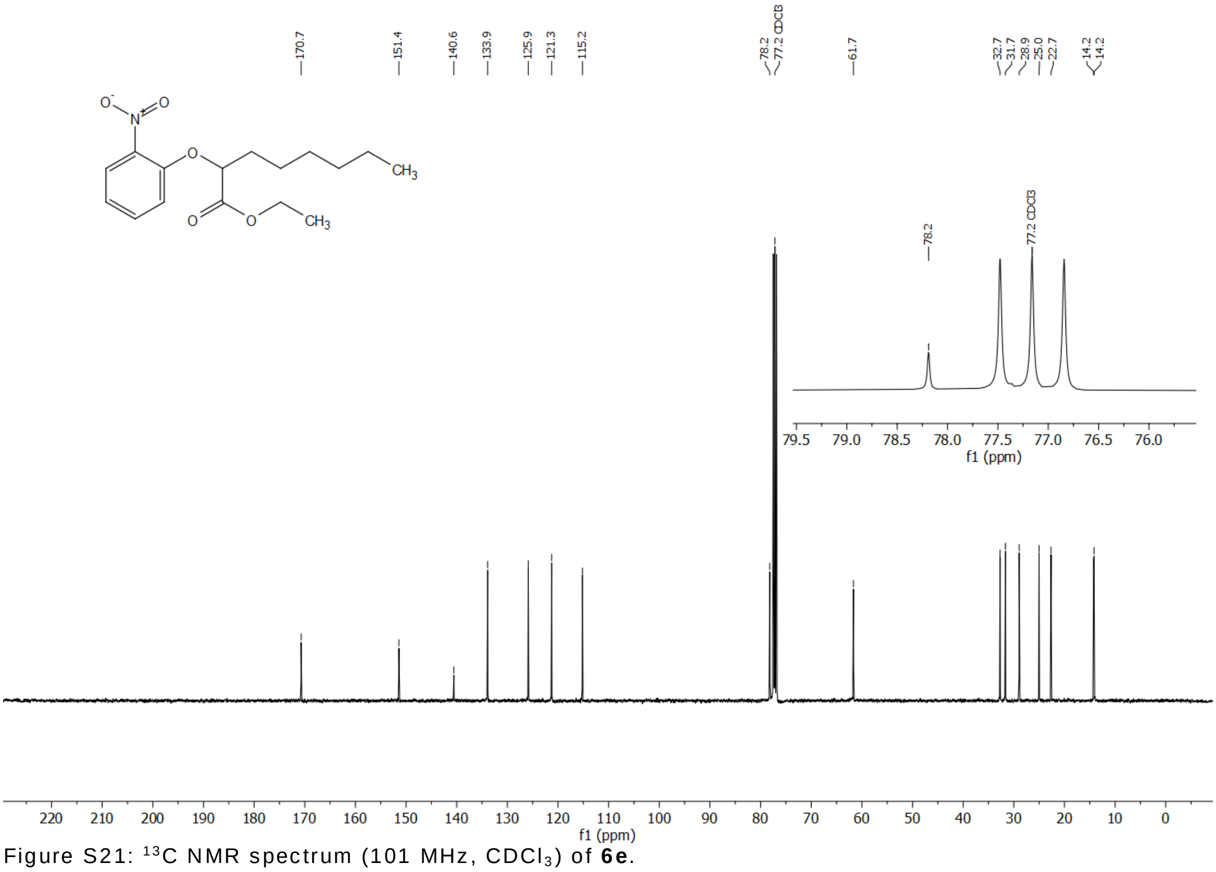

Figure S21: ^{13}C NMR spectrum (101 MHz, $CDCl_3$) of **6e**.

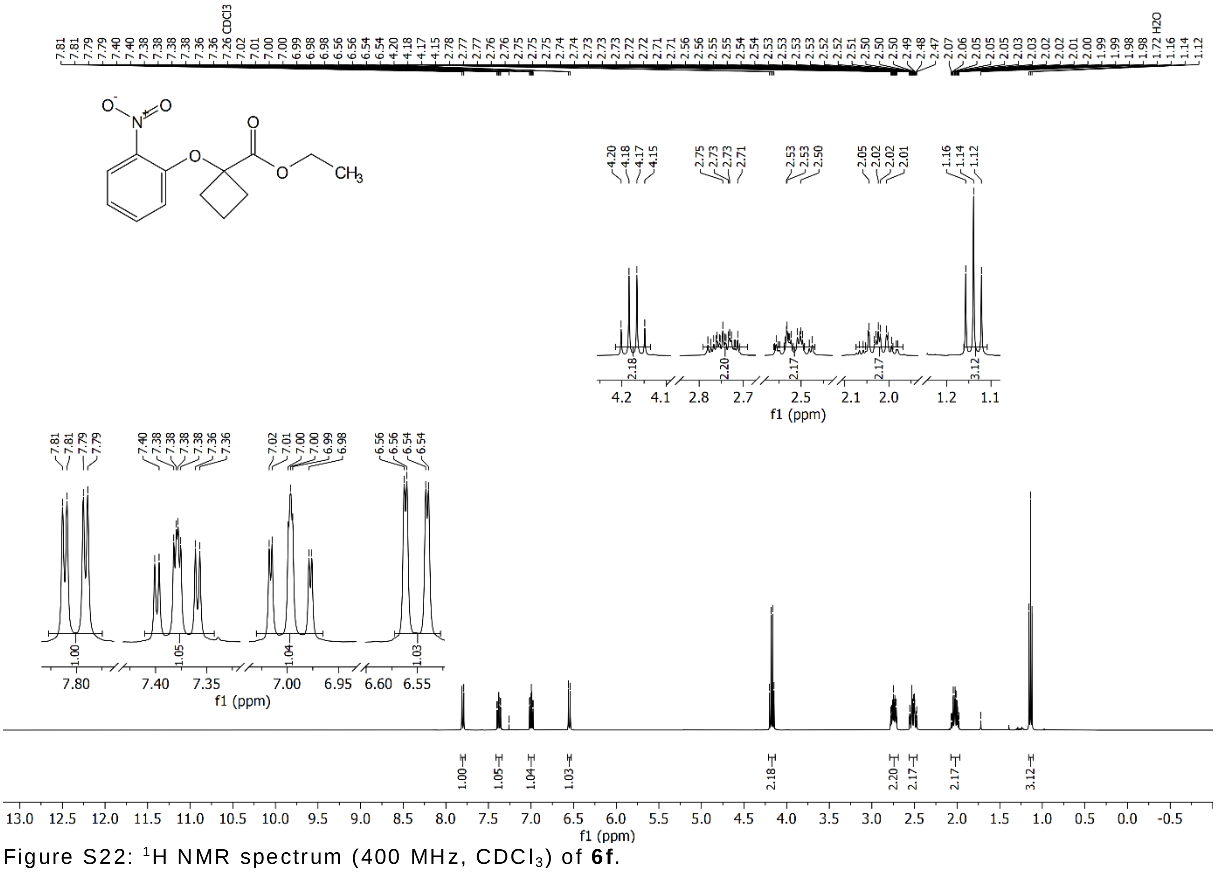

Figure S22: ^{1}H NMR spectrum (400 MHz, $CDCl_3$) of **6f**.

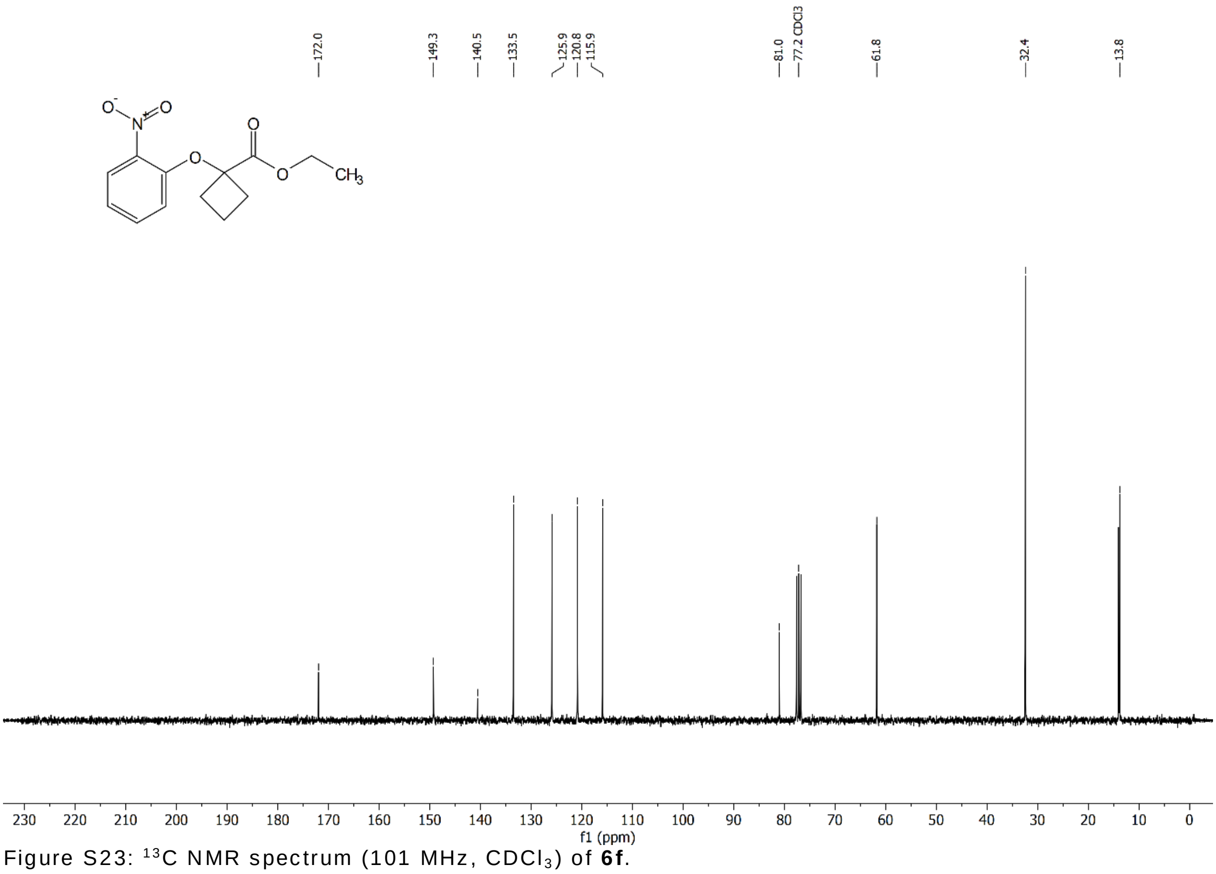

Figure S23: ^{13}C NMR spectrum (101 MHz, $CDCl_3$) of **6f**.

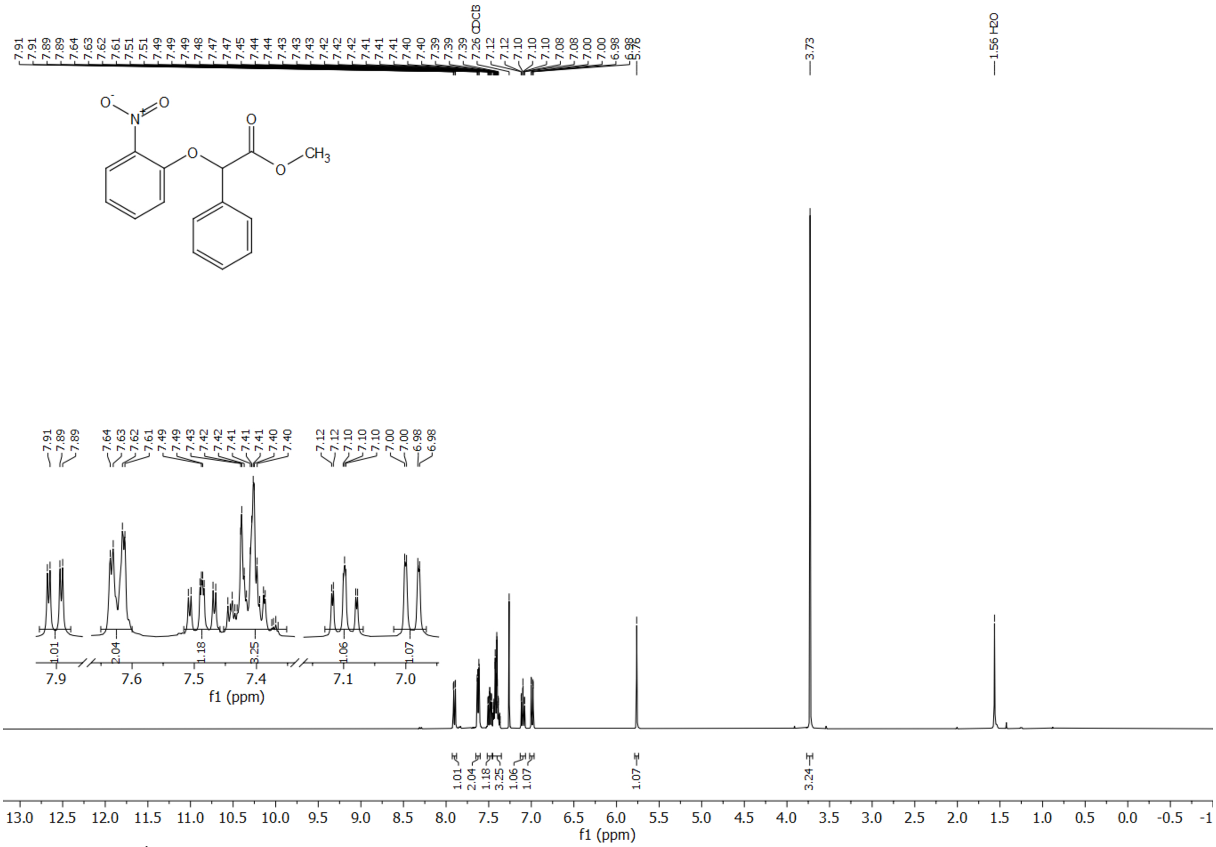

Figure S24: ^{1}H NMR spectrum (400 MHz, $CDCl_3$) of **6h**.

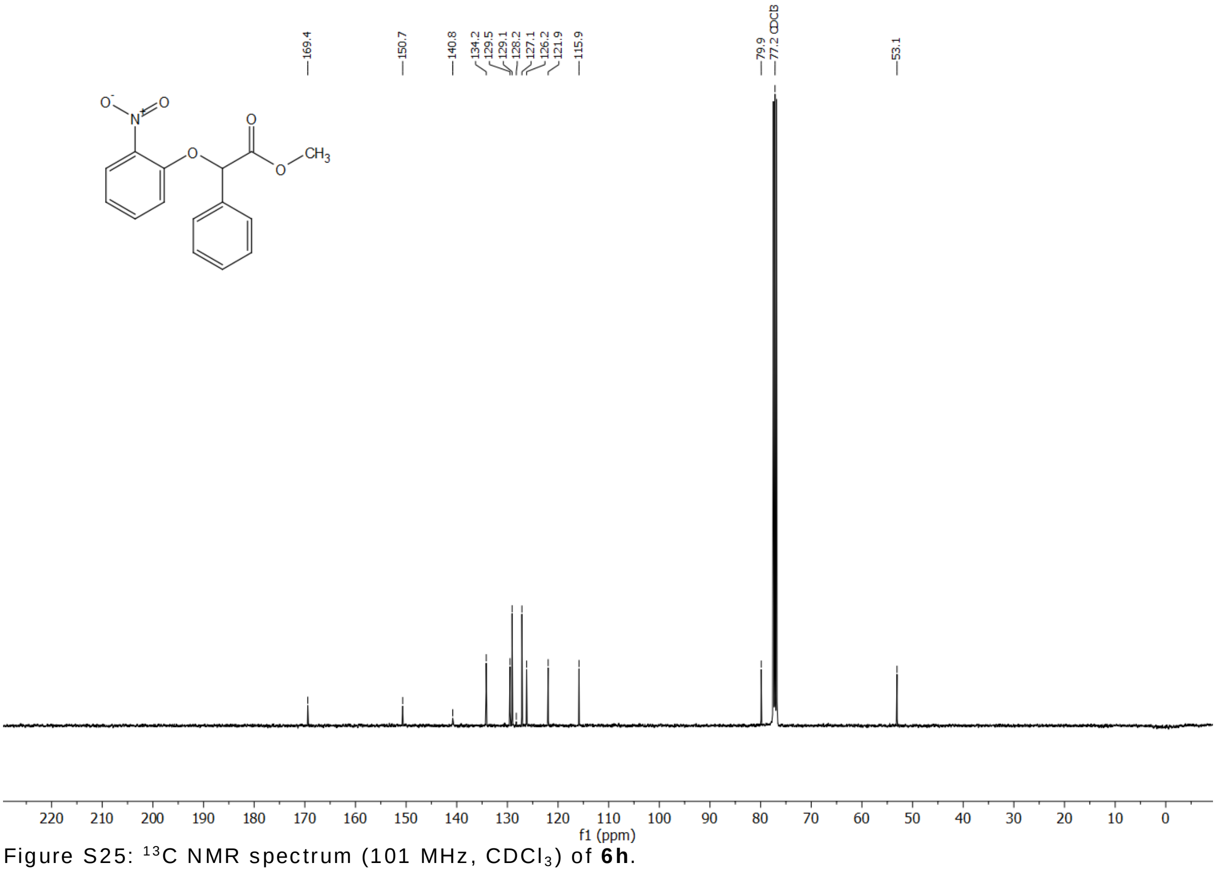

Figure S25: ^{13}C NMR spectrum (101 MHz, $CDCl_3$) of **6h**.

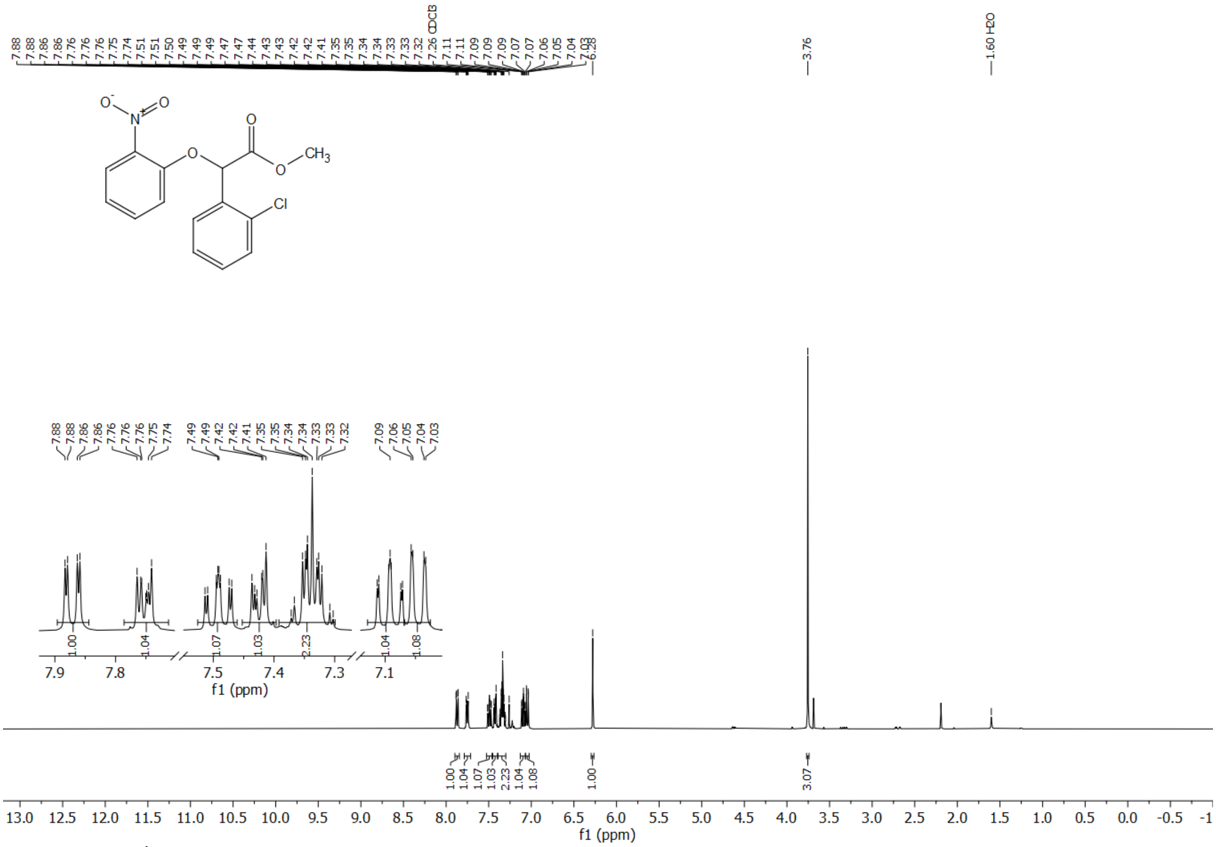

Figure S26: ^{1}H NMR spectrum (400 MHz, $CDCl_3$) of **6i**.

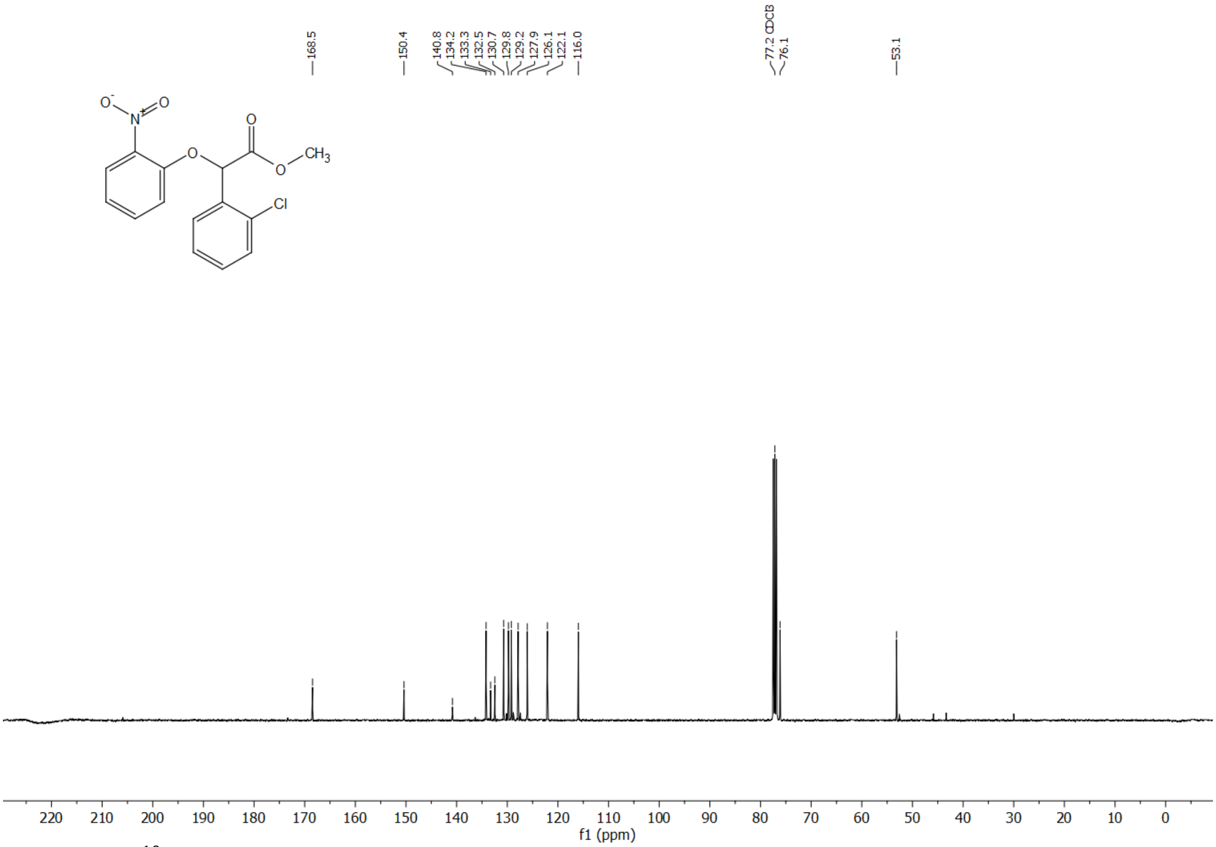

Figure S27: ^{13}C NMR spectrum (101 MHz, $CDCl_3$) of **6i**.

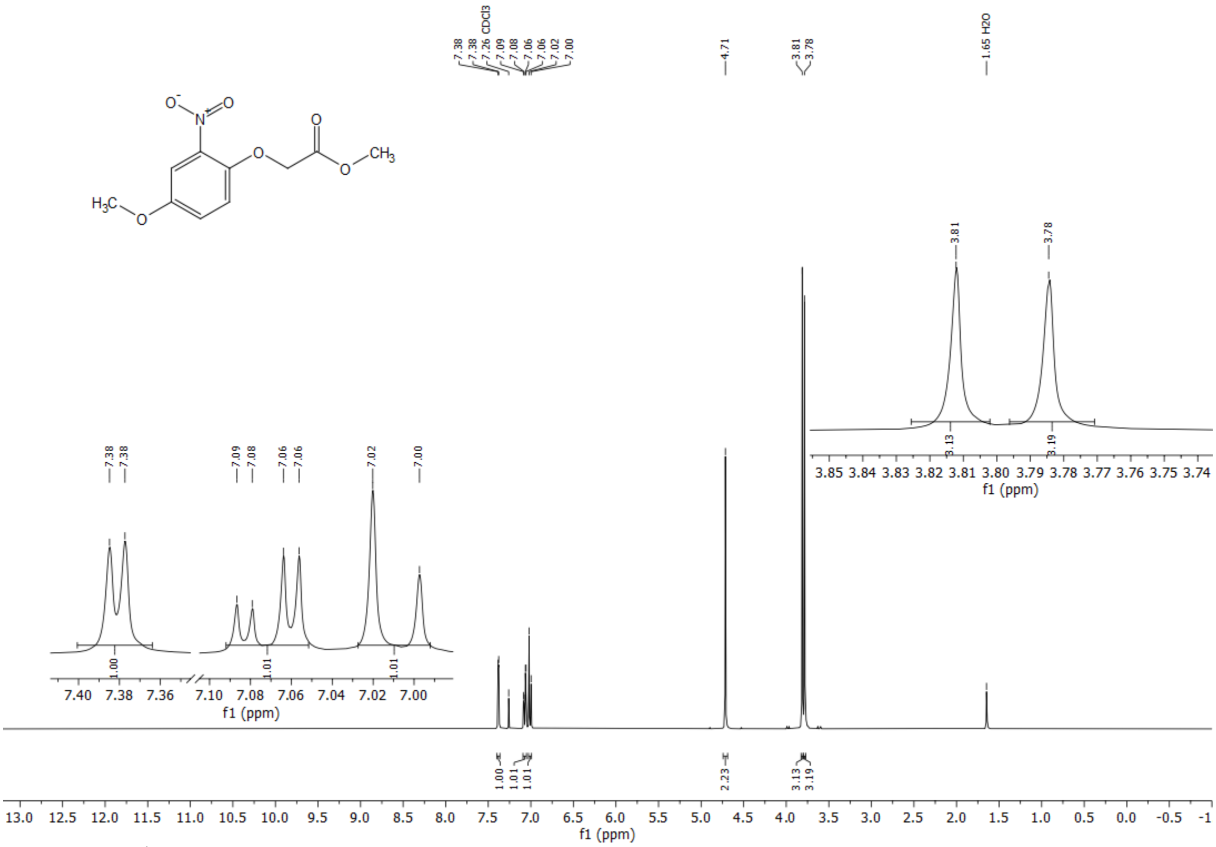

Figure S28: ^{1}H NMR spectrum (400 MHz, $CDCl_3$) of **6j**.

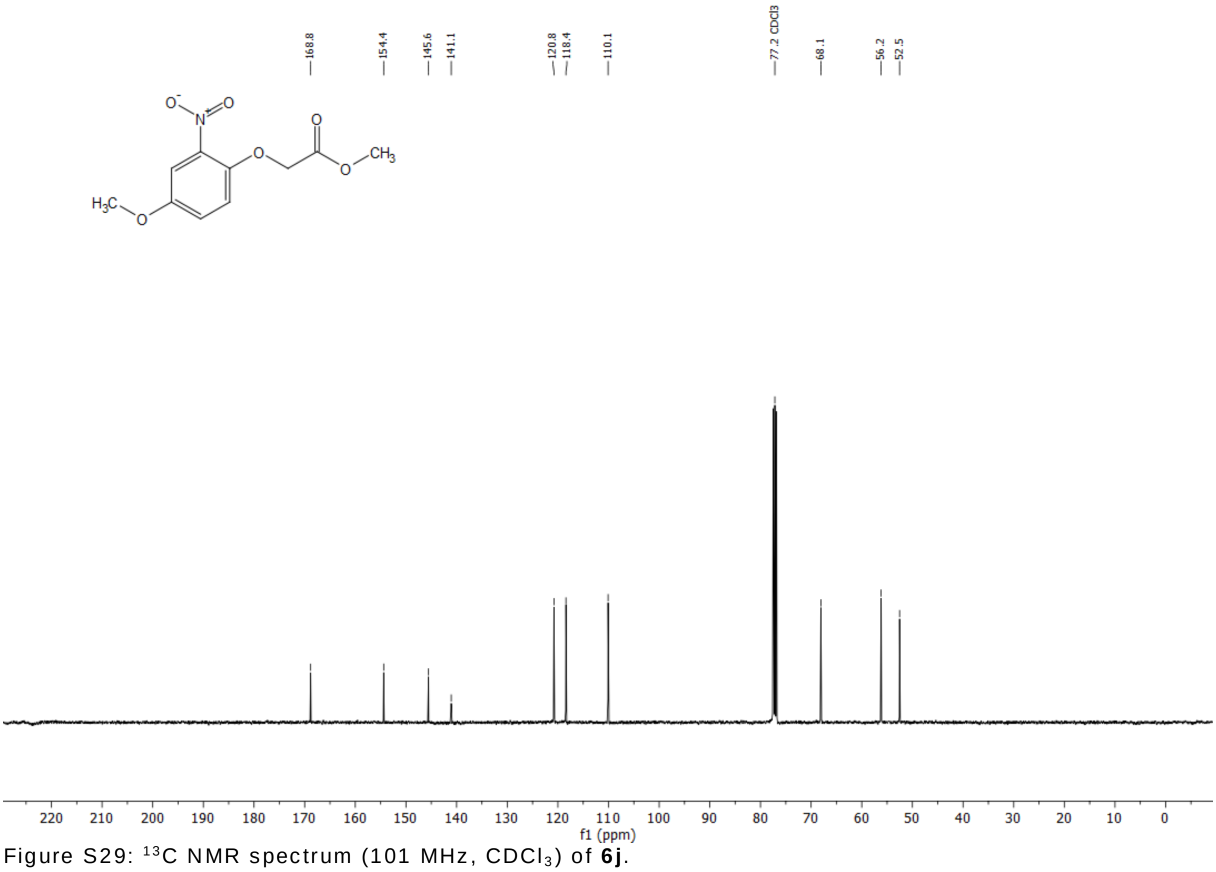

Figure S29: ^{13}C NMR spectrum (101 MHz, $CDCl_3$) of **6j**.

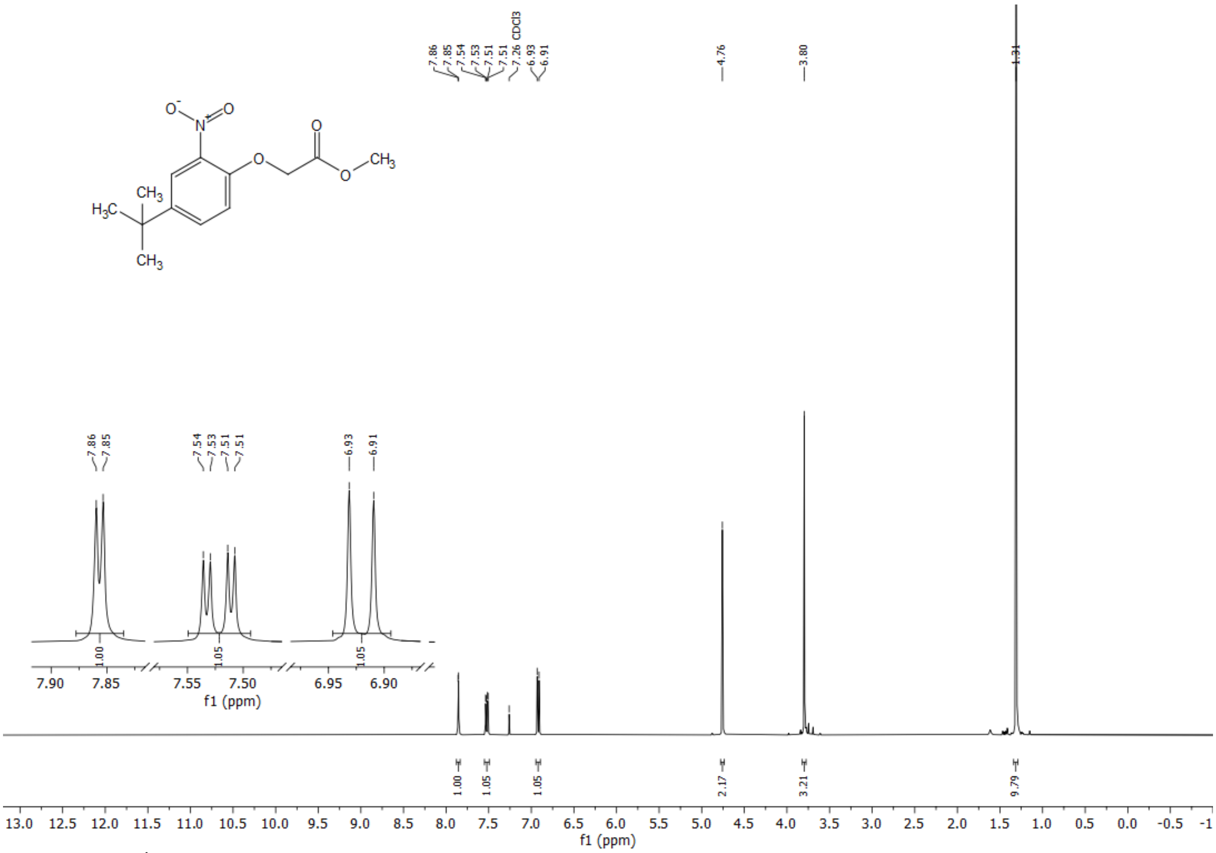

Figure S30: ^{1}H NMR spectrum (400 MHz, $CDCl_3$) of **6k**.

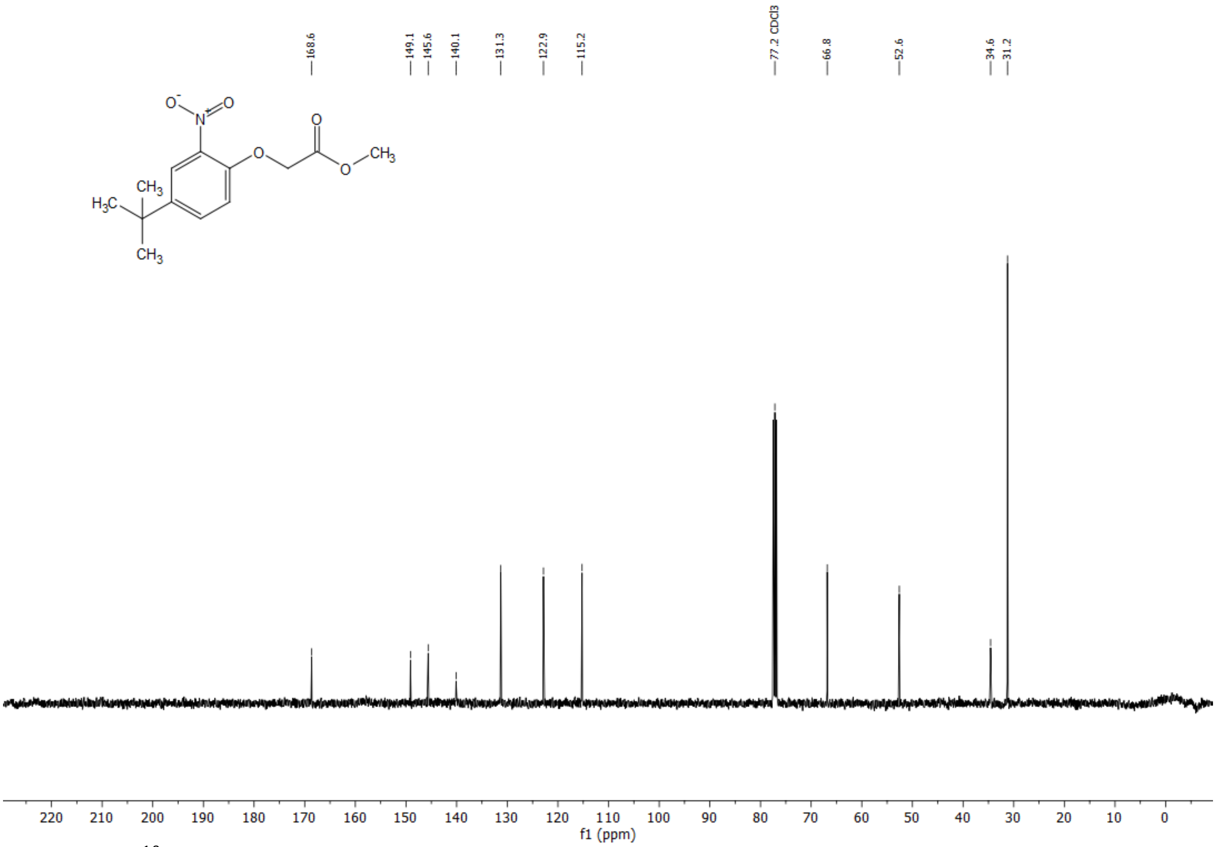

Figure S31: ^{13}C NMR spectrum (101 MHz, $CDCl_3$) of **6k**.

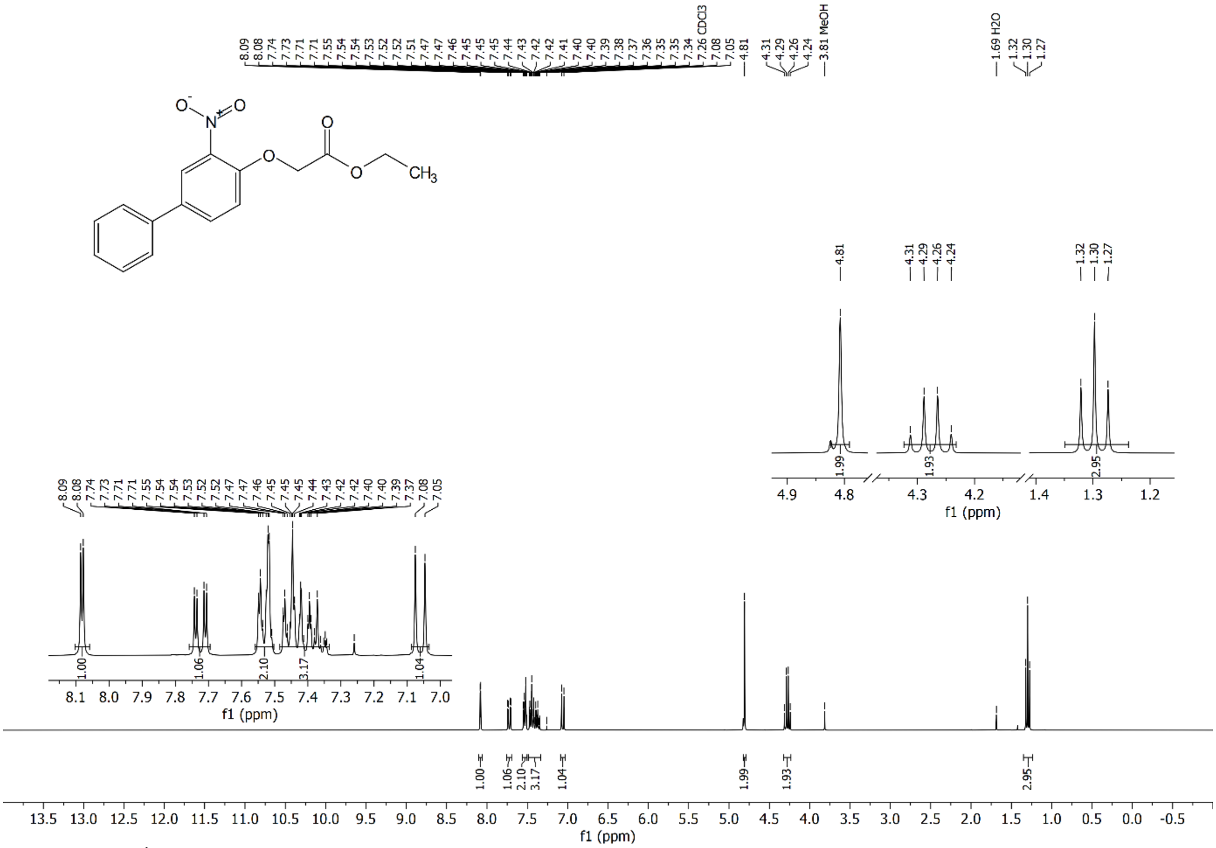

Figure S32: ^{1}H NMR spectrum (300 MHz, $CDCl_3$) of **6l**.

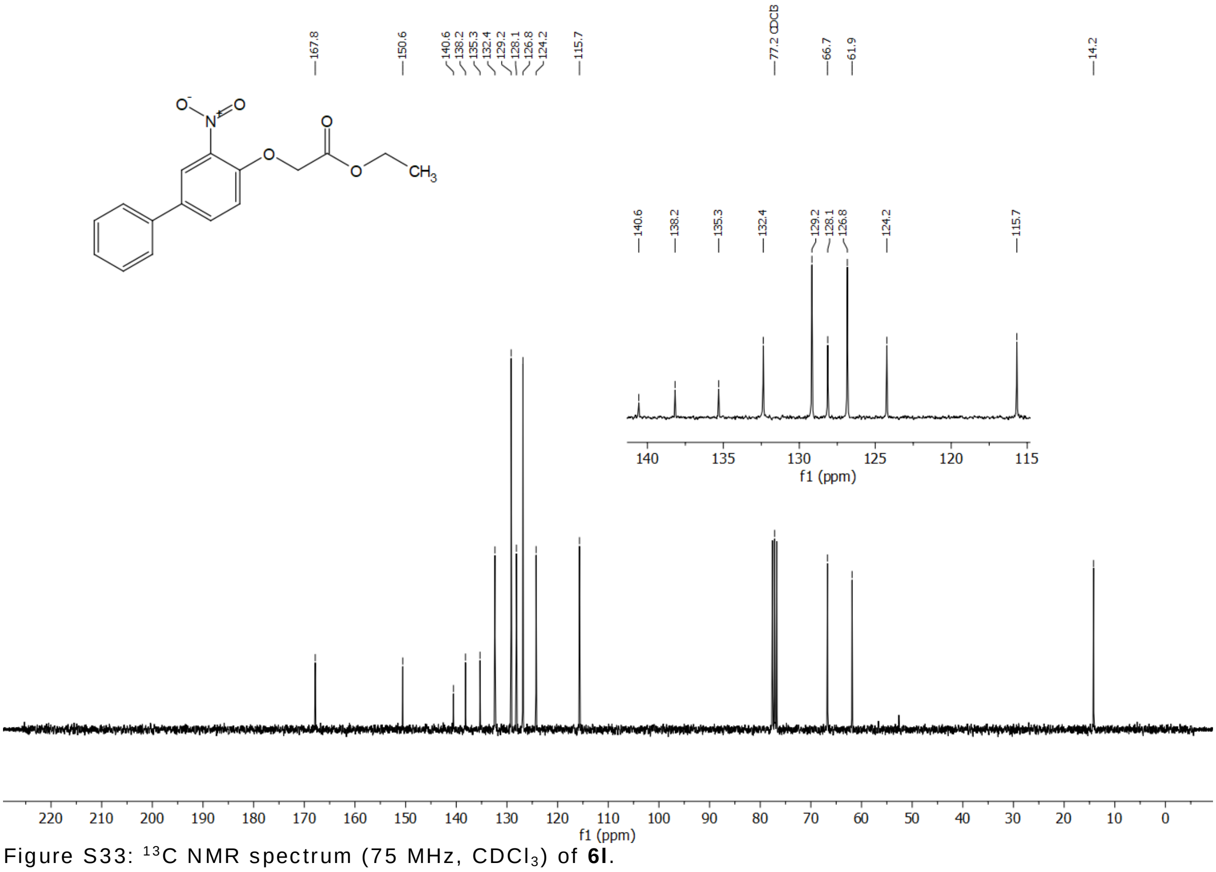

Figure S33: ^{13}C NMR spectrum (75 MHz, $CDCl_3$) of **6l**.

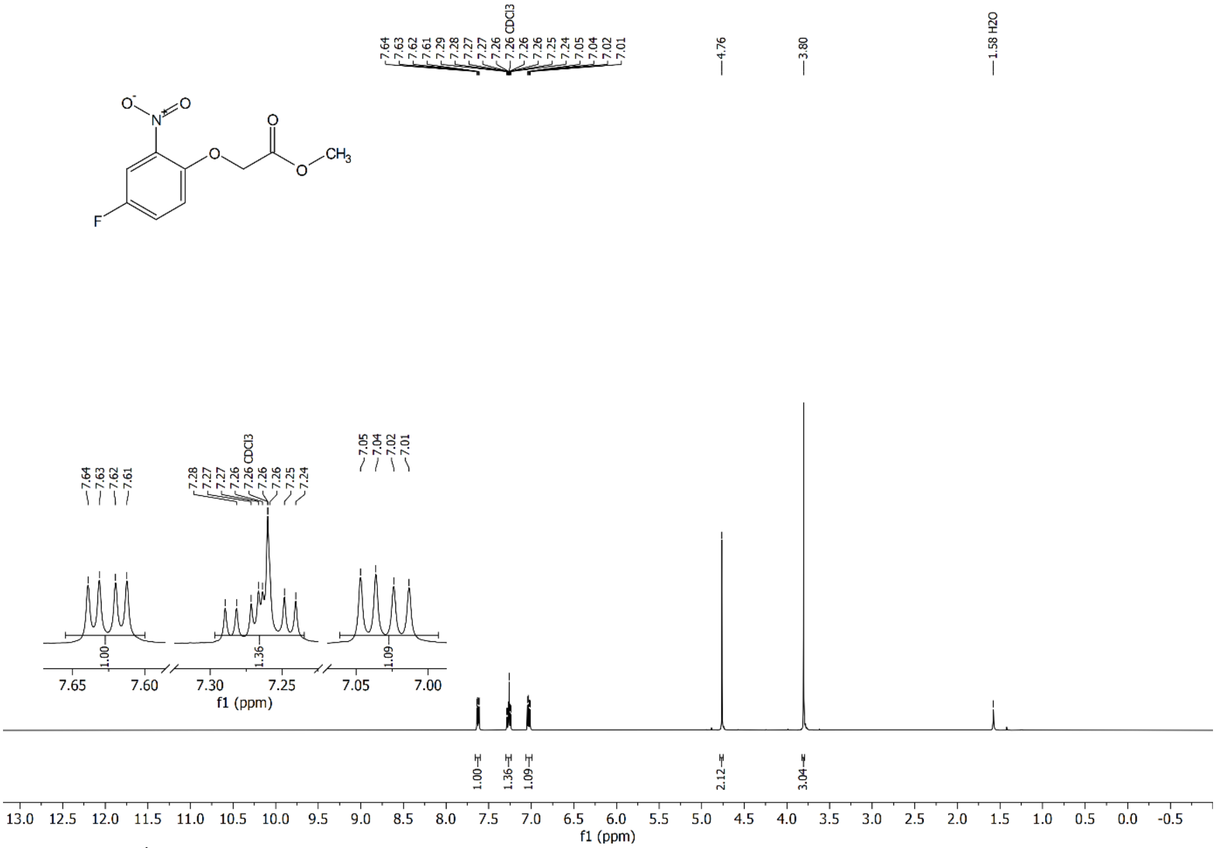

Figure S34: ^{1}H NMR spectrum (400 MHz, $CDCl_3$) of **6m**.

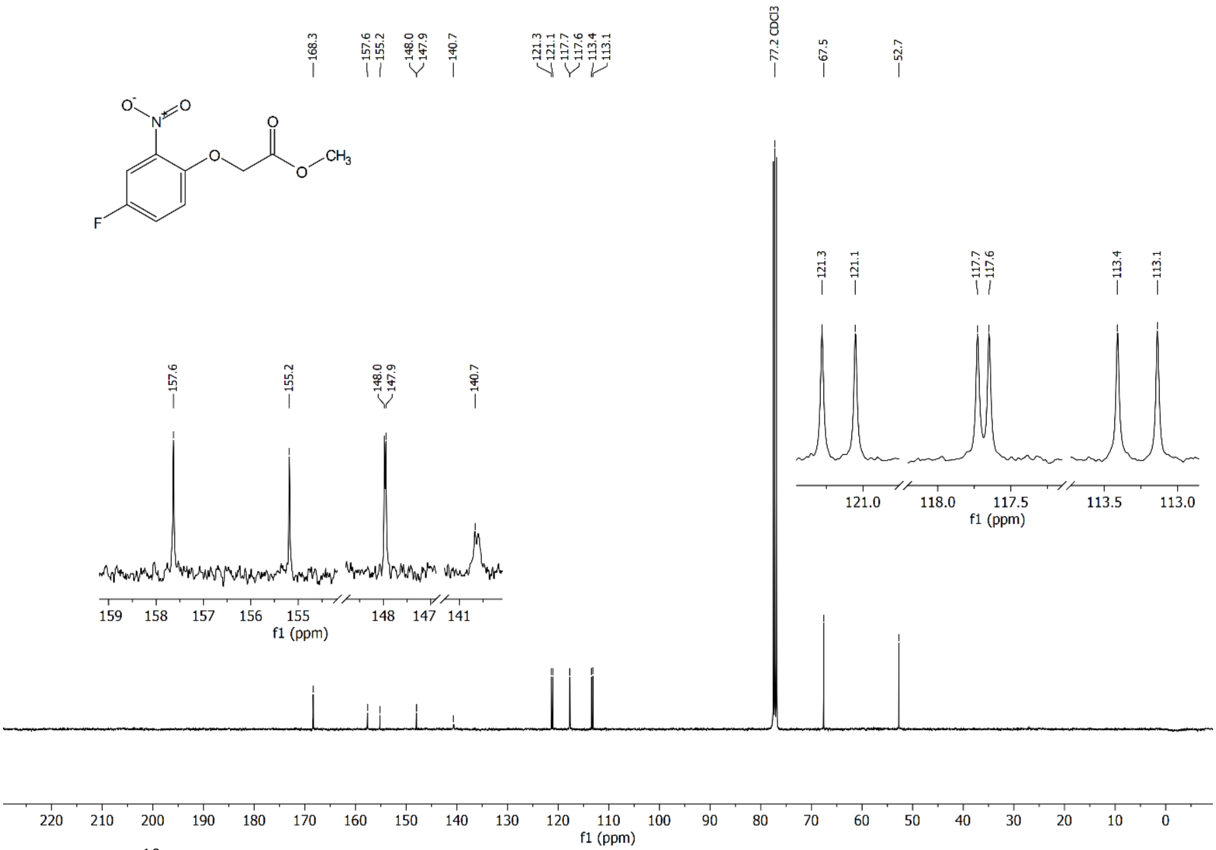

Figure S35: ^{13}C NMR spectrum (101 MHz, $CDCl_3$) of **6m**.

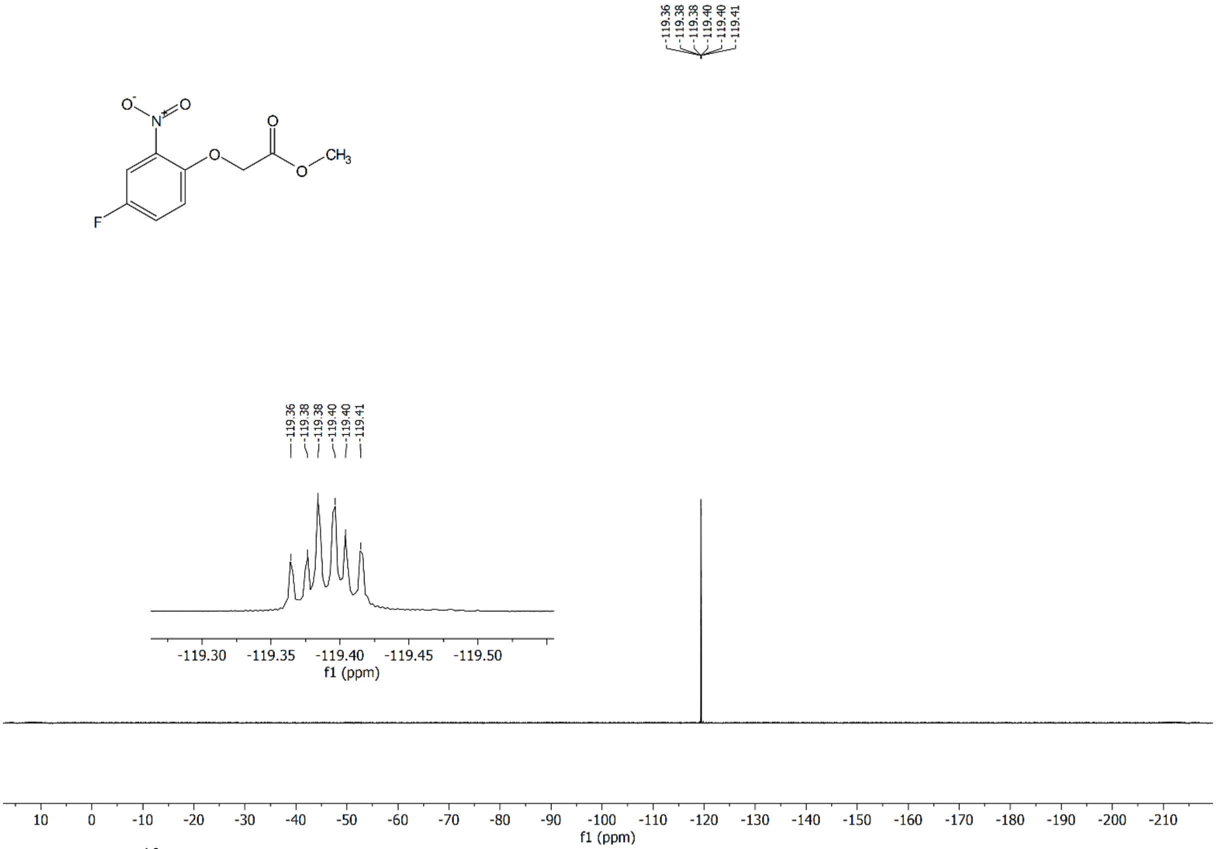

Figure S36: ^{19}F NMR spectrum (376 MHz, $CDCl_3$) of **6m**.

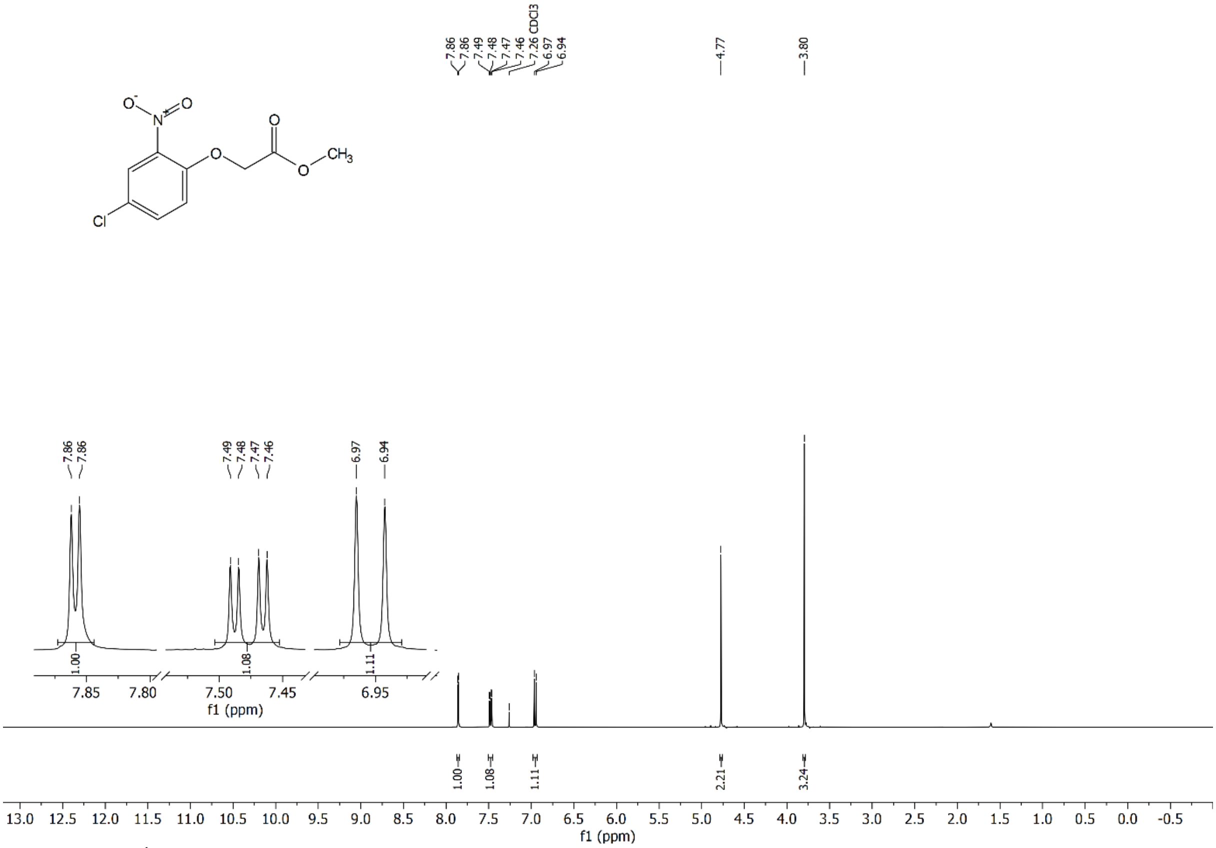

Figure S37: ^{1}H NMR spectrum (400 MHz, $CDCl_3$) of **6n**.

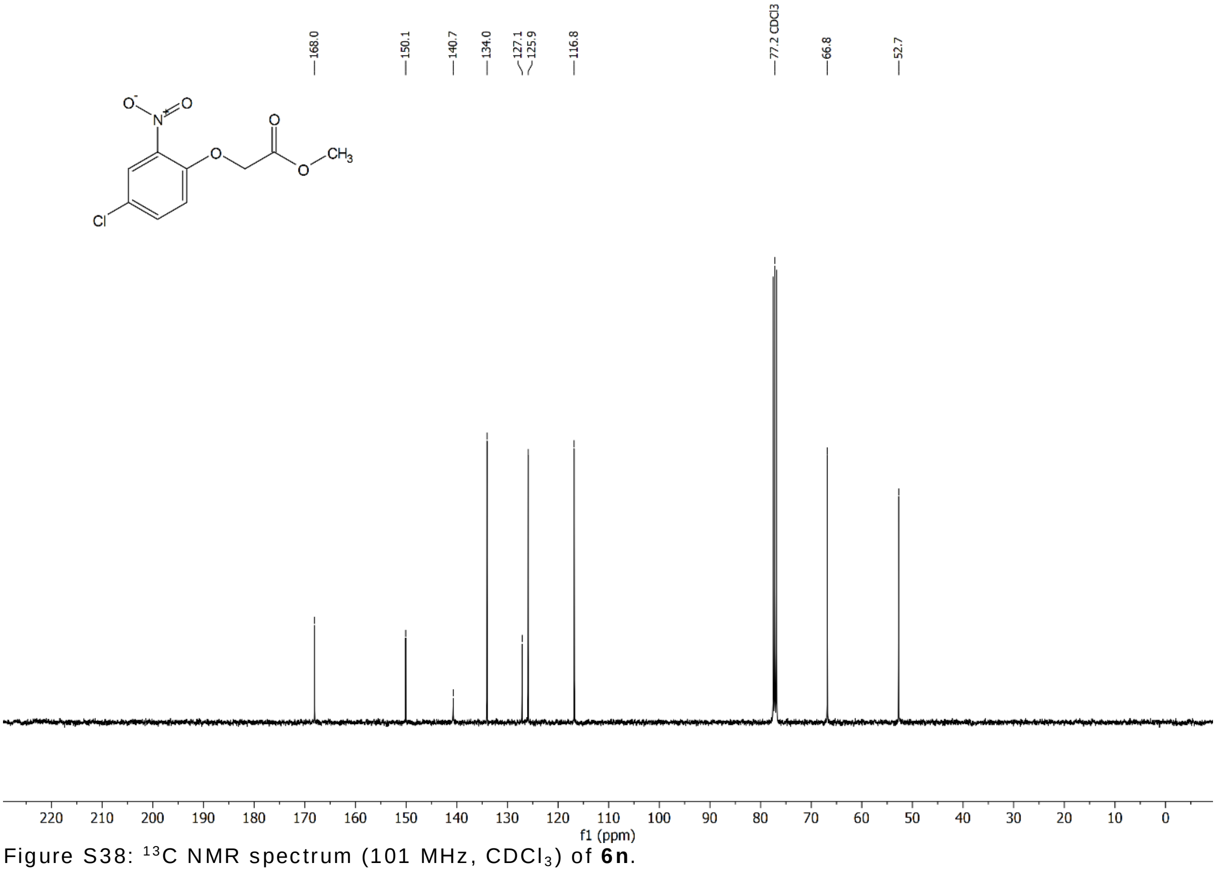

Figure S38: ^{13}C NMR spectrum (101 MHz, $CDCl_3$) of **6n**.

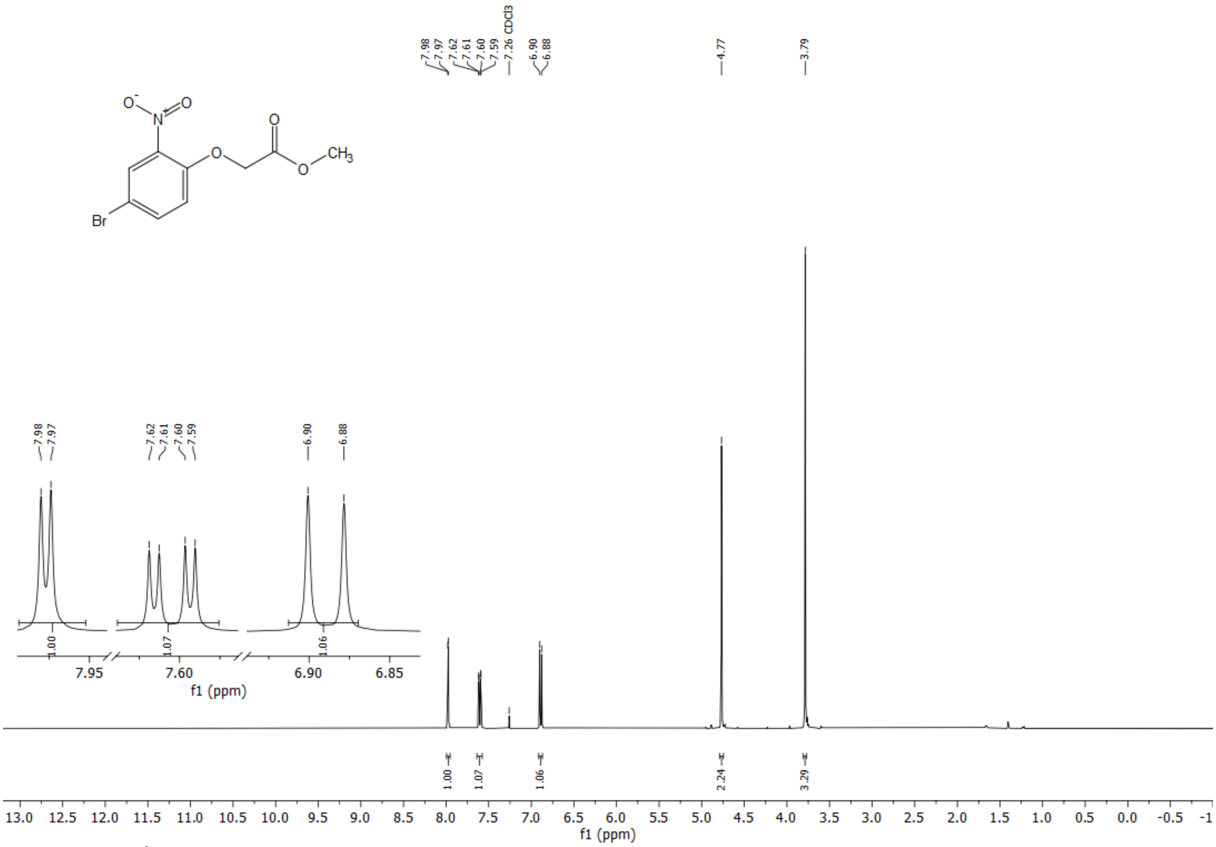

Figure S39: ^{1}H NMR spectrum (400 MHz, $CDCl_3$) of **6p**.

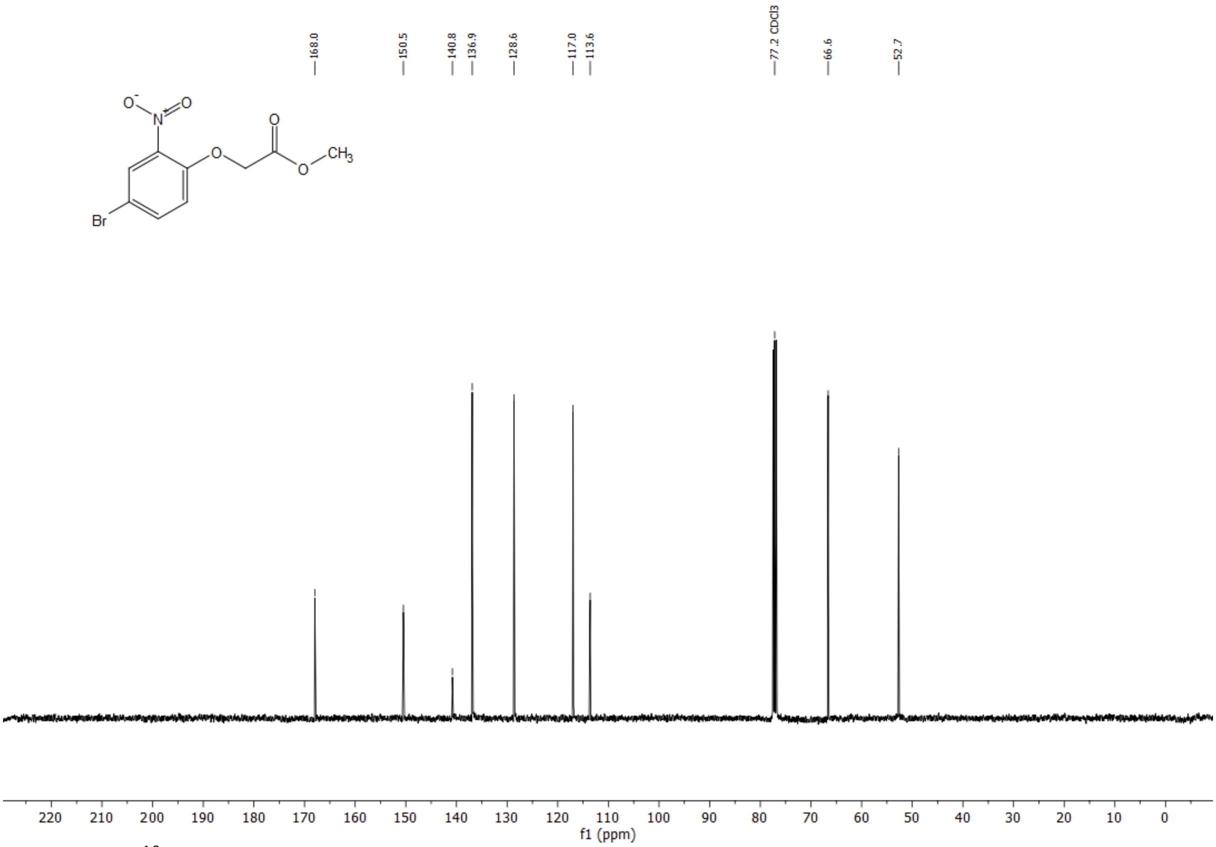

Figure S40: ^{13}C NMR spectrum (101 MHz, $CDCl_3$) of **6p**.

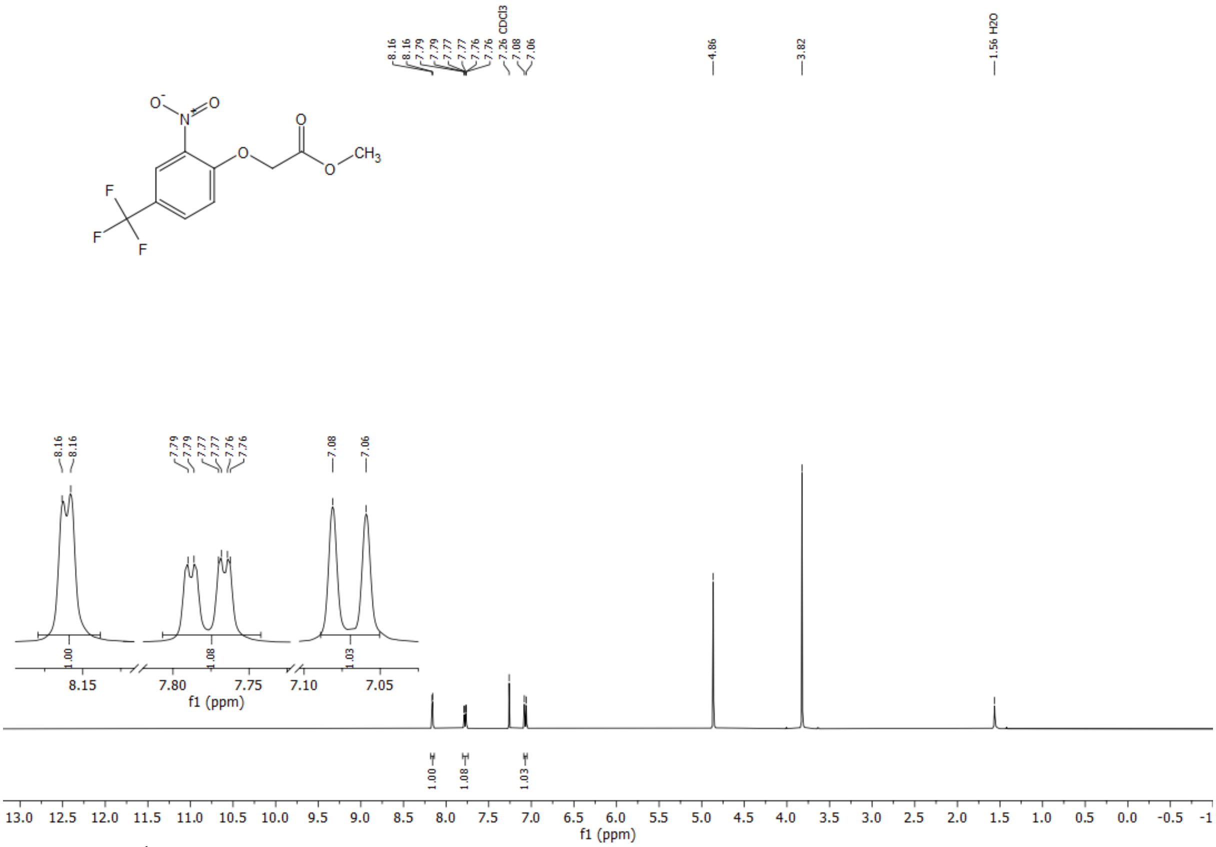

Figure S41: ^{1}H NMR spectrum (400 MHz, $CDCl_3$) of **6q**.

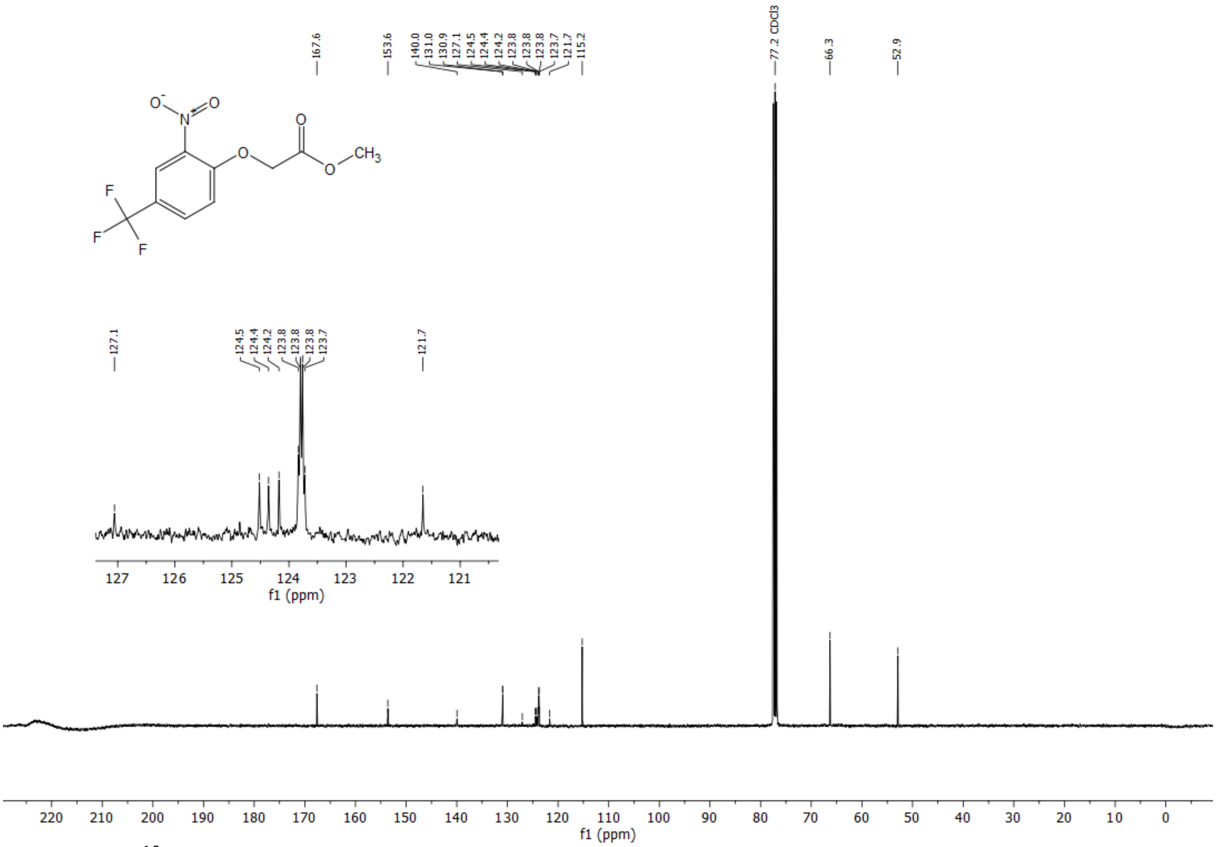

Figure S42: ^{13}C NMR spectrum (101 MHz, $CDCl_3$) of **6q**.

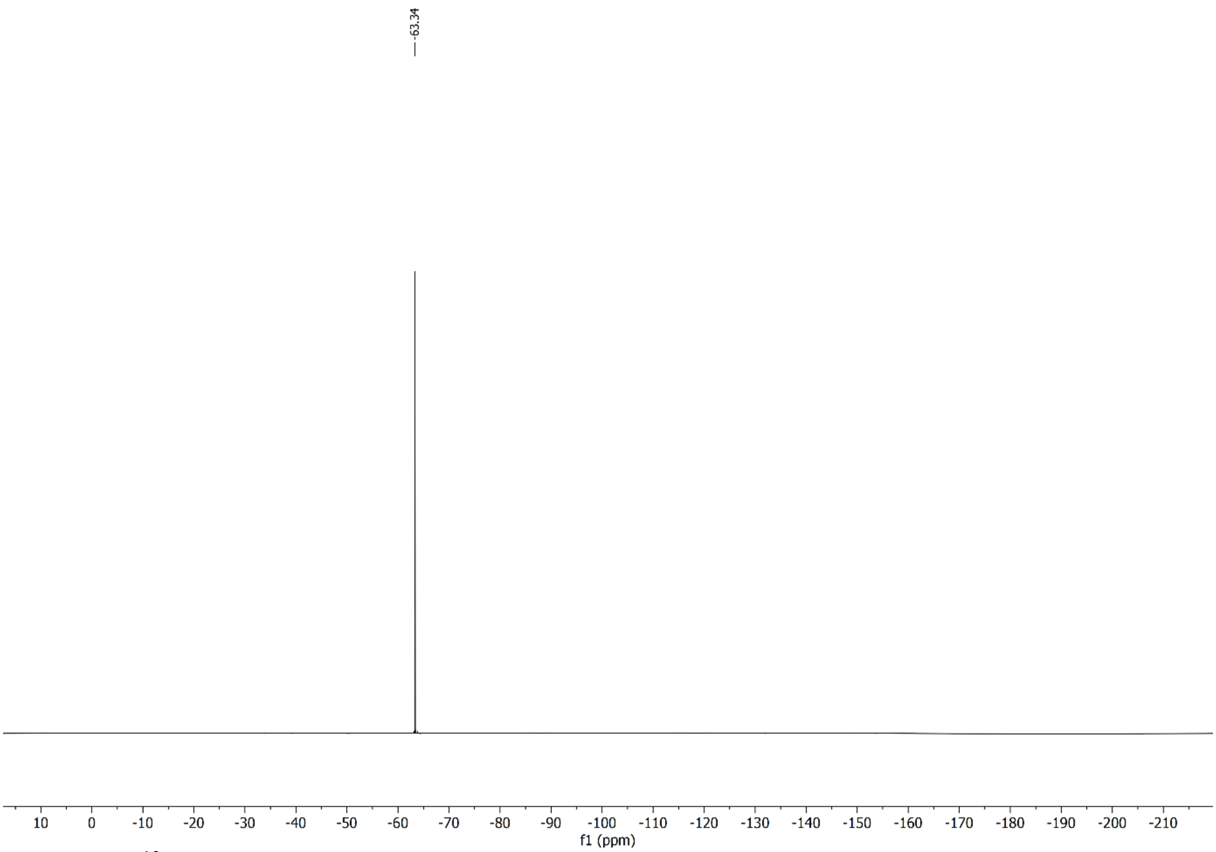

Figure S43: ^{19}F NMR spectrum (376 MHz, $CDCl_3$) of **6q**.

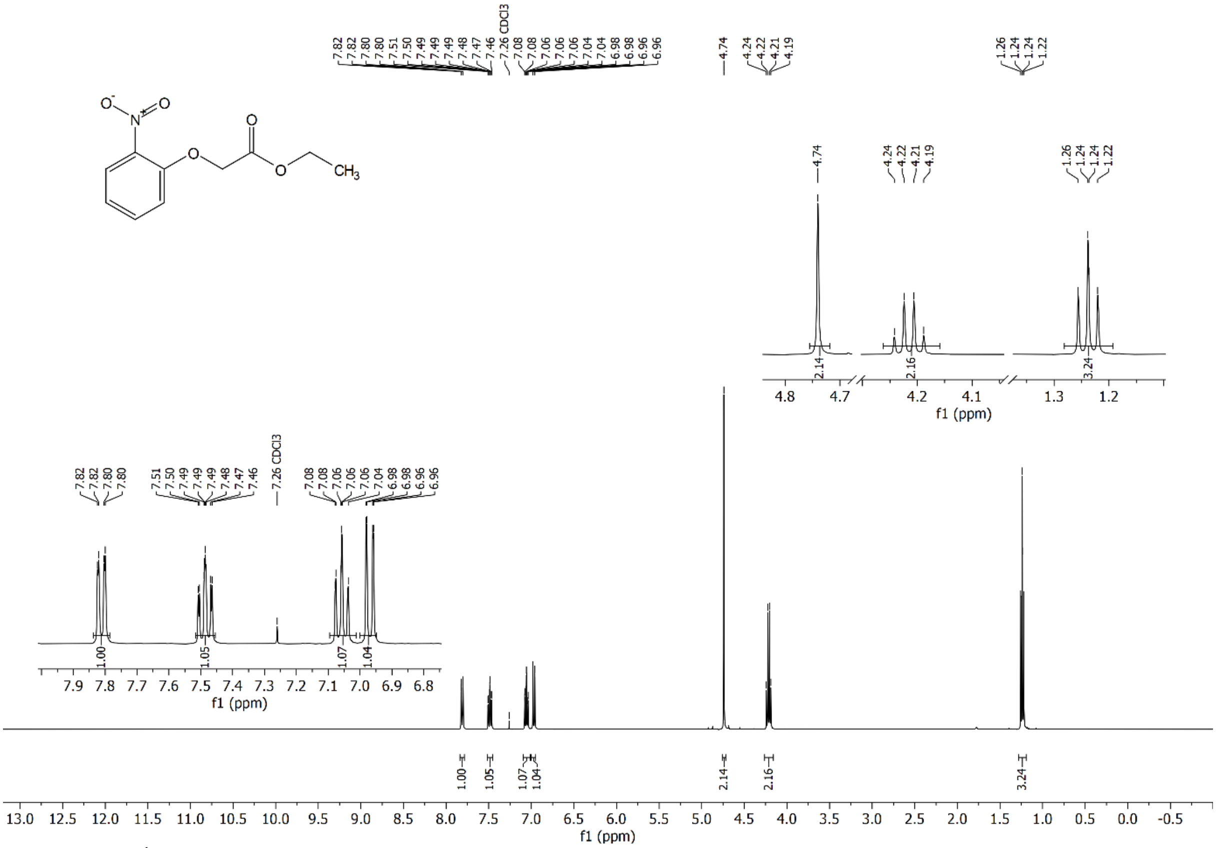

Figure S44: ^{1}H NMR spectrum (400 MHz, $CDCl_3$) of **6s**.

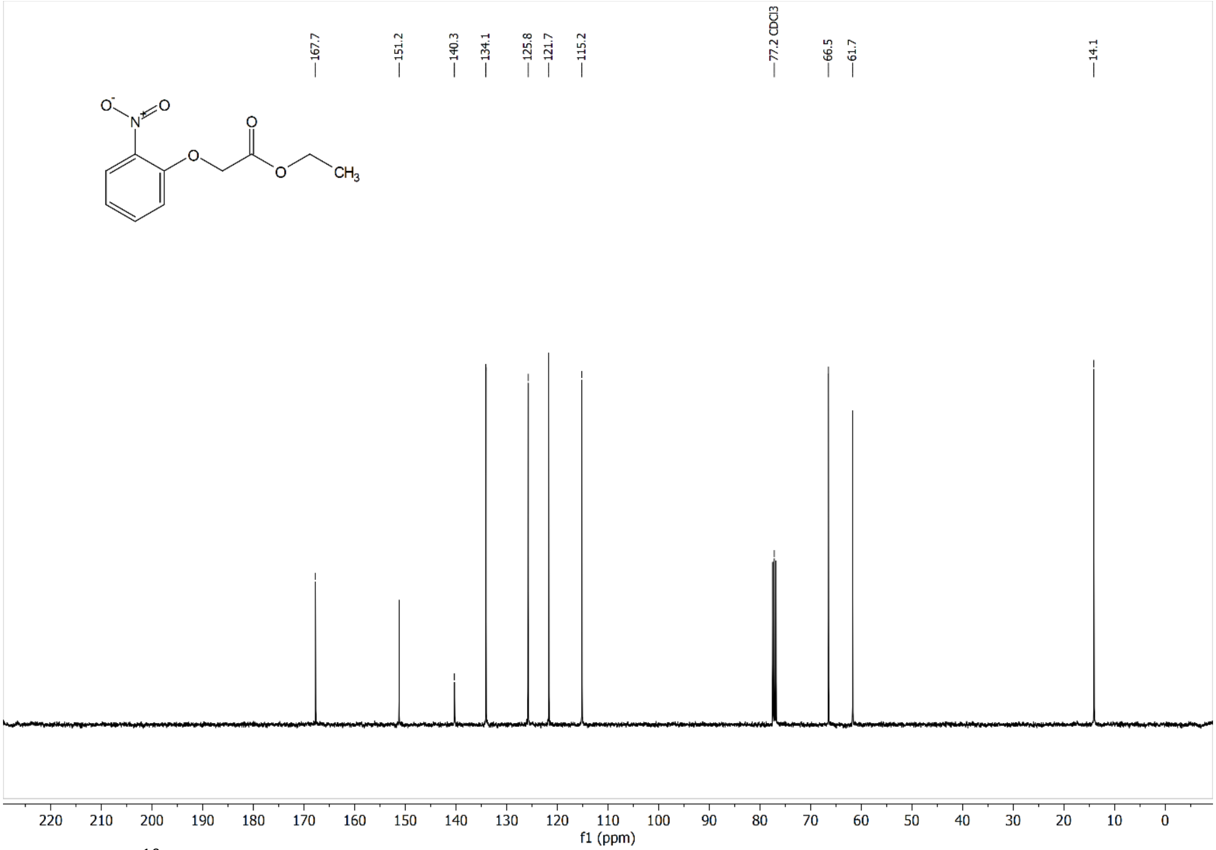

Figure S45: ^{13}C NMR spectrum (101 MHz, $CDCl_3$) of **6s**.

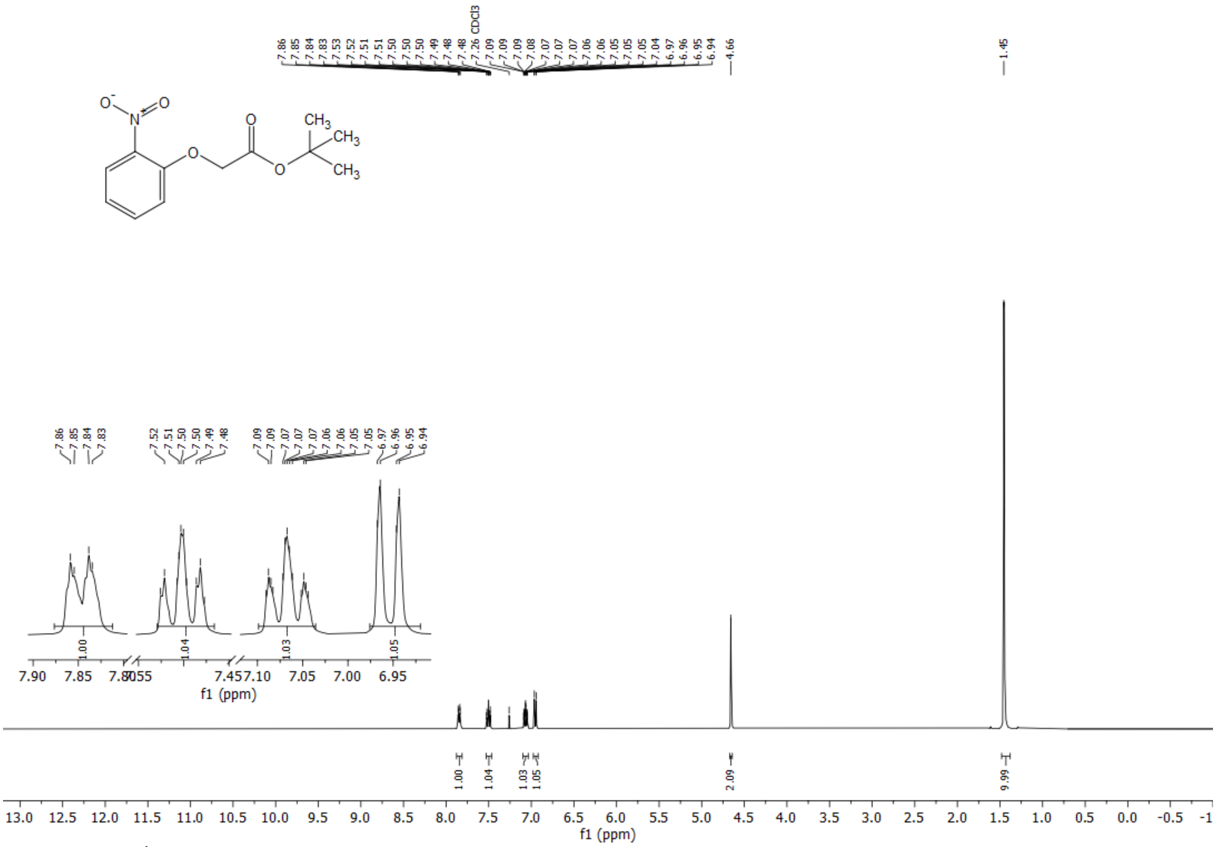

Figure S46: ^{1}H NMR spectrum (400 MHz, $CDCl_3$) of **6t**.

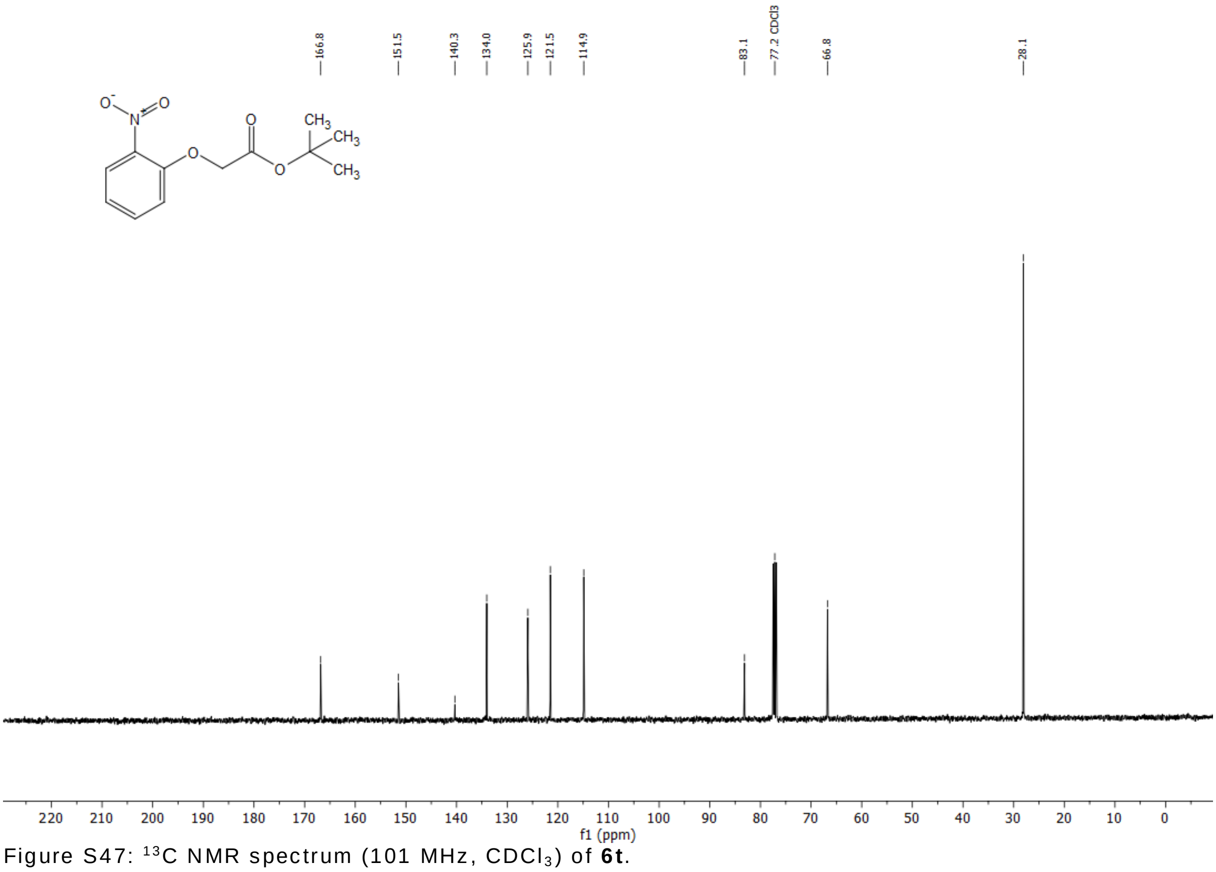

Figure S47: ^{13}C NMR spectrum (101 MHz, $CDCl_3$) of **6t**.

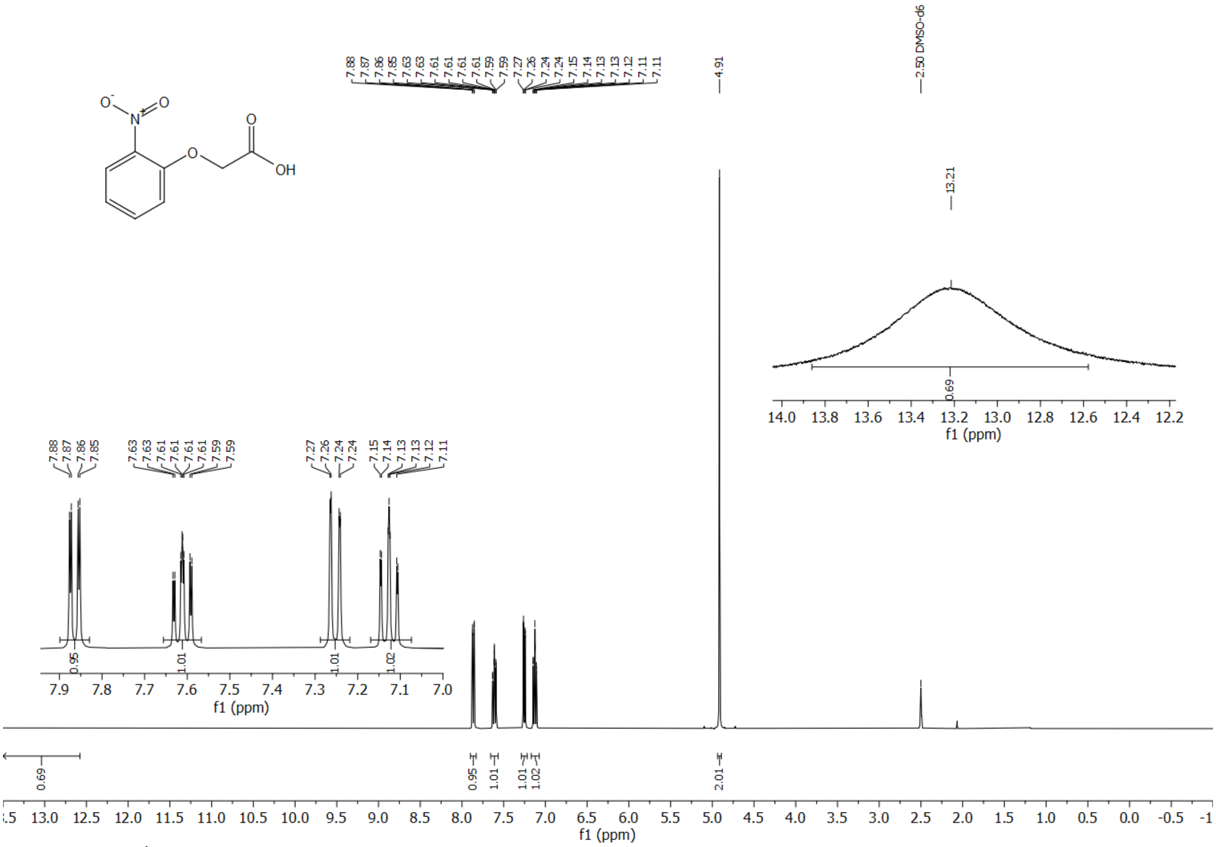

Figure S48: ^{1}H NMR spectrum (400 MHz, $DMSO_{d6}$) of **4a**.

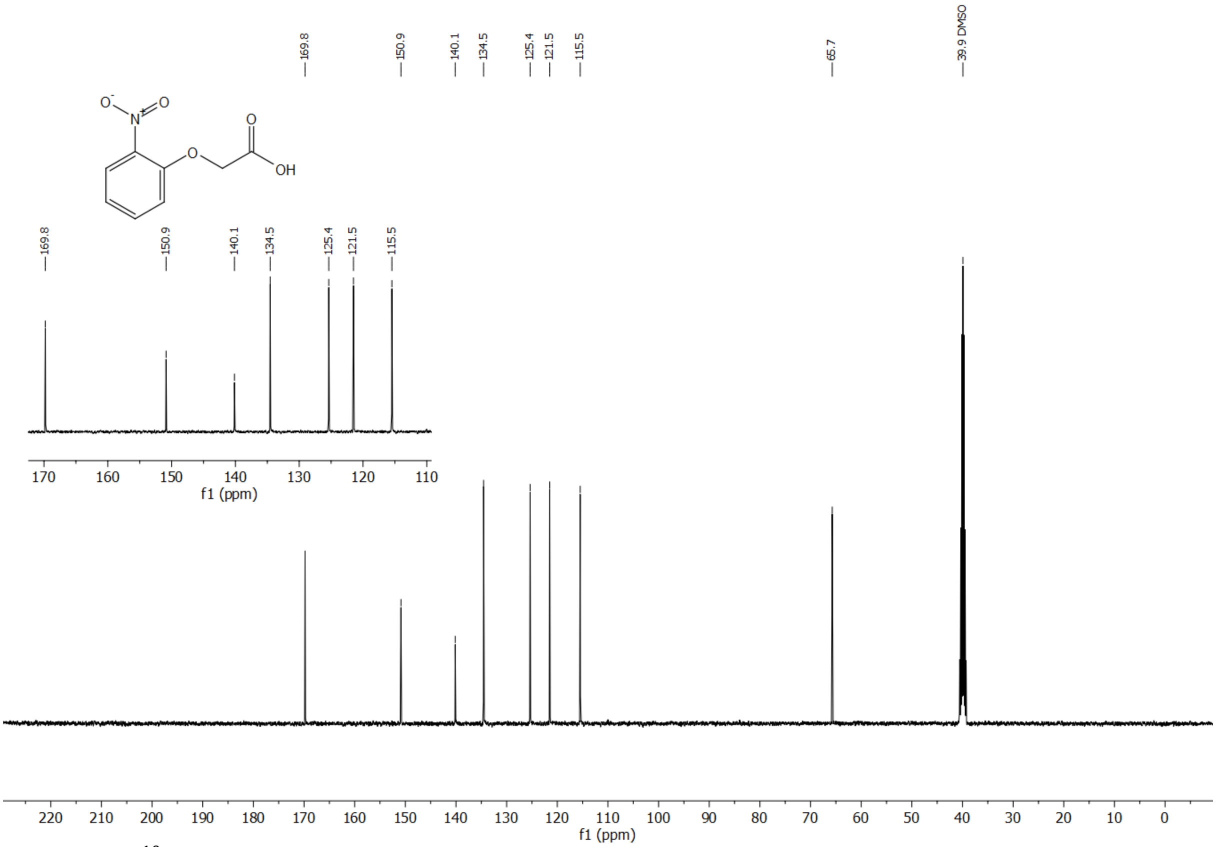

Figure S49: ^{13}C NMR spectrum (101 MHz, $DMSO_{d6}$) of **4a**.

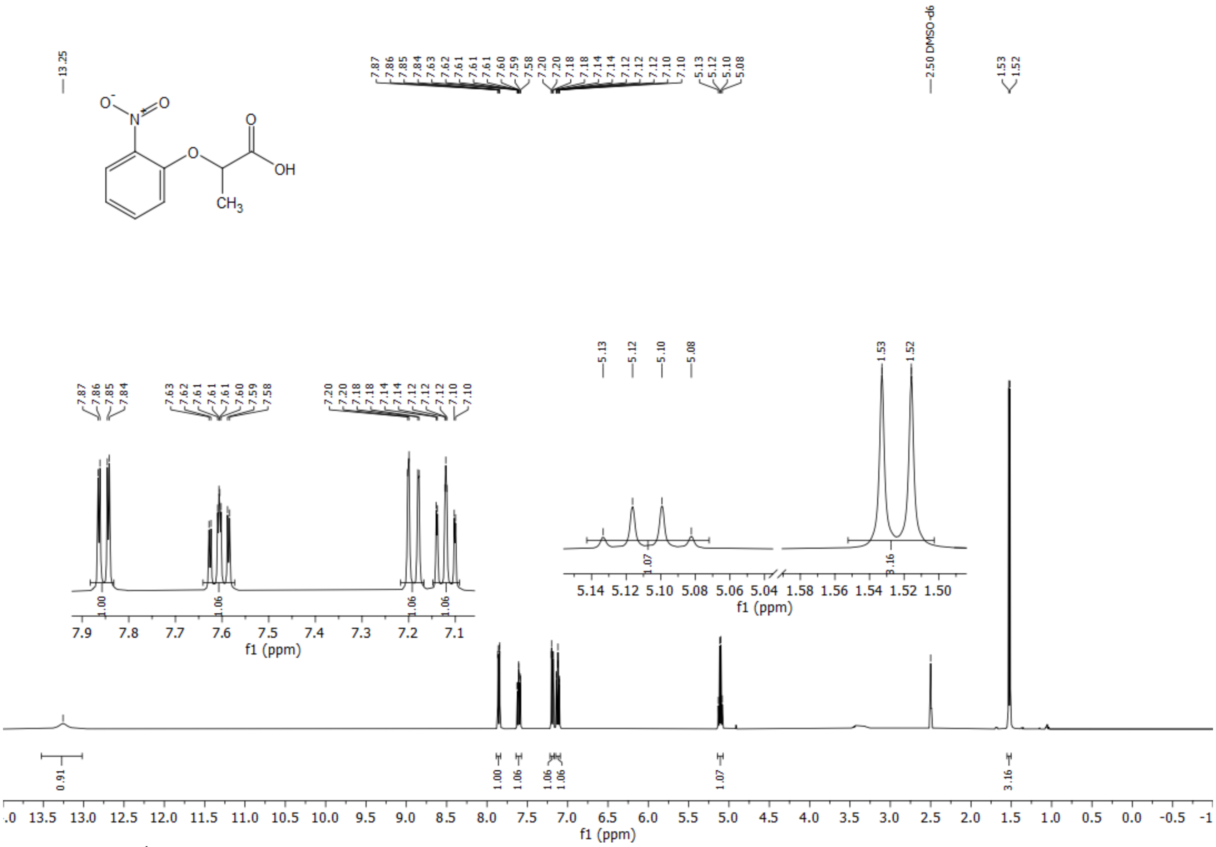

Figure S50: ^{1}H NMR spectrum (400 MHz, $DMSO_{d6}$) of **4b**.

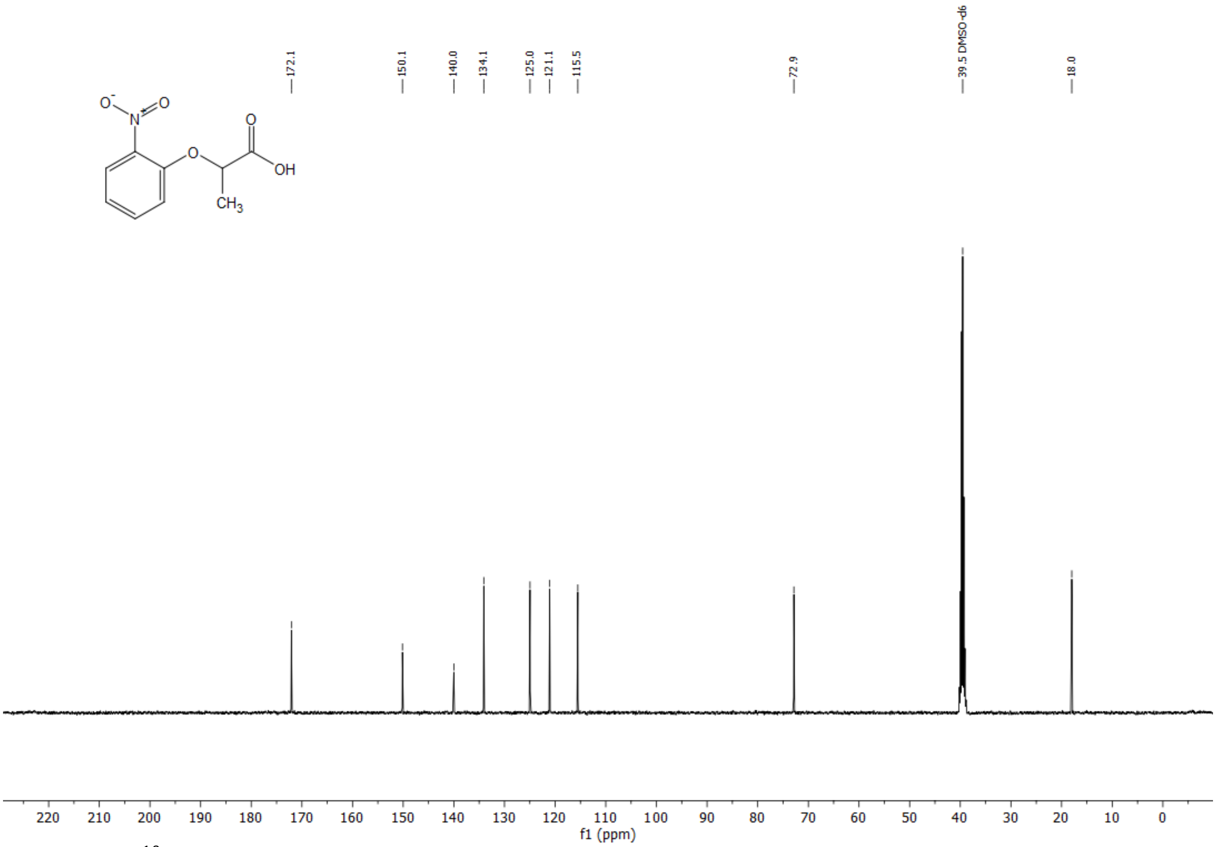

Figure S51: ^{13}C NMR spectrum (101 MHz, $DMSO_{d6}$) of **4b**.

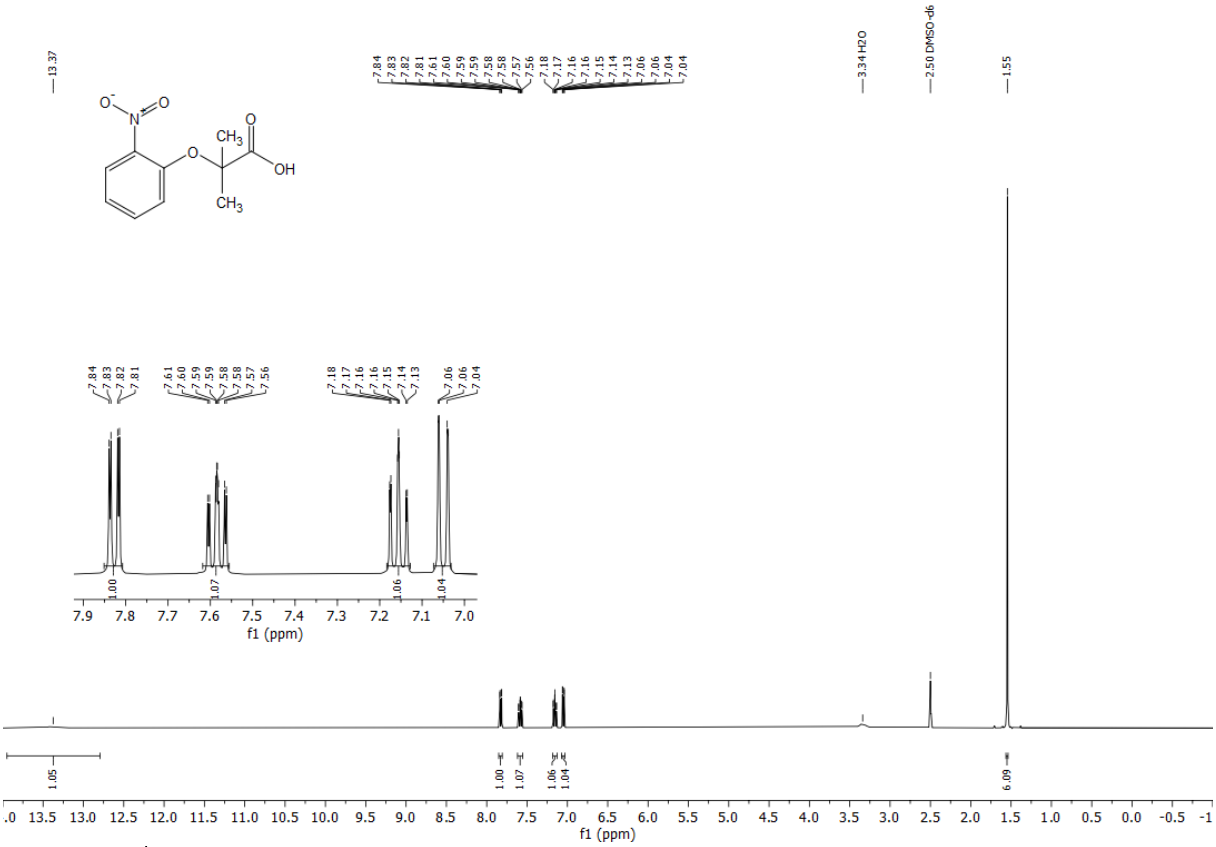

Figure S52: ^{1}H NMR spectrum (400 MHz, $DMSO_{d6}$) of **4c**.

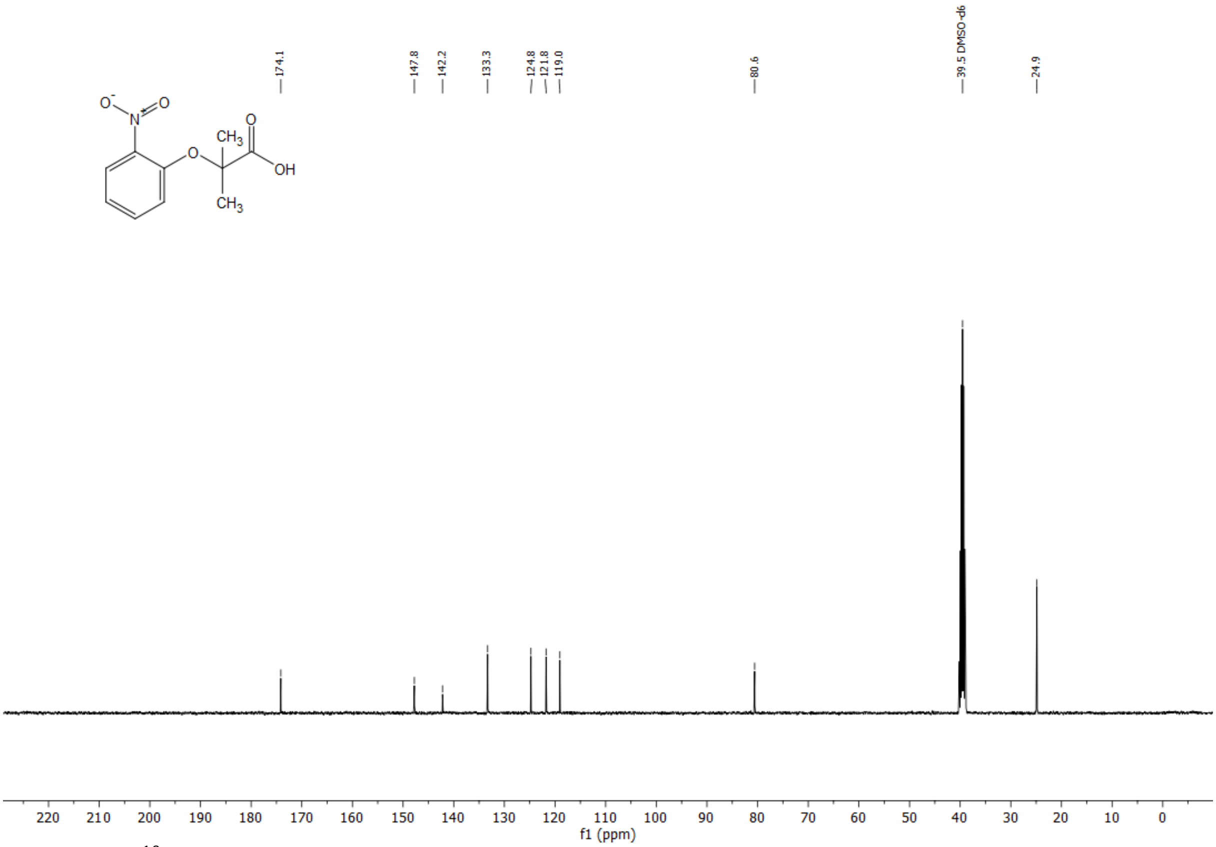

Figure S53: ^{13}C NMR spectrum (101 MHz, $DMSO_{d6}$) of **4c**.

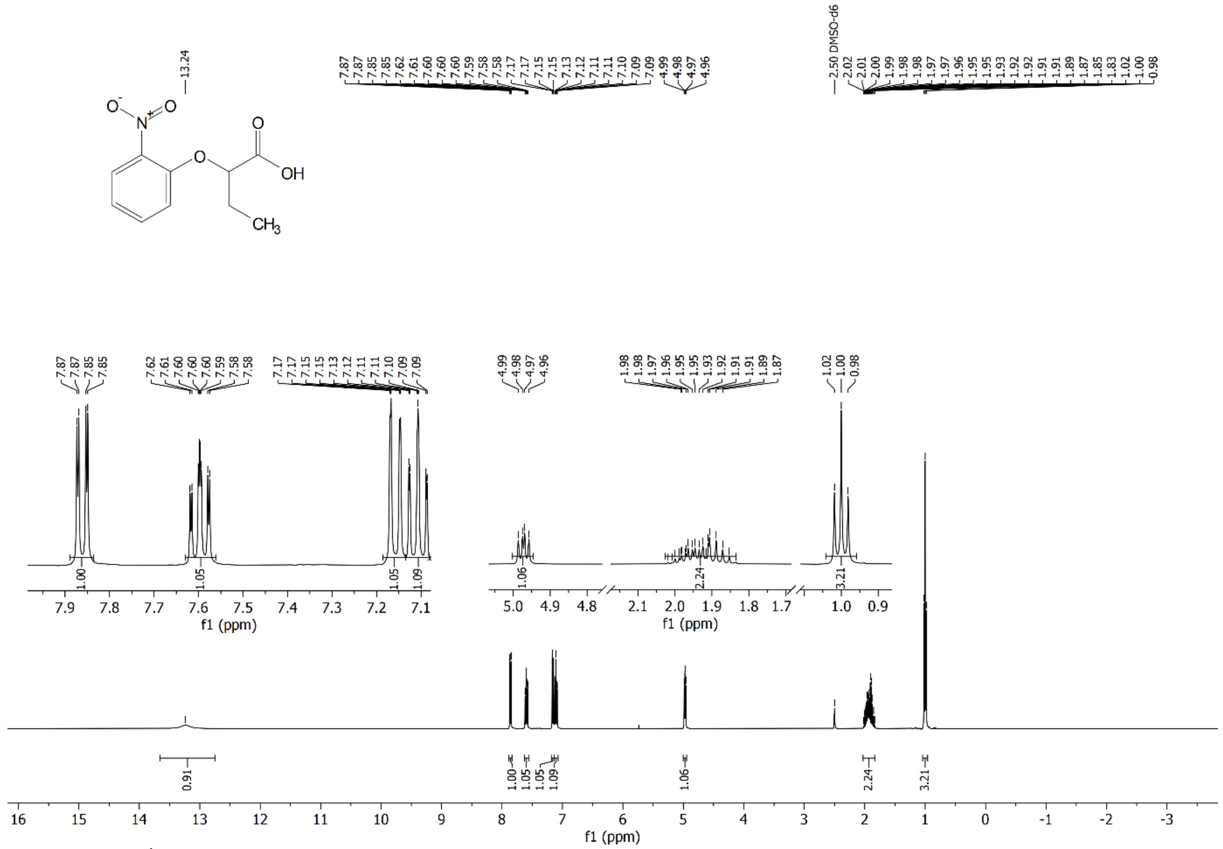

Figure S54: ^{1}H NMR spectrum (400 MHz, DMSO$_{d6}$) of **4d**.

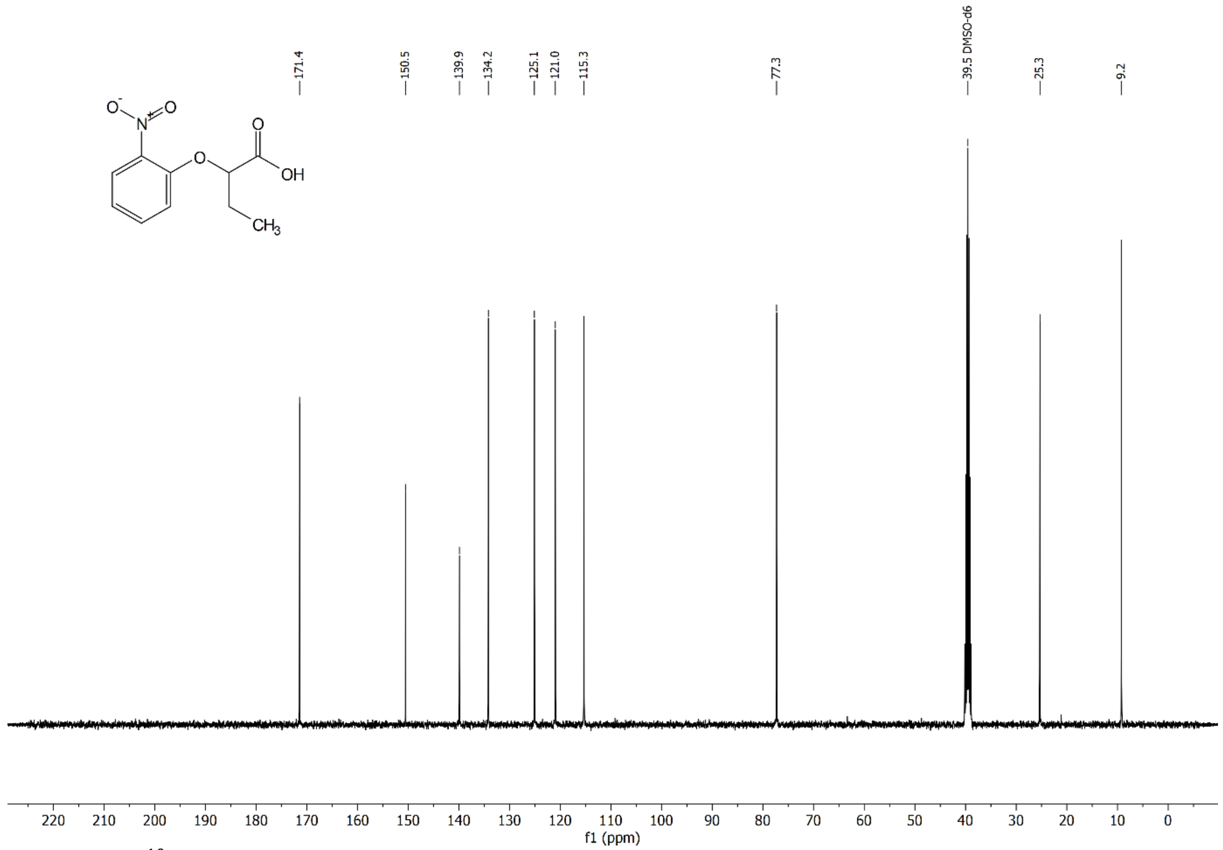

Figure S55: ^{13}C NMR spectrum (101 MHz, DMSO$_{d6}$) of **4d**.

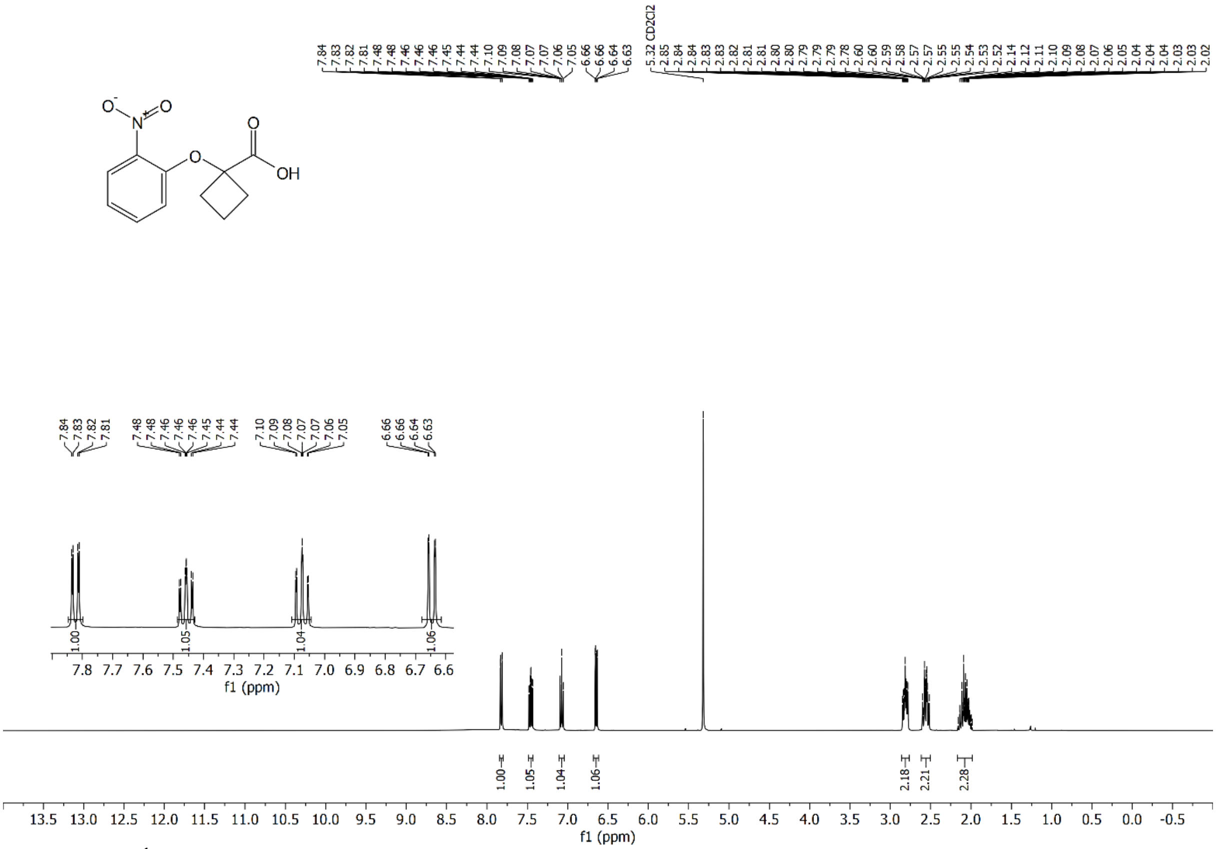

Figure S56: ^{1}H NMR spectrum (400 MHz, CD_2Cl_2) of **4e**.

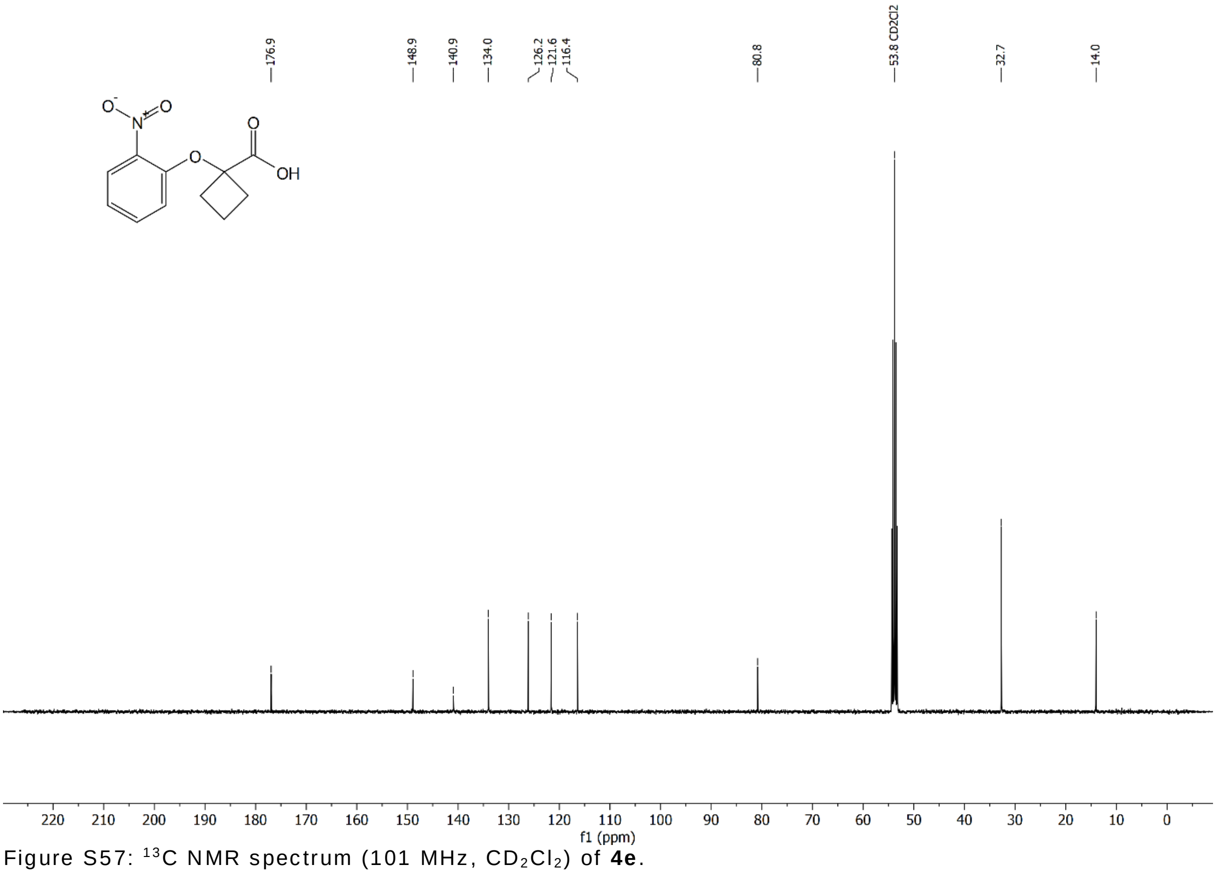

Figure S57: ^{13}C NMR spectrum (101 MHz, CD_2Cl_2) of **4e**.

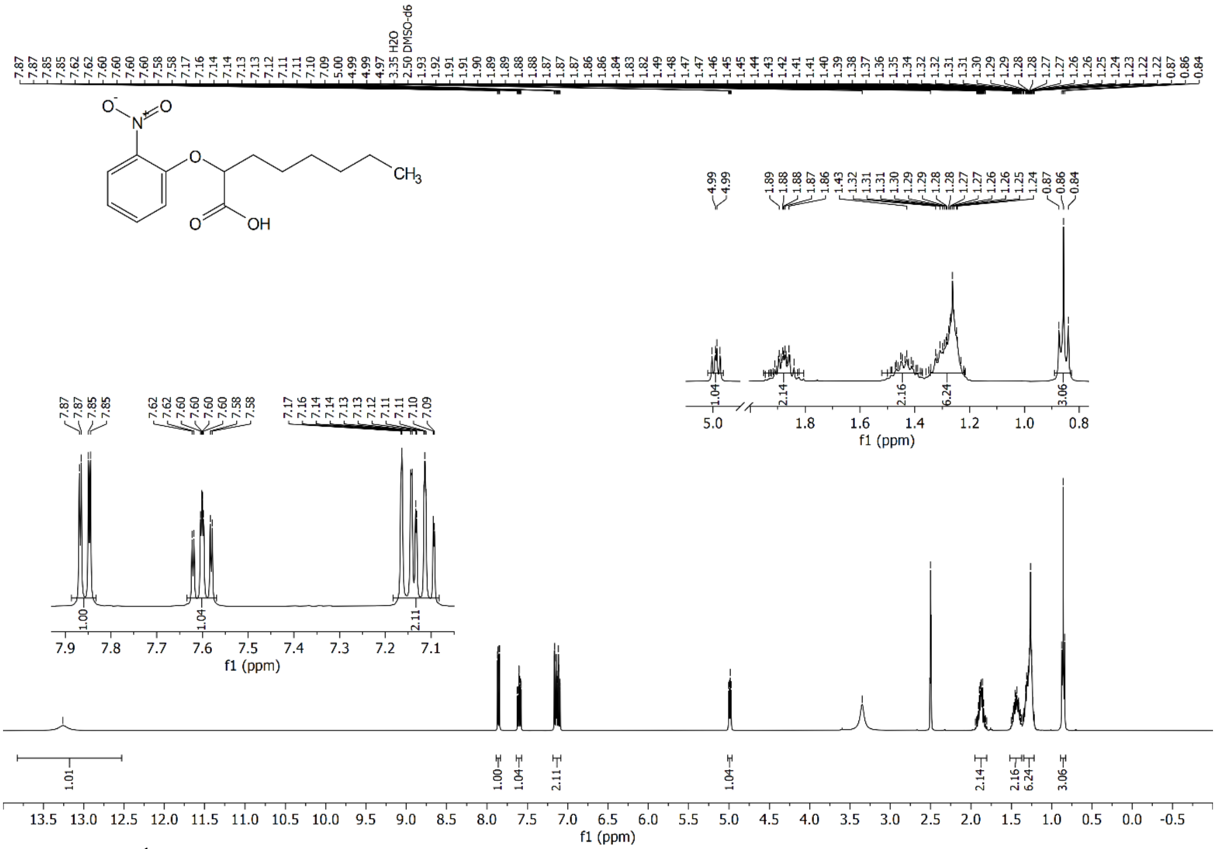

Figure S58: ^{1}H NMR spectrum (400 MHz, $DMSO_{d6}$) of **4f**.

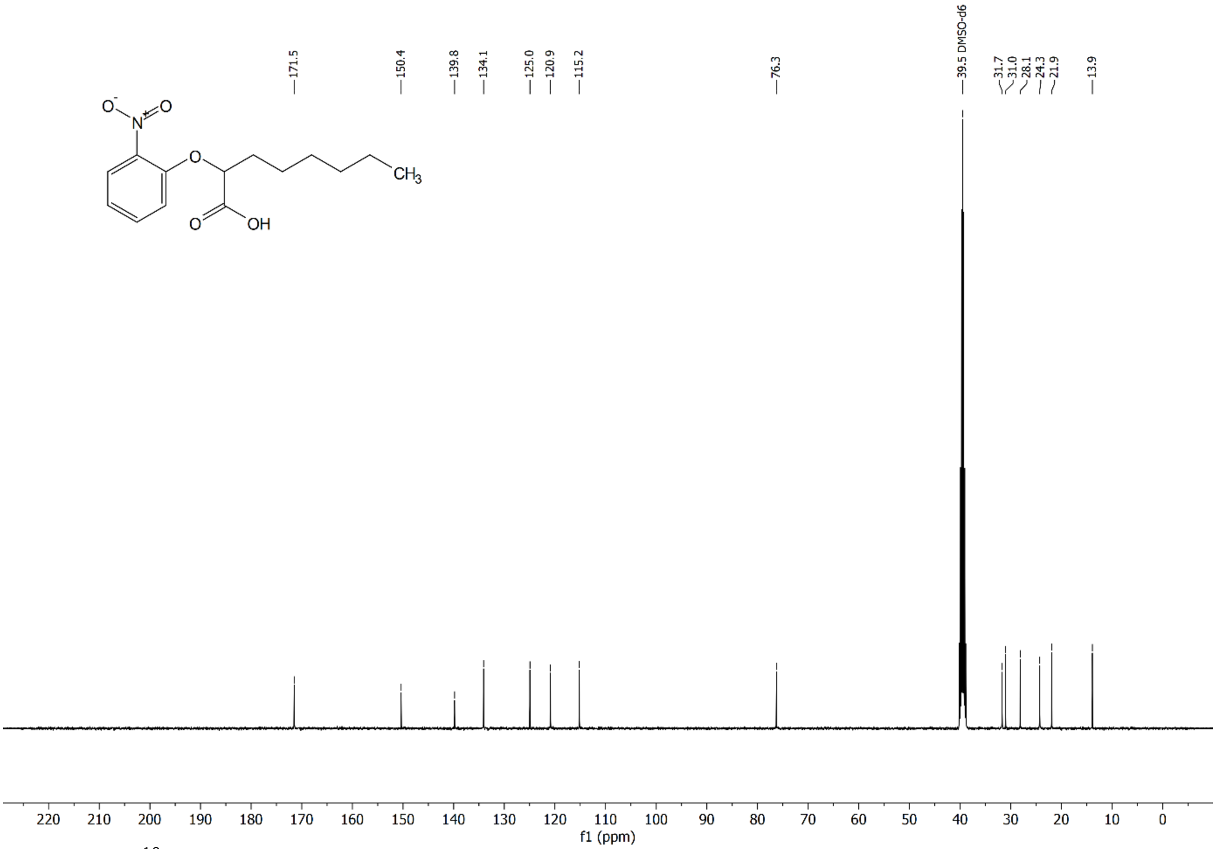

Figure S59: ^{13}C NMR spectrum (101 MHz, $DMSO_{d6}$) of **4f**.

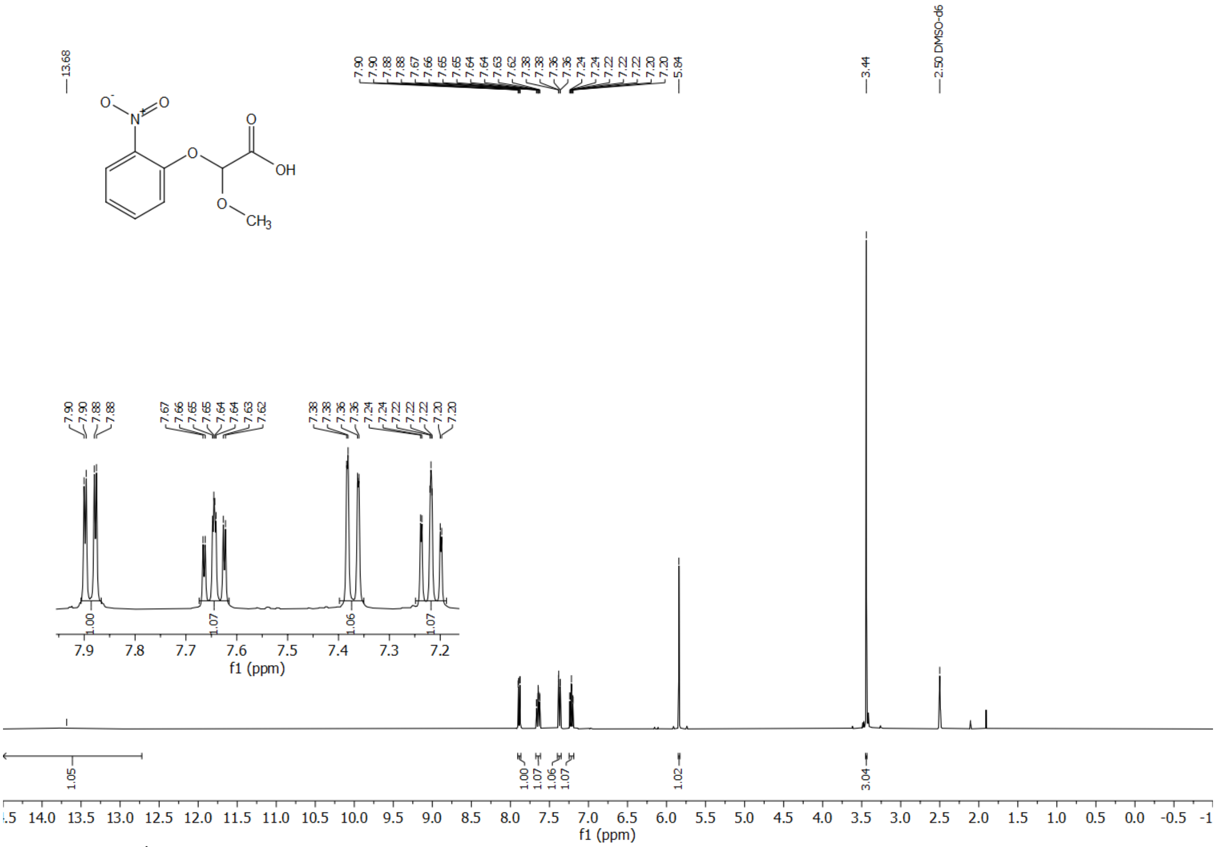

Figure S60: ^{1}H NMR spectrum (400 MHz, DMSO$_{d6}$) of **4g**.

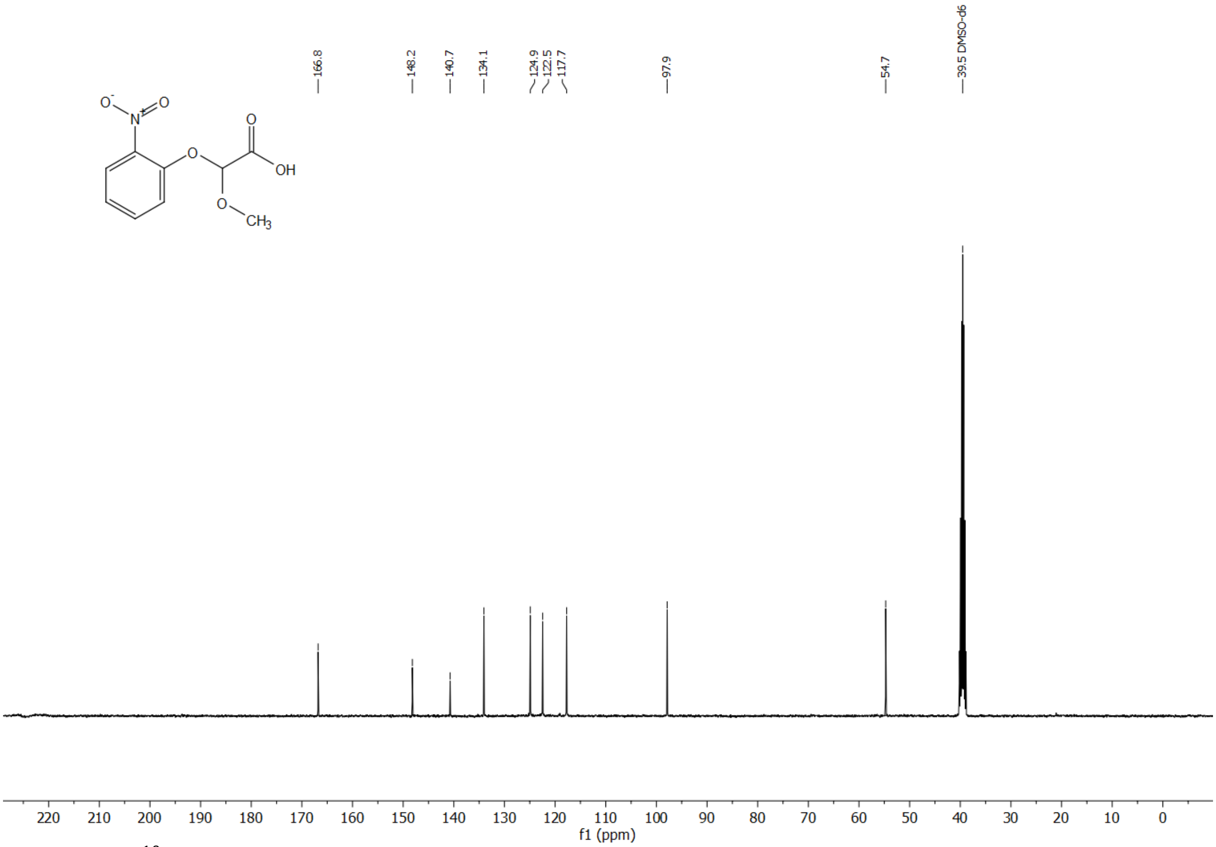

Figure S61: ^{13}C NMR spectrum (101 MHz, DMSO$_{d6}$) of **4g**.

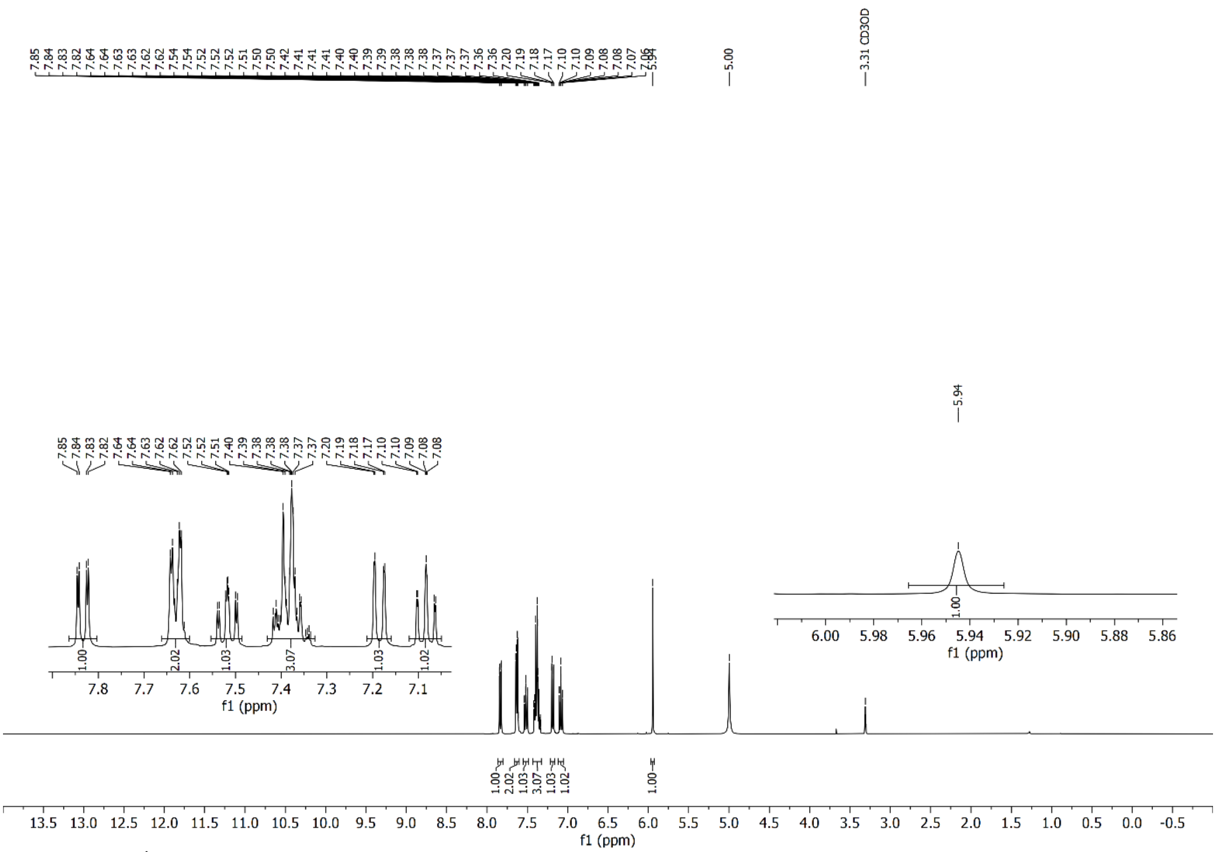

Figure S62: ^{1}H NMR spectrum (400 MHz, CD_3OD) of **4h**.

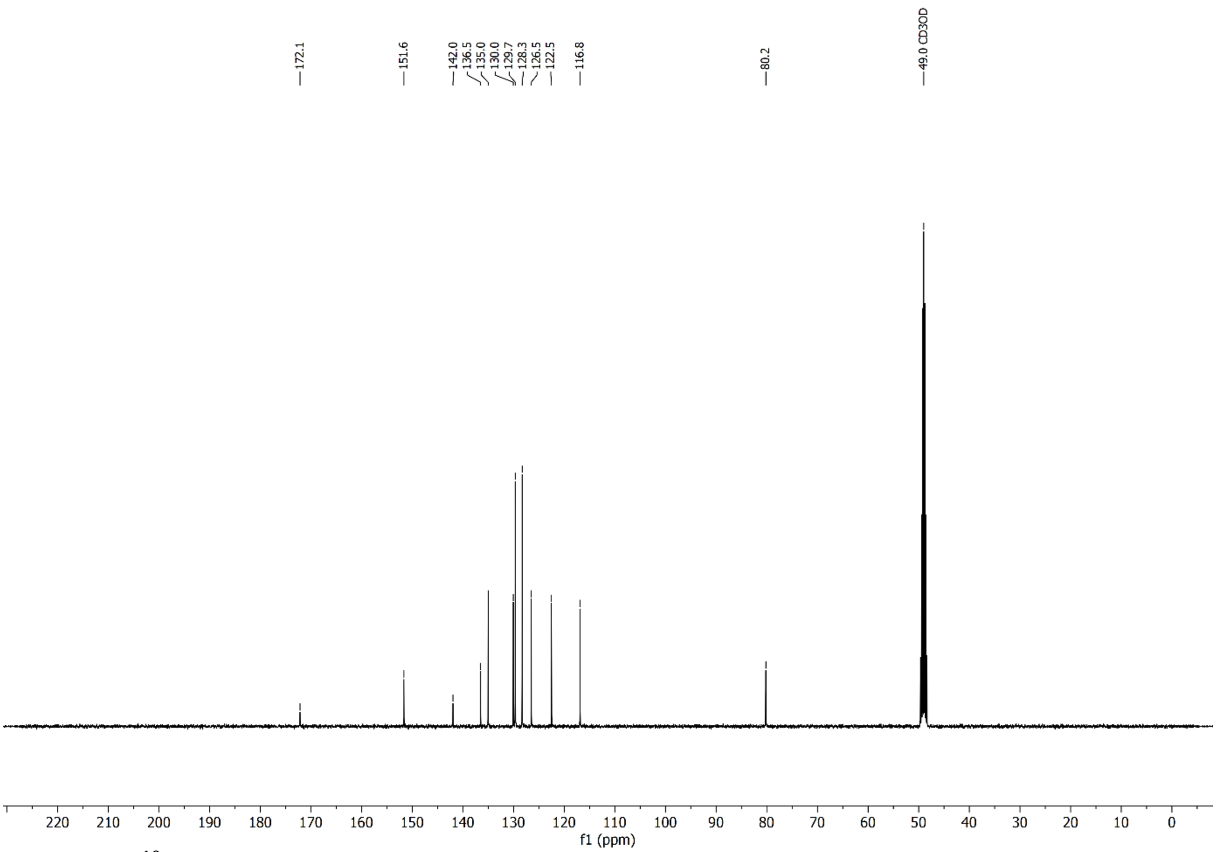

Figure S63: ^{13}C NMR spectrum (101 MHz, CD_3OD) of **4h**.

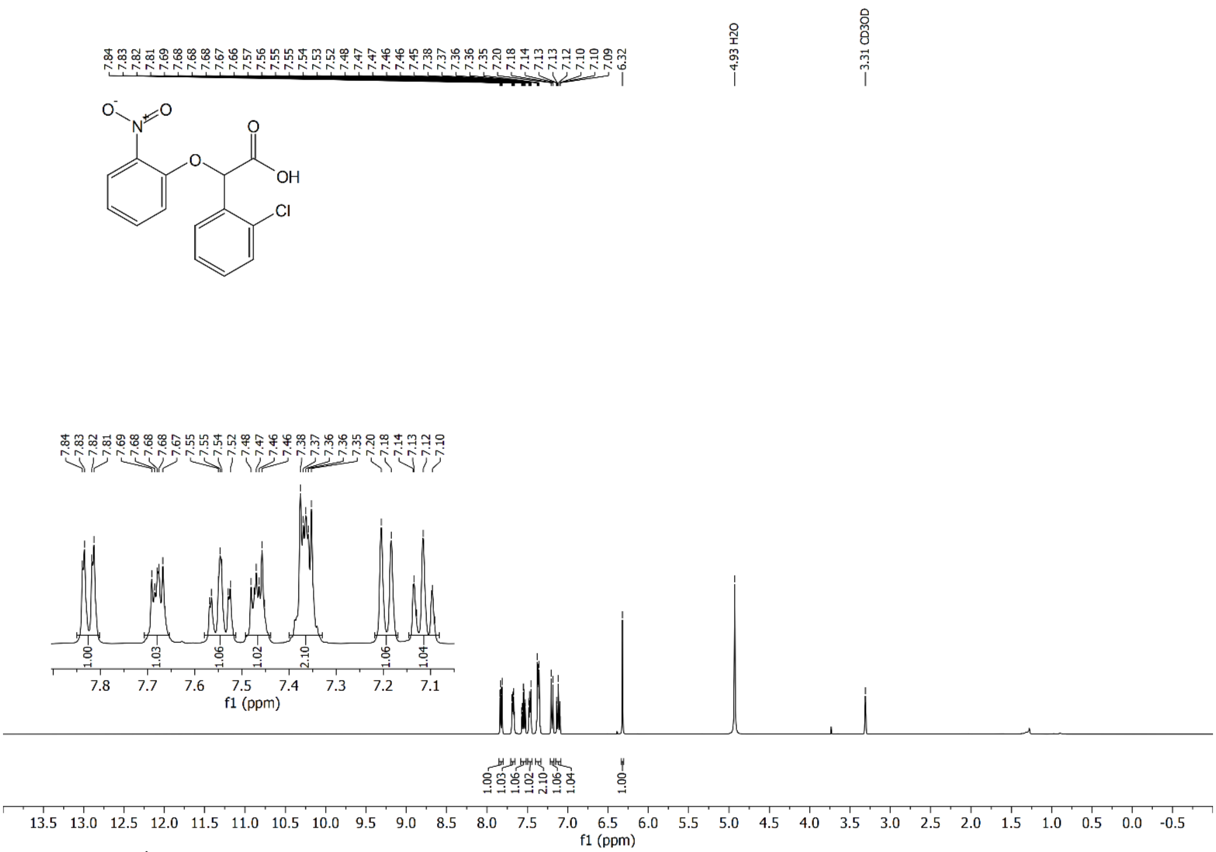

Figure S64: ^{1}H NMR spectrum (400 MHz, CD_3OD) of **4i**.

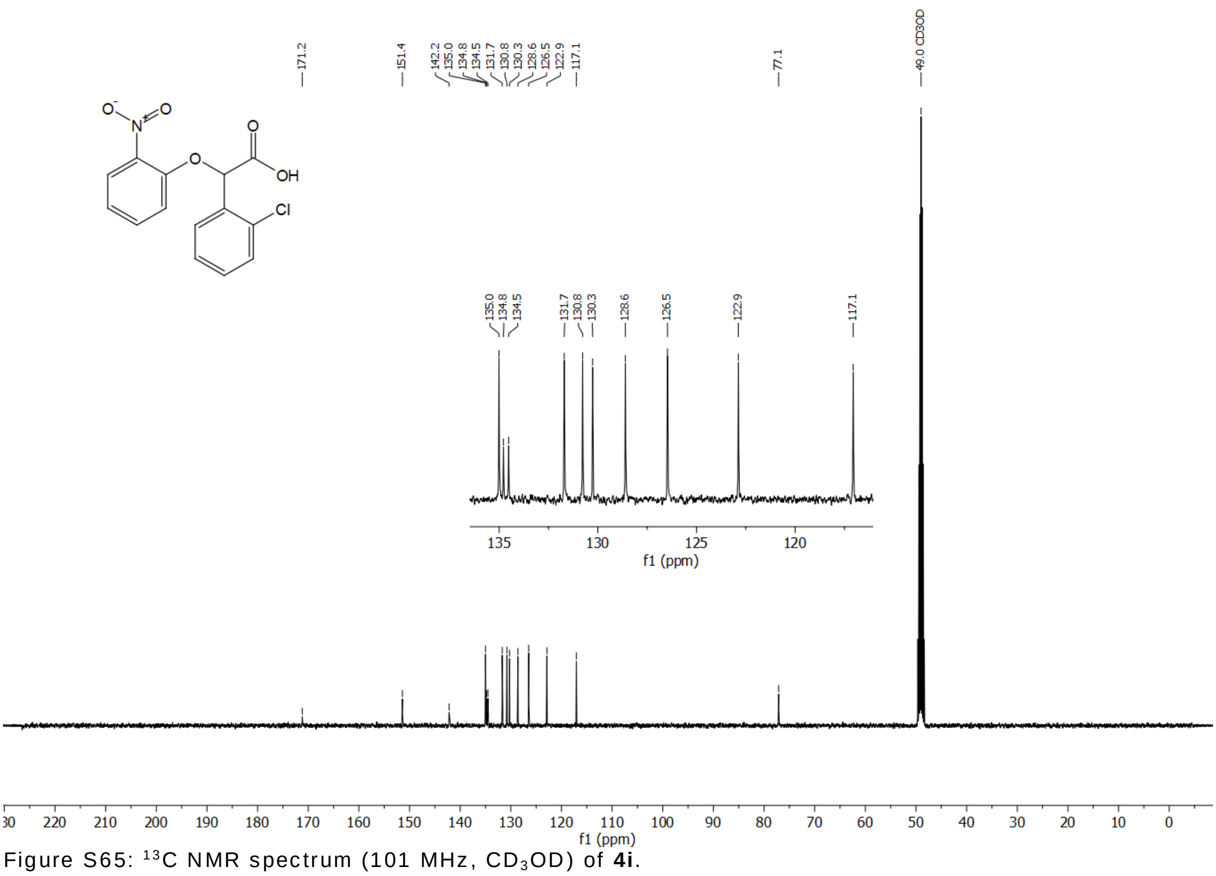

Figure S65: ^{13}C NMR spectrum (101 MHz, CD_3OD) of **4i**.

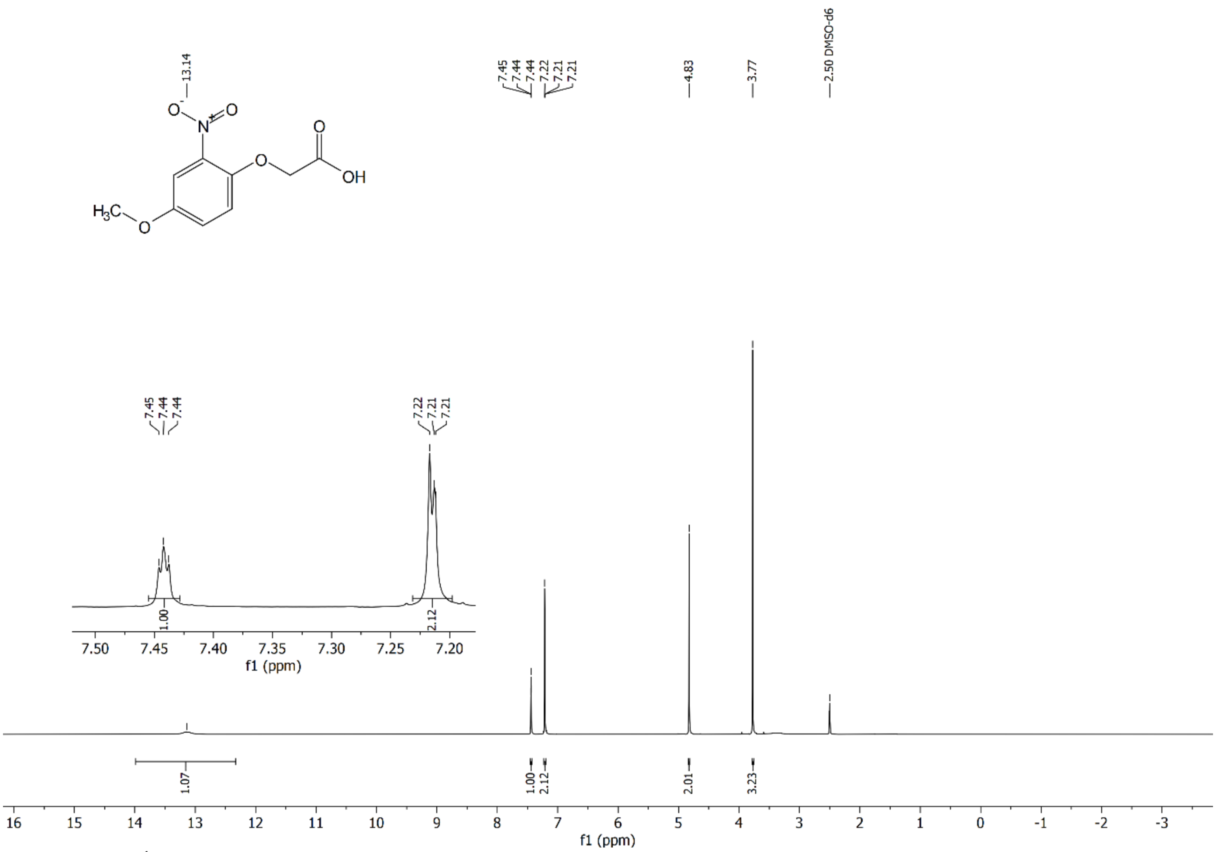

Figure S66: ^{1}H NMR spectrum (400 MHz, $DMSO_{d6}$) of **4j**.

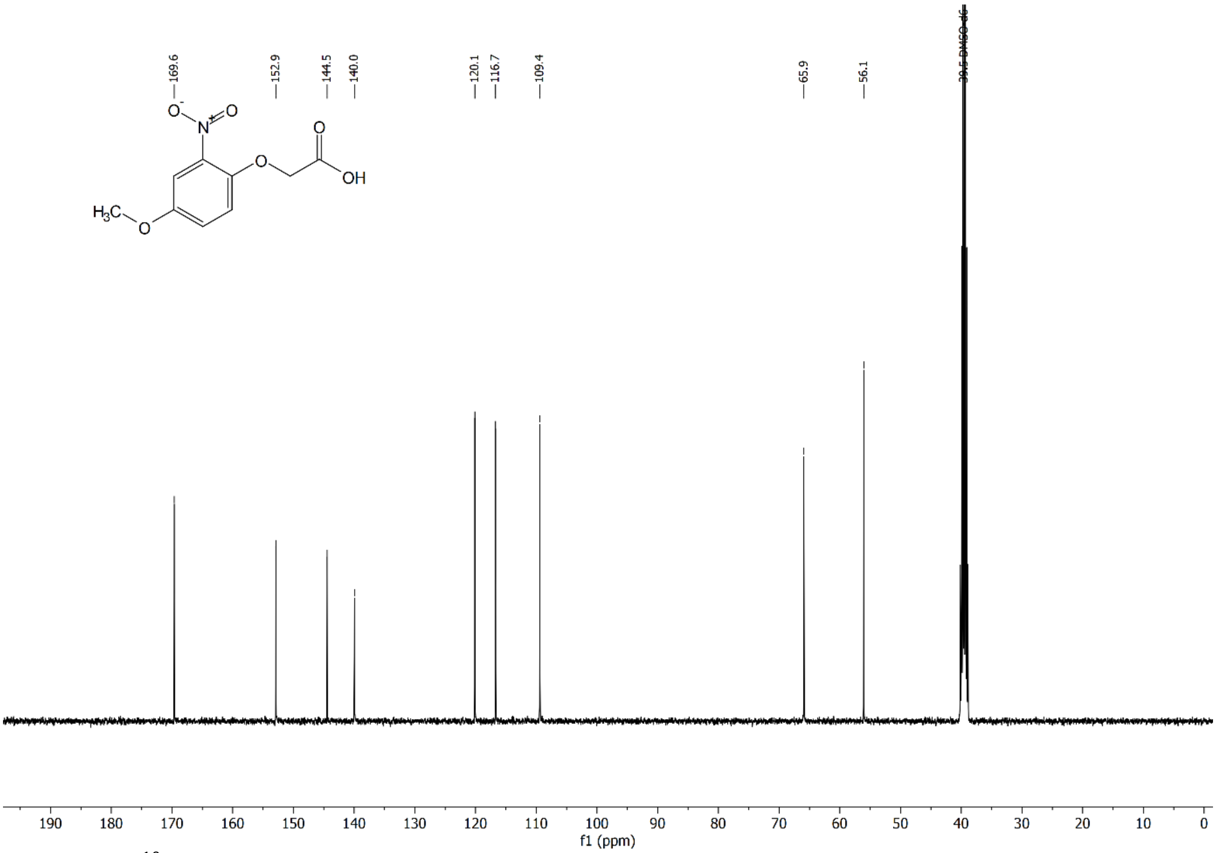

Figure S67: ^{13}C NMR spectrum (101 MHz, $DMSO_{d6}$) of **4j**.

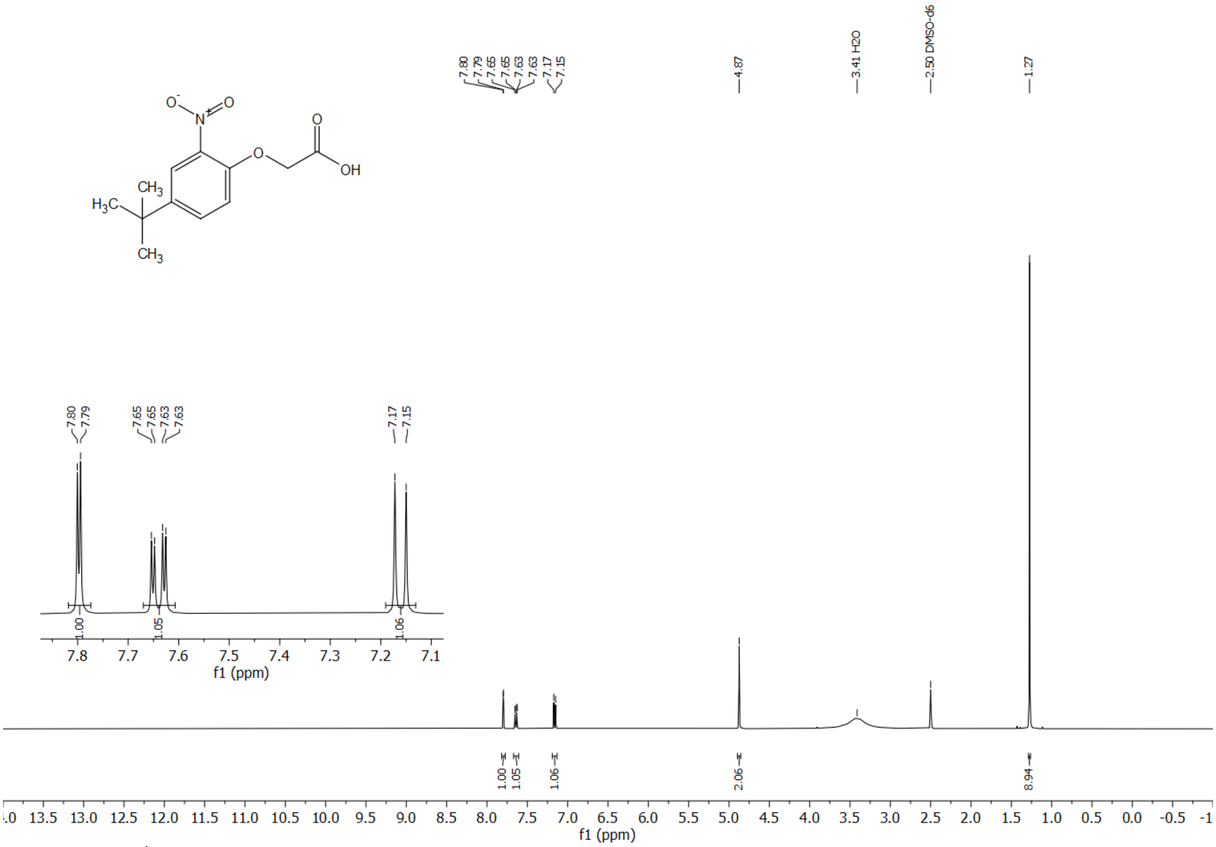

Figure S68: ^{1}H NMR spectrum (400 MHz, DMSO$_{d6}$) of **4k**.

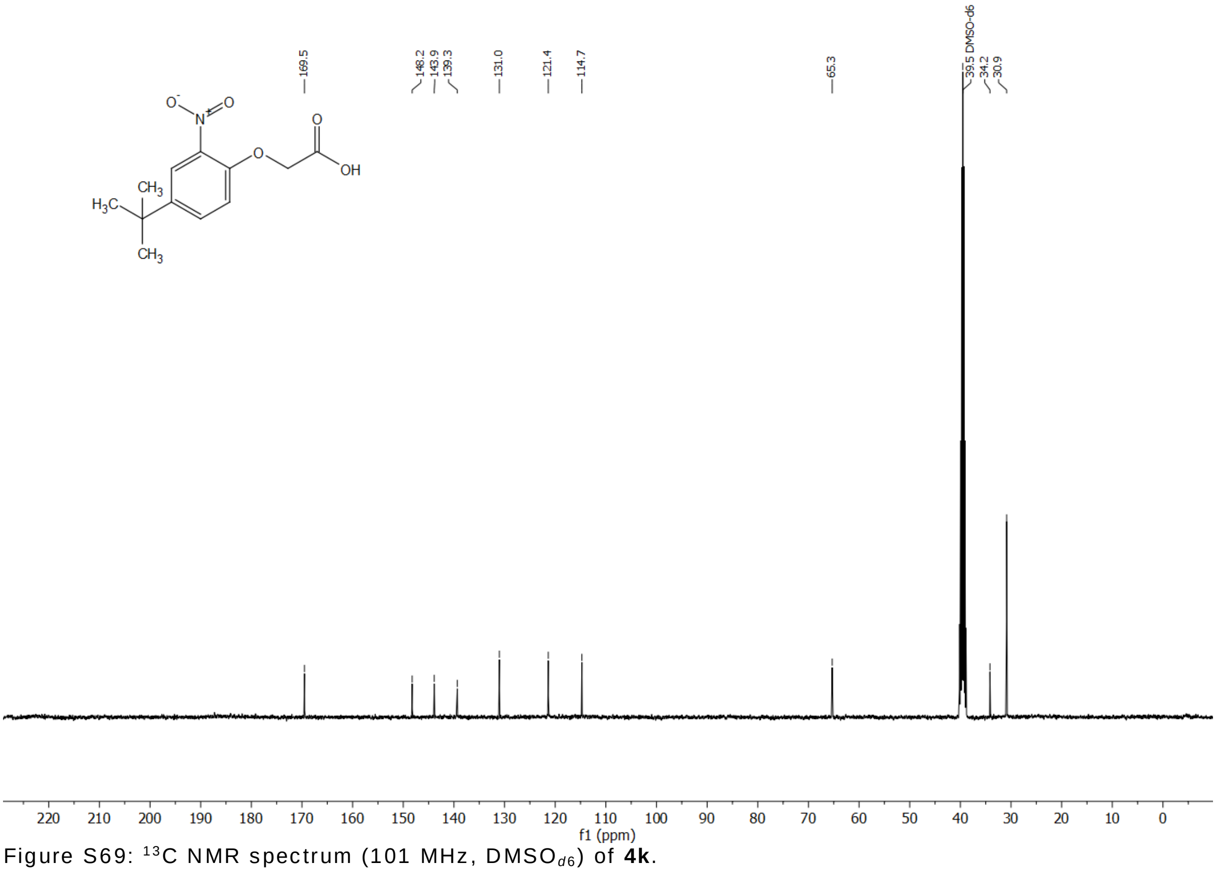

Figure S69: ^{13}C NMR spectrum (101 MHz, DMSO$_{d6}$) of **4k**.

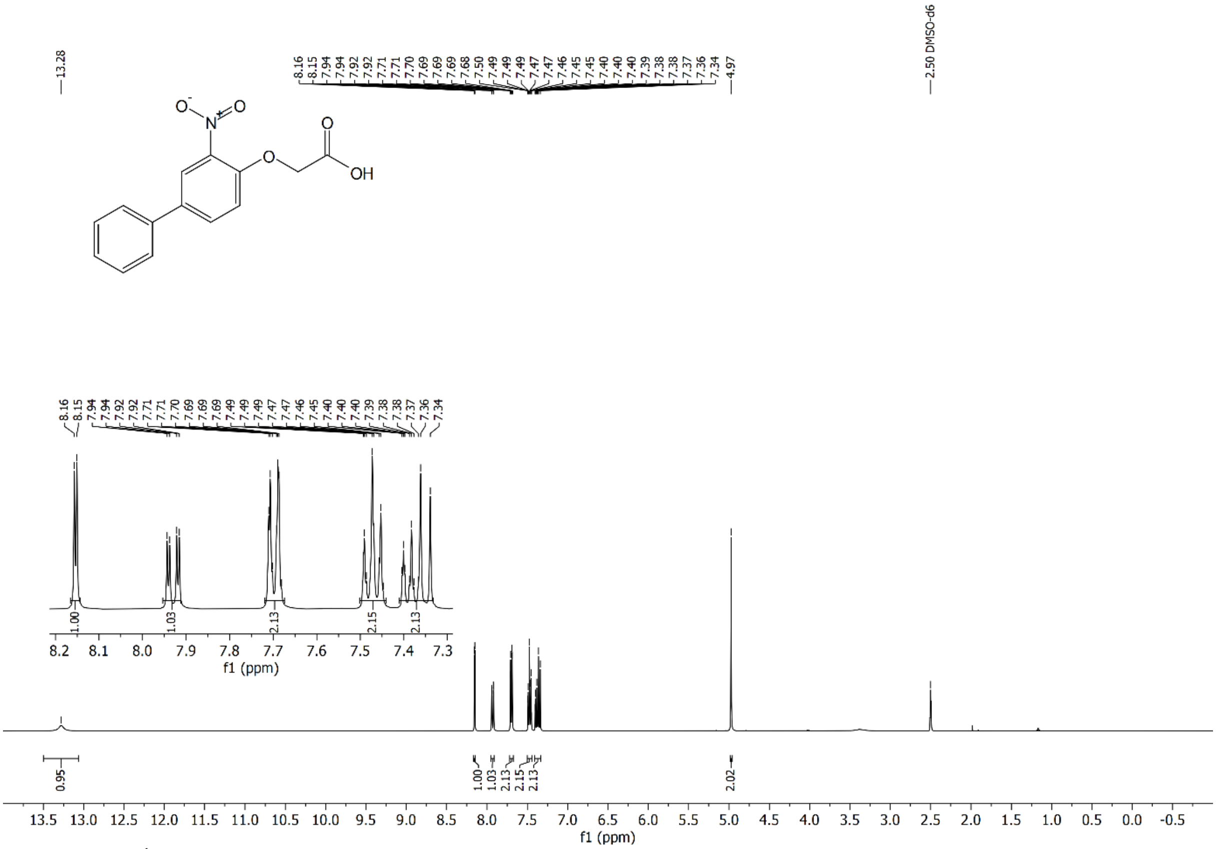

Figure S70: ^{1}H NMR spectrum (400 MHz, $DMSO_{d6}$) of **4l**.

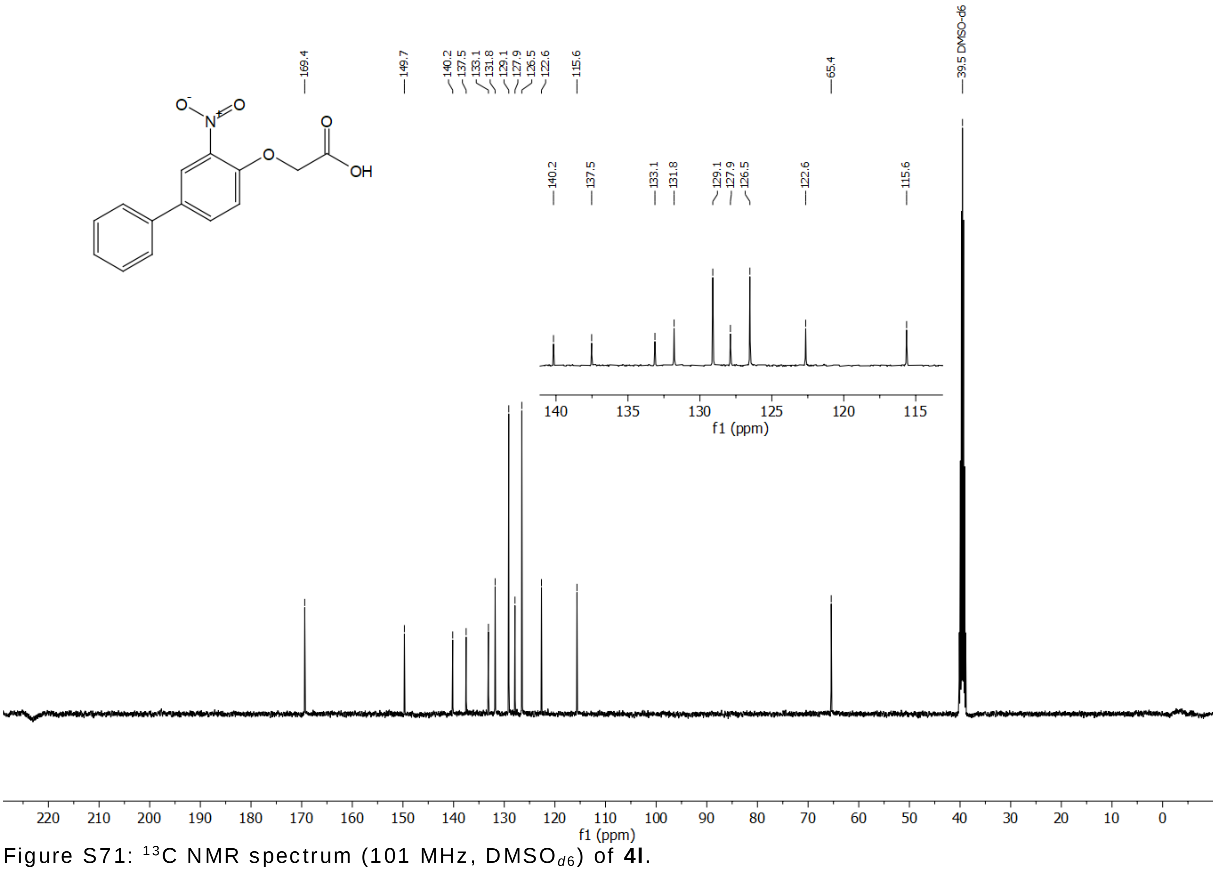

Figure S71: ^{13}C NMR spectrum (101 MHz, $DMSO_{d6}$) of **4l**.

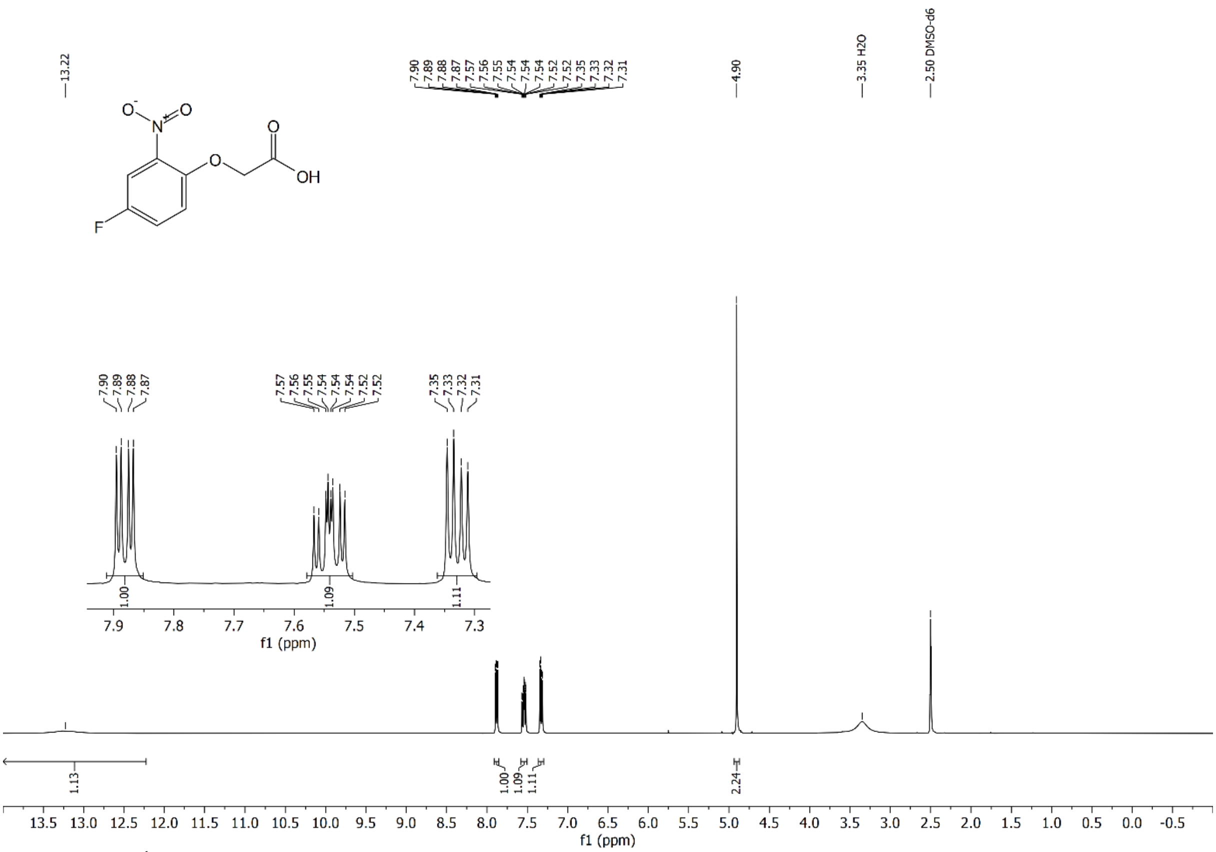

Figure S72: ^{1}H NMR spectrum (400 MHz, $DMSO_{d6}$) of **4m**.

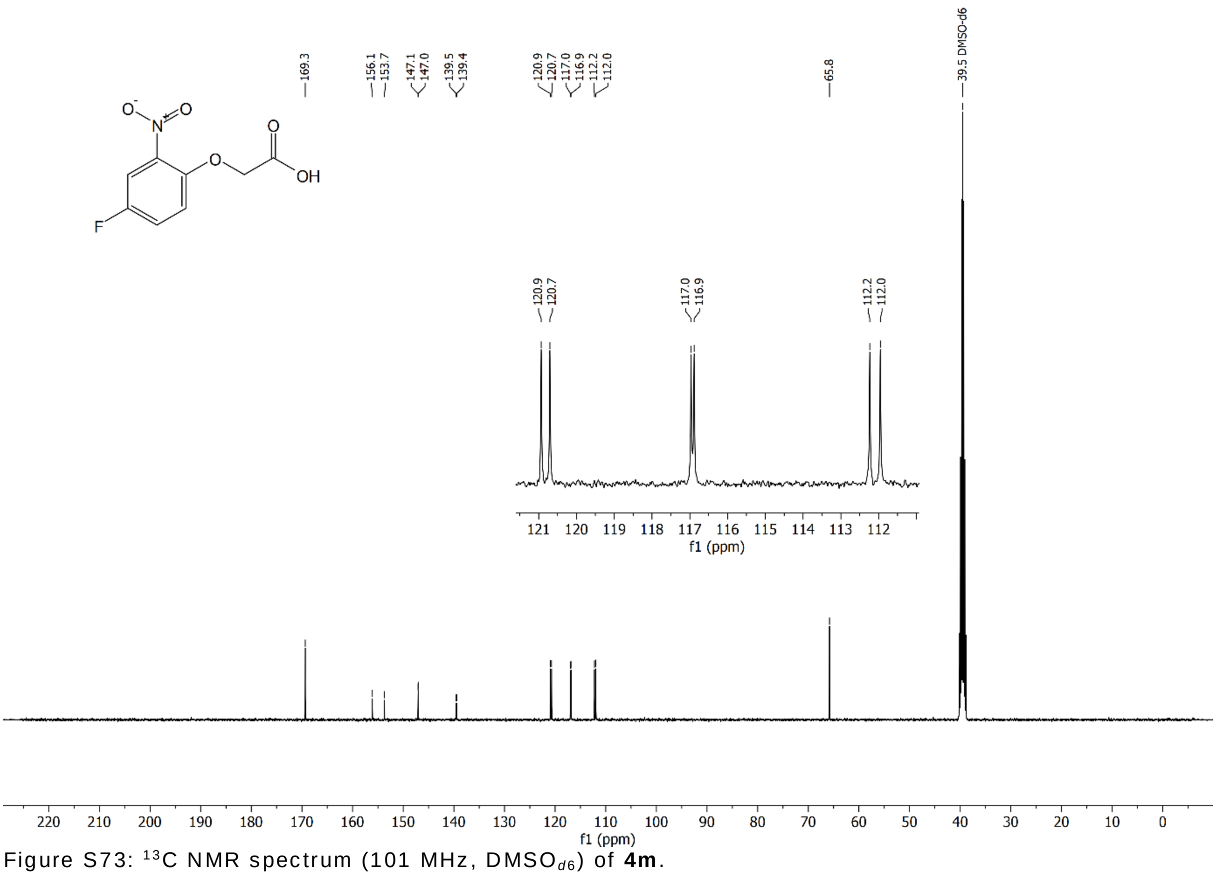

Figure S73: ^{13}C NMR spectrum (101 MHz, $DMSO_{d6}$) of **4m**.

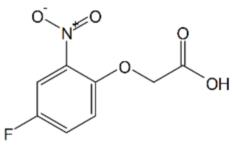

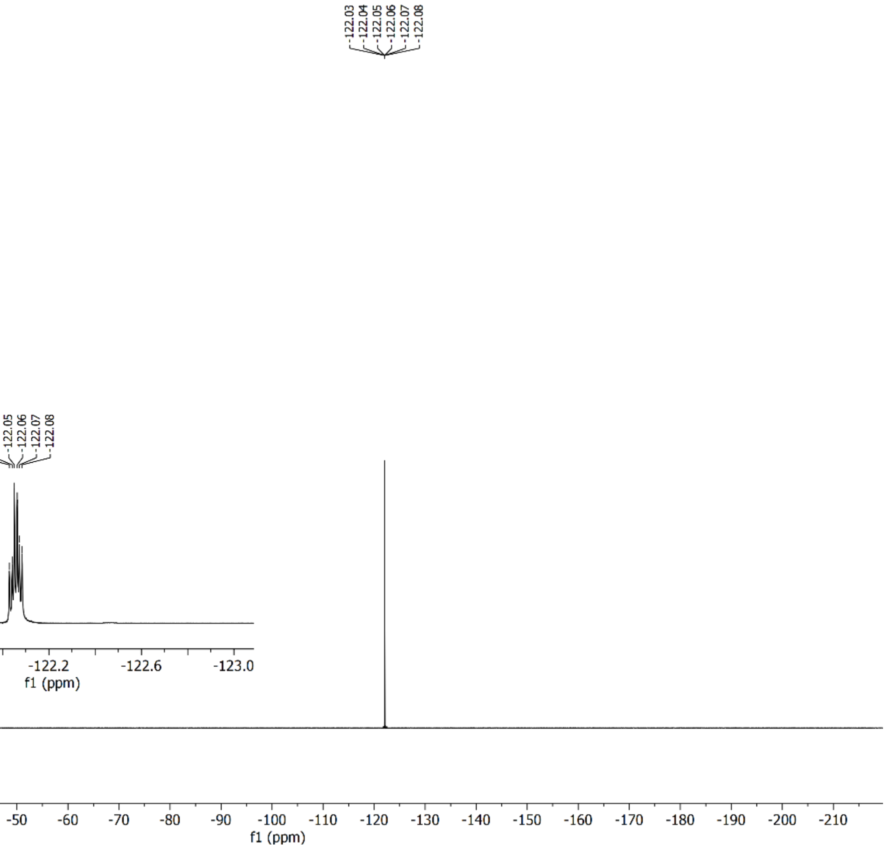

Figure S74: ^{19}F NMR spectrum (376 MHz, DMSO$_{d6}$) of **4m**.

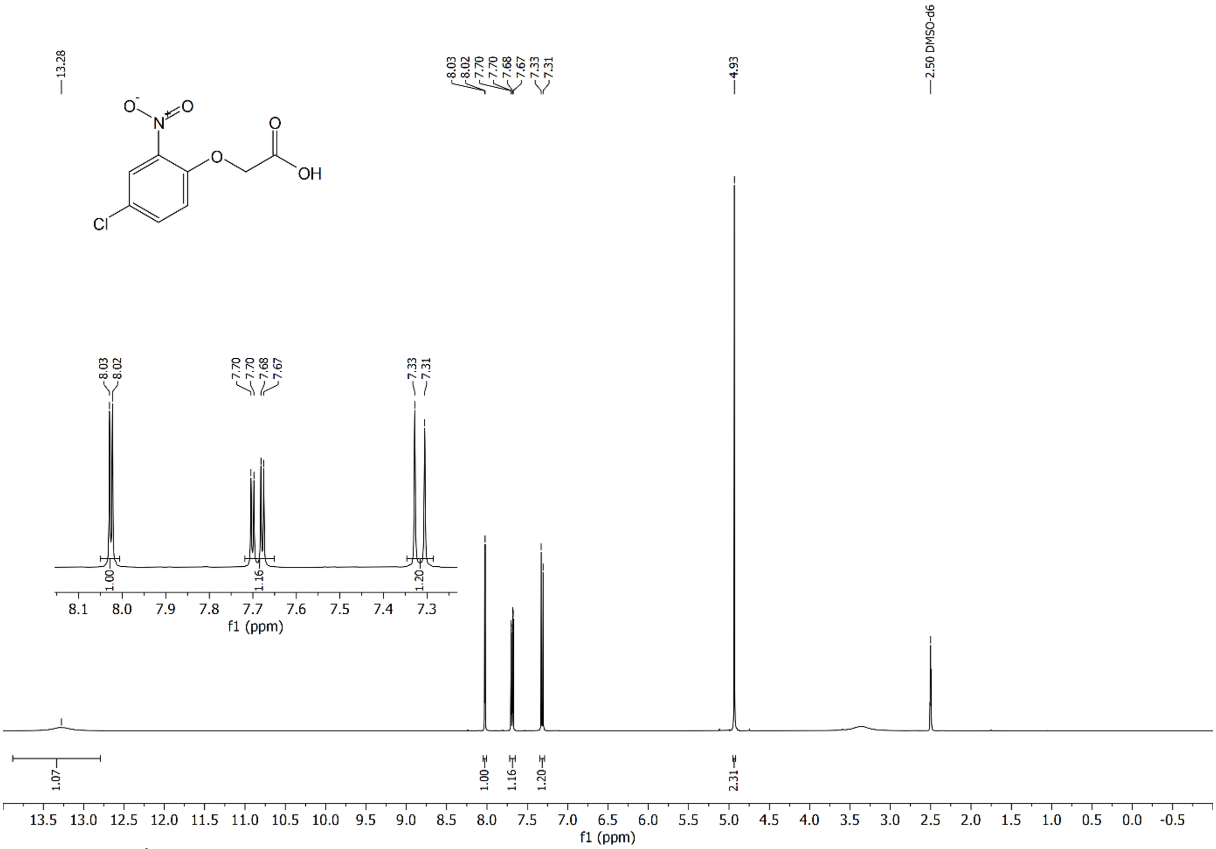

Figure S75: ^{1}H NMR spectrum (400 MHz, $DMSO_{d6}$) of **4n**.

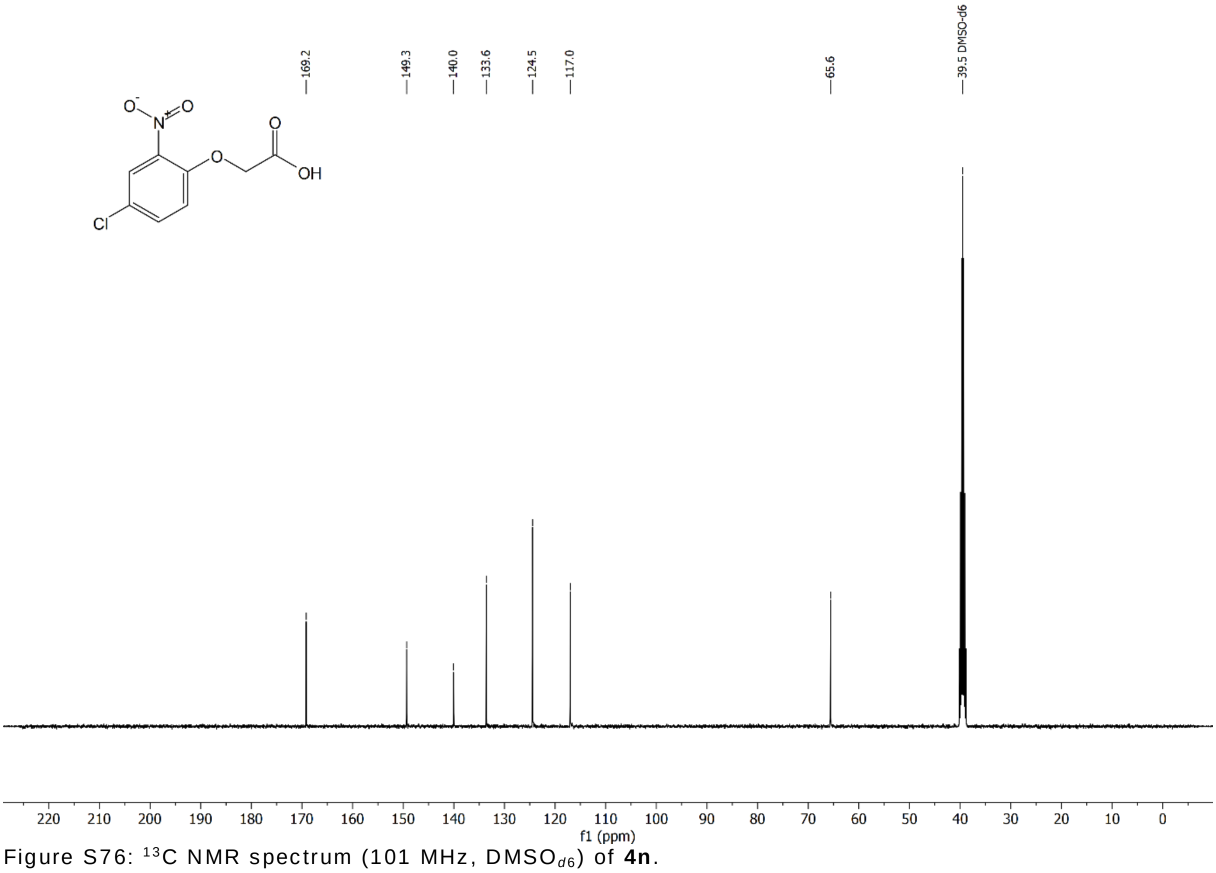

Figure S76: ^{13}C NMR spectrum (101 MHz, $DMSO_{d6}$) of **4n**.

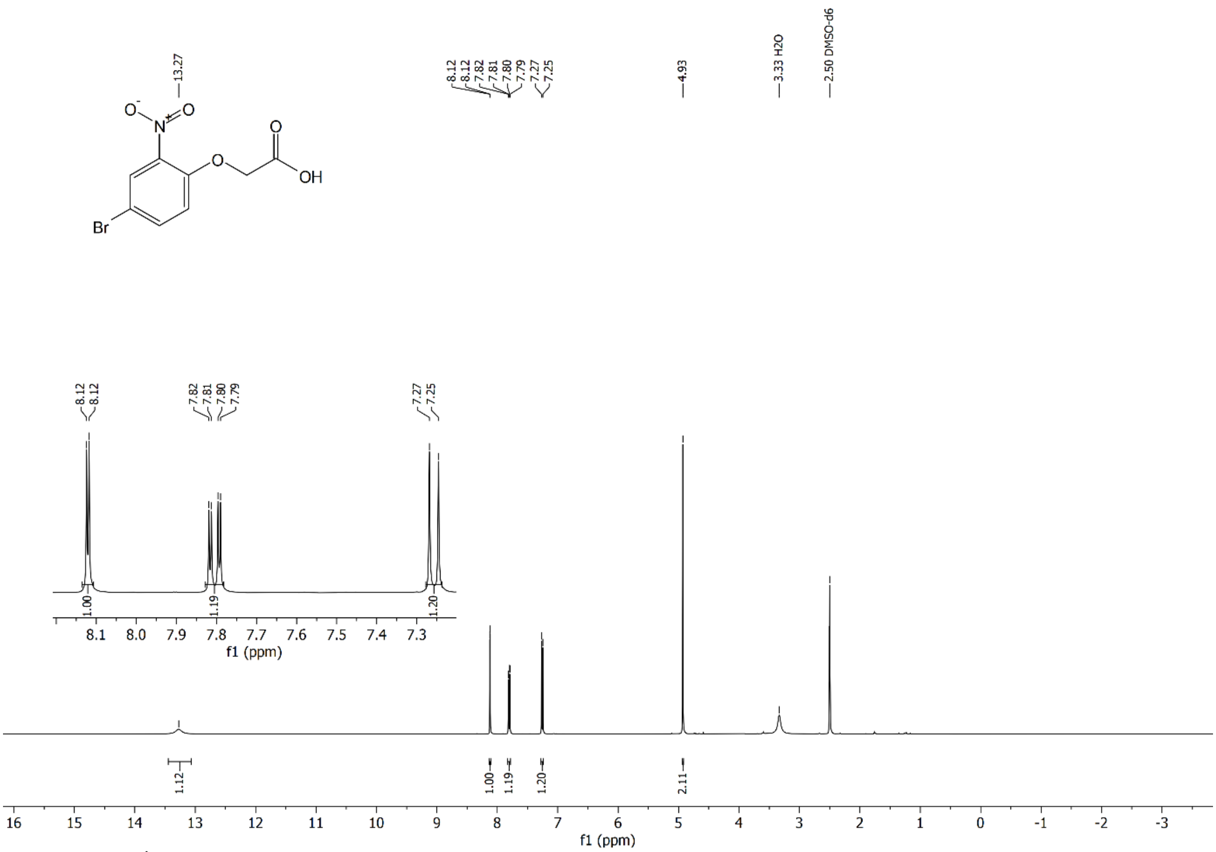

Figure S77: ^{1}H NMR spectrum (400 MHz, DMSO$_{d6}$) of **4o**.

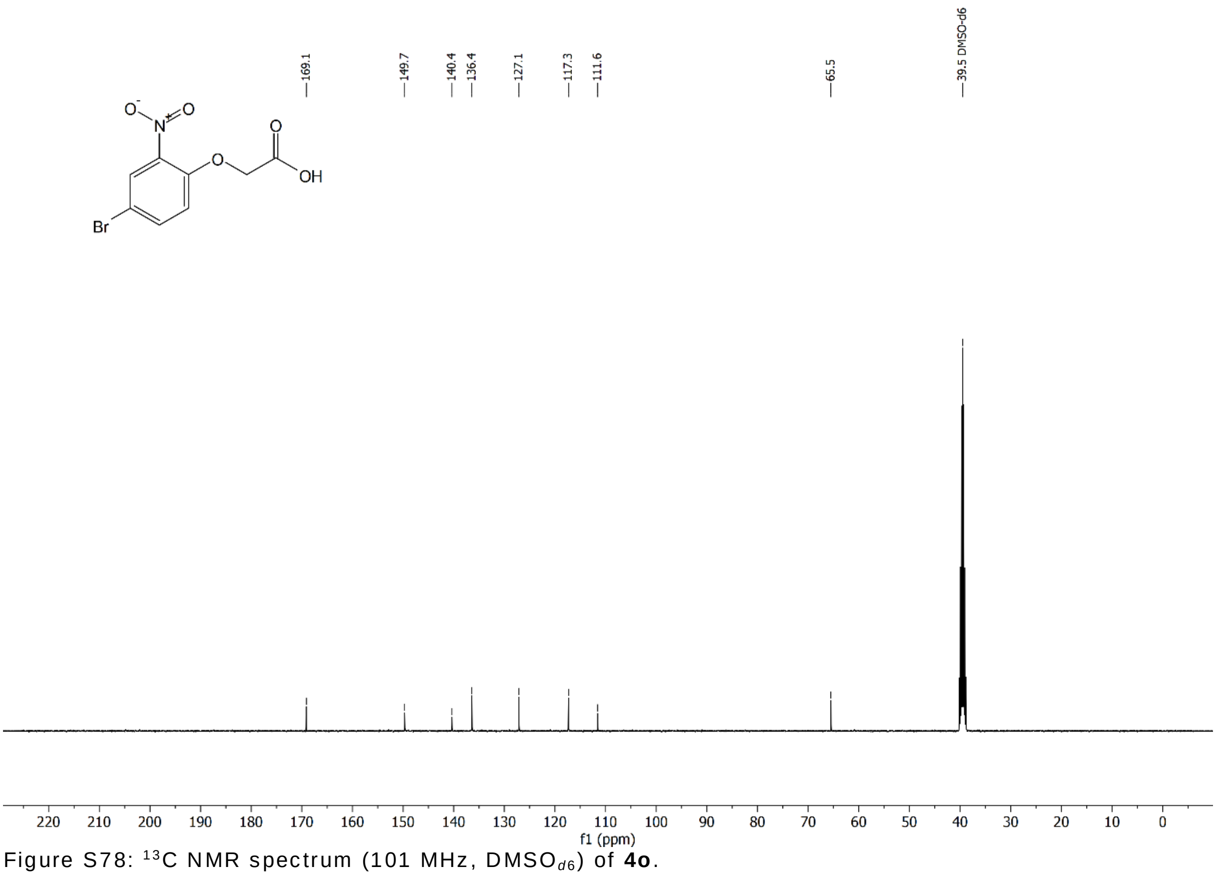

Figure S78: ^{13}C NMR spectrum (101 MHz, DMSO$_{d6}$) of **4o**.

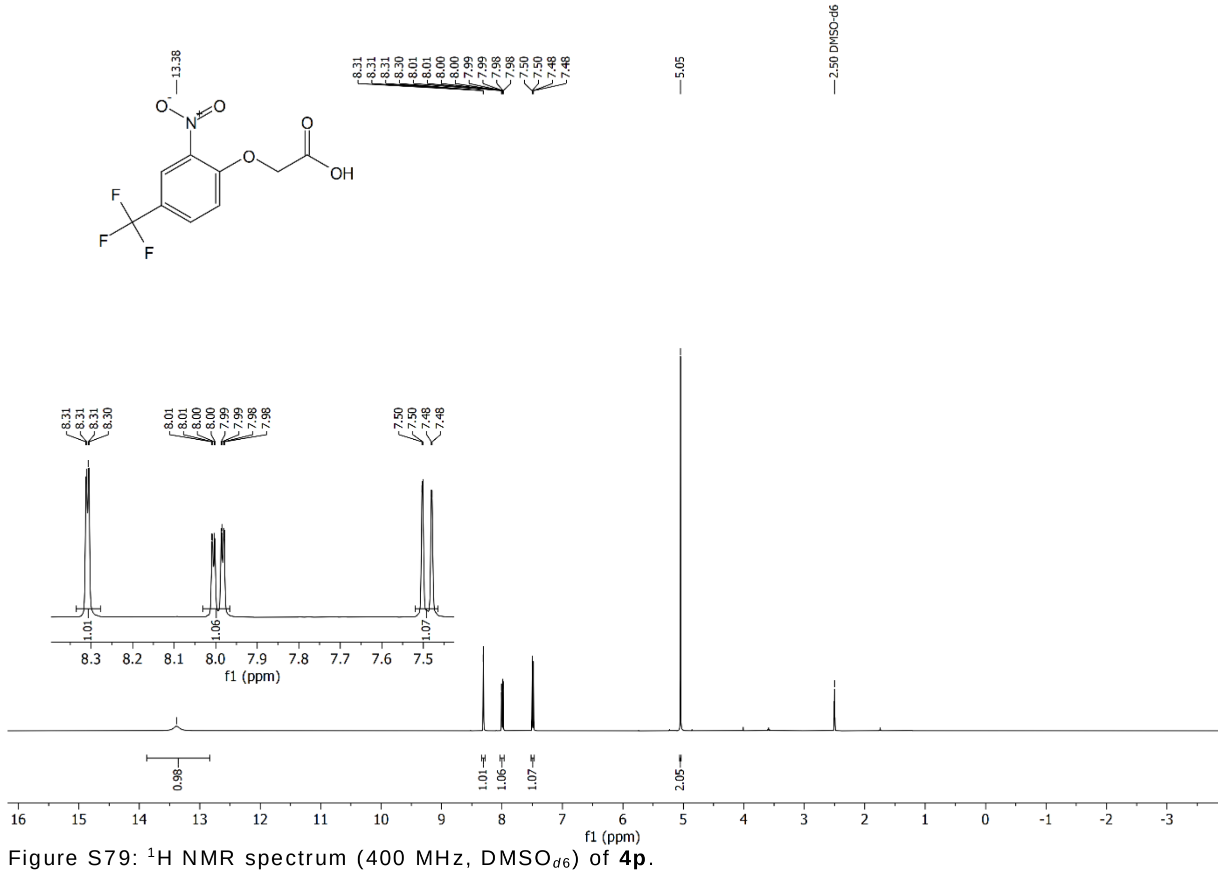

Figure S79: ^{1}H NMR spectrum (400 MHz, DMSO$_{d6}$) of **4p**.

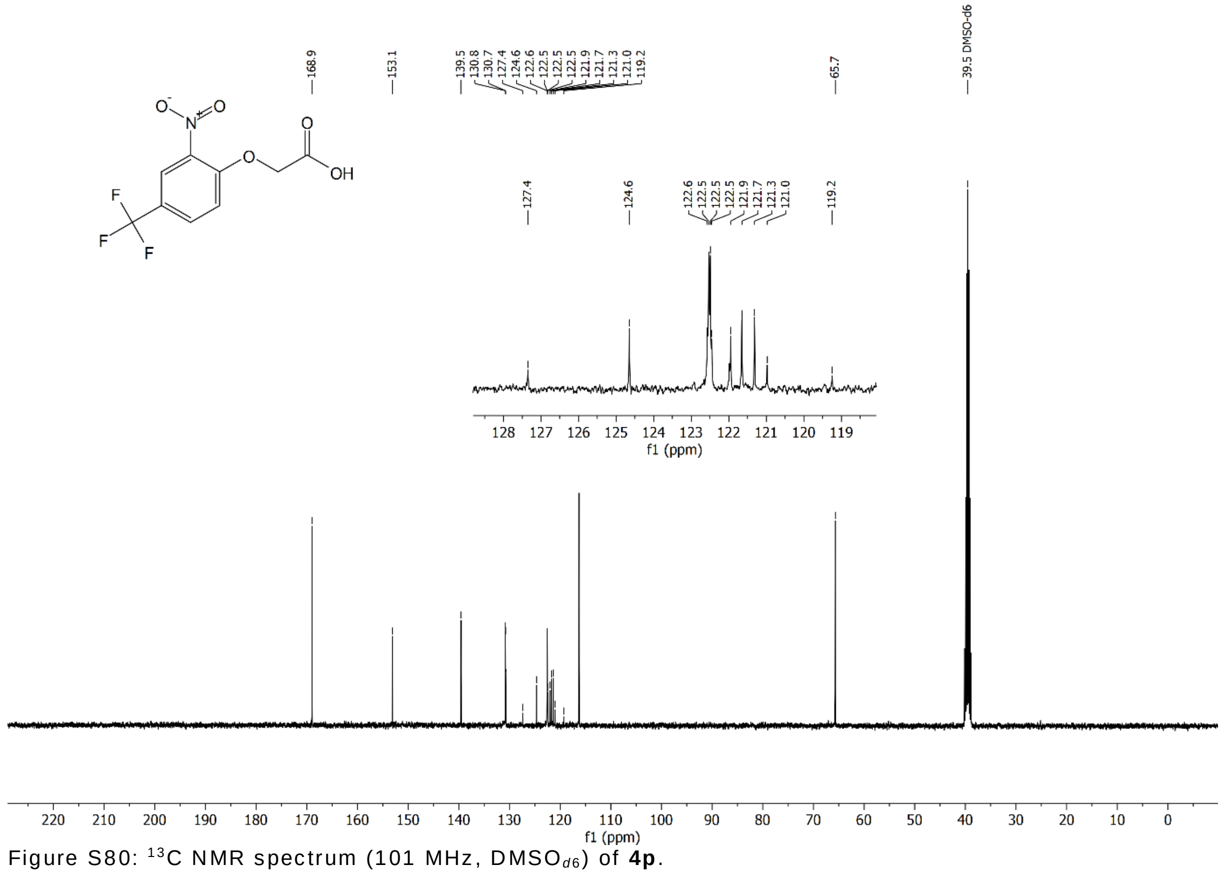

Figure S80: ^{13}C NMR spectrum (101 MHz, DMSO$_{d6}$) of **4p**.

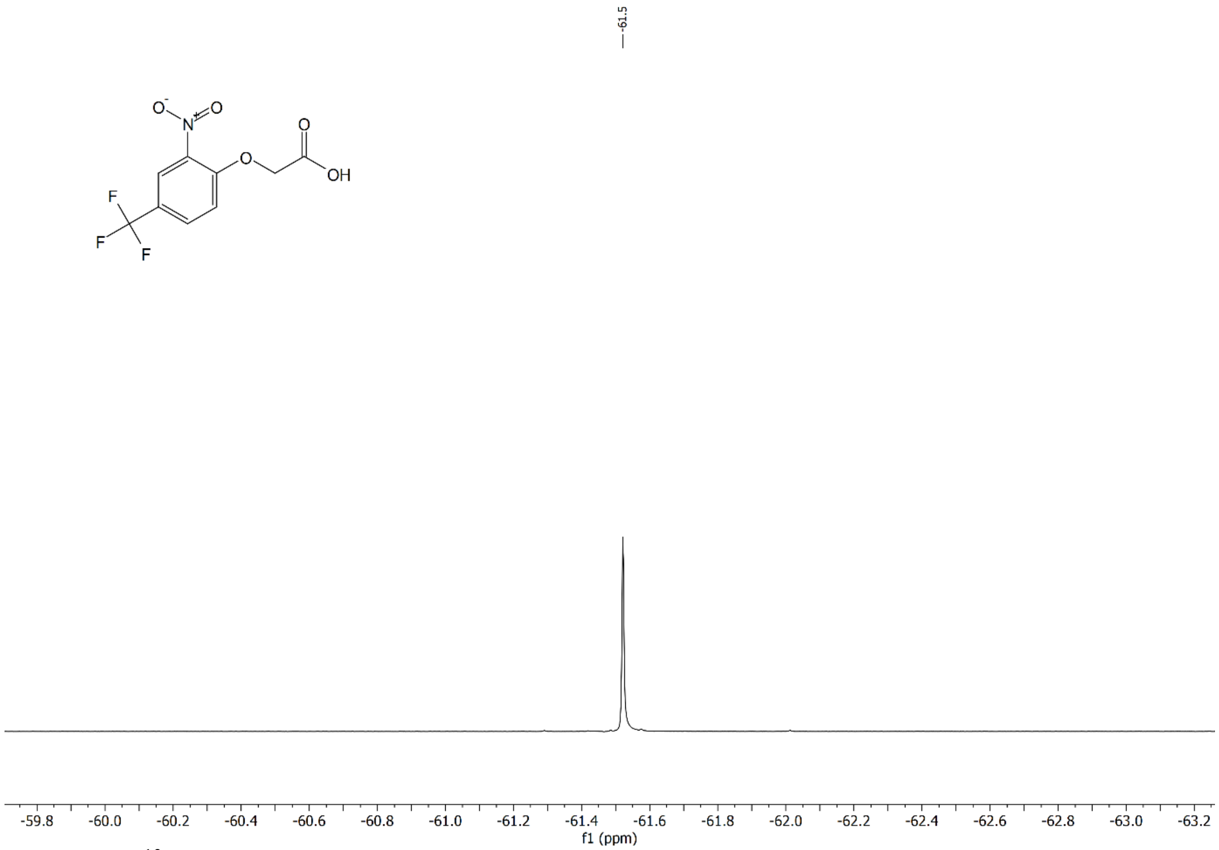

Figure S81: ^{19}F NMR spectrum (376 MHz, $DMSO_{d6}$) of **4p**.

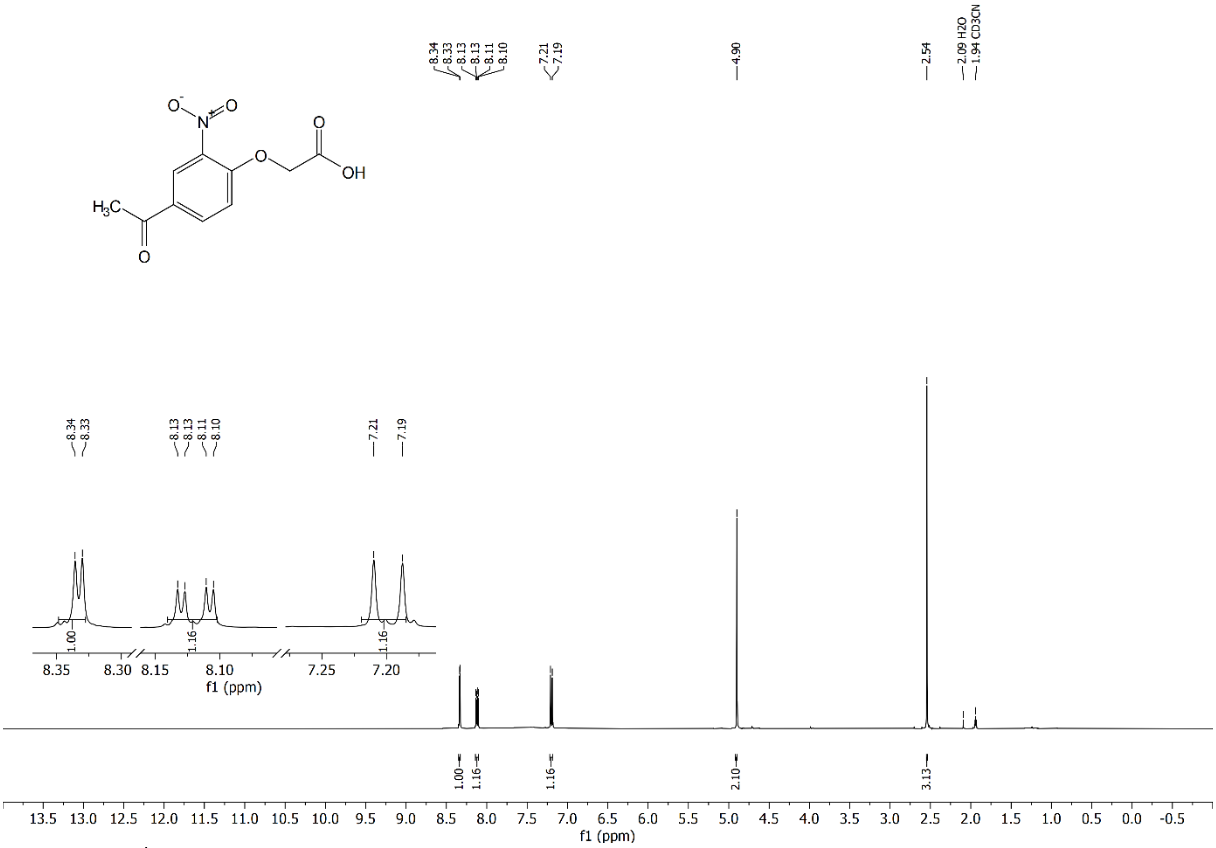

Figure S82: ^1H NMR spectrum (400 MHz, CD_3CN) of **4q**.

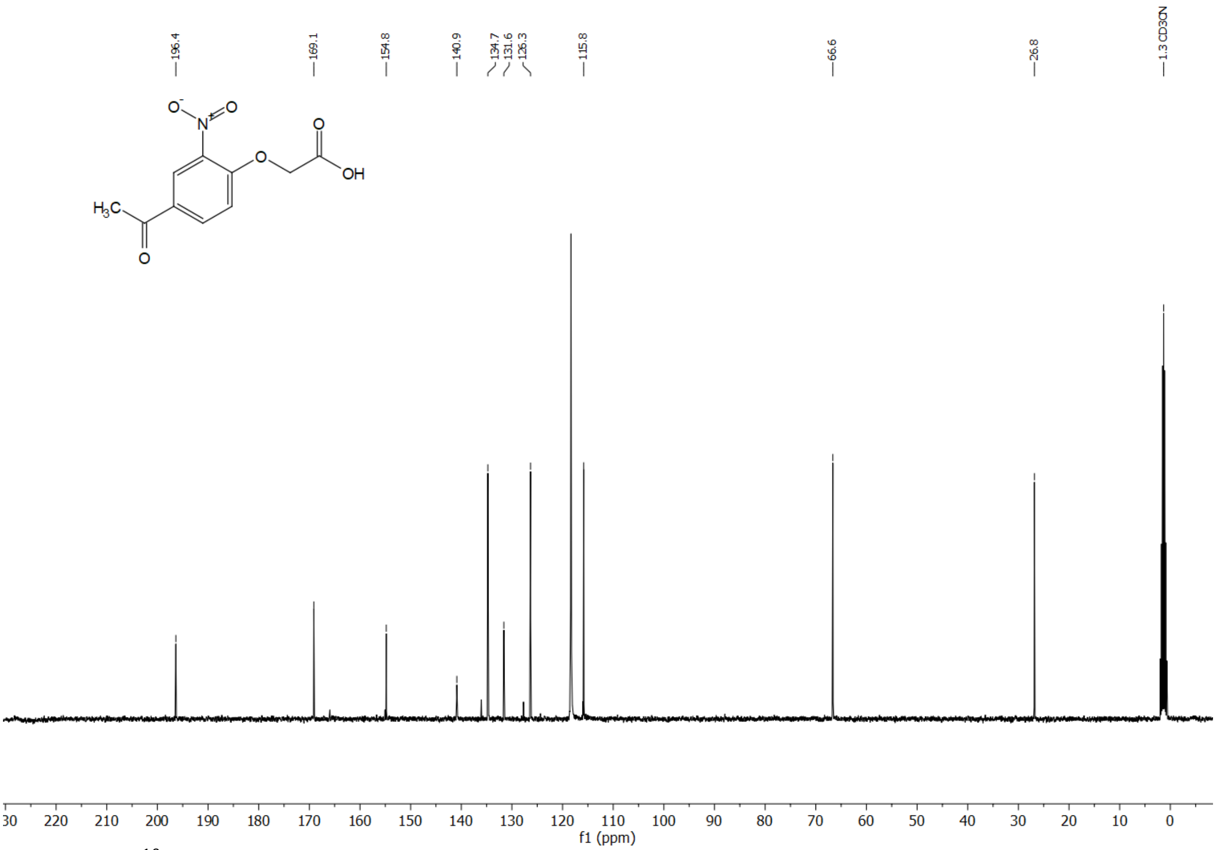

Figure S83: ^{13}C NMR spectrum (101 MHz, CD_3CN) of **4q**.

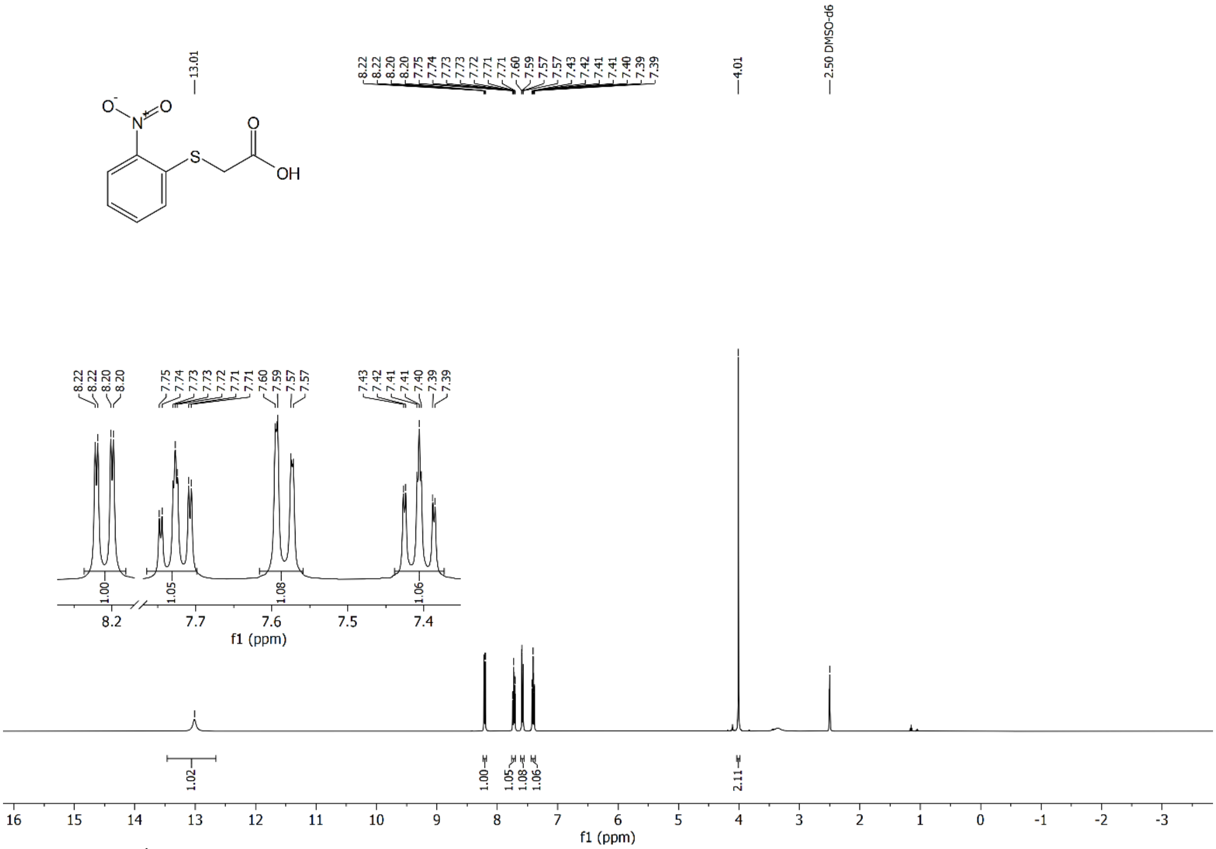

Figure S84: ^{1}H NMR spectrum (400 MHz, $DMSO_{d6}$) of **4r**.

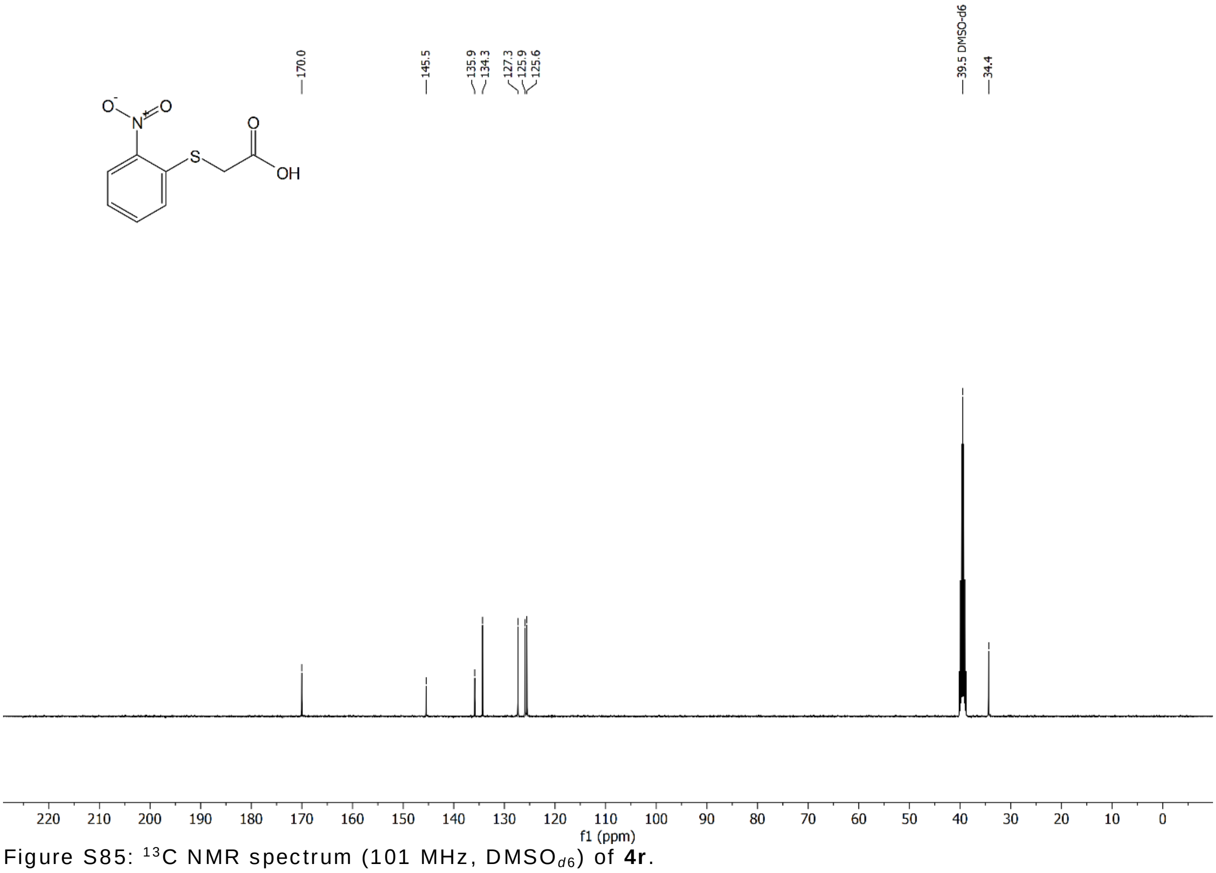

Figure S85: ^{13}C NMR spectrum (101 MHz, $DMSO_{d6}$) of **4r**.

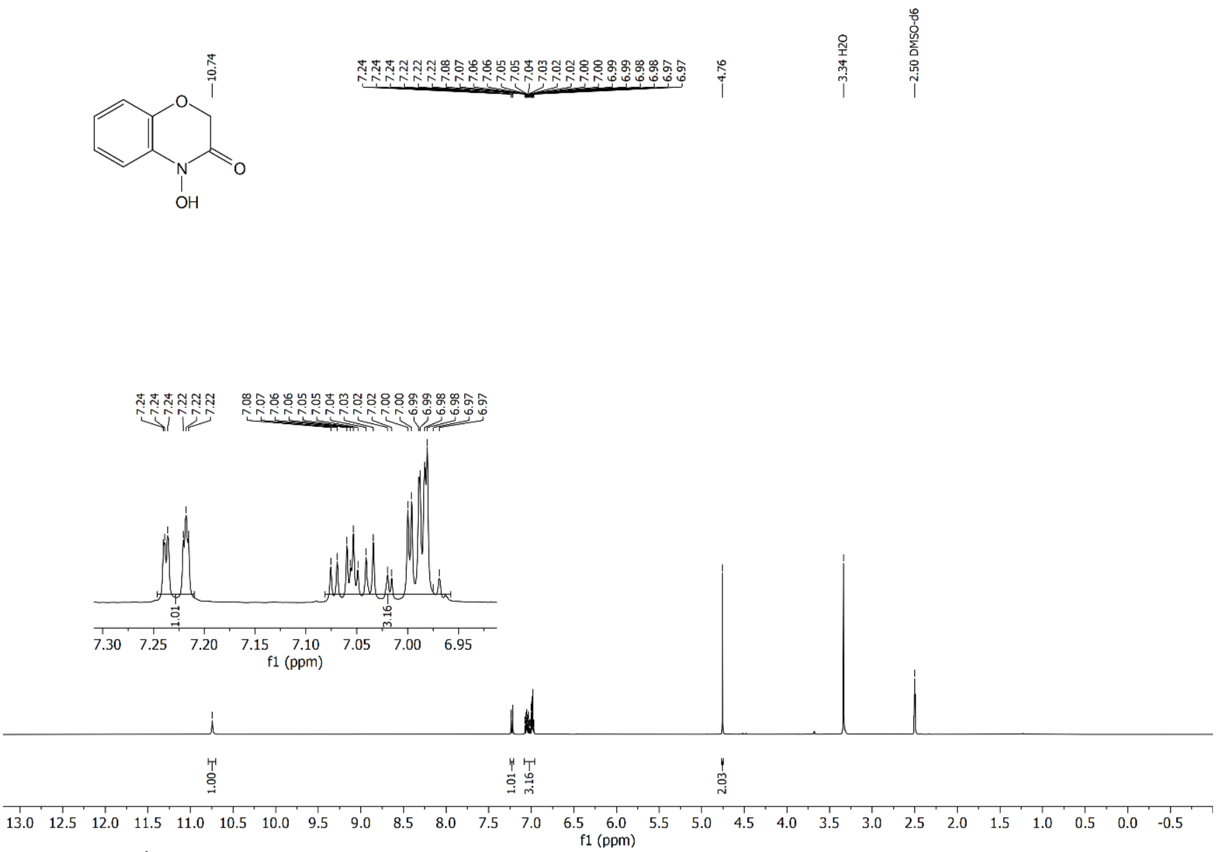

Figure S86: ^{1}H NMR spectrum (400 MHz, $DMSO_{d6}$) of **5a**.

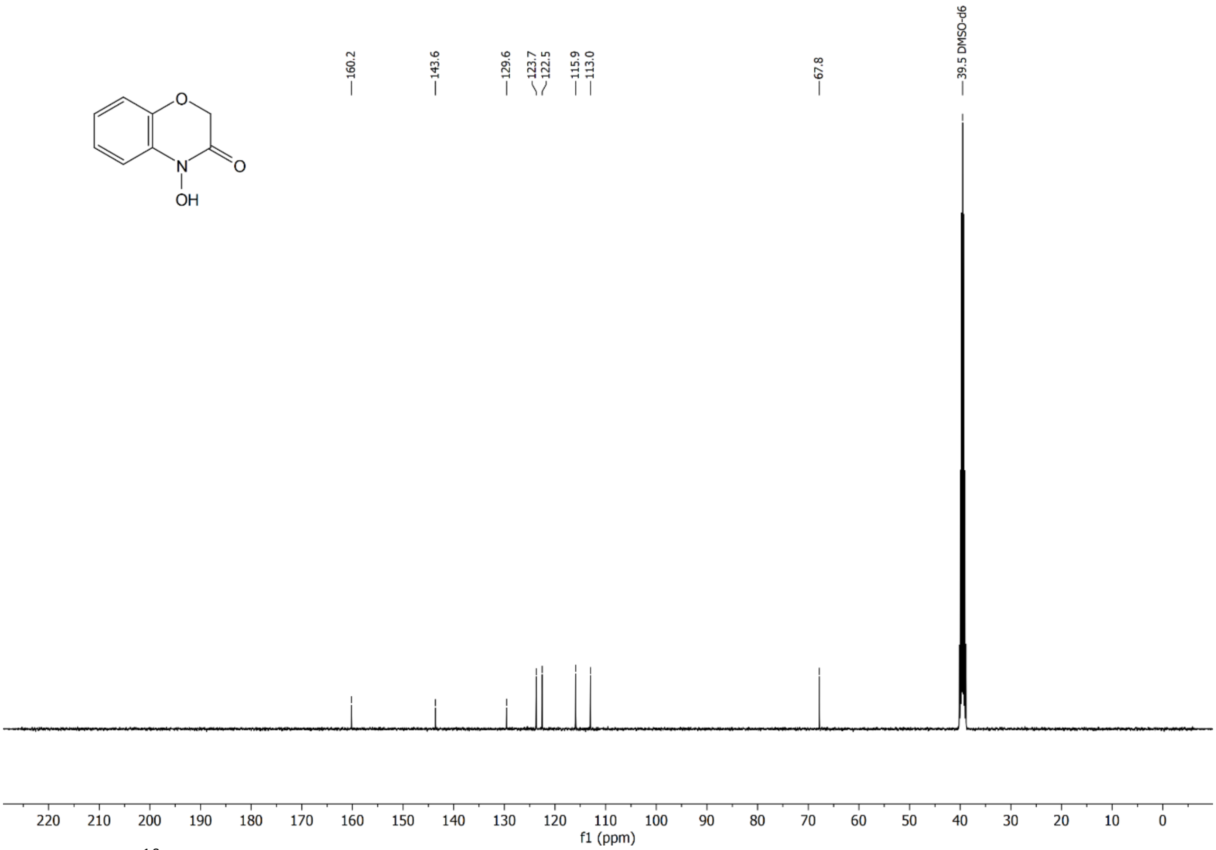

Figure S87: ^{13}C NMR spectrum (101 MHz, $DMSO_{d6}$) of **5a**.

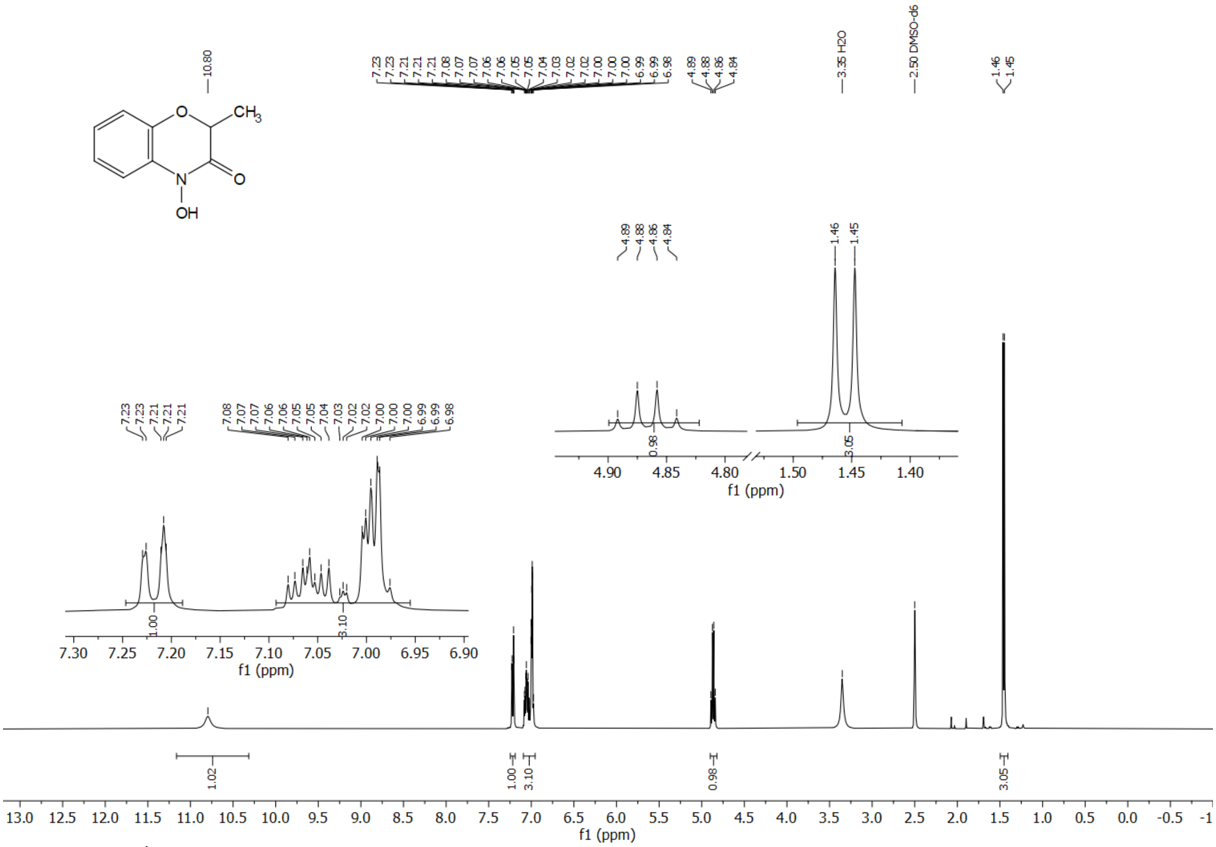

Figure S88: ^{1}H NMR spectrum (400 MHz, $DMSO_{d6}$) of **5b**.

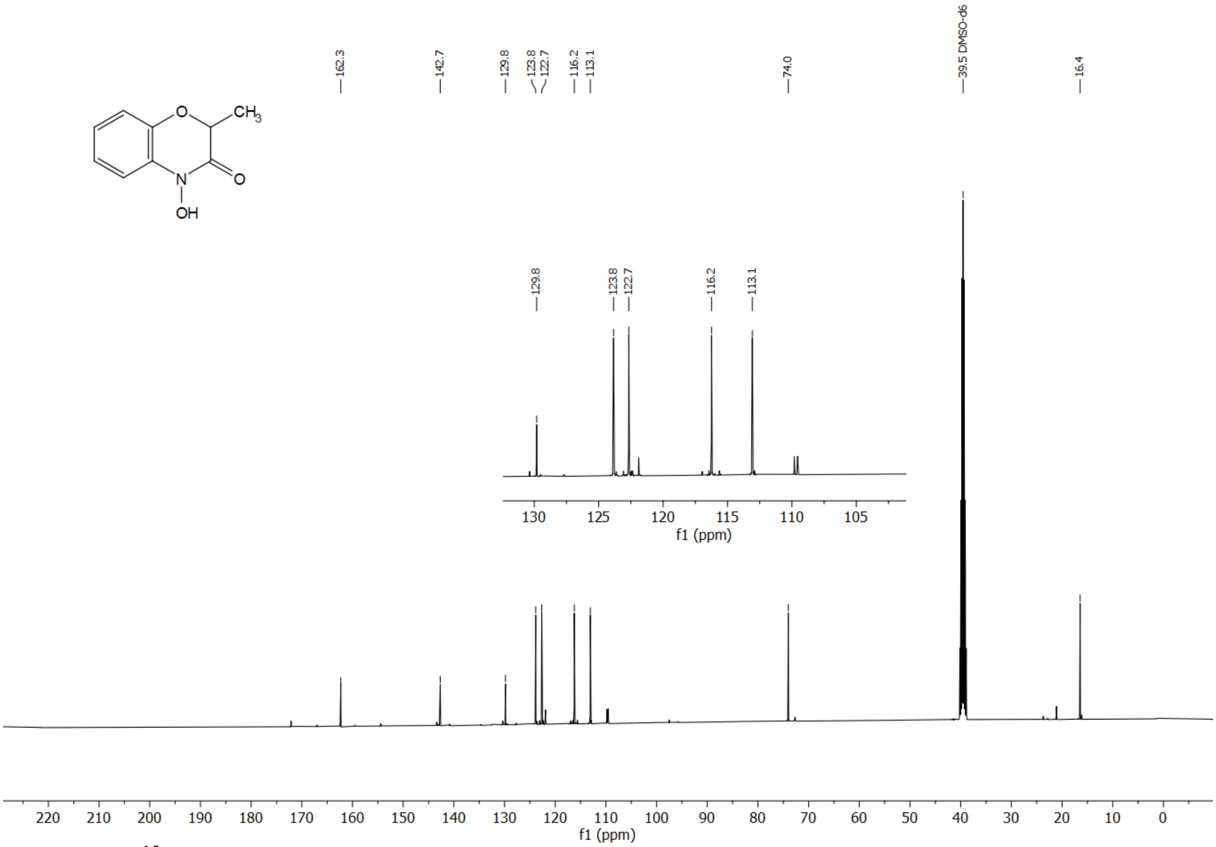

Figure S89: ^{13}C NMR spectrum (101 MHz, $DMSO_{d6}$) of **5b**.

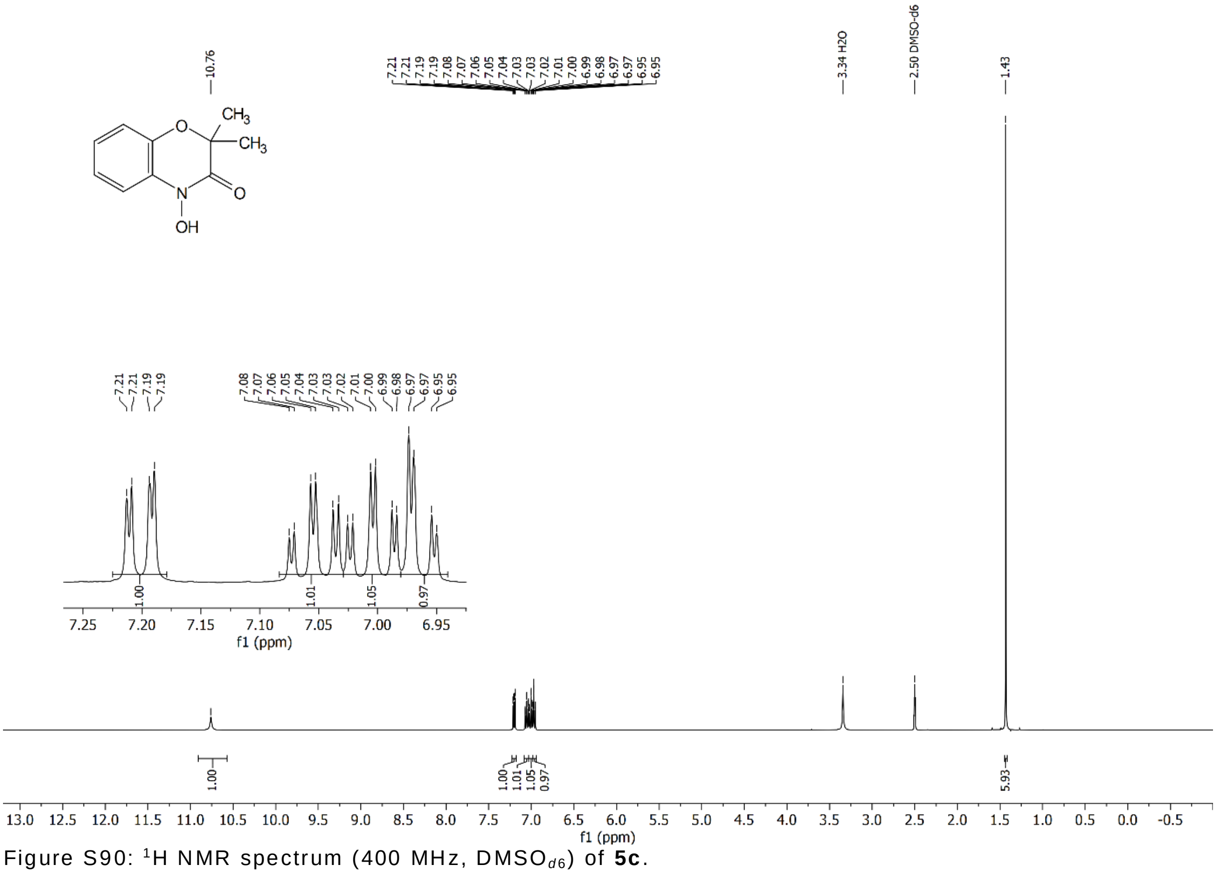

Figure S90: ^{1}H NMR spectrum (400 MHz, $DMSO_{d6}$) of **5c**.

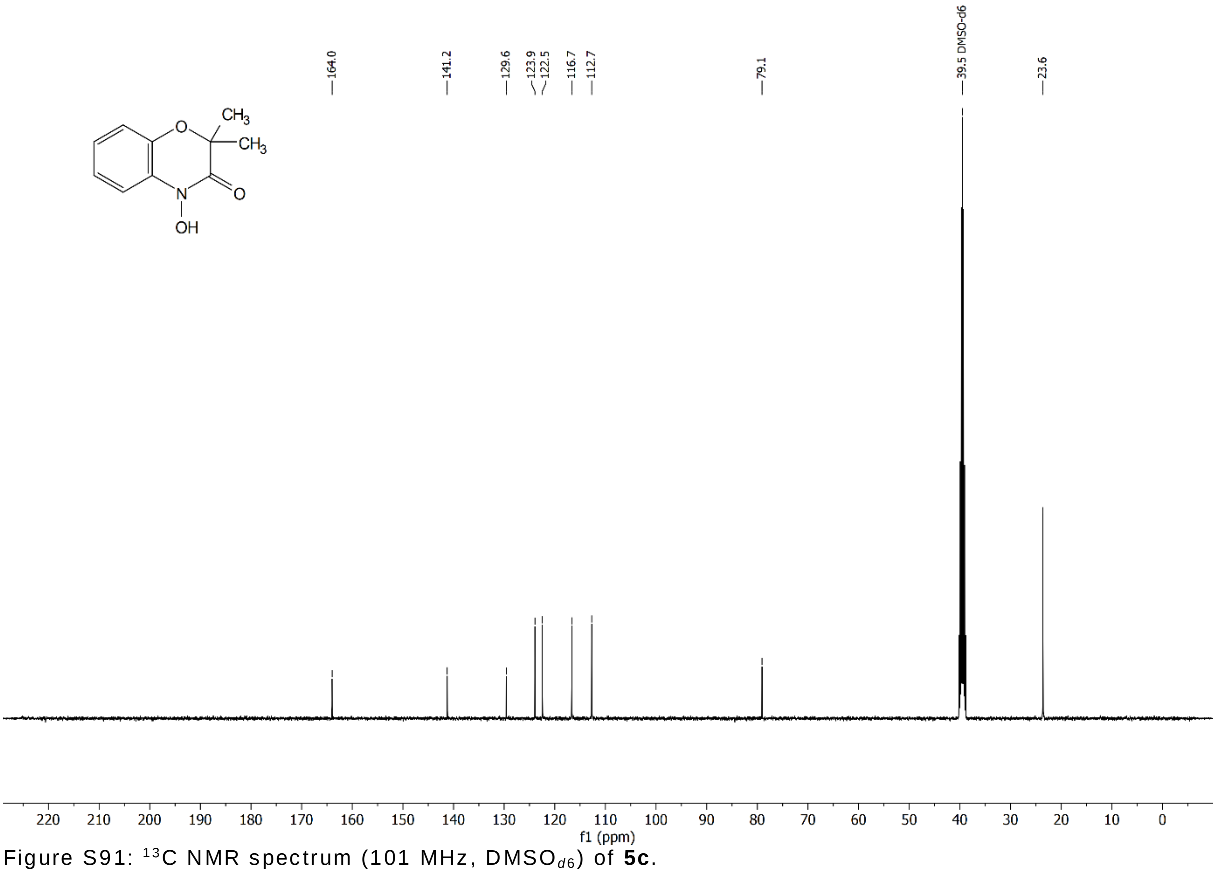

Figure S91: ^{13}C NMR spectrum (101 MHz, $DMSO_{d6}$) of **5c**.

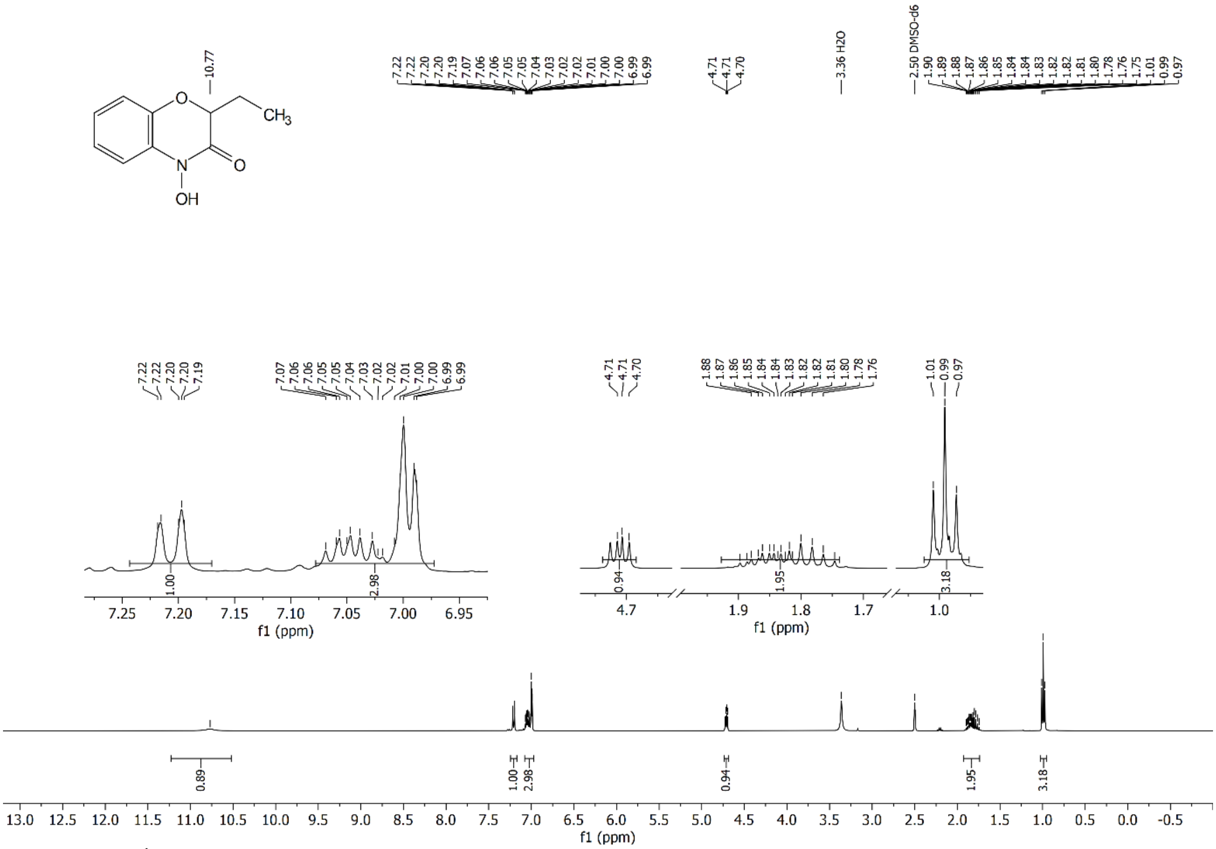

Figure S92: ^{1}H NMR spectrum (400 MHz, $DMSO_{d6}$) of **5d**.

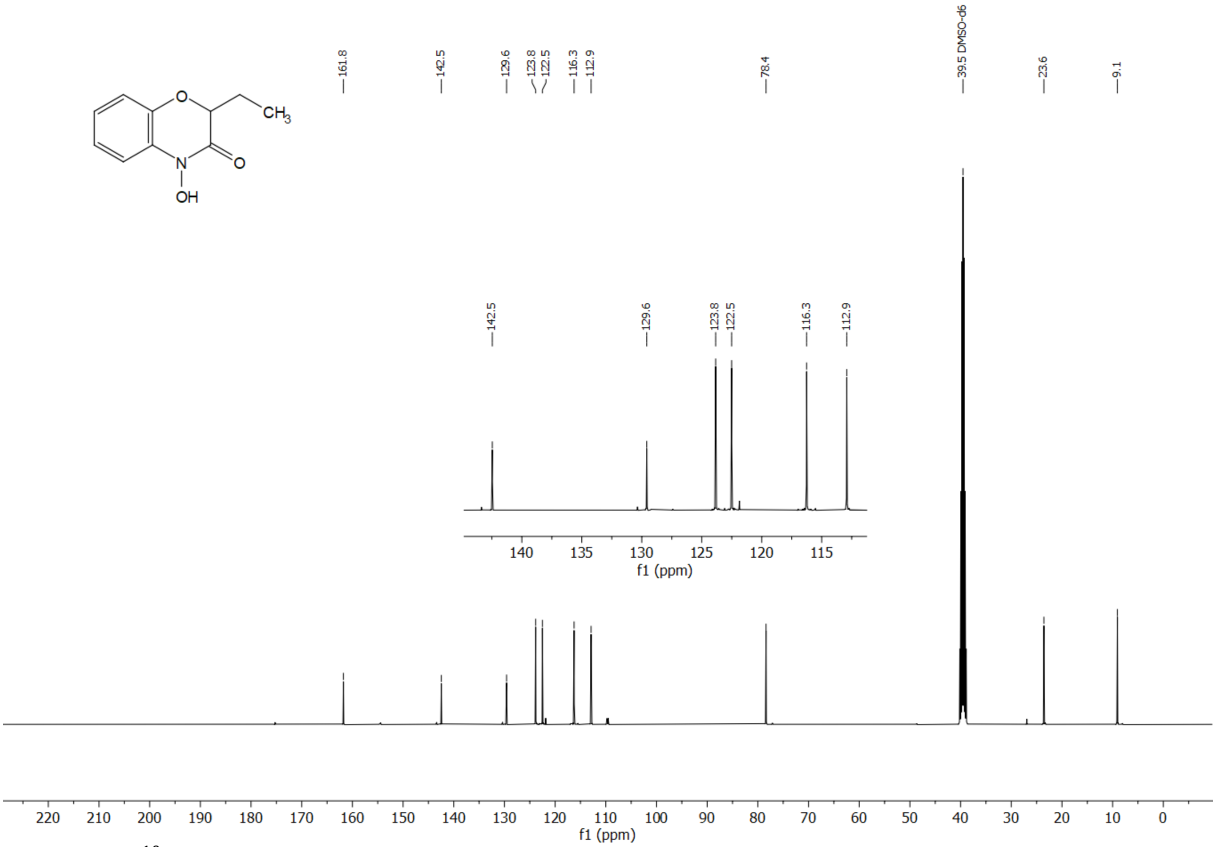

Figure S93: ^{13}C NMR spectrum (101 MHz, $DMSO_{d6}$) of **5d**.

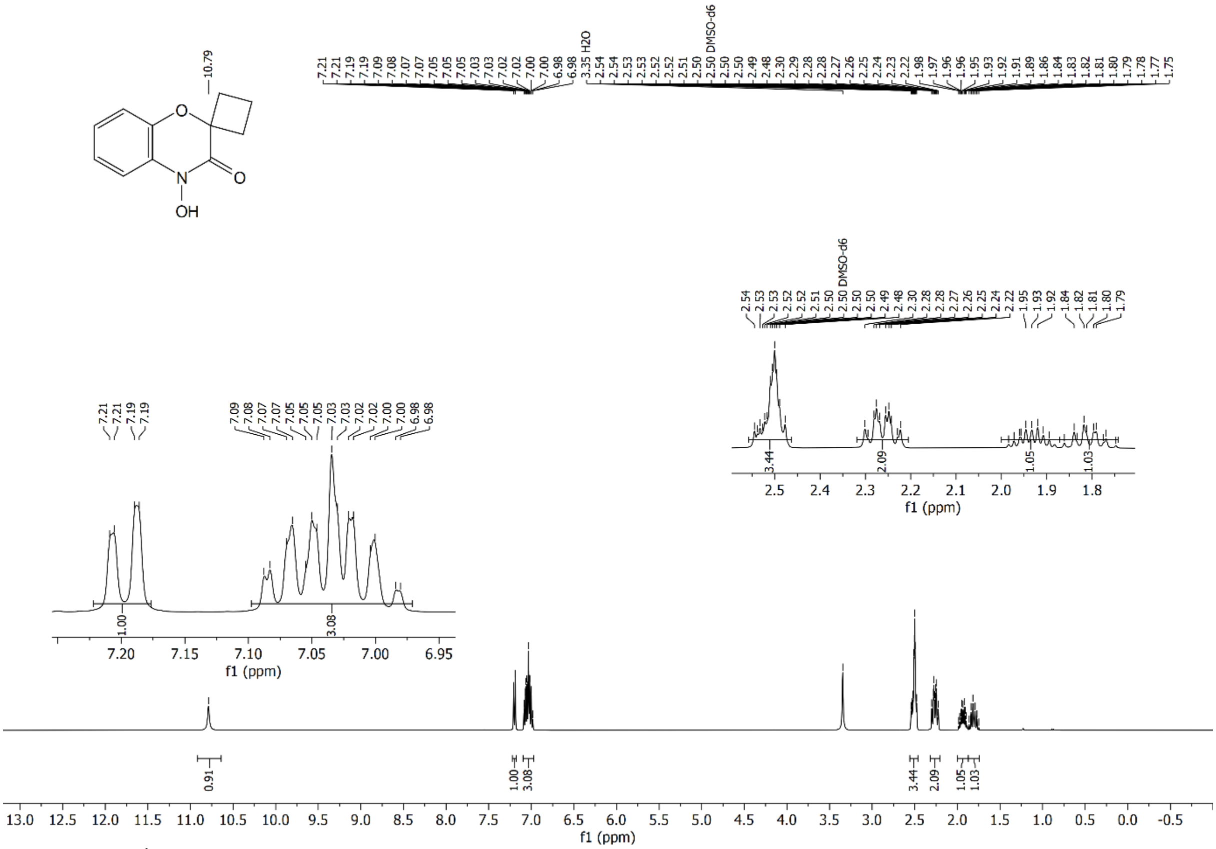

Figure S94: ^{1}H NMR spectrum (400 MHz, DMSO$_{d6}$) of **5e**.

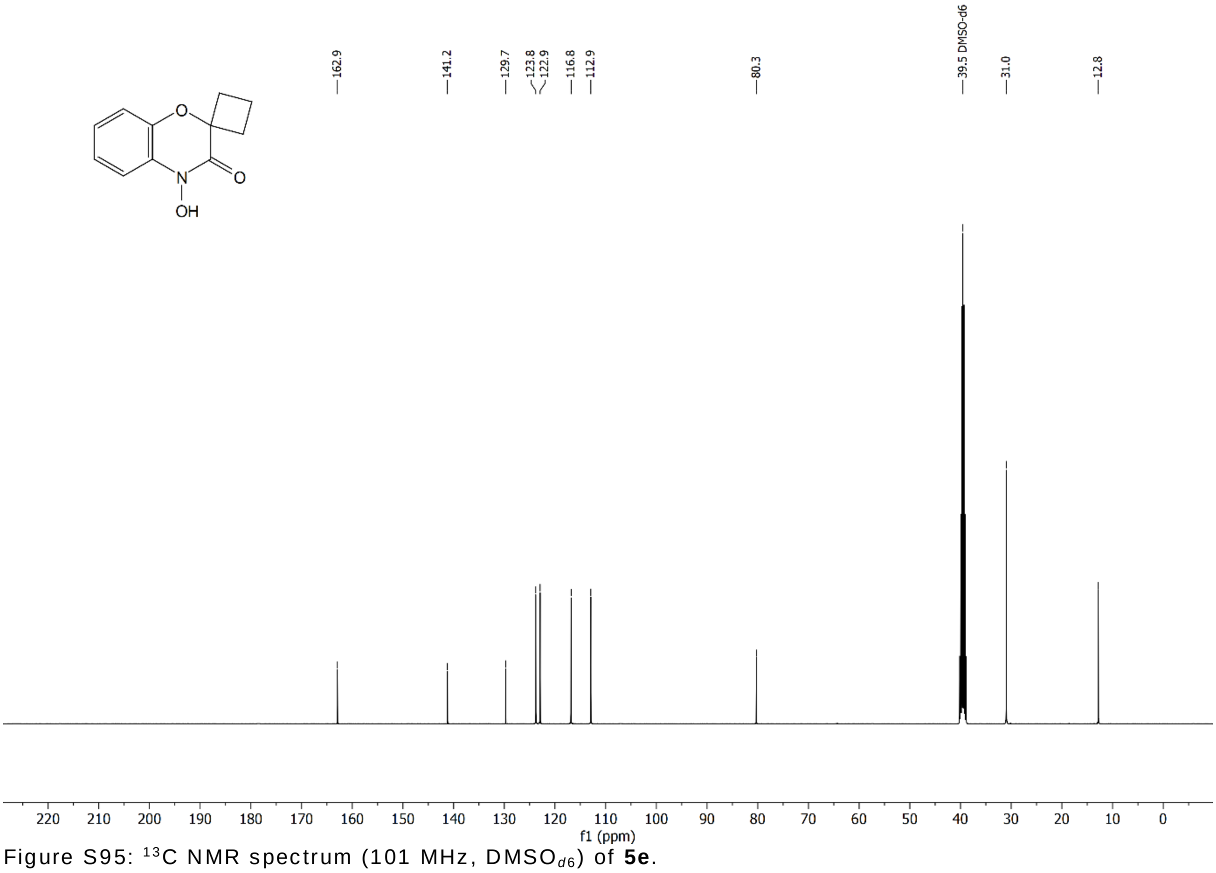

Figure S95: ^{13}C NMR spectrum (101 MHz, DMSO$_{d6}$) of **5e**.

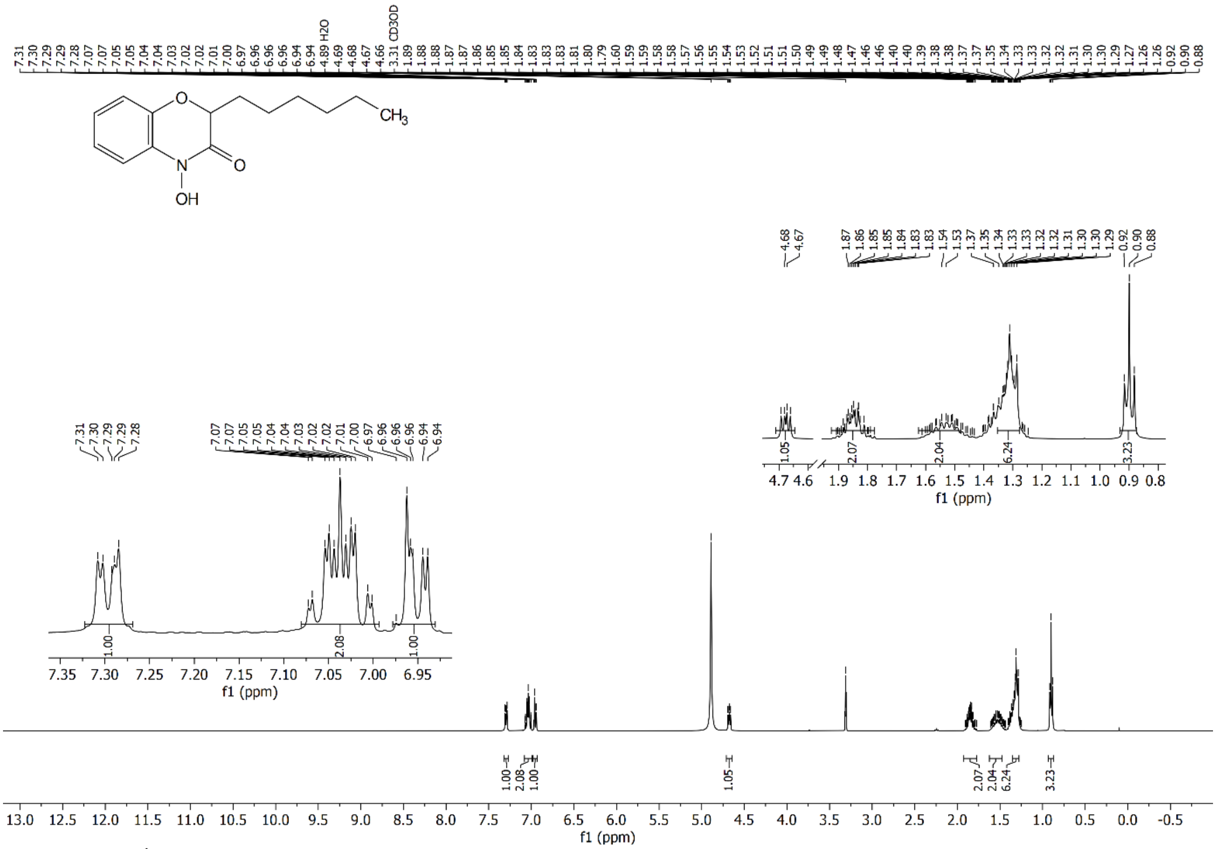

Figure S96: ^{1}H NMR spectrum (400 MHz, CD_3OD) of **5f**.

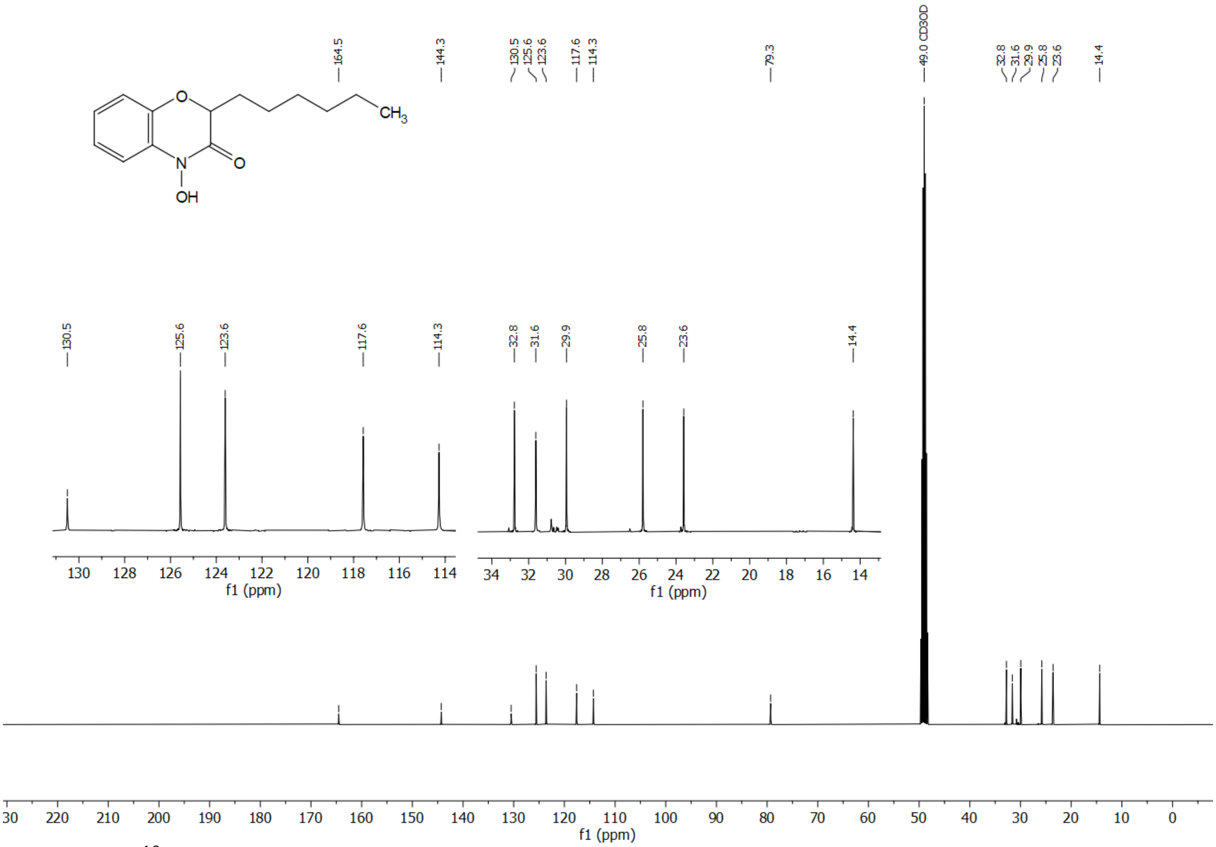

Figure S97: ^{13}C NMR spectrum (101 MHz, CD_3OD) of **5f**.

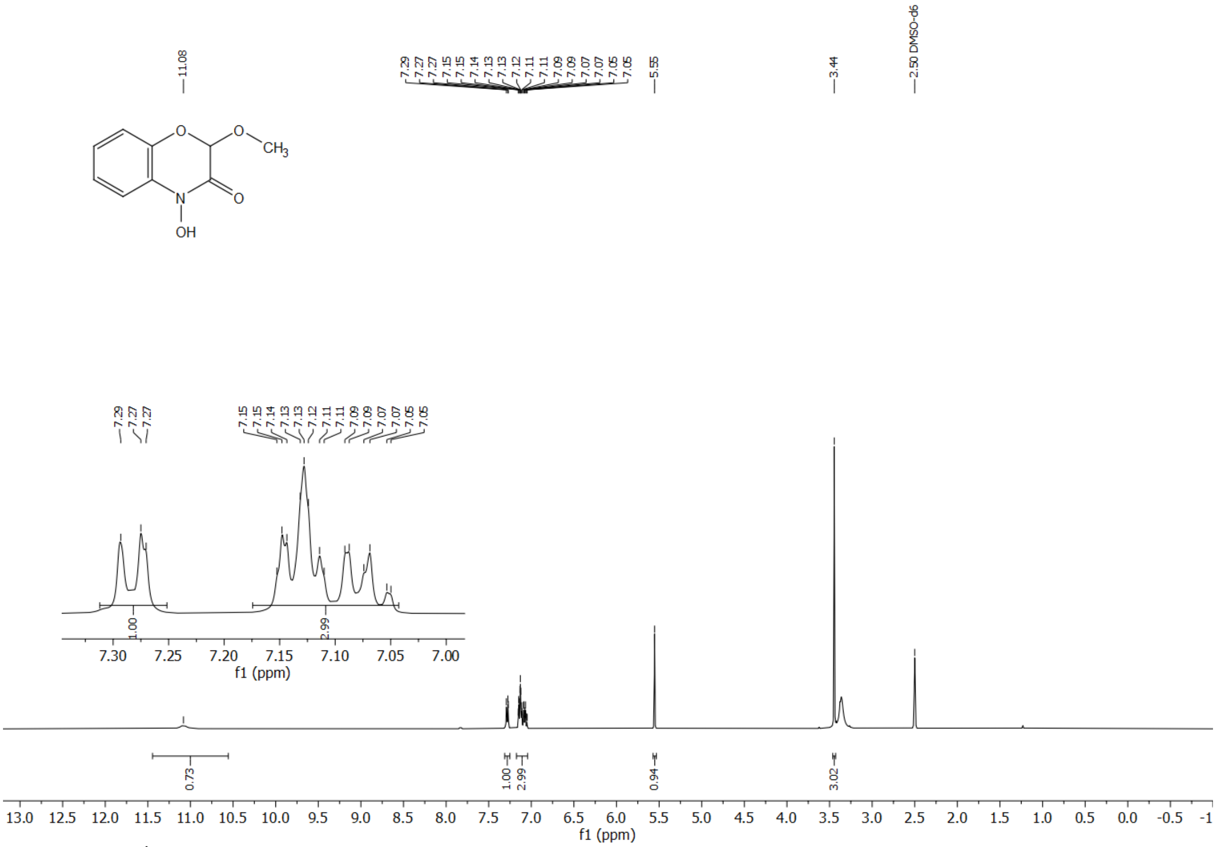

Figure S98: ¹H NMR spectrum (400 MHz, $DMSO_{d6}$) of **5g**.

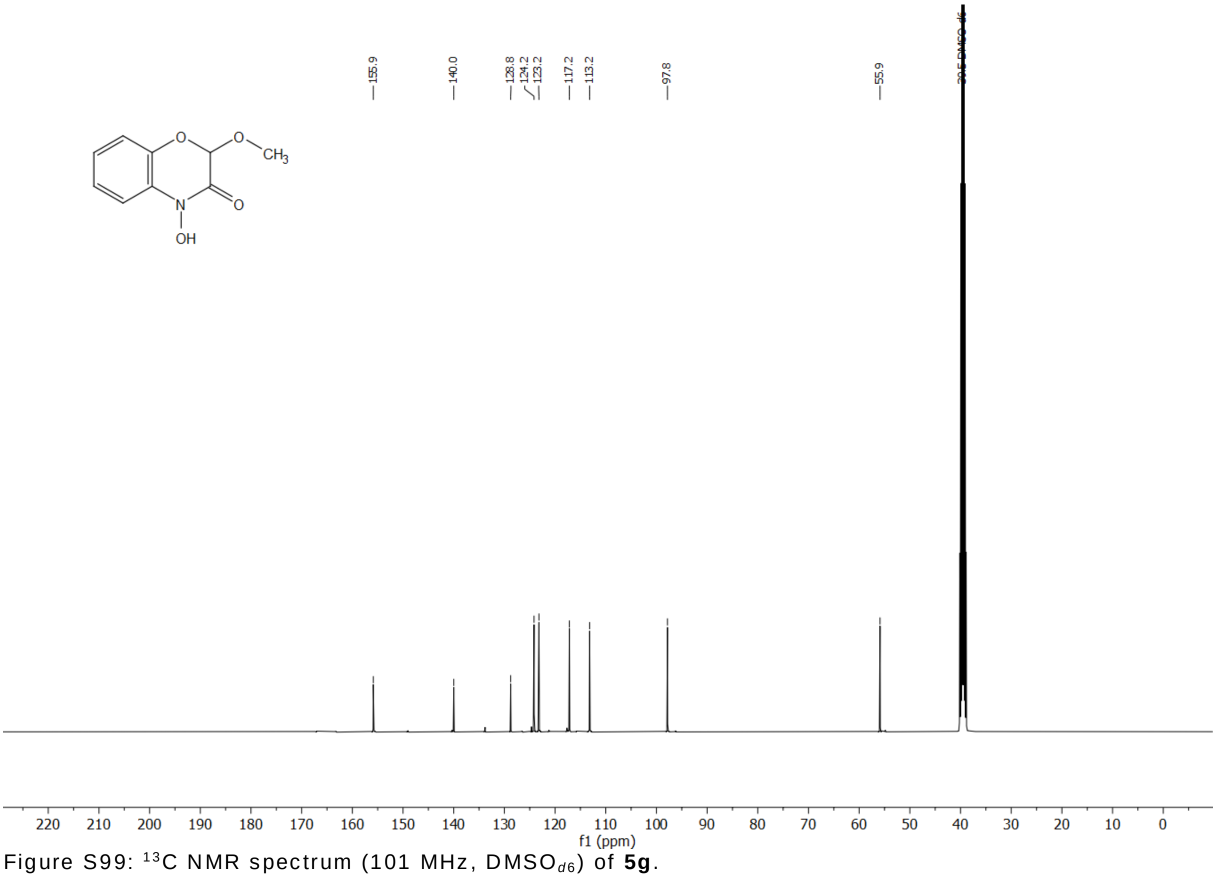

Figure S99: ¹³C NMR spectrum (101 MHz, $DMSO_{d6}$) of **5g**.

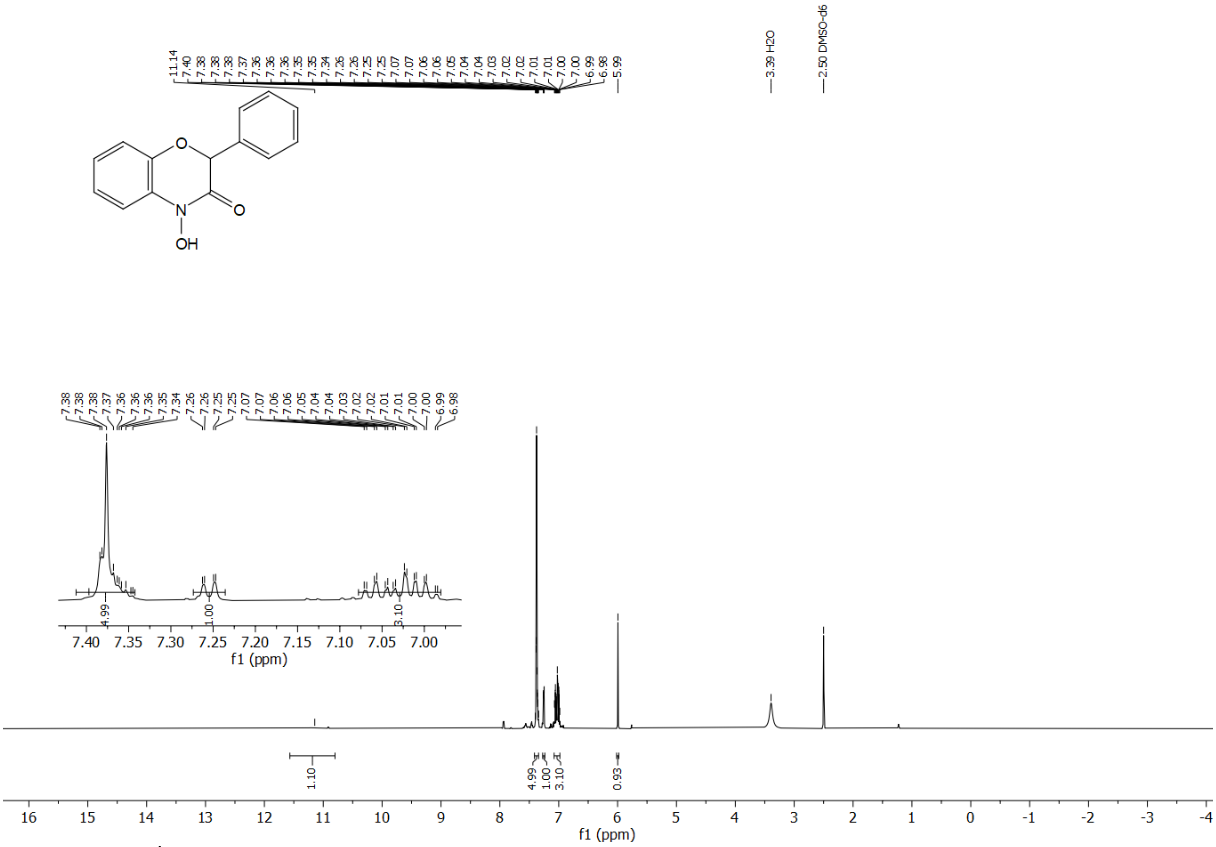

Figure S100: ^{1}H NMR spectrum (400 MHz, $DMSO_{d6}$) of **5h**.

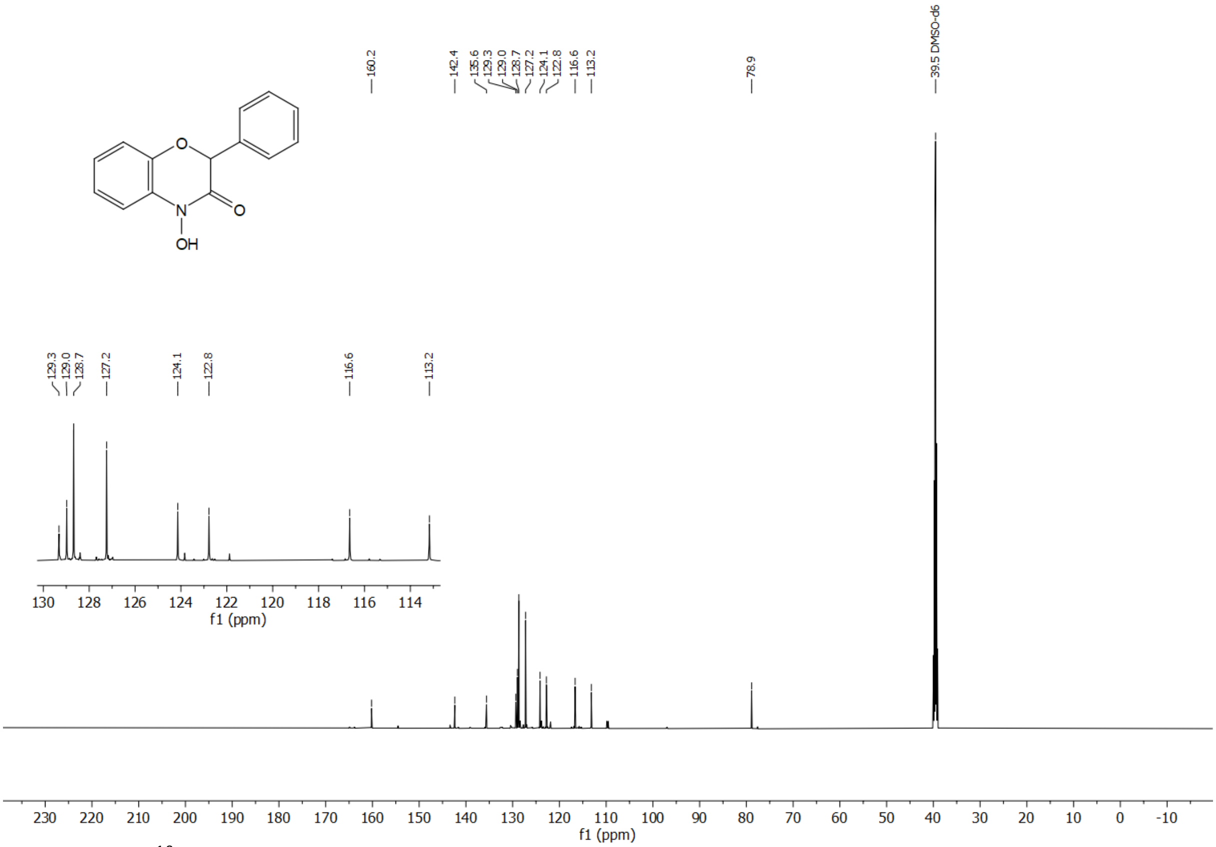

Figure S101: ^{13}C NMR spectrum (101 MHz, $DMSO_{d6}$) of **5h**.

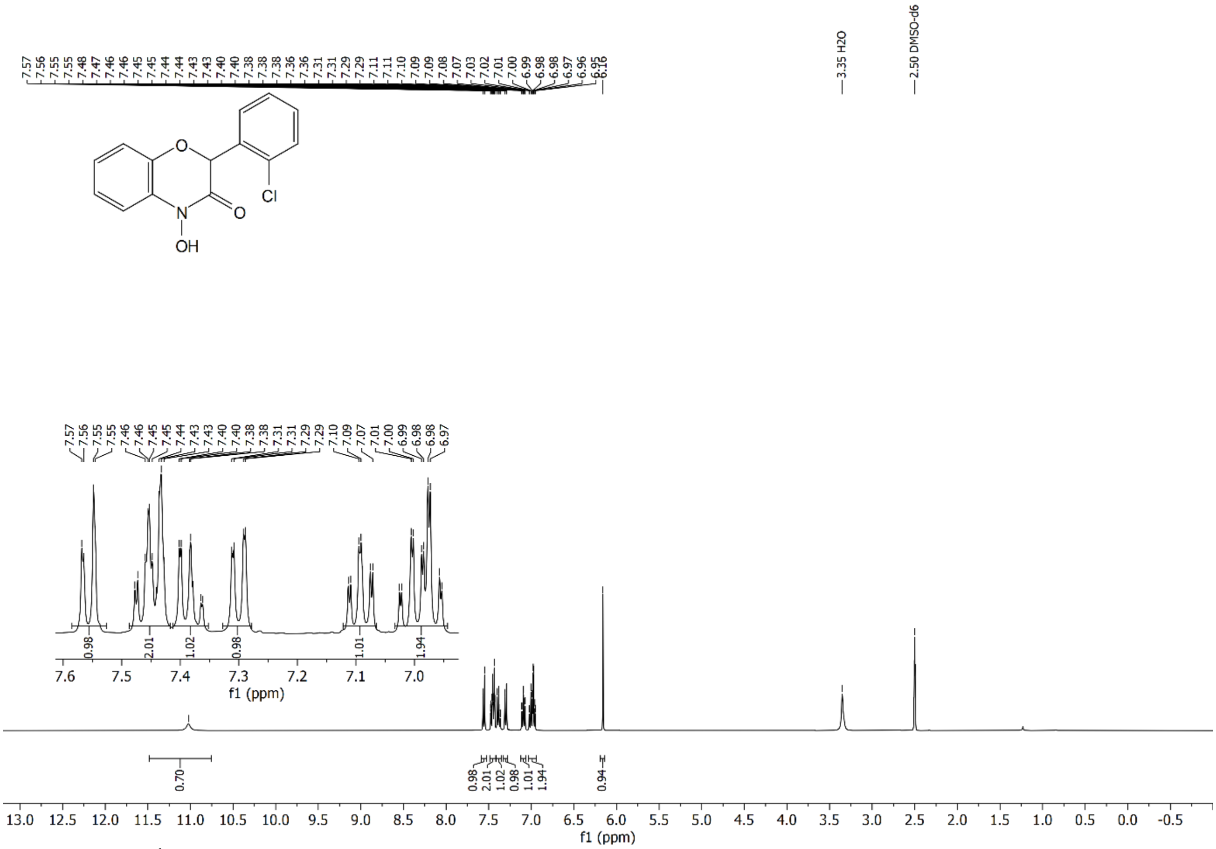

Figure S102: ^{1}H NMR spectrum (400 MHz, $DMSO_{d6}$) of **5i**.

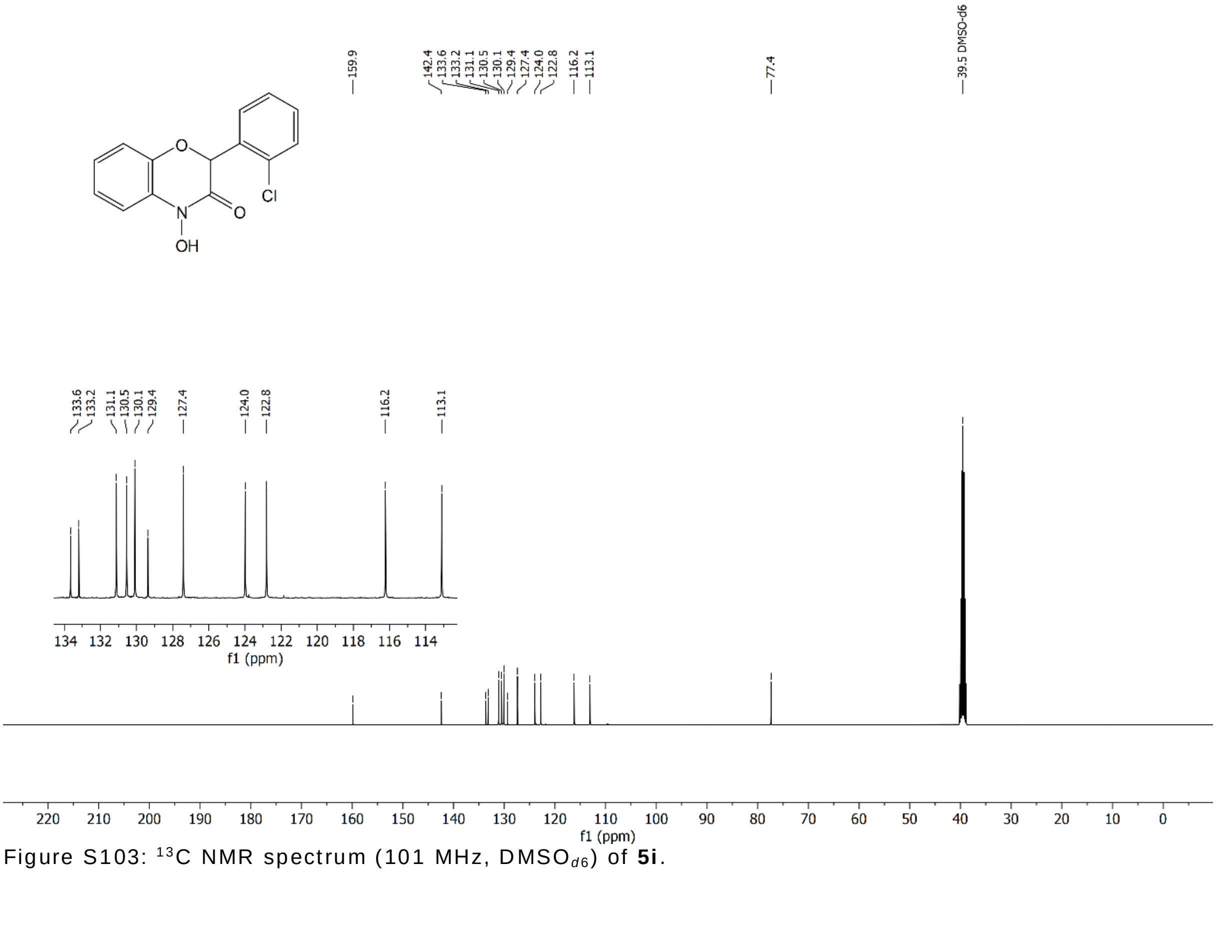

Figure S103: ^{13}C NMR spectrum (101 MHz, $DMSO_{d6}$) of **5i**.

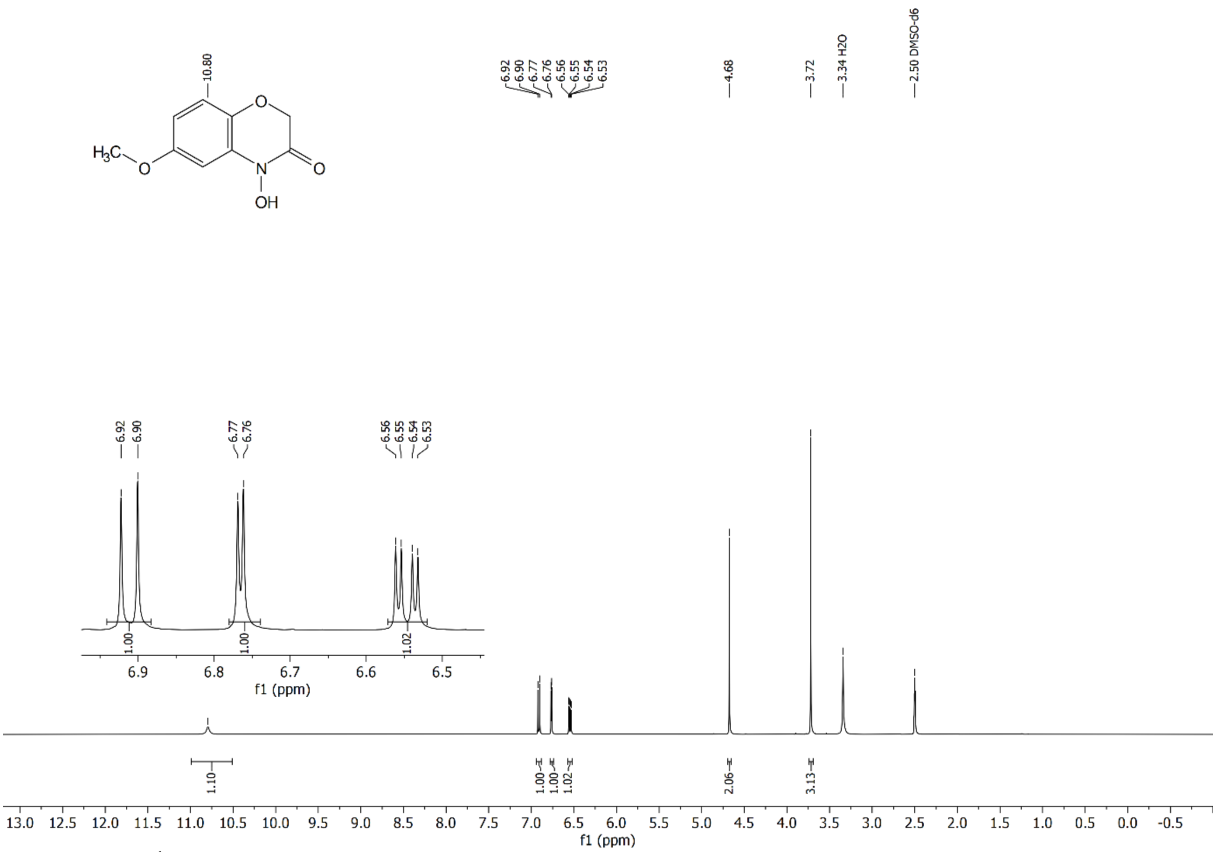

Figure S104: ^{1}H NMR spectrum (400 MHz, DMSO$_{d6}$) of **5j**.

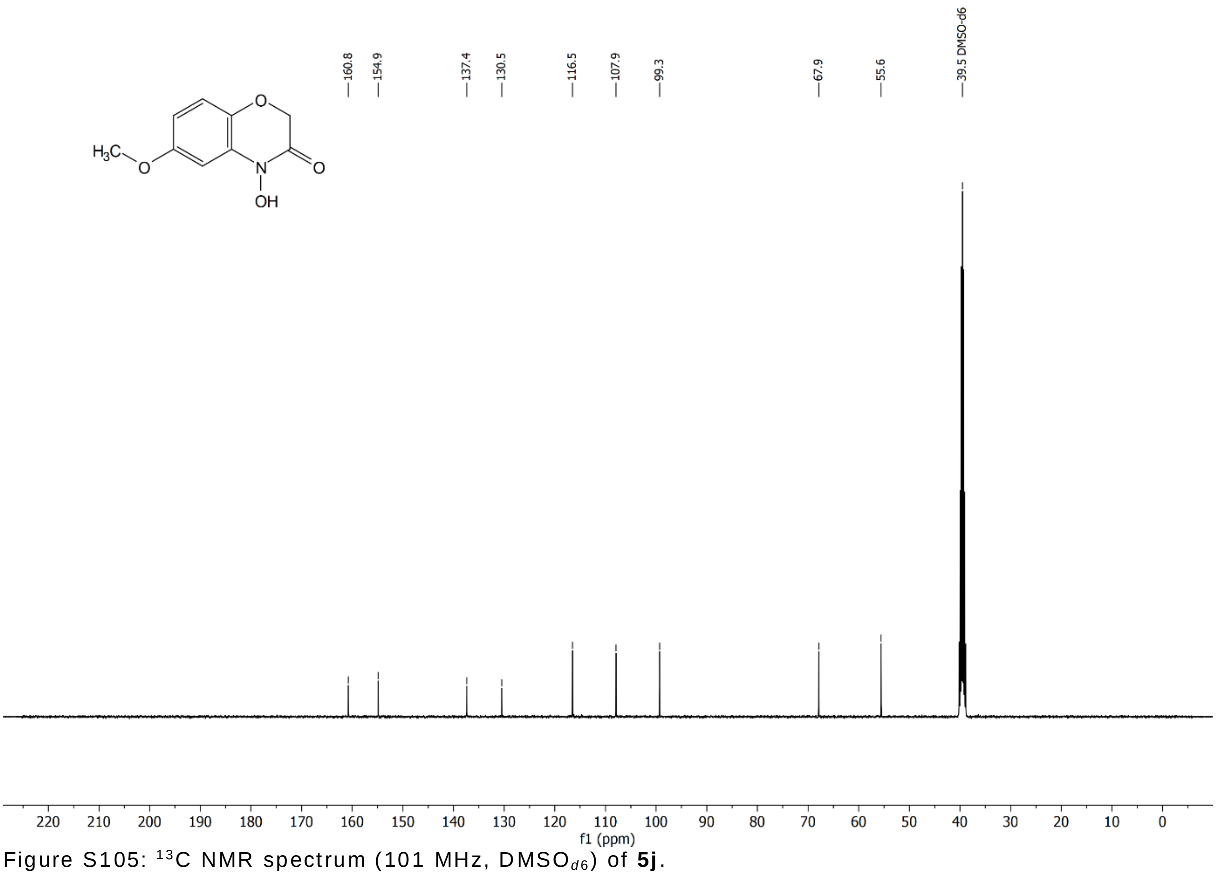

Figure S105: ^{13}C NMR spectrum (101 MHz, DMSO$_{d6}$) of **5j**.

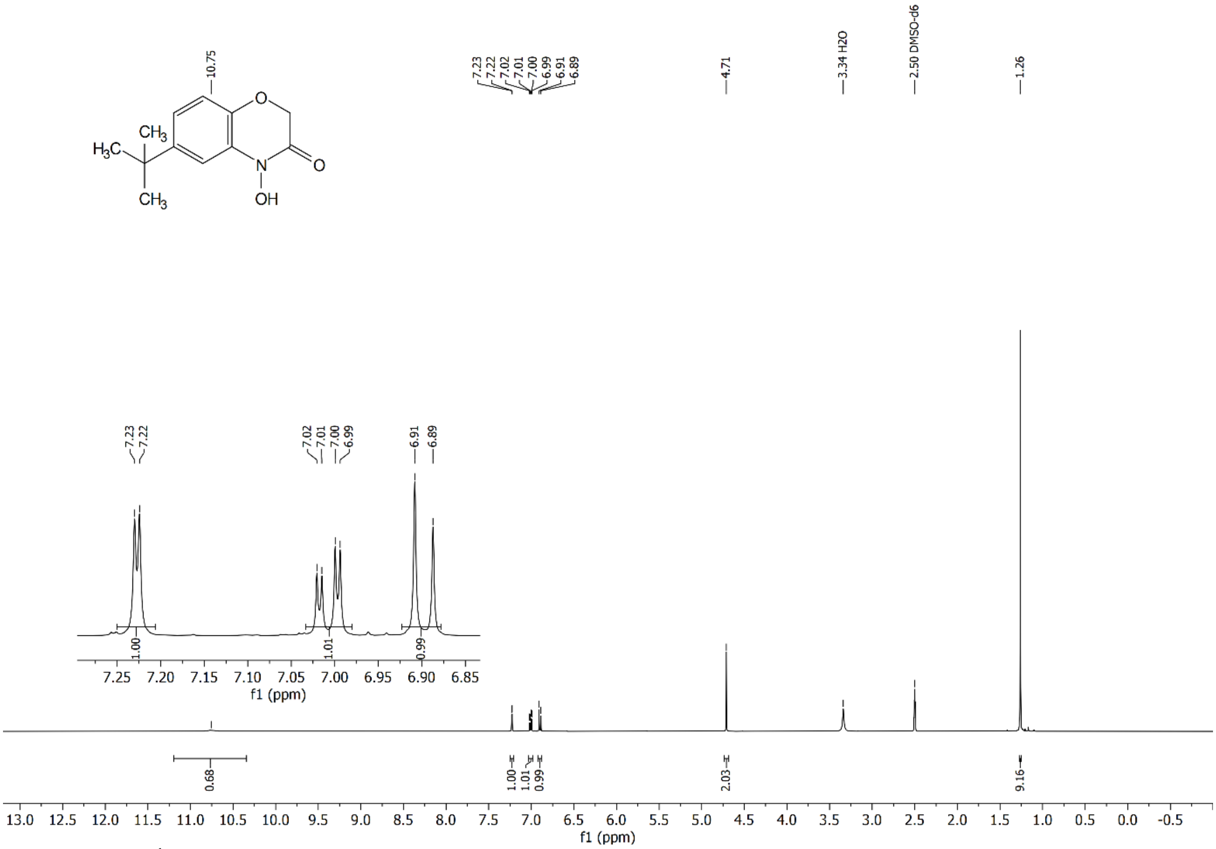

Figure S106: ^{1}H NMR spectrum (400 MHz, DMSO$_{d6}$) of **5k**.

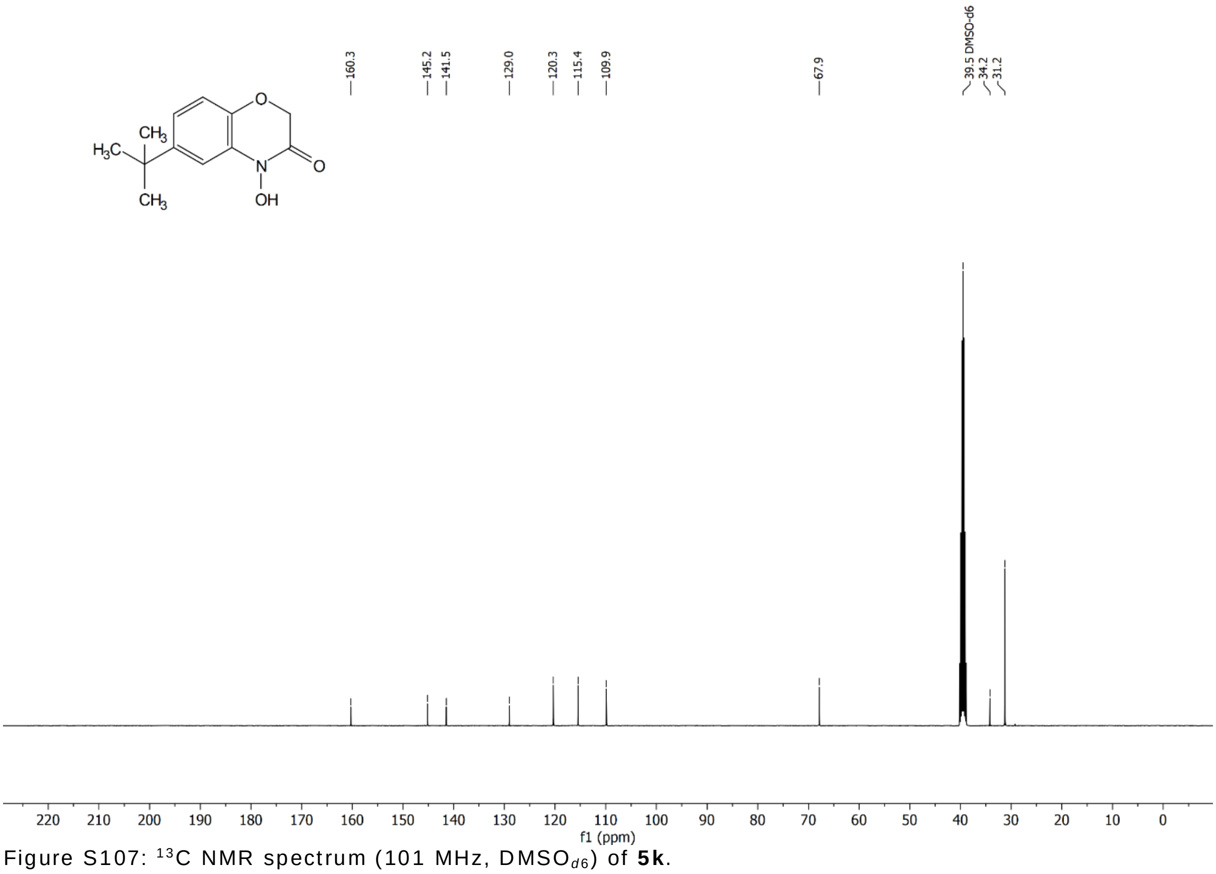

Figure S107: ^{13}C NMR spectrum (101 MHz, DMSO$_{d6}$) of **5k**.

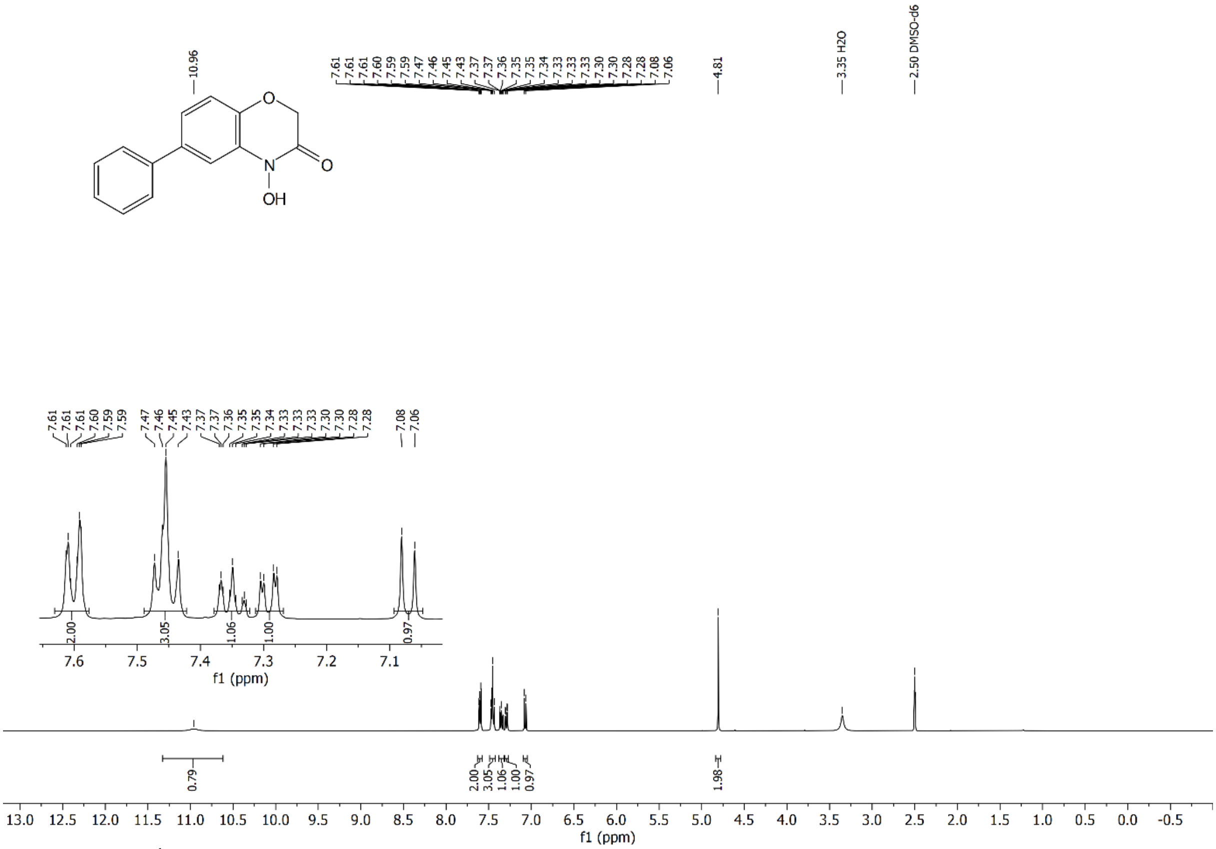

Figure S108: ^{1}H NMR spectrum (400 MHz, $DMSO_{d6}$) of **5l**.

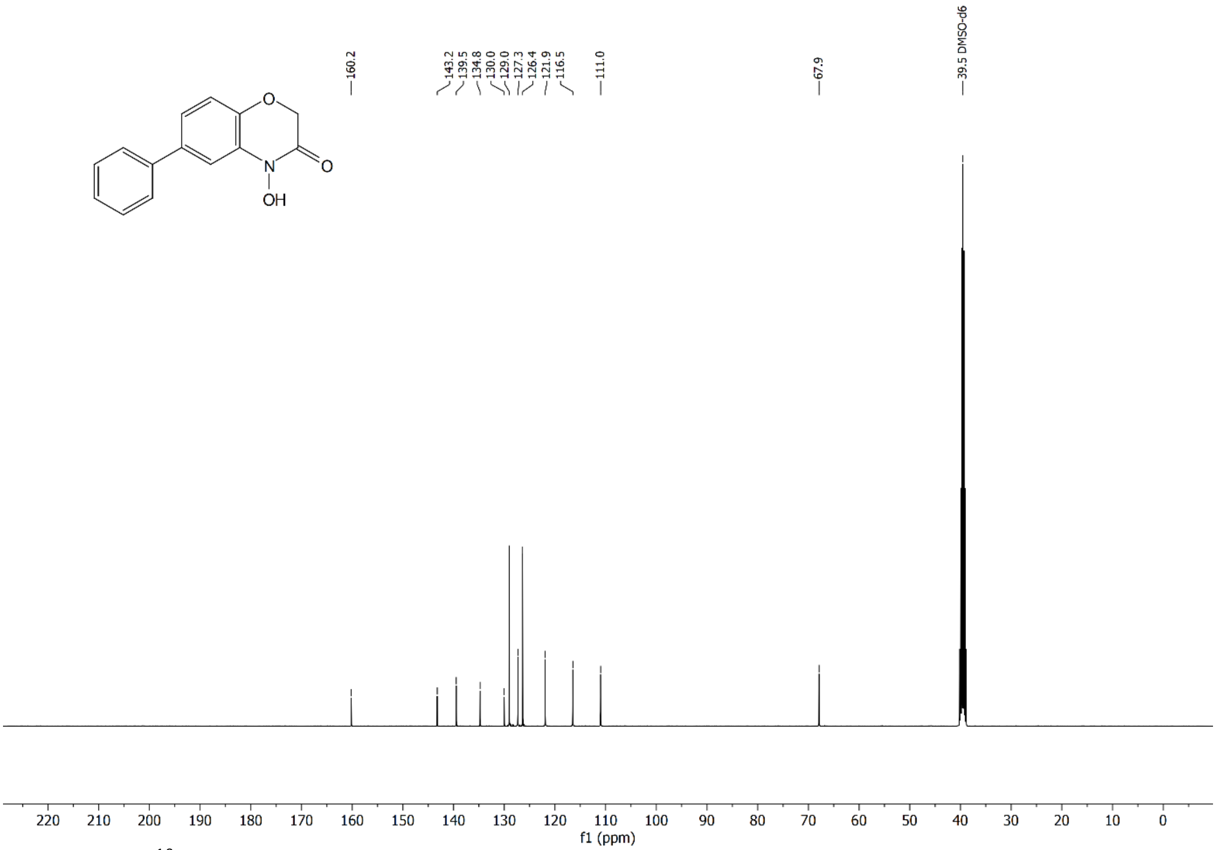

Figure S109: ^{13}C NMR spectrum (101 MHz, $DMSO_{d6}$) of **5l**.

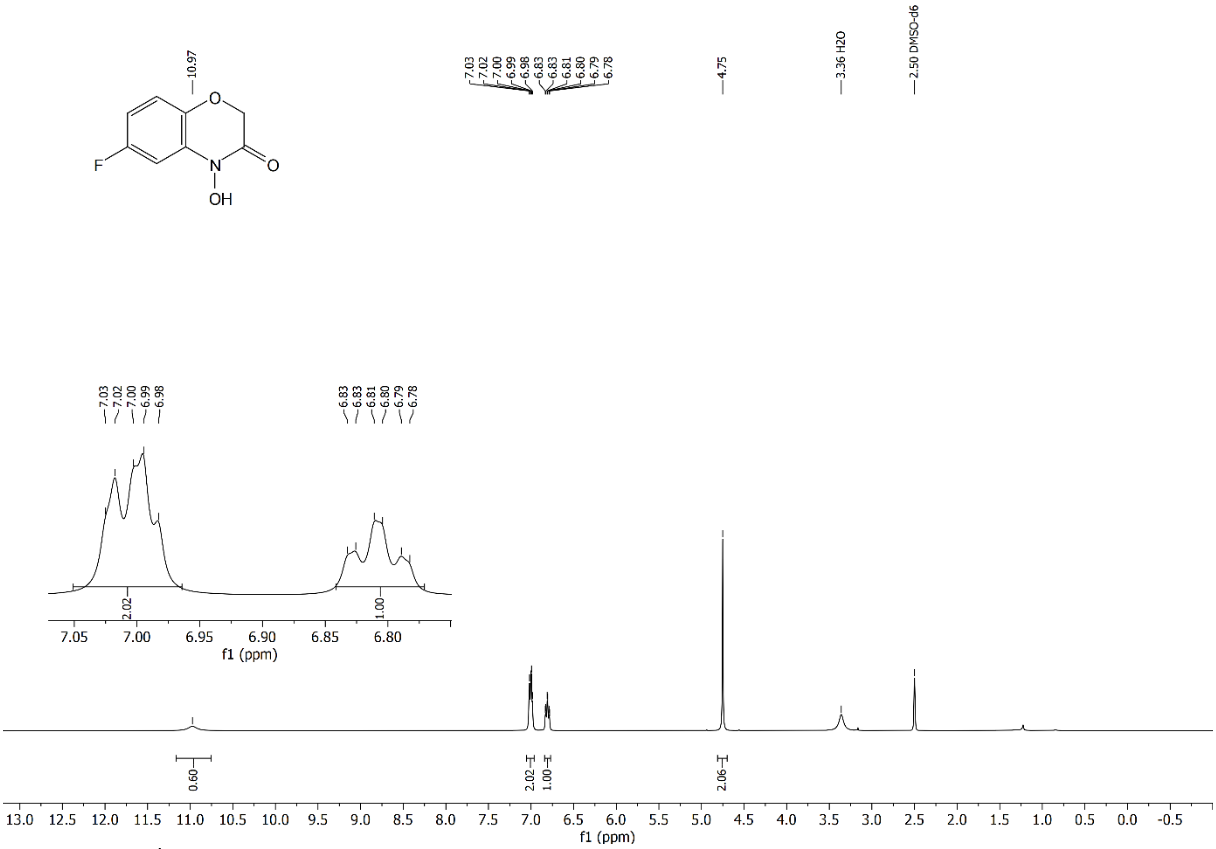

Figure S110: ^{1}H NMR spectrum (400 MHz, DMSO$_{d6}$) of **5m**.

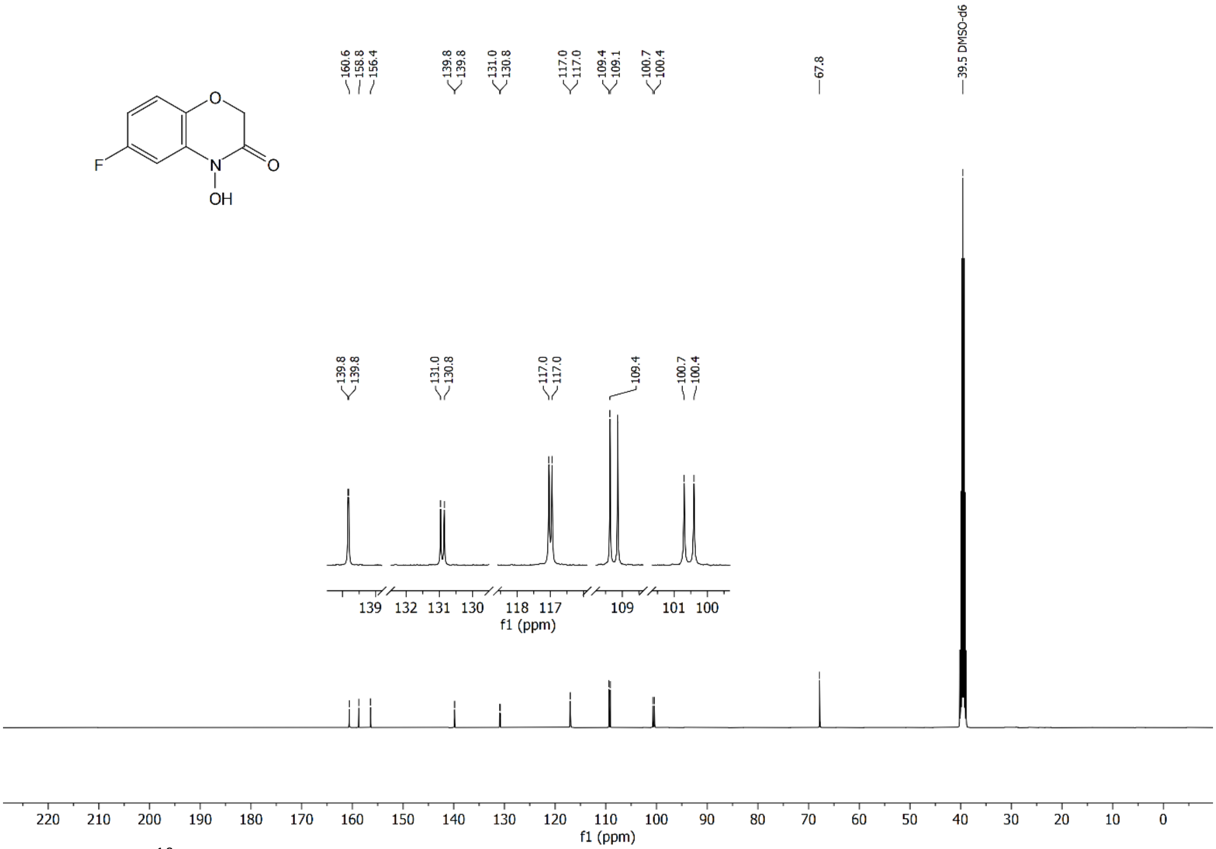

Figure S111: ^{13}C NMR spectrum (101 MHz, DMSO$_{d6}$) of **5m**.

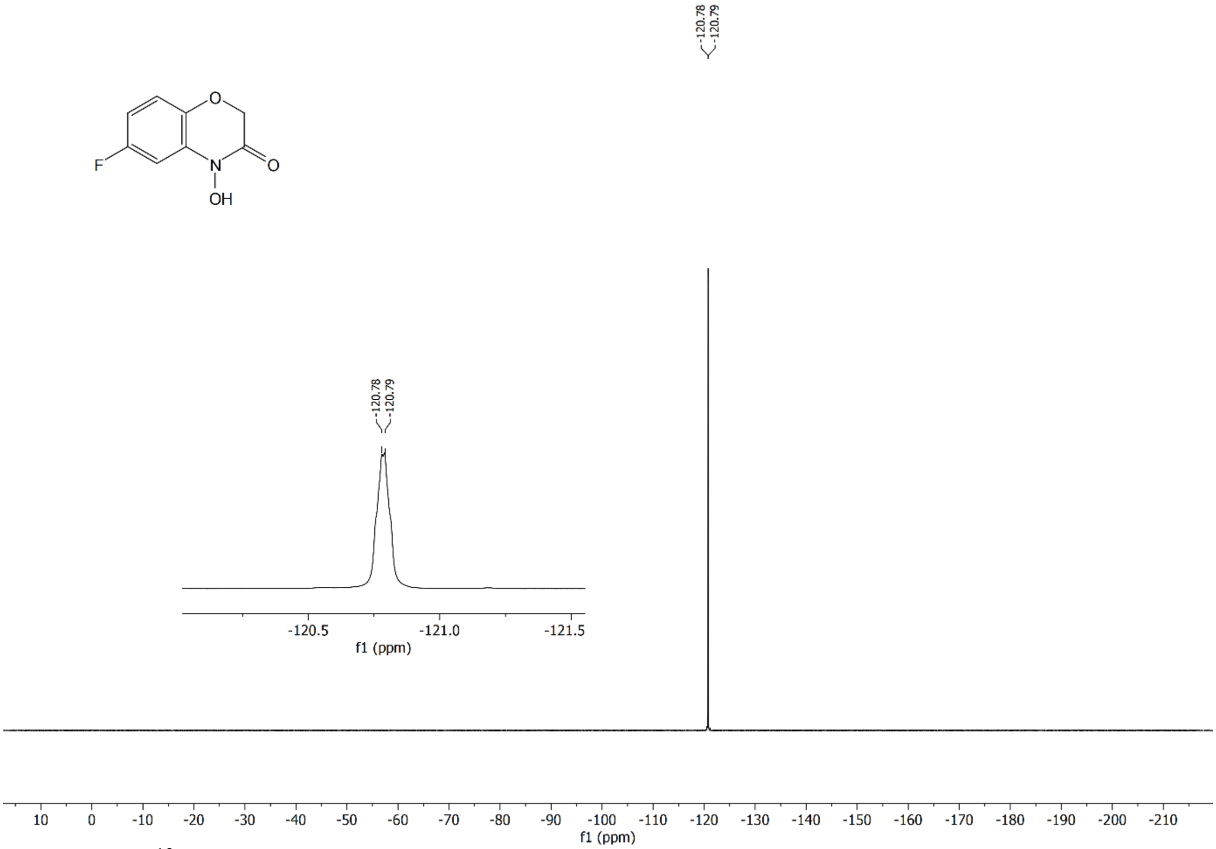

Figure S112: ^{19}F NMR spectrum (376 MHz, $DMSO_{d6}$) of **5m**.

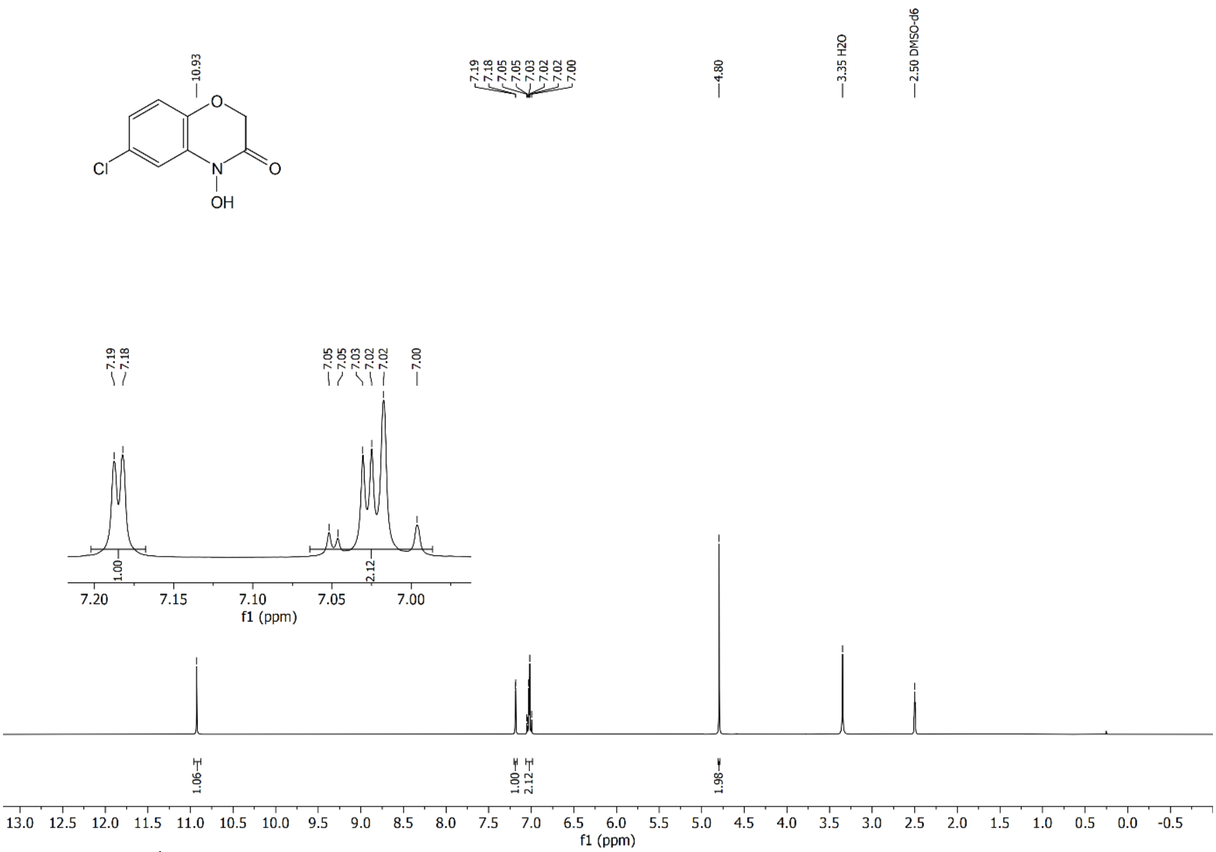

Figure S113: ^{1}H NMR spectrum (400 MHz, $DMSO_{d6}$) of **5n**.

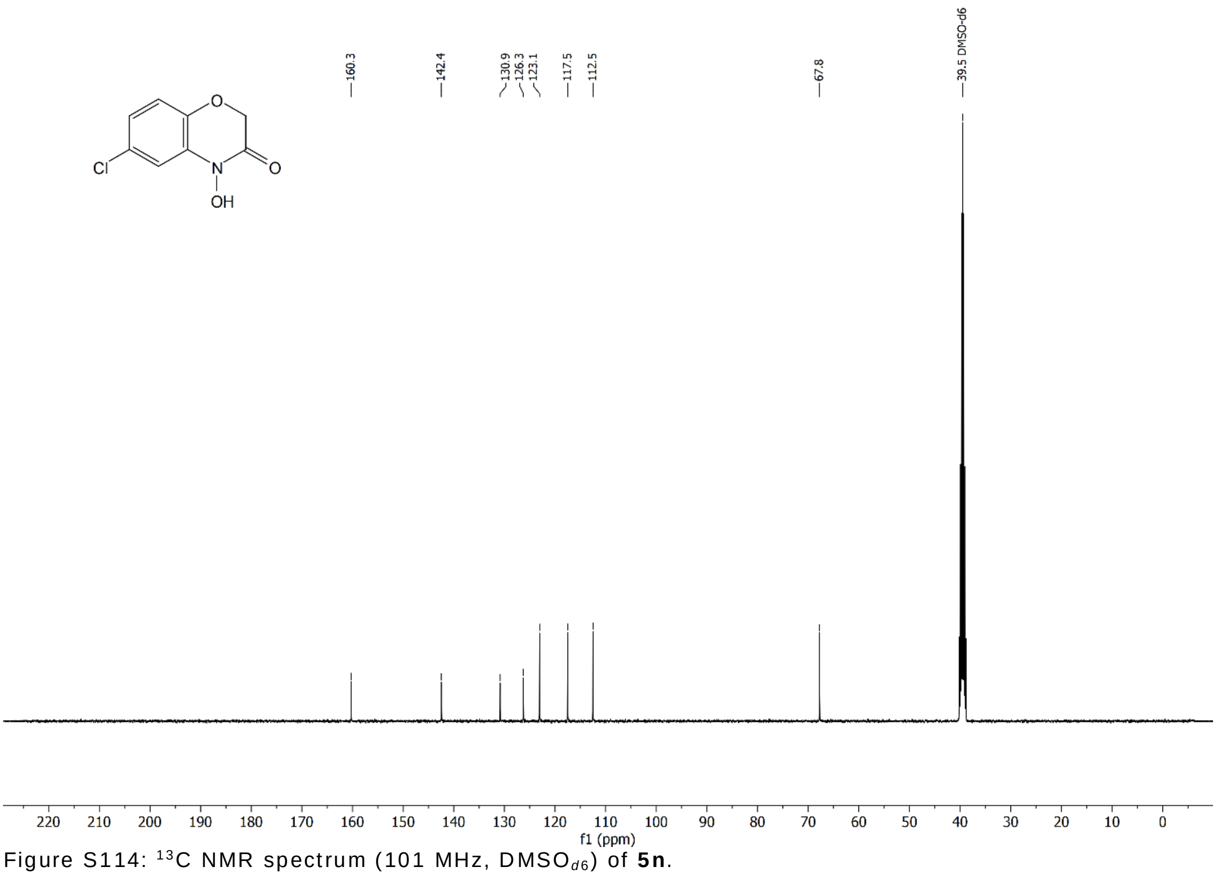

Figure S114: ^{13}C NMR spectrum (101 MHz, $DMSO_{d6}$) of **5n**.

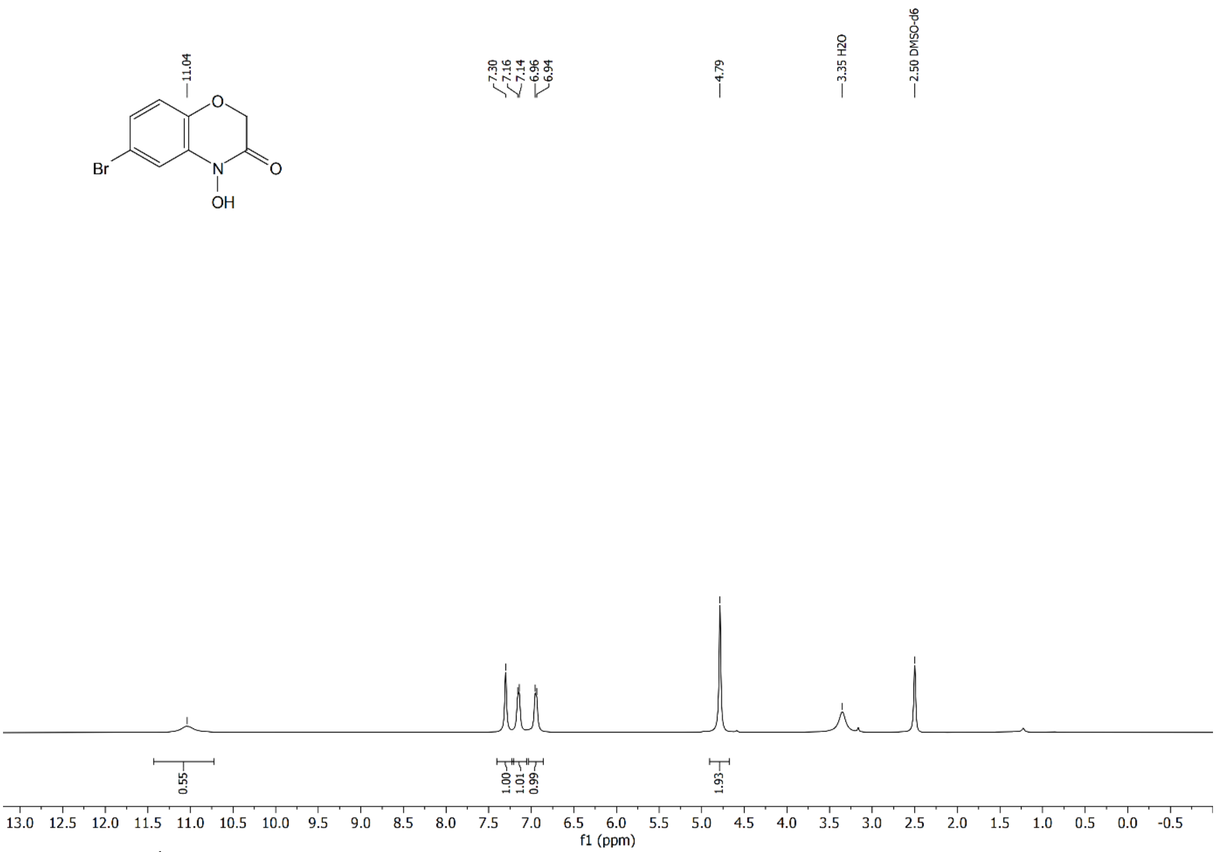

Figure S115: ^{1}H NMR spectrum (400 MHz, DMSO$_{d6}$) of **5o**.

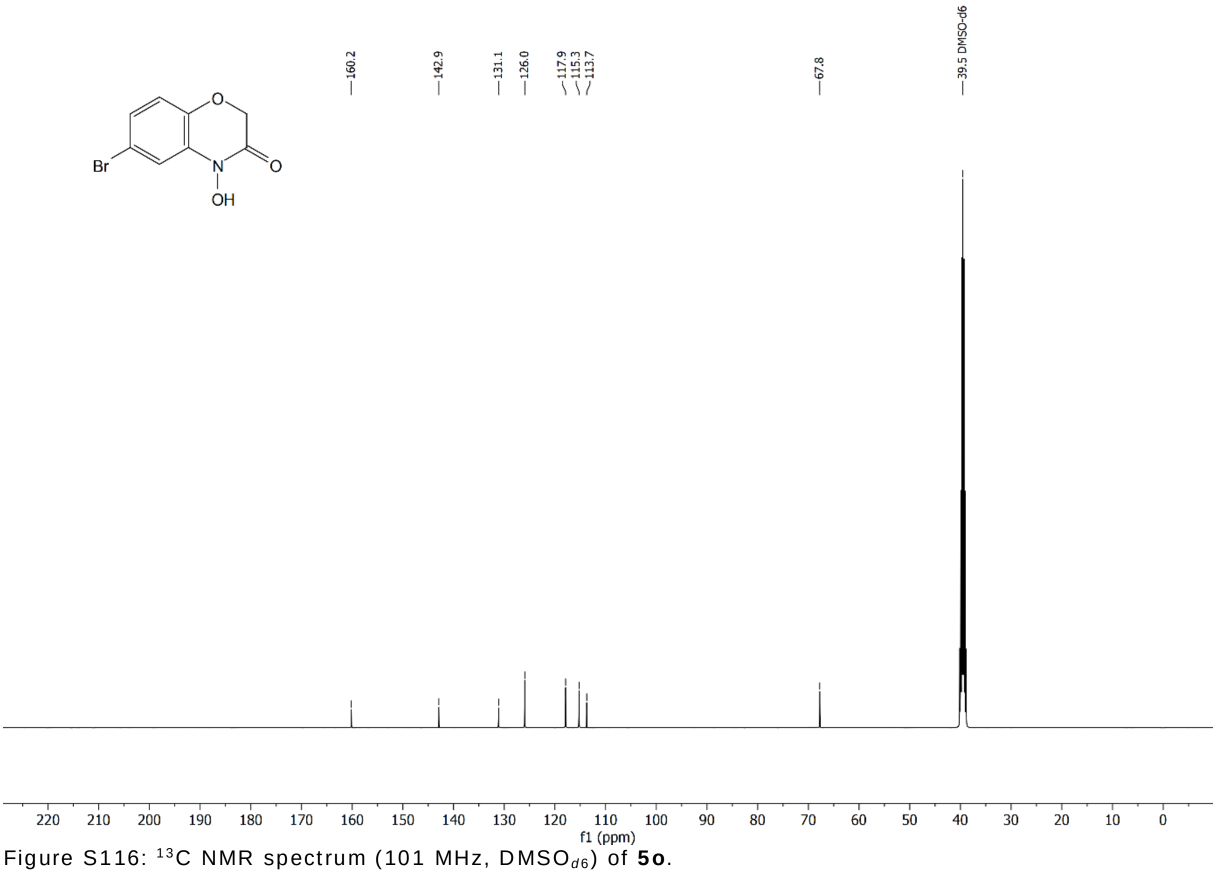

Figure S116: ^{13}C NMR spectrum (101 MHz, DMSO$_{d6}$) of **5o**.

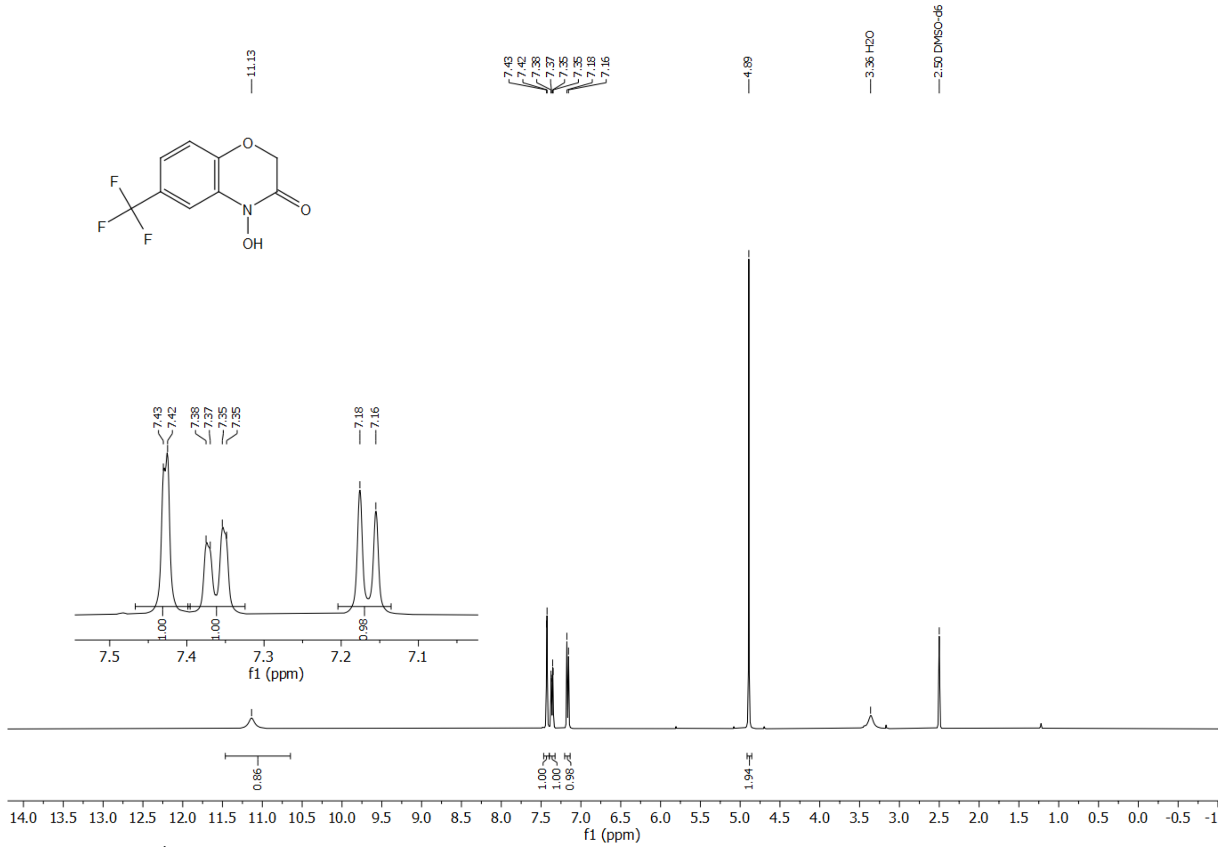

Figure S117: ^{1}H NMR spectrum (400 MHz, $DMSO_{d6}$) of **5p**.

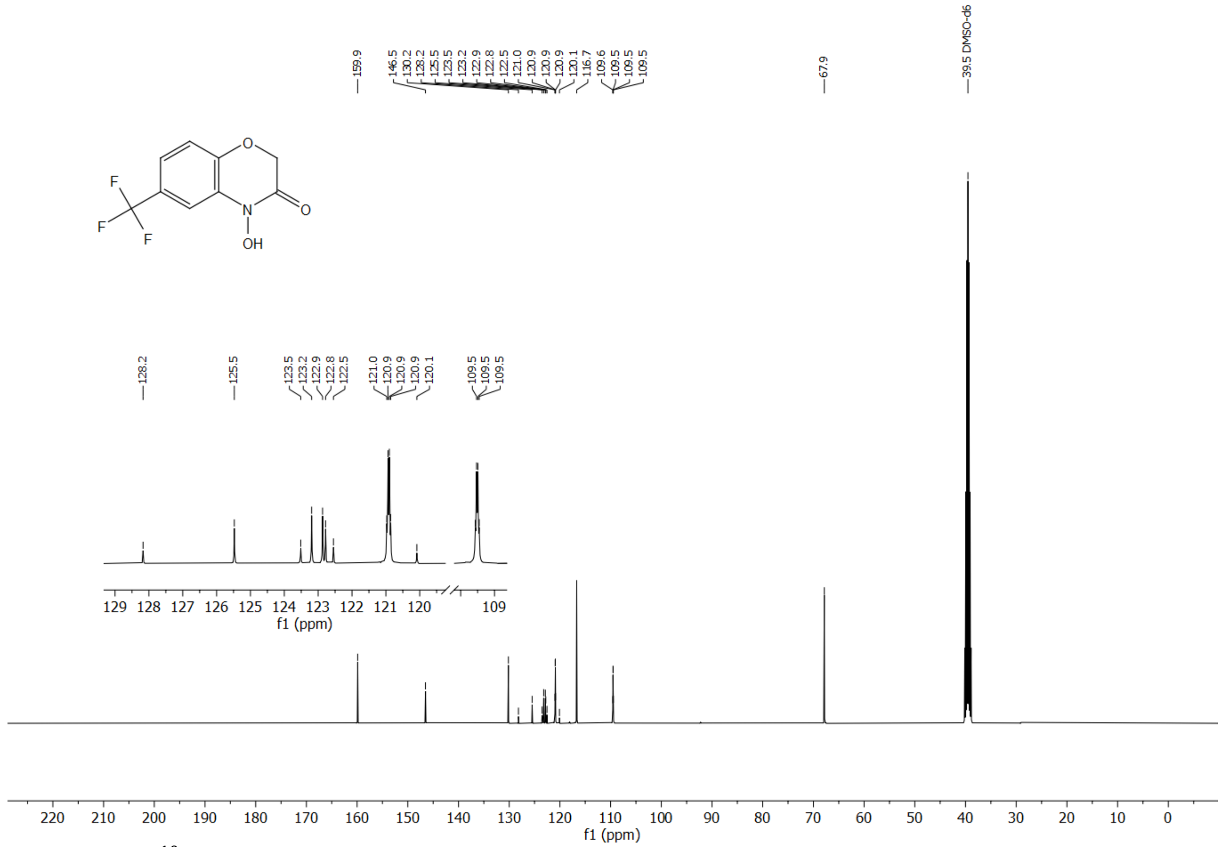

Figure S118: ^{13}C NMR spectrum (101 MHz, $DMSO_{d6}$) of **5p**.

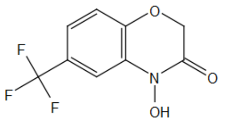

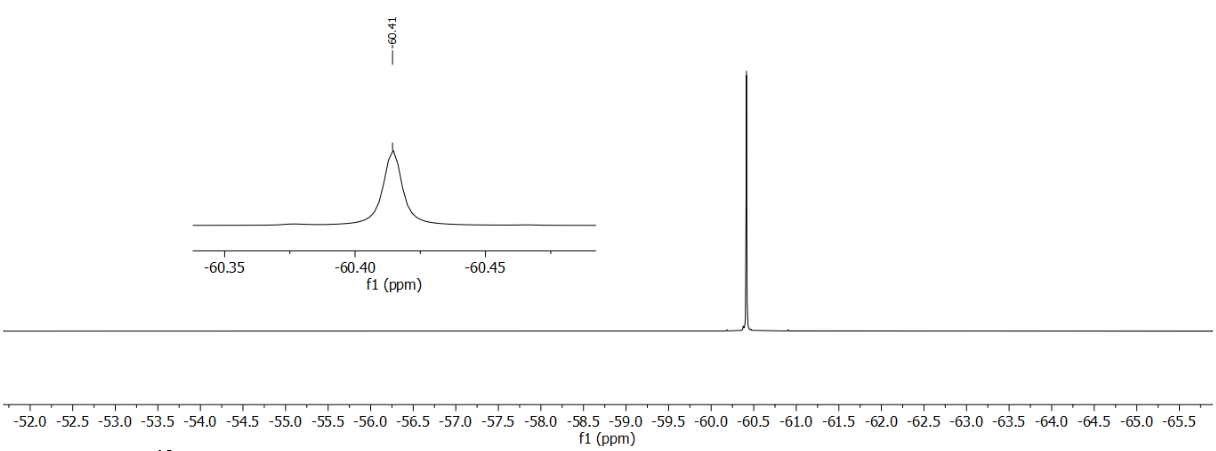

Figure S119: ^{19}F NMR spectrum (376 MHz, $DMSO_{d6}$) of **5p**.

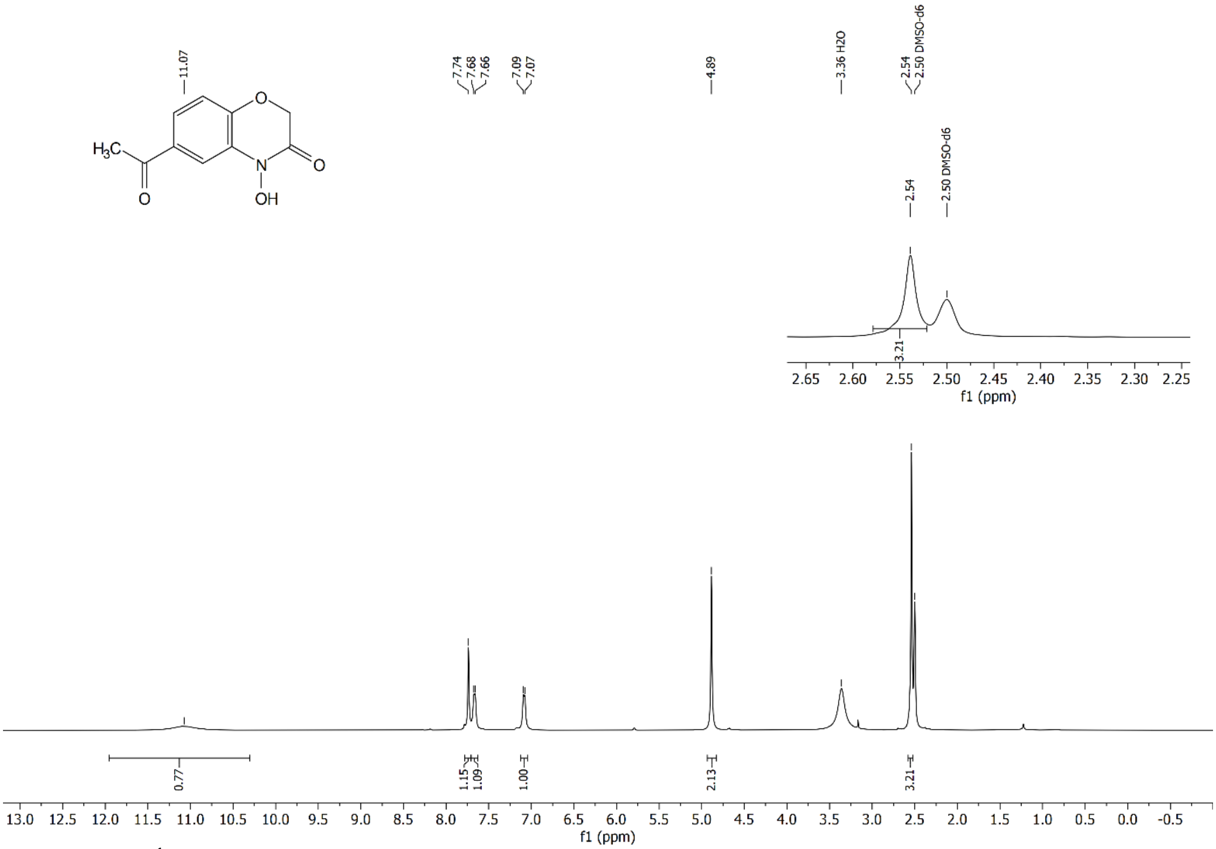

Figure S120: ^{1}H NMR spectrum (400 MHz, $DMSO_{d6}$) of **5q**.

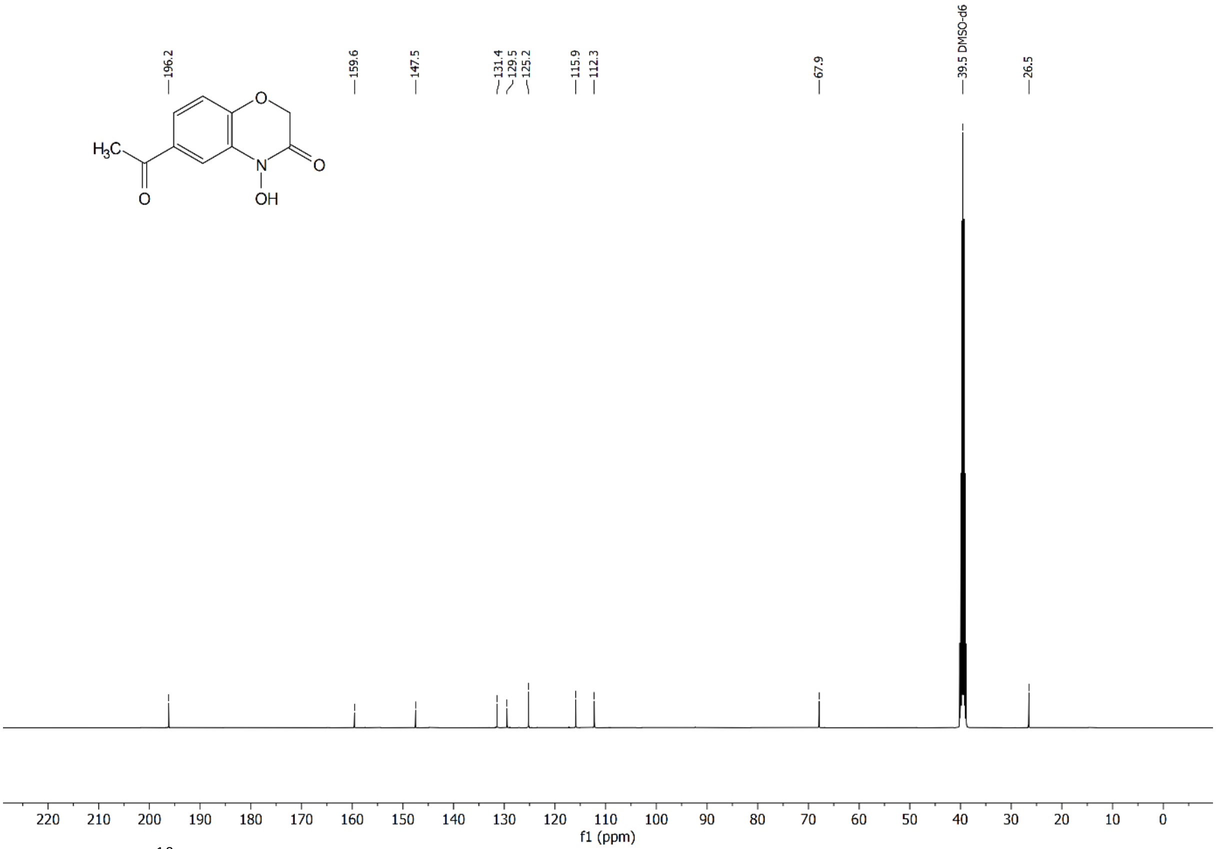

Figure S121: ^{13}C NMR spectrum (101 MHz, $DMSO_{d6}$) of **5q**.

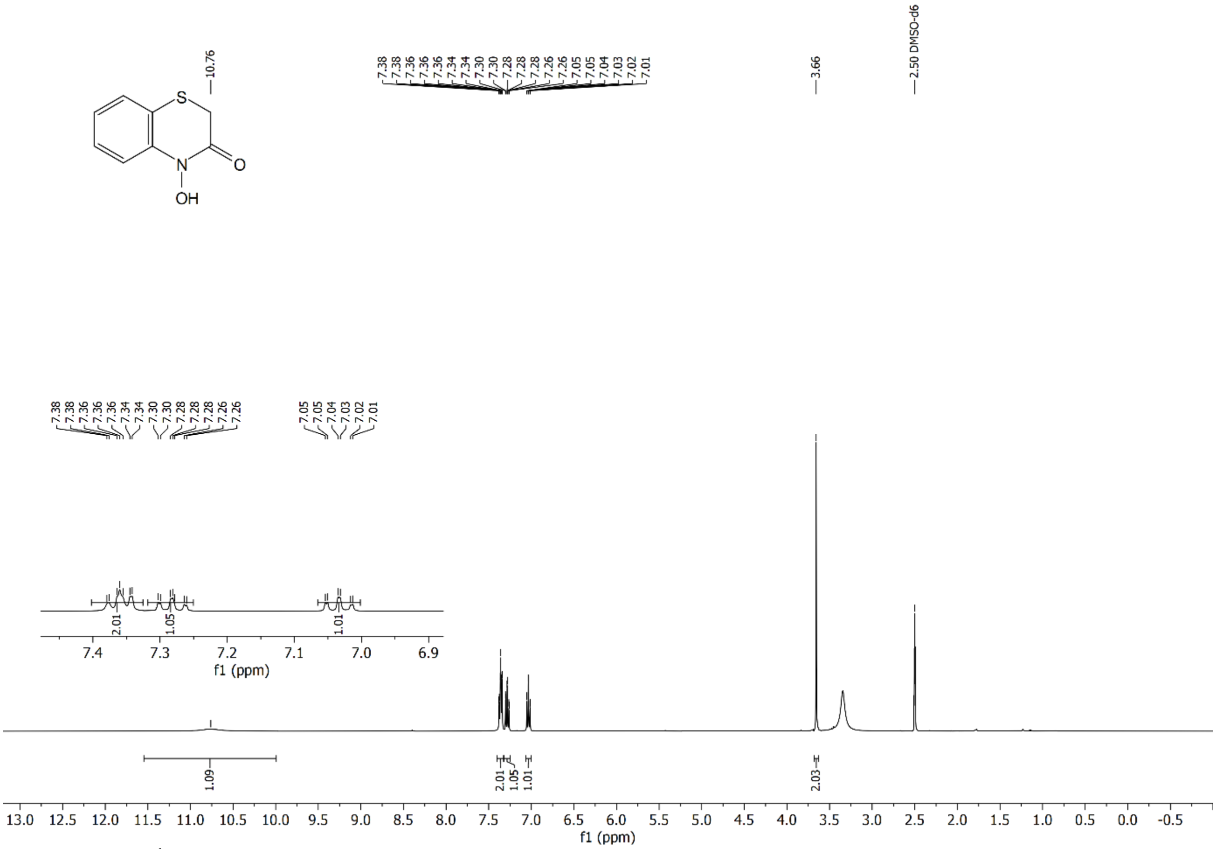

Figure S122: ¹H NMR spectrum (400 MHz, DMSO$_{d6}$) of **5r**.

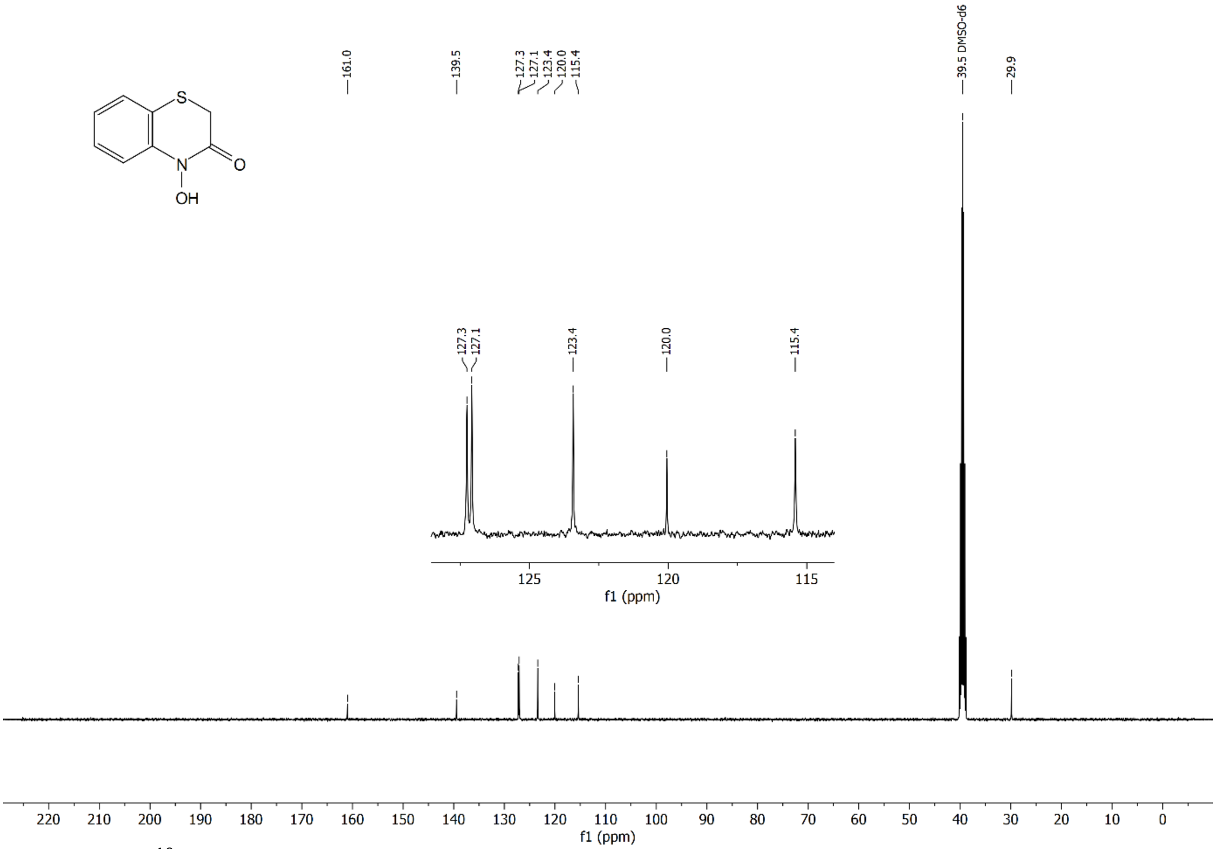

Figure S123: ¹³C NMR spectrum (101 MHz, DMSO$_{d6}$) of **5r**.

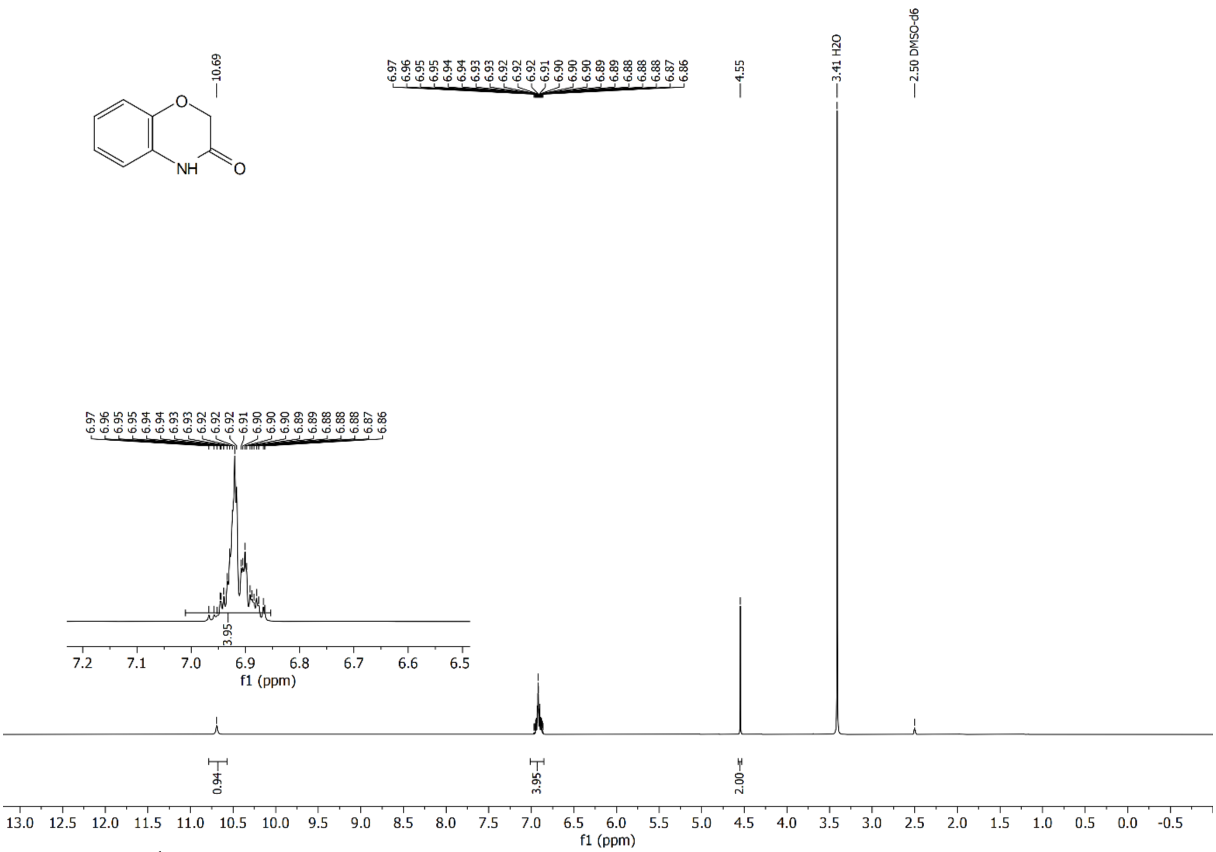

Figure S124: ^{1}H NMR spectrum (400 MHz, DMSO$_{d6}$) of **5s**.

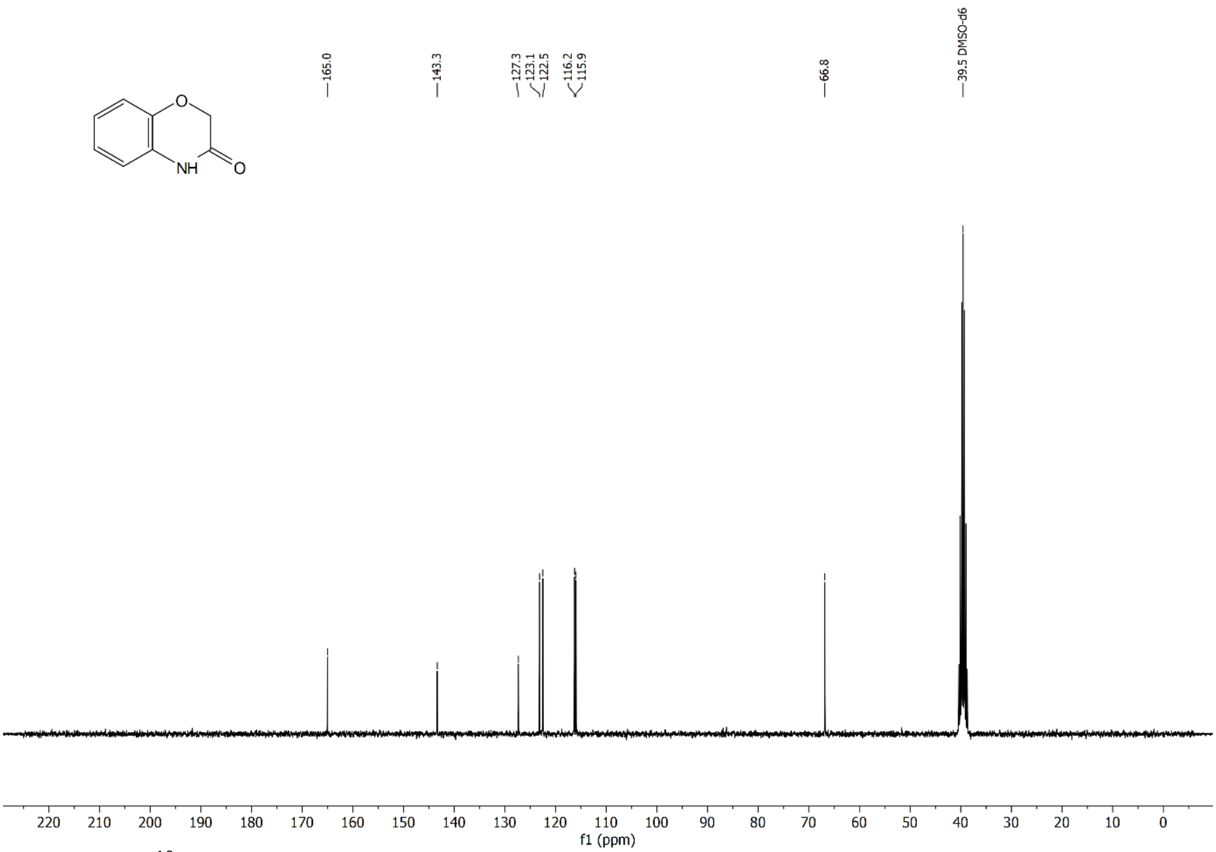

Figure S125: ^{13}C NMR spectrum (101 MHz, DMSO$_{d6}$) of **5s**.

9. References

1 D. Bindl, E. Heinemann, P. K. Mandal and I. Huc, Quantitative helix handedness bias through a single H vs. CH3 stereochemical differentiation, *Chem. Commun.*, 2021, **57**, 5662–5665.
2 C. Empel, S. Jana, C. Pei, T. V. Nguyen and R. M. Koenigs, Photochemical O-H Functionalization of Aryldiazoacetates with Phenols via Proton Transfer, *Org. Lett.*, 2020, **22**, 7225–7229.
3 D. Bindl, P. K. Mandal, L. Allmendinger and I. Huc, Discrete Stacked Dimers of Aromatic Oligoamide Helices, *Angew. Chem., Int. Ed.*, 2022, **61**, e202116509.
4 Y. Zhang, S. Sun, Y. Su, J. Zhao, Y.-H. Li, B. Han and F. Shi, Deconstructive di-functionalization of unstrained, benzo cyclic amines by C-N bond cleavage using a recyclable tungsten catalyst, *Org. Biomol. Chem.*, 2019, **17**, 4970–4974.
5 H. I. E.-Subbagh, A. H. Abadi, L. E. Al-Khawad and K. A. Al-Rashood, Synthesis and Antitumor Activity of Some New Substituted Quinolin-4-one and 1,7-Naphthyridin-4-one Analogs, *Arch. Pharm.*, 1999, **332**, 19–24.
6 J. Atkinson, P. Morand, J. T. Arnason, H. M. Niemeyer and H. R. Bravo, Analogs of the cyclic hydroxamic acid 2,4-dihydroxy-7-methoxy-2H-1,4-benzoxazin-3-one (DIMBOA): decomposition to benzoxazolinones and reaction with beta.-mercaptoethanol, *J. Org. Chem.*, 1991, **56**, 1788–1800.
7 S. Ozden, A. M. Oztürk, H. Göker and N. Altanlar, Synthesis and antimicrobial activity of some new 4-hydroxy-2H- 1,4-benzoxazin-3(4H)-ones, *Farmaco*, 2000, **55**, 715–718.
8 F. A. Macías, D. Marín, A. Oliveros-Bastidas and J. M. G. Molinillo, Optimization of benzoxazinones as natural herbicide models by lipophilicity enhancement, *J. Agric. Food Chem.*, 2006, **54**, 9357–9365.
9 F. A. Macías, J. M. de Siqueira, N. Chinchilla, D. Marín, R. M. Varela and J. M. G. Molinillo, New herbicide models from benzoxazinones: aromatic ring functionalization effects, *J. Agric. Food Chem.*, 2006, **54**, 9843–9851.
10 H. Matschiner, H. Tanneberg and C.-P. Maschmeier, Elektrosynthese cyclischer Hydroxamsäuren mit anschließender analoger Bamberger-Umlagerung, *J. Prakt. Chem.*, 1981, **323**, 924–926.
11 M. Hou, Z. Zhang, X. Lai, Q. Zong, M. Ren, T. Bai and G. Qiu, Photo-Induced Electrophilic Aromatic Substitution of Ferric Acyl Nitrene, *Synthesis*, 2024, **56**, 496–506.
